# Exploring the additive antibacterial potential of *Cinnamomum cassia* volatile oil and imipenem against *Acinetobacter baumannii*: a multi-omics investigation

**DOI:** 10.3389/fmicb.2025.1578322

**Published:** 2025-07-02

**Authors:** Youyuan Lu, Wanlin Xu, Jiahui Xue, Mingxia Xie, Haotian Liu, Ruilin Wang, Gang Li, Hanqing Wang

**Affiliations:** ^1^College of Pharmacy, General Hospital of Ningxia Medical University, Ningxia Medical University, Yinchuan, China; ^2^Key Laboratory of Protection, Development and Utilization of Medicinal Resources in the Liupanshan Area, Ministry of Education, Ningxia Engineering and Technology Research Center for Modernization of Regional Characteristic Traditional Chinese Medicine, Ningxia Medical University, Yinchuan, China; ^3^College of Traditional Chinese Medicine, Hunan University of Chinese Medicine, Changsha, China

**Keywords:** *Acinetobacter baumannii*, volatile oil of *Cinnamomum cassia*, antimicrobial activity, antibiotic agents, imipenem

## Abstract

**Introduction:**

*Acinetobacter baumannii* has been identified as a critical pathogen, and new antibiotics are urgently needed. Volatile oils, which function as natural antibacterial agents, may provide an effective means of inhibiting *A. baumannii*. However, the antibacterial activity and mechanism of the volatile oil derived from the dried bark of *Cinnamomum cassia* (CBV), as well as its additive effect when combined with imipenem (IPM) against *A. baumannii,* remain unclear.

**Methods:**

CBV was extracted using the hydrodistillation method and characterized by gas chromatography–mass spectrometry (GC–MS) analysis. The minimum inhibitory concentrations (MICs) of CBV and IPM against *A. baumannii* were determined using the microdilution method. A checkerboard assay was performed to evaluate the additive effect of CBV (concentration range: 0–1 μL/mL) and IPM (concentration range: 0–256 μg/mL) against *A. baumannii*, with the fractional inhibitory concentration index (FICI) calculated. A time-kill curve analysis was performed to assess the additive effect of CBV (0.125 μL/mL) and IPM (4 μg/mL) against *A. baumannii*. Antibiofilm activity was evaluated using a crystal violet staining assay. Cell membrane integrity was assessed using SYTO 9/PI staining based on fluorescence color. Intracellular protein levels were quantified using a BCA kit according to the manufacturer’s instructions. Scanning electron microscopy (SEM) was used to observe morphological changes in *A. baumannii*. Additionally, the antibacterial mechanism was elucidated through a combination of transcriptomic and proteomic analyses.

**Results:**

An additive effect (FICI = 0.53) was observed when CBV and IPM were combined against *A. baumannii*, reducing the MIC of IPM from 256 μg/mL to 4 μg/mL. CBV and IPM inhibited biofilm formation, damaged the cell membrane, and induced intracellular protein leakage in *A. baumannii*. Compared to CBV or IPM alone, the combination group (at the dosage showing an additive effect) caused significantly greater damage to the cell membrane of *A. baumannii*. CBV and IPM also induced significant changes at both the transcriptomic and proteomic levels in *A. baumannii*. Functional analysis revealed that the differentially expressed genes (DEGs) and proteins (DEPs) were involved in multiple pathways. Both CBV and IPM contributed to the observed antibacterial activity. CBV primarily influenced the ribosome pathway, while IPM mainly influenced oxidative phosphorylation. In the combination treatment, the simultaneous targeting of the ribosome and oxidative phosphorylation pathways was identified as the key antibacterial mechanism.

**Conclusion:**

This study demonstrated that the combination of CBV and IPM exhibits promising antimicrobial activity against *A. baumannii*, suggesting that CBV could serve as a potential natural candidate for the development of novel antibiotic agents. While the current findings establish a mechanistic foundation for CBV’s antimicrobial effects, further research is necessary to facilitate its clinical translation. Specifically, formulation optimization studies are necessary to enhance the therapeutic viability of the CBV/IPM combination, and comprehensive *in vivo* investigations are crucial to validate the antibacterial efficacy and safety profile of CBV/IPM prior to clinical application.

## Introduction

Antimicrobial-resistant pathogens are an emerging global problem that poses a serious threat to human health (Gou et al., 2023). *Acinetobacter baumannii*, a member of the ESKAPE group of pathogens, has shown a high prevalence of multidrug resistance (MDR), with approximately 45% of isolates classified as MDR (Oliveira et al., 2020). As a major cause of nosocomial infections, *A. baumannii* is responsible for respiratory tract and bloodstream infections in critically ill patients. In addition, it contributes to skin and soft tissue infections at surgical sites, as well as catheter-associated urinary tract infections, posing a significant clinical concern (Harding et al., 2017; Lucidi et al., 2024). Mortality rates associated with *A. baumannii* infection generally ranged from 12 to 50% (Novovic and Jovcic, 2023). Historically, fluoroquinolones, piperacillin-tazobactam, and carbapenems have been used for the treatment of infections caused by *A. baumannii*, but their widespread use has contributed to significant resistance, particularly against *β*-lactam antibiotics (Appaneal et al., 2022; Novovic and Jovcic, 2023). As a result, *A. baumannii* has been identified as a critical priority pathogen, and the urgent need for new antibiotics is well recognized (Ayoub Moubareck and Hammoudi Halat, 2020).

The dried bark of *Cinnamomum cassia* Presl. (CB), known as cinnamon, is widely used as a food spice and medicine (Liu et al., 2023; Pages-Rebull et al., 2024). Used as a spice, it serves as an aromatic condiment, natural preservative, and flavoring agent (Pages-Rebull et al., 2024). Used as a traditional Chinese medicine, it was employed to treat dizziness, vomiting, diarrhea, arthritis, and other conditions (Liu et al., 2023). Volatile oils, flavonoids, phenylpropanoids, polyphenols, terpenoids, and other compounds comprise the chemical compound library of CBV (Fan et al., 2024). Volatile oil derived from the dried bark of *Cinnamomum cassia* Presl. (CBV), one of the primary bioactive extracts of CB, exhibits a wide range of pharmacological activities, including antibacterial, anti-inflammatory, antidiabetic, and antioxidant properties (Liu et al., 2024; Liu et al., 2021; Sharifi-Rad et al., 2021). As natural antibacterial agents, volatile oils are a complex mixture of various biologically active compounds (Wang et al., 2024). Due to their synergistic effects, volatile oils exhibit broad-spectrum antibacterial, antifungal, and antiviral activities (Tariq et al., 2019). Thus, volatile oils may serve as an effective agent for inhibiting drug-resistant microbes (Hou et al., 2022). Previous studies have demonstrated that CBV and its emulsion efficiently inhibit *A. baumannii* (Ganić et al., 2022). In this study, we hypothesize that the combination of CBV and imipenem (IPM) may exhibit synergistic effects against *A. baumannii*.

Thus, in this study, to comprehensively reveal the antibacterial activity and mechanism of CBV combined with IPM on *A. baumannii*, microdilution, checkerboard, and time-kill assays were performed to elucidate the antibacterial activity. Biofilm inhibition, cell membrane integrity, microscopy characteristics, and intracellular protein content were also investigated. Moreover, the potential antimicrobial mechanism of combining CBV with IPM against *A. baumannii* was explored through the combined use of transcriptomics and proteomics. This study provides a novel strategy and useful information against *A. baumannii*.

## Materials and methods

### Bacterial strains and reagents

*Acinetobacter baumannii* was isolated from the sputum of a patient at the General Hospital of Ningxia Medical University, China. The strain was identified using the VITEK2 Compact automatic microbial analyzer (BioMerieux, Paris, France), mixed with skim milk, and stored at −80°C. IPM was purchased from Dalian Meilun Biotechnology Co., Ltd. (Dalian, China). The SYTO9/PI Live/Dead Bacterial Double Stain Kit was obtained from Shanghai Fushen Biotechnology Co., Ltd. (Shanghai, China). A 0.1% crystal violet aqueous solution was purchased from Beijing Biotopped Technology Co., Ltd. (Beijing, China). The BCA protein assay kit was obtained from Beijing Ranjke Technology Co., Ltd. (Beijing, China). All other reagents were of analytical grade.

### Preparation of CBV

Hydrodistillation is a traditional method used for extracting volatile oils due to its affordability, simplicity, and scalability (Li et al., 2024). To obtain CBV, a hydrodistillation method was established. Briefly, a total of 300 g of CB powder (40 mesh) was soaked in 3,000 mL of distilled water for 1 h and then subjected to hydrodistillation for 2 h using a Clevenger-type apparatus. The natural oils were dried with anhydrous sodium sulfate and stored at 4°C. GC–MS was used to analyze the chemical composition of CBV.

### GC–MS analysis

GC–MS analyses were conducted using an Agilent 7890B-5977A GC–MS system (Agilent Technologies, USA) following the method of Li S. F. et al. (2022). The identification of peaks was based on NIST databases, and the relative percentage values of each constituent were calculated using the TIC from MS.

### Determination of MIC

The minimum inhibitory concentrations (MICs) of CBV and IPM against *A. baumannii* were determined using the microdilution method, following CLSI guidelines. Briefly, the strain was pre-cultured in Mueller-Hinton Broth (MHB) at 200 rpm and 37°C overnight, after which the cultures were diluted to 1.0 × 10^6^ CFU/mL in MHB. The following solutions were added sequentially to 96-well microtiter plates: 50 μL of MHB solution, 50 μL of test solution serially diluted in wells containing MHB, except for the positive growth control, and 50 μL of bacterial suspension solution, except in the negative control. Then, the microplate was incubated for 24 h at 37°C in 5% CO_2_. In this process, CBV was dissolved in 1% Tween 80 at a starting concentration of 32 μL/mL, based on preliminary experiments. The positive growth control contained MHB, the bacterial suspension, and either distilled water or 1% Tween 80 (depending on the solvent used to dissolve the drugs). The negative growth control contained only MHB solution and distilled water or 1% Tween 80. After incubation, 2% 2,3,5-triphenyltetrazolium chloride (TTC) was added, and the plate was incubated at 37°C for 10 min. The MIC was defined as the lowest concentration at which no visible red formazan was observed. The experiment was conducted in triplicate to ensure the reliability of the results.

### Checkerboard assay

The checkerboard assay was used to evaluate the combined antimicrobial effect of CBV and IPM. Briefly, a series of twofold dilutions of CBV and IPM were mixed in each well of 96-well microtiter plates. The concentrations of CBV and IPM in the 96-well microtiter plates ranged from 0 to 1 μL/mL and from 0 to 256 μg/mL, respectively. The MIC was assessed in the same manner as described above. The fractional inhibitory concentration index (FICI) was used to evaluate the combination effects with the following criteria: FICI≤0.5 denoting synergism; 0.5 < FICI≤1 denoting an additive effect; 1 < FICI≤2 denoting no interaction; and FICI>2 denoting antagonism (Asadi et al., 2023). The minimum FICI combination was named the combination group.

### Time-kill curve analysis

A time-kill curve analysis was conducted to evaluate the additive effect of CBV and IPM against *A. baumannii,* according to the method reported by Chan et al. (2011), with some modifications. Briefly, an overnight MHB culture was diluted to obtain strains with a concentration of approximately 10^6^ CFU/mL. CBV, IPM, or their combination was added to the bacterial MHB. The CBV group received CBV at a final concentration of 0.125 μL/mL. The IPM group received IPM at a final concentration of 4 μg/mL. The combination group received both CBV (final concentration 0.125 μL/mL) and IPM (final concentration 4 μg/mL). At 0, 1, 2, 4, 8, 12, and 24 h of incubation, 10 μL of bacterial culture aliquots were collected from each group, diluted 1,000-fold in PBS, and 10 μL of the diluted solution was plated onto MAC agar plates and incubated at 37°C for 24 h. The number of colonies was counted, and the mean colony counts (log CFU/mL) were used to construct the time-killing curves.

### Biofilm inhibition assay

A crystal violet staining assay was used to evaluate the antibiofilm activity (Li S.-F. et al., 2022; Li W. et al., 2022). Bacterial samples with a concentration of 10^6^ CFU/mL were prepared. The assay was performed in 96-well microtiter plates, with each well containing the following components: 50 μL of MHB, 50 μL of the test agents (CBV at a final concentration of 0.125 μL/mL, IPM at 4 μg/mL, or their combination at both concentrations), and 100 μL of bacterial suspension. The plates were incubated at 37°C for 24 h. Controls included a positive control with distilled water replacing the test agent and a sterility control (negative control) containing only MHB without bacterial inoculum. After incubation, the culture solution was removed, and the wells were gently washed with PBS. According to the method described by Li S.-F. et al. (2022) and Li W. et al. (2022), methanol was not used to fix the biofilm. Instead, a 0.1% crystal violet solution was added to each well and incubated for 15 min. The crystal violet was discarded, and the wells were washed three times with PBS. The crystal violet-stained samples were dissolved in 33% acetic acid, and absorbance was measured at 570 nm. The biofilm formation index (BFI) was calculated based on the relative percentage absorbance compared to the positive control.

### Cell membrane integrity analysis

The effects of CBV, IPM, or their combination on *A. baumannii* membranes were tested using SYTO 9-PI double staining, as described in previous reports (Wang et al., 2022). The bacterial suspension was treated with CBV alone, IPM alone, or a combination of both. The concentrations of CBV, IPM, and the combination were the same as those used in the biofilm inhibition assay. A SYTO 9/PI mixture staining solution was added and incubated for 15 min at room temperature in the dark. A fluorescence microscope was used to observe the cell membrane. Samples were excited at 488 nm, with SYTO 9 dye emitting at 550 nm and PI dye emitting at 635 nm. Due to its high membrane permeability, SYTO 9 stains all bacteria green, whereas PI penetrates only damaged membranes, staining bacteria with compromised membranes red. When co-applied, PI competitively displaces SYTO9 in dead bacteria, resulting in a fluorescence shift from green to red, while live bacteria maintain green fluorescence. This differential staining allows the assessment of cell membrane integrity.

### Scanning electron microscopy (SEM) analysis

*Acinetobacter baumannii* was cultured in MHB medium with CBV alone, IPM alone, or a combination of both (using the same concentrations as in the biofilm inhibition assay) for 24 h at 37°C. The control group did not receive any test agents. Bacterial cells were collected by centrifugation at 3800 rpm for 15 min and washed with PBS. The bacterial cells were then fixed in a 3% glutaraldehyde solution for 2 h, followed by treatment with a 1% osmium tetroxide solution for 1–2 h and subsequently washed with sterile PBS. Then, ethanol solutions at different concentrations (30, 50, 70, 90, 100%) were used for gradient dehydration. The dehydrated bacterial cells were coated with a gold spray. A scanning electron microscope (JEOL, Japan) was used to observe the morphological changes in the bacterial cells.

### Determination of intracellular protein content

A 1% inoculum of *A. baumannii* was added to MHB containing CBV, IPM, or their combination (using the same concentrations as in the biofilm inhibition assay) and cultured at 37°C with shaking at 120 rpm. At 0, 8, 16, 24, 40, and 48 h, 3 mL of culture was collected and centrifuged at 3800 rpm for 10 min. The cells were washed with PBS, followed by the addition of bacterial lysis buffer. The mixture was incubated on ice for 20 min, and the supernatant was used to determine intracellular protein content using a BCA kit, following the manufacturer’s instructions.

### Transcriptomic analysis

*Acinetobacter baumannii* was cultured in MHB medium at 37°C until the OD_600_ reached 0.6. Four groups were established: the control group received PBS and 1% Tween; the CBV group received 0.125 μL/mL CBV and PBS; the IPM group received 4 μg/mL IPM and 1% Tween; and the combination group received 0.125 μL/mL CBV and 4 μg/mL IPM. All groups were incubated at 37°C for 4 h. Bacterial cells were then collected by centrifugation at 3,800 rpm for 15 min and washed with PBS. Samples were sent to Novogene Co., Ltd. (Beijing, China) for transcriptome sequencing. Each experiment was performed in triplicate.

Bowtie 2 was used to map the cleaned data to the reference analysis. DESeq2 was used to identify differentially expressed genes (DEGs) between the treatment and control groups. The thresholds for identifying significant DEGs were set at |log2FoldChange| > 0 and *p*-value < 0.05. Subsequently, Gene Ontology (GO) and the Kyoto Encyclopedia of Genes and Genomes (KEGG) analyses were conducted to categorize the differentially expressed genes (DEGs) into various functional groups.

### DIA proteomic analysis

Proteomic analysis was performed using DIA technology, following the same sample preparation as in the transcriptomic analysis. The bacterial cells were ground at low temperatures, then dissolved in SDT and 1/100 volume of DTT, followed by treatment with an ultrasonic ice bath for 5 min. Then, the solution was incubated at 95°C for 10 min, followed by an ice bath for 2 min, and then centrifuged for 15 min at 4°C (1,200 g). The supernatant was added to the IAM solution and incubated in the dark for 1 h. Approximately four volumes of precooled acetone were then added, and the mixture was kept for 2 h at −20°C. The samples were centrifuged at 4°C for 15 min at 1,200 g. The resulting precipitate was collected, washed with cold acetone, and dissolved in a dissolution buffer. Protein content was determined using the Bradford protein quantitative kit, and protein quality was assessed via SDS-PAGE gel electrophoresis. Sample trypsin treatment was performed according to the report by Zhang et al. (2016).

The samples were analyzed using an upgraded Vanquish Neo UHPLC system (Thermo Fisher, USA) coupled with a Thermo Orbitrap Exactive mass spectrometer. An ES906 C18 column (150 μm × 15 cm, 2 μm) equipped with a 174,500 C18 pre-column (5 mm × 300 μm, 5 μm) was used for the separation. The mobile phase was composed of A (0.1% formic acid in deionized water) and B (0.1% formic acid in 80% acetonitrile) with gradient elution (0–0.2 min, 96–95% A; 0.2–4 min, 95–25% A; 4–5.8 min, 25–35% A; 5.8–6.2 min, 35–1% A; 6.2–6.9 min, 1%A). The flow rate of the mobile phase was 2.5 μL/min, and the column temperature was maintained at 50°C. Mass spectrometry was performed using an ESI source, with the ion source voltage set to 1.9 kV. The mass spectrum was acquired in data-dependent mode. The first-level scanning range was m/z 380–980, and the second-level mass spectrum scanning range was m/z 150–2000.

DIA-NN software was used for the qualitative and quantitative analysis of peptides. Fold change and *p*-value were applied to identify significant differentially expressed proteins (DEPs). Gene Ontology (GO) and Kyoto Encyclopedia of Genes and Genomes (KEGG) analyses were conducted to categorize the DEPs into various functional groups.

### Statistical analysis

All experiments were conducted in triplicate. Results were expressed as mean ± standard deviation (SD). Data were analyzed using one-way analysis of variance (one-way ANOVA) in conjunction with Duncan’s test. GraphPad Prism 7.0 was used to plot the data figure. Statistical significance was defined as a *p*-value of <0.05.

## Results

### CBV composition

The CBV was a pale yellow oil primarily composed of 14 chemical components (Table 1). Based on NIST, 14 compounds were identified, accounting for 98.6% of the total components. The main compound in CBV was cinnamaldehyde, which constituted 64.7% of the composition, followed by *δ*-cadinene, *α*-cubebene, and α-copaene. The total composition of these four compounds reached 82.6%.

**Table 1:** The chemical composition of CBV.

No.	RT (Time)	Peak area ratio (%)	Compound name	Chemical formula	CAS no.
1	28.943	5.2	α-Copaene	C_15_H_24_	3856-25-5
2	29.031	6.3	α-Cubebene	C_15_H_24_	17699-14-8
3	34.967	1.6	γ-Muurolene	C_15_H_24_	30021-74-0
4	35.978	4.1	α-Muurolene	C_15_H_24_	10208-80-7
5	36.876	6.4	δ-Cadinene	C_15_H_24_	483-76-1
6	37.665	1.0	Cubebene	C_15_H_24_	29837-12-5
7	39.019	2.0	Calamenene	C_15_H_22_	72937-55-4
8	44.342	64.7	Cinnamaldehyde	C_9_H_8_O	14371-10-9
9	44.752	1.3	Palustrol	C_15_H_26_O	5986-49-2
10	44.877	1.4	Unknown	–	–
11	45.064	1.4	Epicubenol	C_15_H_26_O	19912-67-5
12	48.166	1.6	T-Muurolol	C_15_H_26_O	19912-62-0
13	48.468	0.9	δ-Cadinol	C_15_H_26_O	19435-97-3
14	54.564	2.1	2-Methoxycinnamaldehyde	C_10_H_10_O_2_	1504-74-1

A total of 14 chemical compounds were identified in CBV, with cinnamaldehyde being the main compound, accounting for 64.7% of the total composition.

### Combined antibacterial effect of CBV and IPM against *Acinetobacter baumannii*

The antibacterial effect of CBV or IPM against clinically isolated *A. baumannii* was evaluated using a MIC assay, following the guidelines set forth by the Clinical and Laboratory Standards Institute (CLSI) in 2023. The MICs of CBV and IPM were 0.25 μL/mL and 256 μg/mL, respectively, indicating that the strain of *A. baumannii* was resistant to IPM, while CBV showed better antibacterial activity. The interaction between CBV and IPM was evaluated using a checkerboard assay. The results showed that the FICI was 0.53, indicating an additive effect (0.5 < FICI ≤ 1). The MIC of CBV was reduced twofold. A more significant reduction was observed for IPM in the presence of CBV, with its MIC decreasing by 32-fold. This suggests that at a concentration of 0.125 μL/mL CBV and 4 μg/mL IPM, CBV can enhance the antibacterial activity of IPM against IPM-resistant *A. baumannii*.

**Figure 1:**
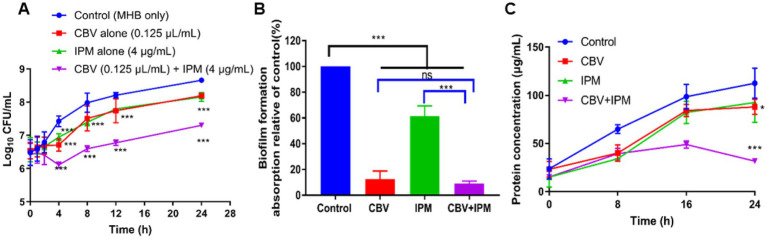
**(A)** Time-kill curves showing the activity of CBV (0.125 μL/mL) and IPM (4 μg/mL), tested alone and in combination, against *A. baumannii* in vitro. Compared to the control, ^***^p < 0.001. **(B)** Crystal violet staining assay evaluation of the anti-biofilm activity of CBV (0.125 μL/mL) and IPM (4 μg/mL), individually and in combination, against *A. baumannii*. ^***^p < 0.001; ns means *p* > 0.05. **(C)** Changes in intracellular protein content of *A. baumannii* following treatment with CBV (0.125 μL/mL), IPM (4 μg/mL), or their combination. Compared to the control, ^***^p < 0.001, ^*^p < 0.05.

To confirm the combined antibacterial effect of CBV and IPM against *A. baumannii*, a time-kill analysis was performed. After 24 h of incubation, all treatment groups significantly inhibited the growth of *A. baumannii* (*p* < 0.01; Figure 1A). Treatment with CBV (0.125 μL/mL) and IPM (4 μg/mL) alone showed the same antibacterial activity, which was observed from 4 to 24 h. In contrast, the combination group (CBV + IPM) showed antibacterial activity from 2 to 24 h. Compared to CBV and IPM alone, the combination treatment demonstrated significantly enhanced antibacterial activity against *A. baumannii*, confirming the additive effect of CBV and IPM.

These results demonstrated that CBV significantly reduced the effective concentration of IPM and enhanced its antibacterial activity against *A. baumannii*. To illustrate the antibacterial activity of the combination, the following studies used a CBV concentration of 0.125 μL/mL and an IPM concentration of 4 μg/mL.

### Effects of CBV and IPM on *Acinetobacter baumannii* biofilm synthesis

To evaluate the effects of CBV and IPM on *A. baumannii* biofilm formation, crystal violet staining was performed (Figure 1B). Following treatment with IPM (4 μg/mL), the biofilm formation ratio was 61.4%, which was significantly lower than that of the control group (*p* < 0.001). Treatment with CBV (0.125 μL/mL) reduced the biofilm formation ratio to 12.7%. Using the CBV-IPM combination, biofilm formation decreased to 9.1%, which was significantly lower than with IPM alone (*p* < 0.01) but not significantly different from CBV alone (*p* > 0.05). These results demonstrate that the CBV-IPM combination exhibits antibiofilm activity, primarily attributed to the action of CBV.

### Effects of CBV and IPM on *Acinetobacter baumannii* cell membrane

**Figure 2:**
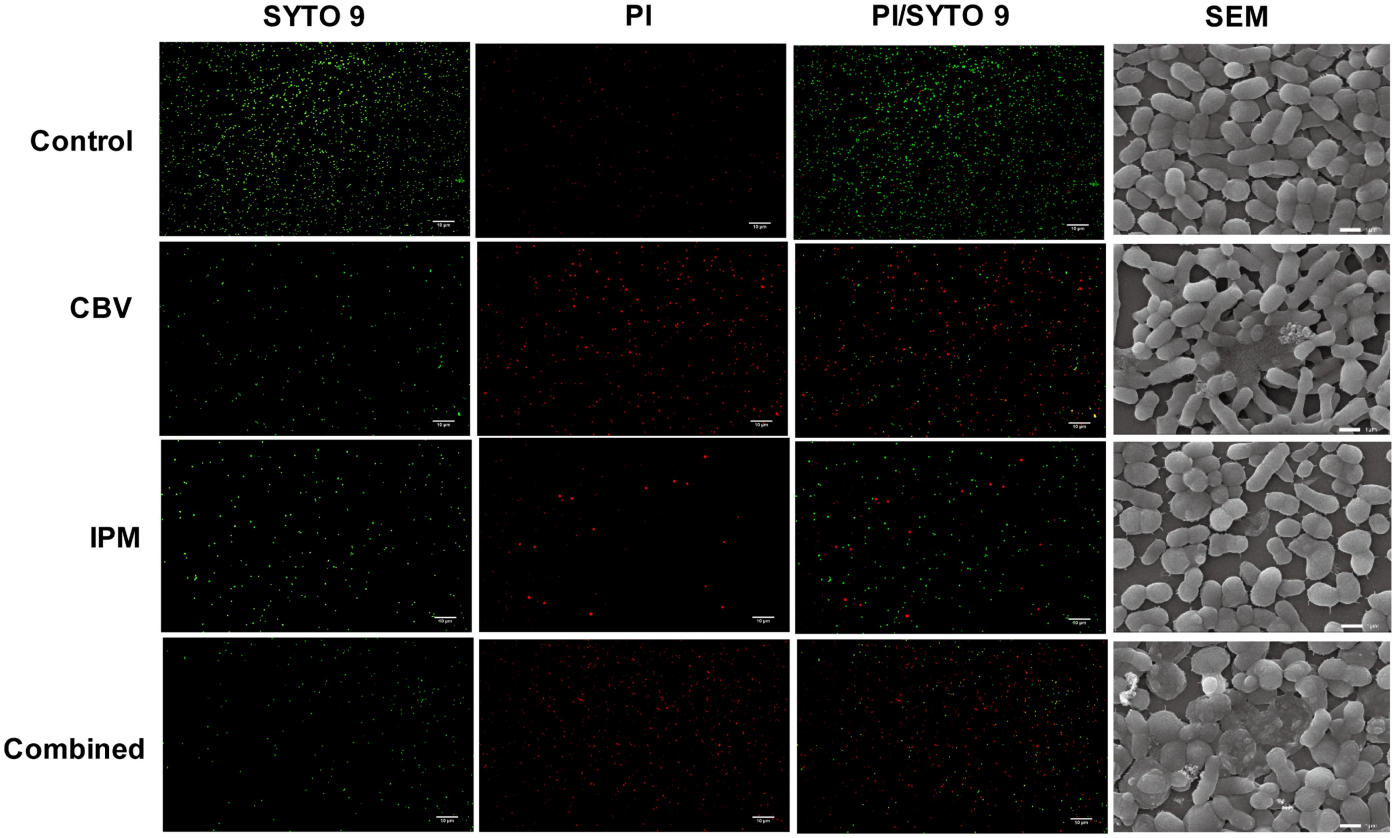
CBV, IPM, and their combination exert antibacterial effects by disrupting membrane integrity and permeability. Live/dead staining images of *A. baumannii* were obtained using a fluorescent microscope (200 × magnification). (SYTO 9) SYTO 9 stains all bacteria green, indicating high membrane permeability. (PI) PI only penetrates damaged membranes, staining bacteria with compromised membranes red. (PI/SYTO 9) Merged images of live/dead fluorescent staining. (SEM) Cell morphology of *A. baumannii* was observed using SEM at 10000x magnification.

SYTO 9/PI staining was used to evaluate cell membrane integrity (Wang et al., 2024). The results (Figure 2) showed that most bacteria in the control group exhibited strong green fluorescence (SYTO 9 staining). Following treatment with CBV or IPM, the intensity of red fluorescence (PI staining) increased significantly, particularly in the CBV and combination treatment groups. This indicates altered membrane permeability in *A. baumannii*. Compared to the three treatment groups, the fluorescence intensity ratio of PI/SYTO 9 varied, with the highest found in the combination group, followed by the CBV group. The IPM group had a relatively lower fluorescence intensity ratio of PI/SYTO 9. These findings demonstrate that both CBV and IPM disrupt *the* membrane integrity of *A. baumannii,* with an enhancement observed in combination therapy.

To further characterize the morphological and cell membrane alterations in *A. baumannii* induced by CBV and IPM, scanning electron microscopy (SEM) was performed (Figure 2). Untreated control cells exhibited a typical rod-shaped morphology with smooth, intact membranes and clearly defined cell boundaries. In contrast, CBV-treated cells displayed marked deformation, whereas IPM treatment resulted in irregular cell shapes. Notably, the combination of CBV and IPM led to complete morphological disintegration.

Membrane damage increases permeability and induces intracellular protein leakage (Cai et al., 2024). To further validate the effects of CBV and IPM on *A. baumannii* membrane integrity, intracellular protein content was quantified (Figure 1C). After 24 h of incubation, the control group cells showed progressive protein accumulation. In contrast, the treatment groups exhibited distinct patterns. At 8 h, all treatments showed significantly lower protein content than the controls (*p* < 0.05), although no inter-group differences were observed (*p* > 0.05). Between 16 and 24 h, both CBV- and IPM-treated cells demonstrated increasing protein levels, paralleling the controls but remaining significantly lower (p < 0.05). Notably, the CBV-IPM combination resulted in continuous protein depletion, with content significantly lower than that of all other groups by 24 h (*p* < 0.01). These results confirm that CBV and IPM disrupt membrane integrity, with combined treatment causing the most severe protein leakage.

The findings confirm that CBV and IPM independently compromise *A. baumannii* membrane integrity, while their combination exhibits additive effects, resulting in increased membrane disruption and enhanced bactericidal efficacy.

### Transcriptomic analysis of *Acinetobacter baumannii* by CBV and IPM treatment

#### Overview of RNA-seq data

**Figure 3:**
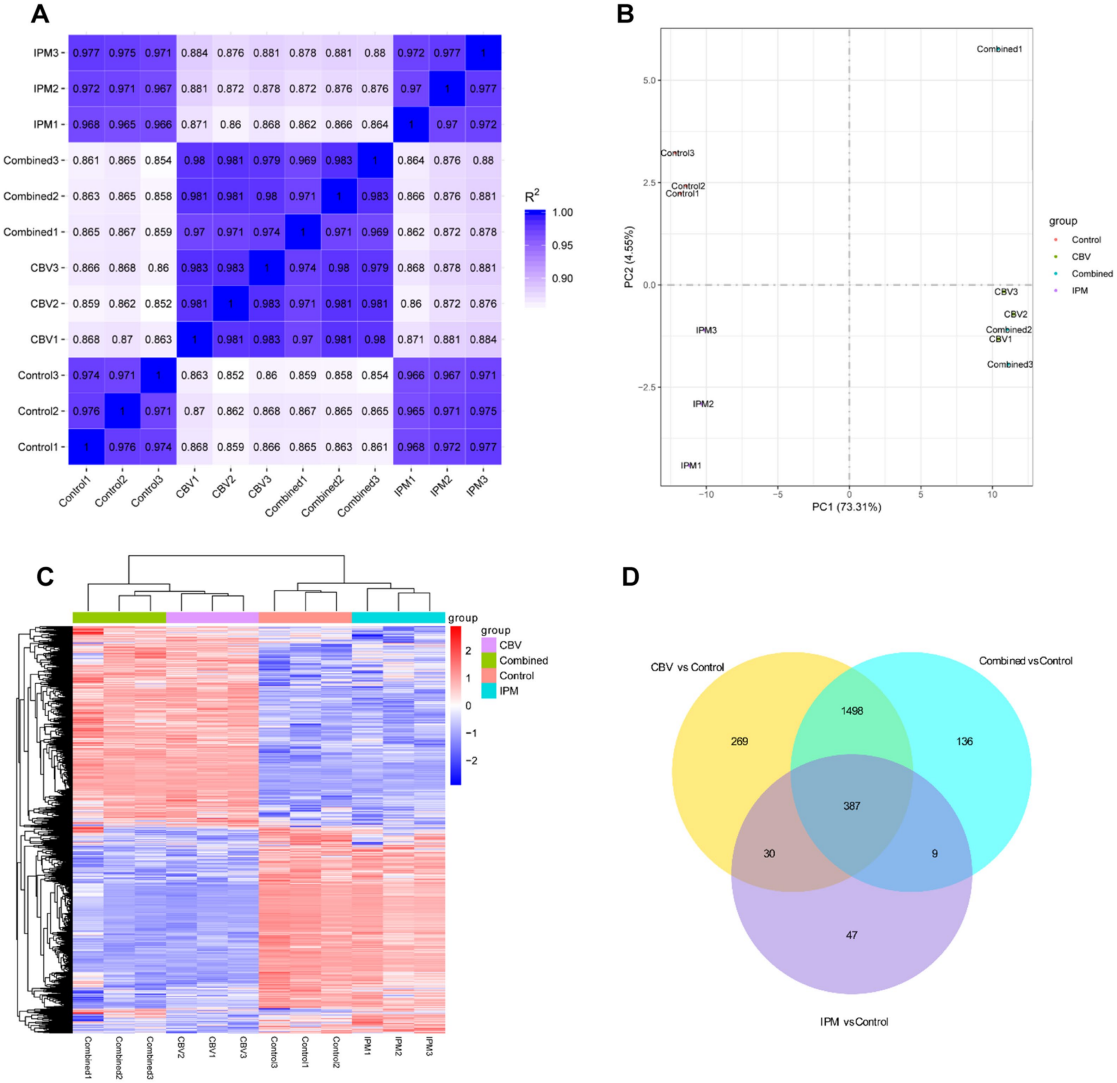
Transcriptomic profiling of *A. baumannii* treated with CBV, IPM, or their combination. Control1-3: control groups (no exposure to CBV and IPM); IPM1-3: IPM group (exposed to 4 μg/mL IPM); CBV1-3: CBV group (exposed to 0.125 μL/mL CBV); Combined1-3: combination group (exposed to 4 μg/mL IPM and 0.125 μL/mL CBV). **(A)** The correlation heatmap demonstrated high reproducibility among biological replicates. **(B)** Principal component analysis (PCA) of transcriptomic profiles resulted in a clear separation of the three groups. The CBV-treated group and the combination-treated group clustered together. **(C)** Hierarchical clustering based on differentially expressed genes (DEGs) resulted in three clusters, which is consistent with the PCA results. **(D)** The Venn diagram displayed shared and group-specific DEGs among the treatment groups and the control.

The raw RNA-seq data are summarized in Supplementary Table S1. In total, 23,195,004, 22,544,542, 22,894,702, and 23,388,444 clean reads were obtained for the control group and the three treatment groups (CBV group, IPM group, and combination group), respectively, with more than 91% of the reads mapping to the *A. baumannii* genome. Q30 percentages (>95%) indicated that the data for subsequent analyses were of high quality. Correlation analysis revealed better biological replicates (Figure 3A). Principal component analysis (PCA) showed a clear separation between the control group and the treatment groups. The CBV group and combination group formed the same cluster (Figure 3B). Hierarchical clustering analysis was used to evaluate the relationship between the differentially expressed gene (DEG) profiles of the four group samples (Figure 3C). It was found that the samples within the same groups clustered together. The four groups formed three clusters, which is consistent with the PCA results. The differentially expressed genes (DEGs) between the treatment groups and the control are shown in Figure 3D. A total of 387 shared DEGs were identified. The most unique DEGs were identified between the CBV and control groups, followed by the combination and control groups.

#### Key DEGs and their primary function between the CBV and control groups

**Figure 4:**
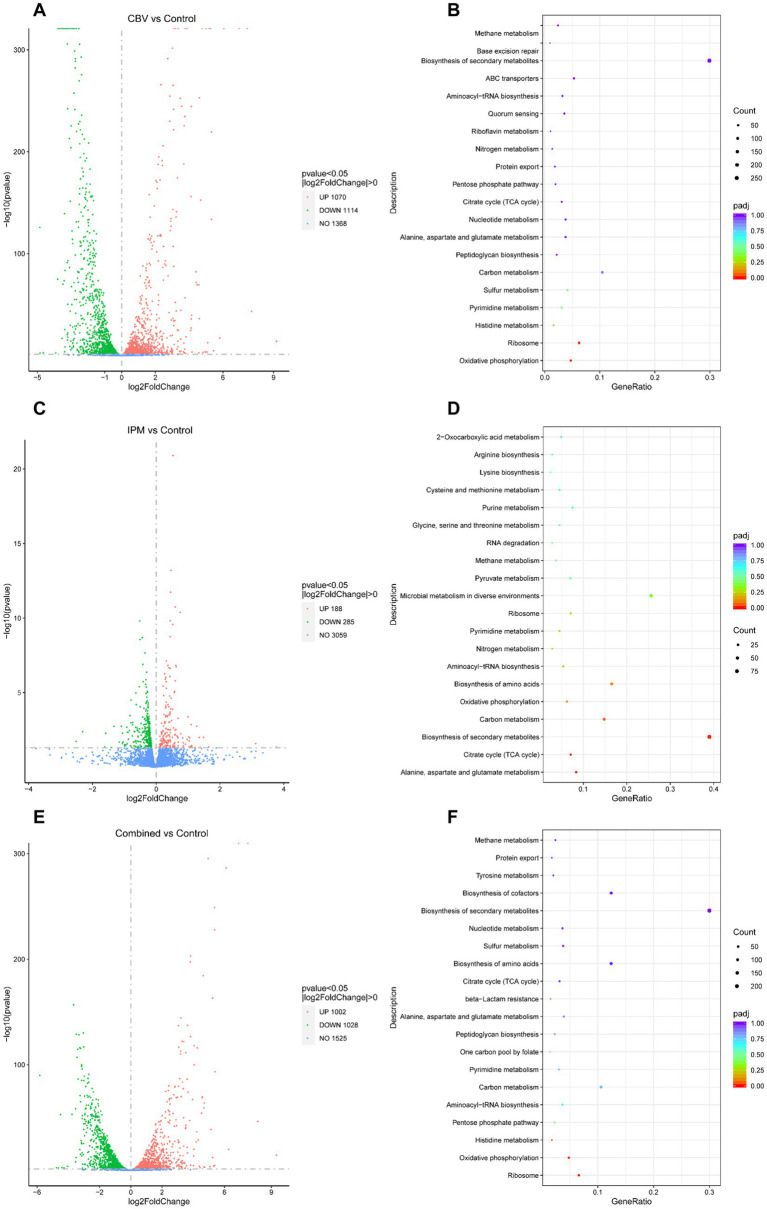
Analysis of DEGs. **(A)** Volcano plot of DEGs in *A. baumannii* following CBV (0.125 μL/mL) treatment. **(B)** KEGG pathways enriched by DEGs following CBV treatment in *A. baumannii*. **(C)** Volcano plot of DEGs in *A. baumannii* following IPM (4 μg/mL) treatment. **(D)** KEGG pathways enriched by DEGs following IPM treatment in *A. baumannii*. **(E)** Volcano plot of DEGs in *A. baumannii* treated with CBV (0.125 μL/mL) and IPM (4 μg/mL). **(F)** KEGG pathways enriched by DEGs following CBV and IPM treatments in *A. baumannii*.

Following CBV treatment, 2,184 genes of *A. baumannii* exhibited differential expression, including 1,070 upregulated and 1,114 downregulated DEGs (Figure 4A and Supplementary Table S2). The top 10 significantly upregulated and downregulated DEGs are listed in Table 2. It was found that the most upregulated genes were efflux pump genes, such as *abaF*, *adeF*, *adeH,* and ATP-binding cassette protein (*FQU82_RS00435*), followed by enzyme-related genes, including *antA*, *antB*, *FQU82_RS02000*, *FQU82_RS00340*, and *FQU82_RS18060*. Additionally, a flavodoxin gene was the most highly upregulated. The downregulated DEGs included two genes encoding outer membrane proteins (*FQU82_RS01960* and *FQU82_RS18570*), two genes encoding hypothetical proteins, and genes encoding phosphoglycerate kinase, ATP synthase, and others. The enriched DEGs of the top 20 KEGG pathways are shown in Figure 4B, where the terms with significant enrichment (padj < 0.05) *p*-values were oxidative phosphorylation (map00190) and ribosome (map03010). This indicates that CBV treatment influenced the energy metabolism and translation of *A. baumannii*.

**Table 2:** Top 10 upregulated and downregulated genes among differentially expressed genes (DEGs) identified in *A. baumannii* following CBV treatment.

Gene_id	Gene name	log2FC (CBV/Control)	padj	Annotation
The top 10 most significantly upregulated genes				
Novel00324	–	9.23	3.78E-14	Flavodoxin-like fold
FQU82_RS10355	*antA*	7.75	3.18E-43	Anthranilate 1,2-dioxygenase large subunit
FQU82_RS02000	*FQU82_RS02000*	7.49	0	Alpha/beta hydrolase
FQU82_RS18060	*FQU82_RS18060*	7.01	0	NAD(P)H-dependent oxidoreductase
FQU82_RS00340	*FQU82_RS00340*	6.11	0	MBL fold metallo-hydrolase
FQU82_RS10350	*antB*	5.86	2.84E-17	Anthranilate 1,2-dioxygenase small subunit
FQU82_RS08030	*abaF*	5.50	4.71E-05	Fosfomycin efflux MFS transporter AbaF
FQU82_RS13370	*adeF*	5.38	1.78E-218	Multidrug efflux RND transporter periplasmic adaptor subunit AdeF
FQU82_RS00435	*FQU82_RS00435*	5.38	4.91E-133	ATP-binding cassette domain-containing protein
FQU82_RS13380	*adeH*	5.32	0	Multidrug efflux RND transporter outer membrane subunit AdeH
The top 10 most significantly downregulated genes				
FQU82_RS01960	*FQU82_RS01960*	−4.80	4.15E-125	OmpW family outer membrane protein
Novel00044	-	−4.78	0.0078443	–
FQU82_RS09855	*FQU82_RS09855*	−4.60	0.0031779	Hypothetical protein
FQU82_RS09930	*FQU82_RS09930*	−3.85	0.0069029	Hypothetical protein
FQU82_RS18570	*FQU82_RS18570*	−3.74	1.49E-74	Outer membrane protein OmpK
FQU82_RS16155	*typA*	−3.73	0	Translational GTPase
FQU82_RS09405	*FQU82_RS09405*	−3.67	0	Phosphoglycerate kinase
FQU82_RS02905	*FQU82_RS02905*	−3.61	0	TonB-dependent siderophore receptor
FQU82_RS01325	*FQU82_RS01325*	−3.55	0	F0F1 ATP synthase subunit delta
FQU82_RS05015	*FQU82_RS05015*	−3.48	5.93E-17	YaeQ family protein

The most upregulated DEGs in A. baumannii after CBV treatment were efflux pump-related genes, while the downregulated DEGs primarily encoded proteins.

#### Key DEGs and their primary function between the IPM and control groups

Following IPM treatment, 473 genes of *A. baumannii* exhibited differential expression, including 188 upregulated and 285 downregulated DEGs (Figure 4C and Supplementary Table S3). The top 10 significantly up- and down-DEGs are listed in Table 3. It is shown that the most upregulated genes were those encoding a hypothetical protein (*FQU82_RS11495*, *FQU82_RS19140*, *FQU82_RS04010*), as well as two other genes, one related to the membrane (*carO*) and the other related to an efflux pump (*adeG*). The others encoded different proteins or enzymes. The downregulated genes were primarily involved in encoding TonB-dependent receptor genes (*FQU82_RS01405*, *FQU82_RS09850*, *FQU82_RS09935*, *FQU82_RS02905*) and enzyme-related genes (Novel00158, FQU82_RS15965, FQU82_RS15805, FQU82_RS03250).

**Table 3:** Top 10 upregulated and downregulated genes among DEGs identified in *A. baumannii* after IPM treatment.

Gene_id	Gene name	log2FC (IPM/Control)	padj	Gene description
The top 10 most significantly upregulated genes				
FQU82_RS11495	FQU82_RS11495	0.78	1.34E-08	Hypothetical protein
FQU82_RS19140	FQU82_RS19140	0.72	0.0101161	Hypothetical protein
FQU82_RS04010	FQU82_RS04010	0.65	1.80E-05	Hypothetical protein
FQU82_RS16605	FQU82_RS16605	0.64	1.53E-05	Rhodanese-like domain-containing protein
FQU82_RS01970	htpG	0.62	7.25E-09	Molecular chaperone HtpG
FQU82_RS18255	FQU82_RS18255	0.62	0.0001001	DUF1304 domain-containing protein
FQU82_RS03420	FQU82_RS03420	0.61	0.0003788	integration host factor subunit alpha
FQU82_RS13375	adeG	0.59	0.0004324	multidrug efflux RND transporter permease subunit AdeG
FQU82_RS04710	ssrA	0.56	2.01E-18	transfer-messenger RNA
FQU82_RS14810	carO	0.55	5.34E-08	ornithine uptake porin CarO type 1
The top 10 most significantly downregulated genes				
Novel00158	–	−0.79	0.0076216	PF00171: Aldehyde dehydrogenase family|PF01619: Proline dehydrogenase
FQU82_RS01400	FQU82_RS01400	−0.47	0.0025288	DUF2946 family protein
FQU82_RS01405	FQU82_RS01405	−0.48	4.01E-07	TonB-dependent copper receptor
FQU82_RS09850	FQU82_RS09850	−0.49	3.61E-08	TonB-dependent receptor
FQU82_RS15965	FQU82_RS15965	−0.50	0.00021	PepSY domain-containing protein
FQU82_RS09935	FQU82_RS09935	−0.61	0.0004548	TonB-dependent siderophore receptor
FQU82_RS09815	FQU82_RS09815	−0.77	0.0011536	IucA/IucC family protein
FQU82_RS02905	FQU82_RS02905	−0.41	3.32E-07	TonB-dependent siderophore receptor
FQU82_RS15805	FQU82_RS15805	−0.40	0.0087677	undecaprenyl-diphosphate phosphatase
FQU82_RS03250	FQU82_RS03250	−0.39	1.46E-05	NAD(P)(+) transhydrogenase (Re/Si-specific) subunit beta

The most upregulated and downregulated DEGs in *A. baumannii* after IPM treatment primarily encoded proteins.

The enriched DEGs of the top 20 KEGG pathways are shown in Figure 4D, in which the terms with significant enrichment (padj<0.05), such as alanine, aspartate, and glutamate metabolism (map00250), biosynthesis of secondary metabolites (map01110), and the citrate cycle (TCA cycle, map00020). Unlike CBV treatment, IPM primarily influences amino acid and carbohydrate metabolism, as well as the biosynthesis of secondary metabolites in *A. baumannii*.

#### Key DEGs and their primary function between the combination and control groups

When treated simultaneously with CBV and IPM, *A. baumannii* exhibited differential expression in 2,030 genes, with 1,002 being upregulated and 1,028 downregulated DEGs (Figure 4E and Supplementary Table S4). The top 10 significantly upregulated and downregulated DEGs are listed in Table 4. Among the upregulated genes, nine overlapped with those upregulated by CBV treatment, and one overlapped with IPM treatment. The majority of these were correlated with efflux pump membrane function. In contrast, the downregulated genes differed substantially from those observed in CBV and IPM treatment alone. Only four overlapped with those downregulated by CBV treatment. The remaining downregulated genes included ferric acinetobactin-related genes (*bauA* and *bauE*), an oxygen carrier gene (*FQU82_RS04610*), a bacterial virulence factor gene (*ata*), a tRNA^Thr^ gene (*FQU82_RS01880*), and a gene encoding a hypothetical protein (*FQU82_RS14275*).

**Table 4:** Top 10 upregulated and downregulated genes among DEGs identified in *A. baumannii* after combination treatment.

Gene_id	Gene name	log2FC (Combination/Control)	padj	Gene description
The top 10 most significantly upregulated genes				
Novel00324	–	9.39	2.46E-14	PF02525: Flavodoxin-like fold
FQU82_RS10355	*antA*	8.19	1.21E-45	Anthranilate 1,2-dioxygenase large subunit
FQU82_RS02000	*FQU82_RS02000*	7.55	0	Alpha/beta hydrolase
FQU82_RS18060	*FQU82_RS18060*	6.96	0	NAD(P)H-dependent oxidoreductase
FQU82_RS10350	*antB*	6.33	1.21E-19	Anthranilate 1,2-dioxygenase small subunit
FQU82_RS00340	*FQU82_RS00340*	6.17	2.39E-284	MBL fold metallo-hydrolase
FQU82_RS00435	*FQU82_RS00435*	5.45	3.98E-92	ATP-binding cassette domain-containing protein
FQU82_RS13380	*adeH*	5.43	8.10E-226	Multidrug efflux RND transporter outer membrane subunit AdeH
FQU82_RS08030	*abaF*	5.42	7.69E-05	Fosfomycin efflux MFS transporter AbaF
FQU82_RS13375	*adeG*	5.42	7.22E-247	Multidrug efflux RND transporter permease subunit AdeG
The top 10 most significantly downregulated genes				
FQU82_RS01960	*FQU82_RS01960*	−5.77	1.39E-88	OmpW family outer membrane protein
Novel00044	–	−4.70	0.0102536	–
FQU82_RS14125	*bauA*	−4.58	0.0145639	TonB-dependent ferric acinetobactin receptor
FQU82_RS04610	*FQU82_RS04610*	−4.44	4.41E-52	Bacteriohemerythrin
FQU82_RS14275	*FQU82_RS14275*	−4.35	0.0075838	Hypothetical protein
FQU82_RS14135	*bauE*	−3.98	0.0003637	Ferric acinetobactin ABC transporter ATP-binding protein
FQU82_RS18570	*FQU82_RS18570*	−3.71	2.72E-52	Outer membrane protein OmpK
FQU82_RS02905	*FQU82_RS02905*	−3.61	4.83E-155	TonB-dependent siderophore receptor
FQU82_RS05495	*ata*	−3.48	1.64E-57	Trimeric autotransporter adhesin
FQU82_RS01880	*FQU82_RS01880*	−3.47	3.52E-08	tRNA-Thr

The most upregulated DEGs in *A. baumannii* following combination treatment were consistent with the CBV treatment alone, while the downregulated DEGs showed significant differences.

Among the top 20 KEGG pathways enriched for DEGs (Figure 4F), three pathways were significantly enriched (padj < 0.05). The ribosome (map03010) and oxidative phosphorylation (map00190) pathways were also significantly enriched in the CBV treatment group. The other significantly enriched pathway – histidine metabolism (map00340) – was not significantly enriched in the CBV treatment alone. While the KEGG pathways significantly enriched in the IPM treatment were also present in the combination treatment group, they were not significantly enriched.

### Proteomic analysis of *Acinetobacter baumannii* by CBV and IPM treatment

#### Overview of proteomic data

**Figure 5:**
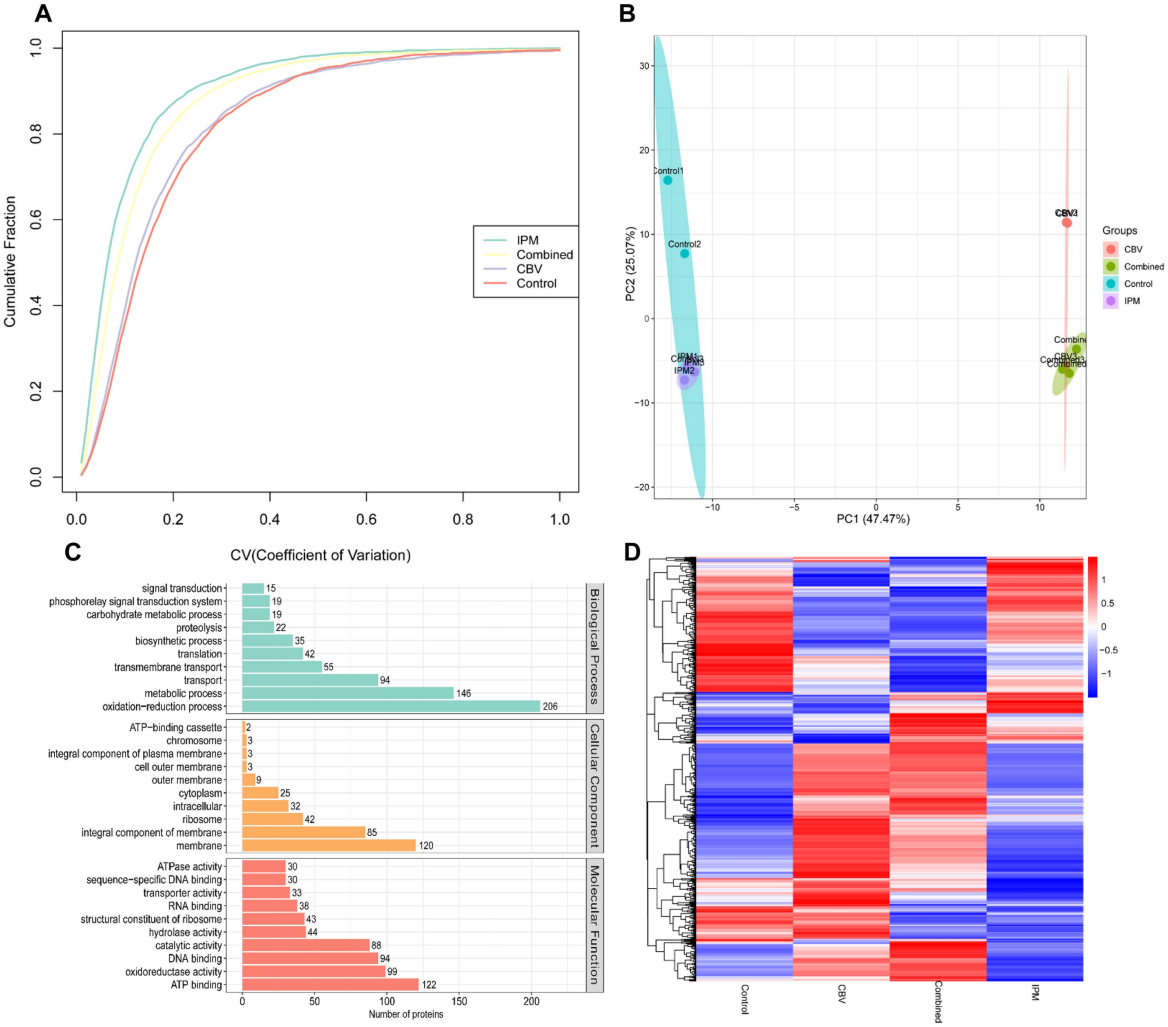
Proteomic profiling of *A. baumannii* treated with CBV (0.125 μL/mL), IPM (4 μg/mL), or their combination. **(A)** The coefficient of variation analysis demonstrated high consistency across biological replicates. **(B)** PCA of proteomic profiles showed clear separation among the three groups, with the control group and IPM-treated group clustering together. **(C)** GO enrichment analysis of the identified proteome following treatment with CBV, IPM, or their combination reveals the top 10 most enriched terms. **(D)** Hierarchical clustering based on differentially expressed proteins (DEPs) revealed a clear distinction among the groups.

The DIA technique was employed for the quantitative analysis of diverse proteins in *A. baumannii* treated with CBV, IPM, and both CBV and IPM. A total of 2,527 proteins were identified. The coefficient of variance and PCA analysis revealed good consistency among biological replicates (Figures 5A,B). GO enrichment analysis illustrated the association of these proteins with biological process (BP), cellular component (CC), and molecular function (MF) (Figure 5C). Differentially expressed proteins (DEPs) were identified based on the following criteria: fold change (FC) > 1.2 and *p* < 0.05 for upregulated proteins, FC < 0.83 and p < 0.05 for downregulated proteins. A total of 1,135 significant DEPs were identified across the four groups. Cluster analysis indicated a clear distinction among the groups based on their DEP profiles (Figure 5D).

#### DEP analysis between the CBV and control groups

**Figure 6:**
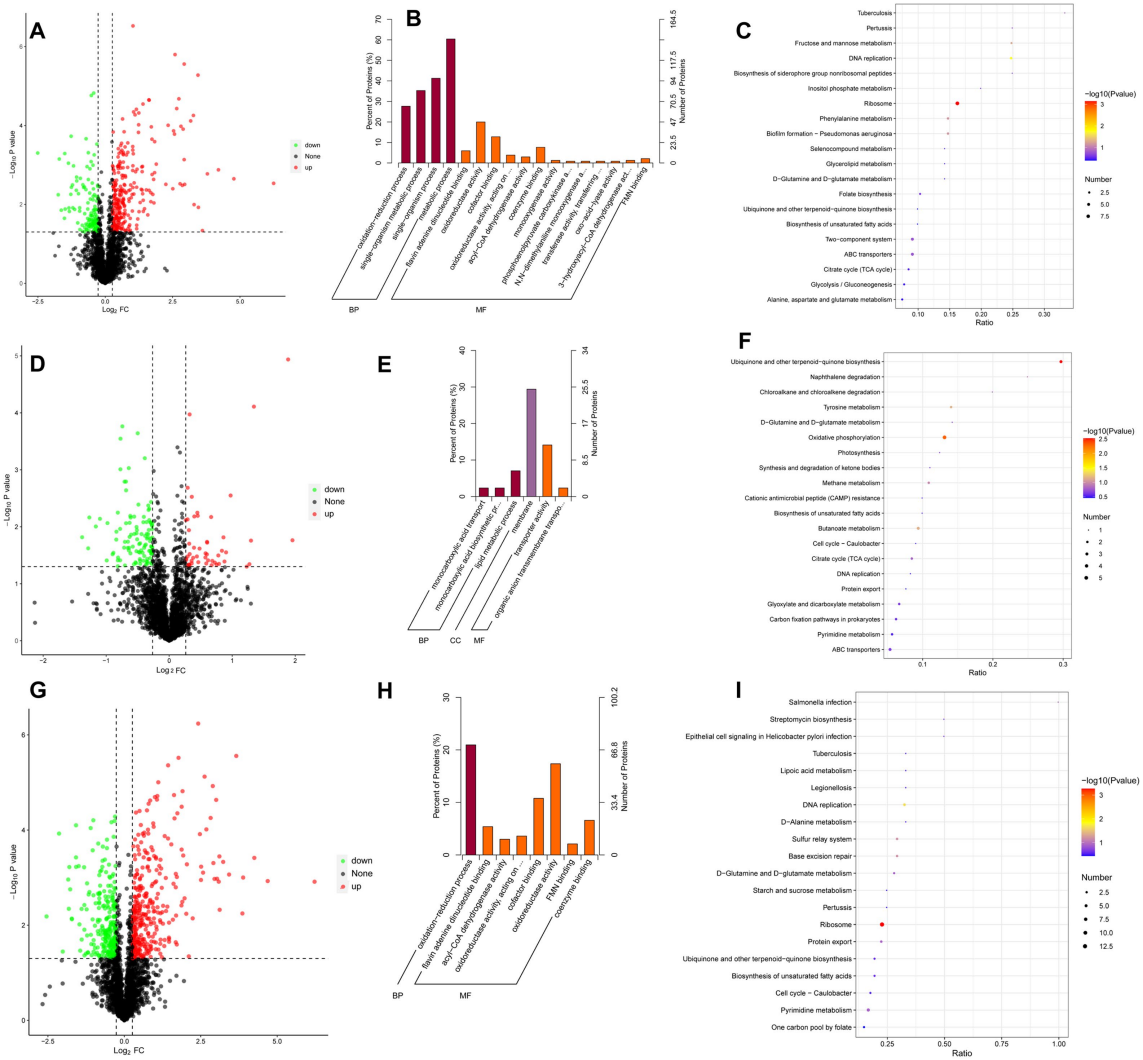
Analysis of DEPs. Dots highlighted in red (FC > 1.2) and green (FC < 0.83) indicate proteins whose expression was significantly altered (*p* < 0.05). **(A)** Volcano plot of DEPs in *A. baumannii* following CBV treatment. **(B)** GO enrichment analysis of DEPs following CBV treatment. **(C)** KEGG pathways enriched in downregulated DEPs after CBV treatment. **(D)** Volcano plot of DEPs in *A. baumannii* following IPM treatment. **(E)** GO enrichment analysis of DEPs following IPM treatment. **(F)** KEGG pathways enriched in downregulated DEPs after IPM treatment. **(G)** Volcano plot of DEPs in *A. baumannii* following combination treatment. **(H)** GO enrichment analysis of DEPs following combination treatment. **(I)** KEGG pathways enriched in downregulated DEPs after combination treatment.

A total of 411 proteins from *A. baumannii* exhibited different expressions, including 278 that were upregulated and 133 downregulated between the CBV treatment and control groups (Figure 6A). GO analysis demonstrated that the DEPs were significantly enriched in BP and MF categories (*p* < 0.05) (Figure 6B). The terms related to the oxidation–reduction process in BP, oxidoreductase activity, and monooxygenase activity in MF may relate to the DEPs that significantly influence the oxidative phosphorylation pathway. KEGG pathway analysis revealed that the downregulated DEPs were significantly enriched in the ribosome (map03010) (Figure 6C), which was consistent with the DEG pathway enriched in the CBV treatment versus control.

#### DEP analysis between the IPM and control groups

Following the IPM treatment, a total of 162 DEPs were identified compared to the control group, including 55 upregulated and 107 downregulated proteins (Figure 6D). GO analysis showed that these DEPs were significantly enriched in the BP, CC, and MF categories (*p* < 0.05) (Figure 6E). The terms in each category were greatly different from those observed in the CBV treatment group, suggesting distinct effects of CBV and IPM on *A. baumannii*. The monocarboxylic acid biosynthetic process and monocarboxylic acid transport may correlate significantly with DEGs and influence the citrate cycle (TCA cycle). KEGG pathway analysis revealed that the downregulated DEPs were significantly enriched in ubiquinone and other terpenoid-quinone biosynthesis (map00130) and oxidative phosphorylation (map00190) (Figure 6F). Additionally, the citrate cycle (TCA cycle) was also enriched, which was consistent with the DEG pathway identified in the comparison between IPM treatment and the control.

#### DEP analysis between the combination and control groups

When treated with a combination of CBV and IPM, a total of 594 DEPs were screened, compared to the control group, including 339 upregulated and 255 downregulated (Figure 6G). Similar to CBV treatment, GO analysis was also significantly enriched in BP and MF categories (*p* < 0.05) (Figure 6H). In comparison, the terms were less than CBV treatment. The terms of BP were only significantly enriched in the oxidation–reduction process. Meanwhile, oxidoreductase activity and monooxygenase activity in MF were also enriched. This finding was consistent with CBV treatment and may correlate with the DEGs that significantly influence the oxidative phosphorylation pathway between the combination and control groups. KEGG pathway analysis of the downregulated DEPs was the same as that of the CBV treatment, which was significantly enriched in the ribosome (map03010) (Figure 6I). This finding is consistent with the enriched pathway of DEGs between combination treatment and the control.

### Correlation analysis between transcriptomic and proteomic data

The analysis of the significantly enriched KEGG pathways among the DEGs revealed that the primary enriched KEGG pathways for both upregulated and downregulated DEGs were consistent. Additionally, the significantly enriched KEGG pathways of downregulated DEPs were consistent with those of DEGs. Consequently, the correlation analysis between transcriptomic and proteomic data focused on the downregulated differentially expressed genes (DEGs) and differentially expressed proteins (DEPs).

#### Commonly shared enriched KEGG pathways of DEGs and DEPs between the CBV and control groups

Based on the significantly downregulated DEGs and DEPs in the CBV and control groups, a total of 19 KEGG pathways were commonly shared (Supplementary Table S5). Among them, only the ribosome pathway (map03010) was significantly enriched in both DEGs and DEPs (*p* < 0.001). Compared to the control, *A. baumannii* treated with 0.125 μL/mL CBV exhibited 54 proteins, including eight significant DEPs (Rps8, Rps13, Rps18, Rpl4, Rpl14, Rpl22, Rpl21, Rpl34, and Rpl36) and 55 genes (e.g., RplA-F, RpsA-Q, RpmA-D, etc.) that were downregulated and involved in ribosomes.

#### Commonly shared enriched KEGG pathways of DEGs and DEPs between the IPM and control groups

Based on the significantly downregulated DEGs and DEPs in the IPM and control groups, a total of 31 KEGG pathways were commonly shared (Supplementary Table S5). Among these, only the oxidative phosphorylation pathway (map00190) was significantly enriched in both DEGs and DEPs (*p* < 0.001). Compared to the control, *A. baumannii* treated with 4 μg/mL IPM exhibited 37 proteins, including five significant DEPs (F0F1 ATP synthase subunit C, cytochrome d ubiquinol oxidase subunit II, cytochrome o ubiquinol oxidase subunit III, succinate dehydrogenase, and cytochrome b556 subunit) and 14 genes (e.g., AtpA, NuoC, SdhA, and so on) that were found downregulated and involved in oxidative phosphorylation.

#### Commonly shared enriched KEGG pathways of DEGs and DEPs between the combination and control groups

Based on the significantly downregulated DEGs and DEPs in the combination and control groups, a total of 26 KEGG pathways were commonly shared (Supplementary Table S5). The significantly enriched KEGG pathways (ribosome pathway) in both DEGs and DEPs were the same as those observed with CBV treatment. Compared to the control, *A. baumannii* treated with 0.125 μL/mL CBV and 4 μg/mL IPM showed 54 proteins (including 13 significant DEPs), and 53 genes involved in ribosome function were found to be downregulated. Compared with CBV treatment, in the ribosome pathway, six significant DEPs (Rps8, Rps18, Rpl4, Rpl14, Rpl21, and Rpl36) were the same, while six significant DEPs (Rps1, Rps19, Rpl1, Rpl6, Rpl9, and RpL7/L12) were only found in the combination treatment. This indicates that combination treatment had a greater impact on the proteins of *A. baumannii* than the CBV treatment in this pathway. For the oxidative phosphorylation pathway, only DEGs were significantly enriched when treated with 0.125 μL/mL CBV and 4 μg/mL IPM (*p* < 0.01). Analysis of the DEPs found that 37 proteins (including five significant DEPs) involved in this pathway were consistent with IPM treatment. However, the total number of significant DEPs increased from 30 to 91, which may explain why DEP enrichment was not significant in the oxidative phosphorylation pathway. Compared to IPM treatment, more significant DEGs (a total of 35) were found to be downregulated.

The above study illustrated that both CBV and IPM contributed to antibacterial activity. CBV primarily targeted the ribosomal pathway, while IPM mainly influenced oxidative phosphorylation. When CBV was combined with IPM, the antibacterial effect was enhanced due to a significant impairment of ribosome assembly, which hindered protein synthesis. Additionally, oxidative phosphorylation was also severely affected, which influenced the production of ATP.

## Discussion

### Combined antibacterial activity of CBV and IPM

The emergence of bacterial antimicrobial resistance reduces the effectiveness of antibiotics and has become one of the major public health threats (Xuan et al., 2023). To combat MDR, the search for new antibiotics or antibacterial agents from natural products has attracted considerable attention (Zeng et al., 2024). Volatile oils, the hydrophobic, concentrated liquids extracted from aromatic plants, contain functional bioactive components and exhibit strong antioxidant and antimicrobial properties (Ebrahimzadeh et al., 2023). In this study, CBV and its combined IPM against *A. baumannii* were investigated. The antibacterial activity assay revealed that CBV exhibited significant inhibitory effects against IPM-resistant *A. baumannii*. The MIC was 0.25 μL/mL, equivalent to 0.25 μg/mL, which is far lower than the MIC of IPM (256 μg/mL). To understand the material basis of CBV’s antibacterial properties, the chemical composition was analyzed using GC–MS. The results illustrated that more than 60% of the compound was cinnamaldehyde. The others were all less than 10%. The antibacterial activity of cinnamaldehyde against *Escherichia coli*, *Salmonella* S., and *Streptococcus mutans* has been reported (Guo et al., 2024). Additionally, the antimicrobial activity of *α*-Copaene, 2-Methoxycinnamaldehyde, and calamenene derivative (Limna Mol et al., 2020) was also revealed. α-Copaene exhibits antibacterial activity against *Staphylococcus aureus*, *Escherichia coli*, *Bacillus cereus*, and *Shigella bogdii* by damaging bacterial membranes and suppressing biofilm formation (Chen et al., 2024). 2-Methoxycinnamaldehyde could inhibit the growth and pathogenic potential of methicillin-resistant *Staphylococcus epidermidis* and *Salmonella enteritidis* (Qian et al., 2022; Wu et al., 2025). Thus, CBV inhibition of *A. baumannii* was the comprehensive function of these compounds, which plays a major role and requires further investigation. When CBV was combined with IPM, an additive function was found. CBV significantly decreased the action concentration of IPM and improved the antibacterial of IPM against *A. baumannii*. Compared to IPM-relebactam and meropenem-vaborbactam, the two novel carbapenem-*β*-lactamase inhibitor combinations that did not improve the activity of IPM or meropenem against *A. baumannii* (Zhanel et al., 2018), the combination of IPM and CBV may be a new option for inhibiting *A. baumannii*.

### The antibacterial mechanisms of CBV and IPM

#### Influence biofilm synthesis and cell membrane integrity

The antimicrobial-resistant mechanisms included the regulation of membrane permeability, enzymatic modification and neutralization of antibiotics, and alteration of antibiotic sites (Kyriakidis et al., 2021). More importantly, biofilm formation was one of the key factors determining the virulence of *A. baumannii* (Lucidi et al., 2024). Thus, for the mechanisms of CBV combined with IPM against *A. baumannii*, we focused on the effects on biofilms using different methods, including crystal violet staining, SYTO 9/PI mixture staining, and SEM analysis. Crystal violet staining has been proven to be a useful method for quantifying biofilms (Castro et al., 2022). The results showed that IPM, CBV, and the combination group inhibited biofilm synthesis.

Regarding membrane permeability, a higher proportion of red fluorescence in SYTO 9/PI staining indicates increased membrane permeability (Wang et al., 2024). The results demonstrated that the CBV group and the combination treatment group showed more red fluorescence. These findings suggest that both CBV and IPM can inhibit biofilm synthesis and increase cell membrane permeability. Furthermore, their combination enhanced these effects.

It has been reported that increased membrane permeability leads to cytoplasmic leakage (Liang et al., 2025). To further confirm that CBV and IPM disrupt the cell membrane, intracellular protein levels were measured. The results revealed that treatment with either CBV or IPM alone caused intracellular protein leakage and showed significantly lower content, mirroring the trend observed in the control group. When treated with the combination of CBV and IPM, intracellular protein levels were consistently lower.

#### Key processes in *Acinetobacter baumannii* involved in the response to the CBV and IPM treatment

The combined use of transcriptomic and proteomic analyses has been used to provide novel insights into antibacterial mechanisms, such as understanding biofilm formation (Yao et al., 2022), DNA damage, metabolic disorders (J. Wang et al., 2023; Huiting Zhang et al., 2023), and so on. To further confirm that CBV and IPM affect biofilm formation, cell membrane integrity, and protein synthesis, transcriptomic and proteomic analyses were performed. Transcriptomic analysis revealed that, compared to the control group, treatment with CBV and its combination could significantly regulate the resistance nodulation division (RND) family efflux pumps, the one major facilitator superfamily (*abaF*), and one ATP-binding cassette (*FQU82_RS00435*) (Table 2 and Table 4). Previous studies have reported that at least five efflux pump superfamily transporters are involved in the antimicrobial resistance of *A. baumannii* (María Pérez-Varela et al., 2019). In this study, three efflux pump superfamilies of transporters were identified among the top 10 significantly upregulated differentially expressed genes (DEGs). RND efflux pumps can expel a wide spectrum of antibiotics and toxic compounds and have been shown to be associated with MDR in *A. baumannii* (Huang et al., 2022). To reveal the function of RND efflux pumps in CBV and combination treatment against *A. baumannii*, the transporters in the DEGs belonging to RND efflux pumps were summarized in Table S6. It was shown that the *adeAB*, *adeIJK,* and *adeM* were significantly downregulated. While the *adeFGH*, *adeL*, *adeN,* and *abaF* were significantly upregulated. These RND efflux pump transporters exhibited different trends when treated with CBV and in combination. Efflux pumps are the primary elements in the evolution of organisms. It means that antibiotic resistance was not the primary role of bacterial efflux pumps. They naturally contribute to the processes of microbial physiology, such as physiology, pathogenicity, and metabolism (Alcalde-Rico et al., 2016; Zack et al., 2024). In this study, CBV and combination treatment led to the expression of various efflux systems. It may induce various physiological changes within the bacterial cells, which in turn influence the survival of *A. baumannii*. The mechanisms underlying the participation of these efflux pumps in CBV and combination treatment against *A. baumannii* remain unclear and require further study. For the downregulated genes, the three treatment groups showed differences. CBV treatment significantly affects outer membrane proteins and ATP, indicating that CBV acts against *A. baumannii* by inhibiting the cell membrane and energy supply. IPM treatment greatly influenced the TonB-dependent receptor and the enzyme. While combination treatment not only decreased the expression of outer membrane proteins and TonB-dependent receptors but also inhibited the expression of bacterial virulence factors (*ata*). Overall, the DEGs illustrated that the antibacterial activity of CBV and combination treatment was correlated with the cell membrane, ATP, and efflux pumps. It was a comprehensive function, especially for efflux pumps. Functional annotation analysis confirmed that the differentially expressed genes (DEGs) were significantly enriched in oxidative phosphorylation (map00190), ribosome (map03010), citrate cycle (TCA cycle, map00020), and various metabolic pathways. Bacterial oxidative phosphorylation has been identified as a potential target for antibacterial drugs (Guo et al., 2025). The X33 antimicrobial oligopeptide against *A. baumannii* was found to target oxidative phosphorylation, the ribosome, the citrate cycle, and other pathways (Lu et al., 2024). Additionally, the citrate cycle and oxidative phosphorylation have been reported as the primary pathways modulated by the ferric uptake regulator, which influences the virulence of *A. baumannii* (Peng et al., 2025). These findings are consistent with our results, demonstrating that oxidative phosphorylation, ribosomes, and the citrate cycle play critical roles in combating *A. baumannii*.

Correlation analysis of transcriptomic and proteomic data revealed that the number of commonly shared enriched KEGG pathways, as well as the number of differentially expressed genes (DEGs) and differentially expressed proteins (DEPs), was the largest for IPM treatment, followed by the combination treatment. The lowest number was observed in the CBV treatment group (Supplementary Table S5). A total of 8 shared KEGG pathways existed across the three treatment groups. Four shared KEGG pathways were exclusive to the CBV and combination groups, and seven shared KEGG pathways were exclusive to the IPM and combination groups. These results illustrated that the antibacterial processes of IPM and CBV differed, and the combined antibacterial effect was influenced by both CBV and IPM. Further analysis of the key processes in *A. baumannii* involved in the response to CBV and IPM revealed that the significantly enriched KEGG pathways in both DEGs and DEPs were limited to the ribosome and oxidative phosphorylation. For ribosomal pathways, it was found to be significantly enriched in CBV and combination treatment. Ribosomes are crucial for bacterial survival and growth, as they influence the protein synthesis process. Approximately 60% of approved antibacterial agents combat bacteria by targeting ribosomes (Zhang et al., 2021). This study found that CBV downregulated the genes and proteins in the ribosomes of *A. baumannii,* exhibiting antibacterial activity. Targeting ribosomes was one of the main reasons for *A. baumannii’s* response to CBV and combination treatment.

Regarding the oxidative phosphorylation pathways, it was significantly enriched in the IPM treatment. Although the pathway was not significantly enriched for DEPs in the combination treatment group, the DEPs involved were completely consistent with those in the IPM treatment. Oxidative phosphorylation controls ATP synthesis and energy production, making it a potential target for antifungal therapy (Wang et al., 2021). This study found that both IPM and the combination treatment significantly affected the process of oxidative phosphorylation. Therefore, oxidative phosphorylation is recognized as another key process contributing to the combined antibacterial mechanism against *A. baumannii*.

## Conclusion

This study provides the first experimental evidence that CBV effectively combines with IPM to overcome drug resistance in *A. baumannii*. The results reveal a dual antimicrobial mechanism: the CBV/IPM combination not only compromises bacterial membrane integrity but also disrupts ribosome function and oxidative phosphorylation pathways. This unique multi-target action distinguishes CBV from conventional antibiotic potentiators, accounting for its remarkable additive effect with IPM. Importantly, CBV’s natural origin and potent activity against multidrug-resistant strains highlight it as a promising next-generation antibiotic enhancer for combating recalcitrant *A. baumannii* infections. While the current study establishes a mechanistic foundation for CBV’s additive antimicrobial effects, further research is needed to support clinical translation. Specifically, formulation optimization studies are required to enhance the therapeutic viability of CBV/IPM, and comprehensive *in vivo* investigations are essential to validate the combined antibacterial efficacy and safety profile of CBV/IPM before clinical application.

## Data Availability

The data for transcriptomic analysis has been released, and are available in the NCBI repository under accession number PRJNA1243479.
