# Supplementary material for: Exploring the additive antibacterial potential of Cinnamomum cassia volatile oil and imipenem against Acinetobacter baumannii: a multi-omics investigation
Source: Front Microbiol. 2025 Jul 2;16:1578322. doi: 10.3389/fmicb.2025.1578322 (PMC12263672; doi:10.3389/fmicb.2025.1578322)
Supplement: Supplementary file 1 [file Table_1.DOCX]

Supplementary Material

Composition Changes in *Lycium ruthenicum* Fruit

Exploring the Synergistic Antibacterial Potential of *Cinnamomum cassia* Volatile Oil and Imipenem against *Acinetobacter baumannii*: A Multi-Omics Investigation

Youyuan Lu ^1,3^, Wanlin Xu ^1^, Jiahui Xue ^1^, Mingxia Xie ^2^, Haotian Liu ^1^, Ruilin Wang ^1^, Gang Li ^1, *^, Hanqing Wang ^1,3*^

^1^College of Pharmacy, General Hospital of Ningxia Medical University, Ningxia Medical University, Yinchuan, China

^2^College of Traditional Chinese Medicine, Hunan University of Chinese Medicine, Changsha, China

^3^Key Laboratory of Protection, Development and Utilization of Medicinal Resources in Liupanshan Area, Ministry of Education, Ningxia Engineering and Technology Research Center for Modernization of Regional Characteristic Traditional Chinese Medicine, Ningxia Medical University, Yinchuan, China

*** Correspondence:**Hanqing Wang
[wwwhhq@163.com](mailto:wwwhhq@163.com)

Gang Li
[zyxyjl@163.com](mailto:zyxyjl@163.com)

**Table S1** **Statistics for RNA-seq data**

| Categories | Control group | | | CBV group | | | IPM group | | | Combined group | | |
| --- | --- | --- | --- | --- | --- | --- | --- | --- | --- | --- | --- | --- |
|  | Control 1 | Control 2 | Control 3 | CBV 1 | CBV 2 | CBV 3 | IPM 1 | IPM 2 | IPM 3 | Combined 1 | Combined 2 | Combined 3 |
| Raw reads number | 8,841,512 | 7,269,456 | 7,642,076 | 7,777,440 | 7,792,660 | 7,581,018 | 7,979,522 | 7,599,674 | 8,015,946 | 9,180,300 | 7,535,482 | 7,580,744 |
| Clean reads number | 8,647,264 | 7,118,990 | 7,428,750 | 7,544,574 | 7,545,692 | 7,454,276 | 7,723,124 | 7,368,508 | 7,803,070 | 8,982,492 | 7,170,818 | 7,235,134 |
| Q20 (%) | 99.1 | 99.07 | 99.14 | 99.17 | 99.11 | 99.11 | 98.6 | 99.15 | 99.18 | 98.6 | 99.15 | 99.18 |
| Q30 (%) | 97.17 | 97.09 | 97.28 | 97.39 | 97.21 | 97.22 | 95.81 | 97.35 | 97.43 | 95.81 | 97.35 | 97.43 |
| Total mapped reads | 8,287,835 | 6,804,560 | 7,118,566 | 6,909,343 | 6,897,075 | 6,838,746 | 7,387,045 | 7,015,899 | 7,444,299 | 8,228,022 | 6,575,735 | 6,630,291 |
| Total mapped (%) | 95.84 | 95.58 | 95.82 | 91.58 | 91.4 | 91.74 | 95.65 | 95.21 | 95.4 | 91.6 | 91.7 | 91.64 |

**Table S2** **The DEGs identified in *A. baumannii* after CBV treatment**

| **Gene_id** | **Gene name** | **Gene description** | **log2FC (CBV/Control)** | **pvalue** | **padj** |
| --- | --- | --- | --- | --- | --- |
| FQU82_RS05490 | lon | endopeptidase La | 3.141060125 | 0 | 0 |
| FQU82_RS17155 | rplB | 50S ribosomal protein L2 | -3.106660834 | 0 | 0 |
| FQU82_RS17045 | rpoA | DNA-directed RNA polymerase subunit alpha | -2.7979159 | 0 | 0 |
| FQU82_RS17140 | rpsC | 30S ribosomal protein S3 | -3.105456245 | 0 | 0 |
| FQU82_RS17050 | rpsD | 30S ribosomal protein S4 | -2.855318069 | 0 | 0 |
| FQU82_RS01915 | rplJ | 50S ribosomal protein L10 | -3.166289066 | 0 | 0 |
| FQU82_RS01910 | rplA | 50S ribosomal protein L1 | -3.144576814 | 0 | 0 |
| FQU82_RS17095 | rplF | 50S ribosomal protein L6 | -2.809140336 | 0 | 0 |
| FQU82_RS17170 | rplC | 50S ribosomal protein L3 | -3.213824168 | 0 | 0 |
| FQU82_RS01920 | rplL | 50S ribosomal protein L7/L12 | -3.053662118 | 0 | 0 |
| FQU82_RS13840 | rpsB | 30S ribosomal protein S2 | -2.495795529 | 0 | 0 |
| FQU82_RS01340 | atpD | F0F1 ATP synthase subunit beta | -2.824228855 | 0 | 0 |
| FQU82_RS17110 | rplE | 50S ribosomal protein L5 | -2.939833378 | 0 | 0 |
| FQU82_RS17085 | rpsE | 30S ribosomal protein S5 | -2.7116193 | 0 | 0 |
| FQU82_RS01330 | atpA | F0F1 ATP synthase subunit alpha | -3.274908055 | 0 | 0 |
| FQU82_RS17165 | rplD | 50S ribosomal protein L4 | -3.318190435 | 0 | 0 |
| FQU82_RS01905 | rplK | 50S ribosomal protein L11 | -3.135970091 | 0 | 0 |
| FQU82_RS10500 | FQU82_RS10500 | fimbrial protein | 3.92865642 | 0 | 0 |
| FQU82_RS17075 | rplO | 50S ribosomal protein L15 | -2.465570067 | 0 | 0 |
| FQU82_RS17060 | rpsM | 30S ribosomal protein S13 | -2.755270486 | 0 | 0 |
| FQU82_RS17055 | rpsK | 30S ribosomal protein S11 | -2.894073871 | 0 | 0 |
| FQU82_RS17150 | rpsS | 30S ribosomal protein S19 | -3.162185151 | 0 | 0 |
| FQU82_RS17100 | rpsH | 30S ribosomal protein S8 | -2.949030799 | 0 | 0 |
| FQU82_RS17145 | rplV | 50S ribosomal protein L22 | -3.041825351 | 0 | 0 |
| FQU82_RS17175 | rpsJ | 30S ribosomal protein S10 | -3.239642945 | 0 | 0 |
| FQU82_RS17135 | rplP | 50S ribosomal protein L16 | -2.875826574 | 0 | 0 |
| FQU82_RS18060 | FQU82_RS18060 | NAD(P)H-dependent oxidoreductase | 7.012228723 | 0 | 0 |
| FQU82_RS17090 | rplR | 50S ribosomal protein L18 | -2.837318548 | 0 | 0 |
| FQU82_RS17160 | rplW | 50S ribosomal protein L23 | -3.363009859 | 0 | 0 |
| FQU82_RS01335 | atpG | F0F1 ATP synthase subunit gamma | -3.13561675 | 0 | 0 |
| FQU82_RS17040 | rplQ | 50S ribosomal protein L17 | -2.635490313 | 0 | 0 |
| FQU82_RS17130 | rpmC | 50S ribosomal protein L29 | -2.846675179 | 0 | 0 |
| FQU82_RS17105 | rpsN | 30S ribosomal protein S14 | -2.941179476 | 0 | 0 |
| FQU82_RS18365 | FQU82_RS18365 | alkene reductase | 3.802228039 | 0 | 0 |
| FQU82_RS16155 | typA | translational GTPase TypA | -3.728305432 | 0 | 0 |
| FQU82_RS13375 | adeG | multidrug efflux RND transporter permease subunit AdeG | 5.233417182 | 0 | 0 |
| FQU82_RS13835 | tsf | translation elongation factor Ts | -3.041229586 | 0 | 0 |
| FQU82_RS00340 | FQU82_RS00340 | MBL fold metallo-hydrolase | 6.106133638 | 0 | 0 |
| FQU82_RS02000 | FQU82_RS02000 | alpha/beta hydrolase | 7.486594227 | 0 | 0 |
| FQU82_RS08415 | FQU82_RS08415 | indolepyruvate ferredoxin oxidoreductase family protein | 3.401595537 | 0 | 0 |
| FQU82_RS02655 | FQU82_RS02655 | zinc-binding alcohol dehydrogenase family protein | 5.052624576 | 0 | 0 |
| FQU82_RS01325 | FQU82_RS01325 | F0F1 ATP synthase subunit delta | -3.548863554 | 0 | 0 |
| FQU82_RS17580 | rpsP | 30S ribosomal protein S16 | -2.8730288 | 0 | 0 |
| FQU82_RS01320 | FQU82_RS01320 | F0F1 ATP synthase subunit B | -3.429522585 | 0 | 0 |
| FQU82_RS04310 | FQU82_RS04310 | ribose-phosphate pyrophosphokinase | -3.384711236 | 0 | 0 |
| FQU82_RS03015 | ndk | nucleoside-diphosphate kinase | -3.441855084 | 0 | 0 |
| FQU82_RS14475 | FQU82_RS14475 | LysR family transcriptional regulator | 3.244695791 | 0 | 0 |
| FQU82_RS13380 | adeH | multidrug efflux RND transporter outer membrane subunit AdeH | 5.318871195 | 0 | 0 |
| FQU82_RS00445 | ssuD | FMNH2-dependent alkanesulfonate monooxygenase | 4.825049442 | 0 | 0 |
| FQU82_RS02905 | FQU82_RS02905 | TonB-dependent siderophore receptor | -3.612714691 | 0 | 0 |
| FQU82_RS01315 | atpE | F0F1 ATP synthase subunit C | -3.477231871 | 0 | 0 |
| FQU82_RS09405 | FQU82_RS09405 | phosphoglycerate kinase | -3.668727326 | 0 | 0 |
| FQU82_RS12700 | purH | bifunctional phosphoribosylaminoimidazolecarboxamide formyltransferase/IMP cyclohydrolase | -3.163707212 | 1.58E-306 | 1.06E-304 |
| FQU82_RS17070 | secY | preprotein translocase subunit SecY | -2.445386714 | 3.67E-306 | 2.42E-304 |
| FQU82_RS06935 | clpB | ATP-dependent chaperone ClpB | 3.059911545 | 2.88E-302 | 1.86E-300 |
| FQU82_RS17080 | rpmD | 50S ribosomal protein L30 | -2.736337467 | 1.55E-299 | 9.85E-298 |
| FQU82_RS04495 | fusA | elongation factor G | -2.347251673 | 1.32E-293 | 8.20E-292 |
| FQU82_RS18840 | fahA | fumarylacetoacetase | 2.781806322 | 3.60E-292 | 2.20E-290 |
| FQU82_RS01345 | FQU82_RS01345 | F0F1 ATP synthase subunit epsilon | -2.68888492 | 5.61E-292 | 3.38E-290 |
| FQU82_RS17125 | rpsQ | 30S ribosomal protein S17 | -2.686552239 | 1.27E-289 | 7.50E-288 |
| FQU82_RS17120 | rplN | 50S ribosomal protein L14 | -2.704612141 | 5.39E-283 | 3.14E-281 |
| FQU82_RS02280 | pnp | polyribonucleotide nucleotidyltransferase | -2.324328254 | 2.63E-276 | 1.51E-274 |
| FQU82_RS01310 | atpB | F0F1 ATP synthase subunit A | -2.468253917 | 2.52E-270 | 1.42E-268 |
| FQU82_RS18860 | hppD | 4-hydroxyphenylpyruvate dioxygenase | 2.376293721 | 1.33E-266 | 7.35E-265 |
| FQU82_RS18805 | hutU | urocanate hydratase | 3.107083503 | 6.42E-266 | 3.51E-264 |
| FQU82_RS17115 | rplX | 50S ribosomal protein L24 | -2.772485718 | 3.00E-258 | 1.62E-256 |
| FQU82_RS14800 | FQU82_RS14800 | sulfate ABC transporter ATP-binding protein | 2.903334233 | 2.31E-255 | 1.22E-253 |
| FQU82_RS00450 | FQU82_RS00450 | sulfonate ABC transporter substrate-binding protein | 4.660644839 | 1.19E-253 | 6.22E-252 |
| FQU82_RS18370 | FQU82_RS18370 | helix-turn-helix transcriptional regulator | 3.523300702 | 4.98E-253 | 2.56E-251 |
| Novel00129 | - | PF02190:ATP-dependent protease La (LON) substrate-binding domain|PF05362:Lon protease (S16) C-terminal proteolytic domain|PF00004:ATPase family associated with various cellular activities (AAA) | 3.609412808 | 2.11E-245 | 1.07E-243 |
| FQU82_RS18325 | sfnG | dimethyl sulfone monooxygenase SfnG | 4.179071507 | 2.51E-245 | 1.26E-243 |
| FQU82_RS01405 | FQU82_RS01405 | TonB-dependent copper receptor | -3.152039315 | 6.89E-243 | 3.40E-241 |
| FQU82_RS10770 | FQU82_RS10770 | carboxymuconolactone decarboxylase family protein | 3.131272293 | 2.43E-242 | 1.18E-240 |
| FQU82_RS14620 | FQU82_RS14620 | glyceraldehyde-3-phosphate dehydrogenase | -2.724605026 | 2.87E-242 | 1.38E-240 |
| FQU82_RS17440 | FQU82_RS17440 | Glu/Leu/Phe/Val dehydrogenase | -2.32038563 | 1.13E-236 | 5.33E-235 |
| FQU82_RS18320 | msuE | FMN reductase | 3.79732154 | 2.00E-235 | 9.36E-234 |
| FQU82_RS10765 | FQU82_RS10765 | NAD(P)-dependent alcohol dehydrogenase | 2.898944029 | 1.07E-230 | 4.94E-229 |
| FQU82_RS17880 | ettA | energy-dependent translational throttle protein EttA | -2.426889831 | 2.20E-230 | 1.00E-228 |
| FQU82_RS11300 | eno | phosphopyruvate hydratase | -2.508309267 | 4.19E-228 | 1.89E-226 |
| FQU82_RS10160 | FQU82_RS10160 | NAD(P)H-dependent oxidoreductase | 3.753330408 | 3.14E-226 | 1.40E-224 |
| FQU82_RS15300 | FQU82_RS15300 | phosphoenolpyruvate carboxykinase (GTP) | -2.94333319 | 3.78E-226 | 1.66E-224 |
| FQU82_RS10775 | FQU82_RS10775 | cupin domain-containing protein | 3.132343826 | 3.08E-222 | 1.33E-220 |
| FQU82_RS15400 | carB | carbamoyl-phosphate synthase large subunit | -2.397571506 | 3.50E-222 | 1.50E-220 |
| FQU82_RS13890 | ahcY | adenosylhomocysteinase | -2.620433112 | 1.23E-220 | 5.21E-219 |
| FQU82_RS13370 | adeF | multidrug efflux RND transporter periplasmic adaptor subunit AdeF | 5.382735247 | 4.25E-220 | 1.78E-218 |
| FQU82_RS12630 | rpsR | 30S ribosomal protein S18 | -2.278157396 | 8.95E-218 | 3.70E-216 |
| FQU82_RS09850 | FQU82_RS09850 | TonB-dependent receptor | -3.049884168 | 1.23E-213 | 5.00E-212 |
| FQU82_RS16745 | rpsI | 30S ribosomal protein S9 | -2.163930432 | 3.23E-213 | 1.30E-211 |
| FQU82_RS02165 | infB | translation initiation factor IF-2 | -1.90260356 | 2.17E-209 | 8.65E-208 |
| FQU82_RS02625 | gltP | glutamate/aspartate:proton symporter GltP | -2.431929989 | 1.41E-208 | 5.57E-207 |
| FQU82_RS14805 | FQU82_RS14805 | CysB family HTH-type transcriptional regulator | 2.510877945 | 1.32E-206 | 5.14E-205 |
| FQU82_RS09375 | sstT | serine/threonine transporter SstT | -2.957727652 | 8.83E-205 | 3.41E-203 |
| FQU82_RS12635 | rplI | 50S ribosomal protein L9 | -2.18585813 | 8.26E-199 | 3.16E-197 |
| FQU82_RS18845 | maiA | maleylacetoacetate isomerase | 2.25455008 | 1.16E-195 | 4.38E-194 |
| FQU82_RS04305 | rplY | 50S ribosomal protein L25 | -2.402722605 | 3.58E-195 | 1.34E-193 |
| FQU82_RS11450 | FQU82_RS11450 | cytochrome ubiquinol oxidase subunit I | -2.028297218 | 1.81E-194 | 6.70E-193 |
| FQU82_RS14635 | uvrB | excinuclease ABC subunit UvrB | 2.450415791 | 4.71E-192 | 1.73E-190 |
| FQU82_RS09415 | fba | fructose-bisphosphate aldolase class II | -2.10877699 | 8.27E-192 | 3.00E-190 |
| FQU82_RS01365 | FQU82_RS01365 | MFS transporter | 3.730796708 | 5.00E-189 | 1.79E-187 |
| FQU82_RS02515 | katG | catalase/peroxidase HPI | 2.221968859 | 2.34E-188 | 8.32E-187 |
| FQU82_RS14785 | FQU82_RS14785 | alpha/beta hydrolase | 2.469042204 | 2.65E-187 | 9.31E-186 |
| FQU82_RS12705 | purD | phosphoribosylamine--glycine ligase | -2.293994643 | 1.18E-184 | 4.10E-183 |
| FQU82_RS09550 | rpsA | 30S ribosomal protein S1 | -1.812883005 | 1.17E-183 | 4.04E-182 |
| FQU82_RS16740 | rplM | 50S ribosomal protein L13 | -2.227830531 | 7.05E-181 | 2.41E-179 |
| FQU82_RS14310 | purB | adenylosuccinate lyase | -2.223855236 | 8.66E-177 | 2.93E-175 |
| FQU82_RS12555 | FQU82_RS12555 | LLM class flavin-dependent oxidoreductase | 3.359240988 | 3.70E-175 | 1.24E-173 |
| FQU82_RS06045 | argG | argininosuccinate synthase | -2.387172158 | 4.67E-174 | 1.55E-172 |
| FQU82_RS19020 | FQU82_RS19020 | efflux RND transporter permease subunit | 2.325878139 | 4.69E-173 | 1.54E-171 |
| FQU82_RS18800 | hutH | histidine ammonia-lyase | 2.086999583 | 2.07E-172 | 6.74E-171 |
| FQU82_RS02705 | FQU82_RS02705 | hypothetical protein | 2.13989159 | 4.97E-171 | 1.61E-169 |
| FQU82_RS00215 | yidC | membrane protein insertase YidC | -2.020158147 | 3.77E-169 | 1.21E-167 |
| FQU82_RS15615 | rplU | 50S ribosomal protein L21 | -1.824047231 | 5.59E-169 | 1.77E-167 |
| FQU82_RS15885 | gatA | Asp-tRNA(Asn)/Glu-tRNA(Gln) amidotransferase subunit GatA | -2.251908547 | 3.95E-165 | 1.24E-163 |
| FQU82_RS12600 | cyoA | ubiquinol oxidase subunit II | -2.075259354 | 8.22E-165 | 2.56E-163 |
| FQU82_RS18850 | FQU82_RS18850 | VOC family protein | 2.16757723 | 9.87E-165 | 3.05E-163 |
| FQU82_RS17065 | rpmJ | 50S ribosomal protein L36 | -2.435206548 | 1.54E-164 | 4.71E-163 |
| FQU82_RS02160 | nusA | transcription termination factor NusA | -2.058414693 | 3.13E-161 | 9.51E-160 |
| FQU82_RS12625 | rpsF | 30S ribosomal protein S6 | -1.866419387 | 3.78E-161 | 1.14E-159 |
| FQU82_RS03425 | FQU82_RS03425 | hypothetical protein | -2.442860793 | 2.14E-159 | 6.38E-158 |
| FQU82_RS02910 | tig | trigger factor | -2.090211026 | 5.25E-159 | 1.55E-157 |
| FQU82_RS02895 | leuA | 2-isopropylmalate synthase | 1.687844175 | 1.14E-158 | 3.35E-157 |
| FQU82_RS15645 | adeJ | multidrug efflux RND transporter permease subunit AdeJ | -1.679776226 | 2.67E-157 | 7.77E-156 |
| FQU82_RS02720 | rpmB | 50S ribosomal protein L28 | -1.734349541 | 3.92E-157 | 1.13E-155 |
| FQU82_RS07990 | FQU82_RS07990 | dihydrodipicolinate synthase family protein | 4.165386958 | 8.10E-157 | 2.32E-155 |
| FQU82_RS15640 | adeI | multidrug efflux RND transporter periplasmic adaptor subunit AdeI | -1.782691508 | 9.80E-157 | 2.78E-155 |
| FQU82_RS04490 | rpsG | 30S ribosomal protein S7 | -1.750737305 | 3.36E-156 | 9.48E-155 |
| FQU82_RS16870 | FQU82_RS16870 | malate dehydrogenase | -2.130874683 | 1.00E-155 | 2.81E-154 |
| FQU82_RS01225 | guaA | glutamine-hydrolyzing GMP synthase | -2.074725187 | 2.77E-154 | 7.68E-153 |
| FQU82_RS00440 | ssuC | aliphatic sulfonate ABC transporter permease SsuC | 4.736165293 | 2.62E-153 | 7.20E-152 |
| FQU82_RS04485 | rpsL | 30S ribosomal protein S12 | -1.616160943 | 9.82E-153 | 2.68E-151 |
| FQU82_RS14975 | purM | phosphoribosylformylglycinamidine cyclo-ligase | -1.991123276 | 1.52E-152 | 4.13E-151 |
| FQU82_RS11535 | brnQ | branched-chain amino acid transport system II carrier protein | -2.989646129 | 3.80E-152 | 1.02E-150 |
| Novel00291 | - | PF03947:Ribosomal Proteins L2, C-terminal domain|PF00181:Ribosomal Proteins L2, RNA binding domain|PF07650:KH domain|PF00189:Ribosomal protein S3, C-terminal domain|PF00297:Ribosomal protein L3|PF03118:Bacterial RNA polymerase, alpha chain C terminal domain|PF01193:RNA polymerase Rpb3/Rpb11 dimerisation domain|PF01000:RNA polymerase Rpb3/RpoA insert domain|PF00573:Ribosomal protein L4/L1 family|PF00344:SecY|PF00281:Ribosomal protein L5|PF00673:ribosomal L5P family C-terminus|PF00163:Ribosomal protein S4/S9 N-terminal domain|PF01479:S4 domain | -2.462390835 | 3.39E-149 | 9.06E-148 |
| FQU82_RS08820 | tauD | taurine dioxygenase | 1.985246687 | 1.15E-147 | 3.05E-146 |
| FQU82_RS18765 | glmS | glutamine--fructose-6-phosphate transaminase (isomerizing) | -2.084699312 | 1.75E-147 | 4.59E-146 |
| FQU82_RS09280 | metK | methionine adenosyltransferase | -2.163625017 | 6.55E-146 | 1.71E-144 |
| FQU82_RS02430 | FQU82_RS02430 | acyl-CoA dehydrogenase family protein | 3.088934701 | 4.20E-144 | 1.09E-142 |
| FQU82_RS16450 | aspS | aspartate--tRNA ligase | -1.940928614 | 1.68E-143 | 4.32E-142 |
| FQU82_RS00455 | FQU82_RS00455 | sulfonate ABC transporter substrate-binding protein | 3.304744749 | 6.55E-142 | 1.67E-140 |
| FQU82_RS00205 | rnpA | ribonuclease P protein component | -2.359713279 | 1.63E-141 | 4.14E-140 |
| FQU82_RS09005 | ychF | redox-regulated ATPase YchF | -3.332840005 | 5.49E-140 | 1.38E-138 |
| FQU82_RS12605 | cyoB | cytochrome o ubiquinol oxidase subunit I | -1.664317683 | 4.43E-139 | 1.11E-137 |
| FQU82_RS12620 | cyoE | heme o synthase | -2.24390254 | 1.45E-137 | 3.61E-136 |
| FQU82_RS15575 | FQU82_RS15575 | flavodoxin family protein | 3.276388783 | 8.00E-137 | 1.97E-135 |
| FQU82_RS12235 | pepN | aminopeptidase N | 1.742893773 | 1.35E-136 | 3.30E-135 |
| FQU82_RS18095 | FQU82_RS18095 | aldo/keto reductase | 2.982067207 | 3.27E-135 | 7.96E-134 |
| FQU82_RS00435 | FQU82_RS00435 | ATP-binding cassette domain-containing protein | 5.377754664 | 2.03E-134 | 4.91E-133 |
| FQU82_RS17575 | rimM | ribosome maturation factor RimM | -1.885814178 | 3.15E-134 | 7.56E-133 |
| FQU82_RS16555 | secD | protein translocase subunit SecD | -1.948098852 | 6.73E-134 | 1.60E-132 |
| Novel00354 | - | PF01979:Amidohydrolase family|PF00491:Arginase family|PF00324:Amino acid permease|PF01175:Urocanase Rossmann-like domain|PF17392:Urocanase C-terminal domain|PF17391:Urocanase N-terminal domain|PF00221:Aromatic amino acid lyase | 2.961188844 | 1.07E-132 | 2.54E-131 |
| FQU82_RS15395 | carA | glutamine-hydrolyzing carbamoyl-phosphate synthase small subunit | -2.50509323 | 3.14E-132 | 7.38E-131 |
| FQU82_RS16170 | FQU82_RS16170 | amino acid permease | -2.642011646 | 6.61E-132 | 1.55E-130 |
| FQU82_RS11455 | cydB | cytochrome d ubiquinol oxidase subunit II | -1.932177205 | 2.29E-131 | 5.32E-130 |
| FQU82_RS09285 | tkt | transketolase | -1.907116327 | 2.55E-128 | 5.87E-127 |
| FQU82_RS10965 | FQU82_RS10965 | MBL fold metallo-hydrolase | 2.977464202 | 1.12E-127 | 2.58E-126 |
| FQU82_RS13895 | metF | methylenetetrahydrofolate reductase [NAD(P)H] | -2.501816563 | 1.40E-126 | 3.18E-125 |
| FQU82_RS01960 | FQU82_RS01960 | OmpW family outer membrane protein | -4.802118642 | 1.84E-126 | 4.15E-125 |
| FQU82_RS02425 | FQU82_RS02425 | SfnB family sulfur acquisition oxidoreductase | 2.925951152 | 2.11E-123 | 4.74E-122 |
| FQU82_RS19040 | FQU82_RS19040 | solute carrier family 23 protein | -2.888358538 | 4.12E-123 | 9.20E-122 |
| FQU82_RS03385 | rpmI | 50S ribosomal protein L35 | -1.598223007 | 5.00E-123 | 1.11E-121 |
| FQU82_RS11735 | frr | ribosome recycling factor | -1.975321153 | 1.28E-122 | 2.82E-121 |
| FQU82_RS03435 | rho | transcription termination factor Rho | -1.847733934 | 4.29E-121 | 9.41E-120 |
| FQU82_RS15650 | adeK | multidrug efflux RND transporter outer membrane channel subunit AdeK | -1.44611057 | 3.61E-120 | 7.87E-119 |
| Novel00009 | - | PF00012:Hsp70 protein|PF01025:GrpE | 2.370288068 | 1.55E-119 | 3.35E-118 |
| FQU82_RS08815 | tauC | taurine ABC transporter permease TauC | 1.70667015 | 1.91E-119 | 4.11E-118 |
| FQU82_RS14790 | cysT | sulfate ABC transporter permease subunit CysT | 2.310824997 | 1.55E-118 | 3.31E-117 |
| FQU82_RS03560 | raiA | ribosome-associated translation inhibitor RaiA | 2.191531367 | 3.46E-118 | 7.37E-117 |
| Novel00328 | - | PF08240:Alcohol dehydrogenase GroES-like domain|PF00107:Zinc-binding dehydrogenase|PF00440:Bacterial regulatory proteins, tetR family|PF16925:Tetracyclin repressor-like, C-terminal domain|PF06993:Protein of unknown function (DUF1304) | 2.381028805 | 5.33E-118 | 1.13E-116 |
| FQU82_RS14780 | FQU82_RS14780 | sulfate ABC transporter substrate-binding protein | 1.828016185 | 7.34E-118 | 1.54E-116 |
| FQU82_RS14815 | dapD | 2,3,4,5-tetrahydropyridine-2,6-dicarboxylate N-succinyltransferase | -2.096116171 | 1.49E-117 | 3.11E-116 |
| FQU82_RS02745 | FQU82_RS02745 | MotA/TolQ/ExbB proton channel family protein | -2.188744373 | 7.21E-117 | 1.50E-115 |
| FQU82_RS18795 | FQU82_RS18795 | amino acid permease | 1.767514405 | 7.84E-116 | 1.62E-114 |
| FQU82_RS00325 | dnaK | molecular chaperone DnaK | 1.621398392 | 3.43E-115 | 7.04E-114 |
| FQU82_RS00210 | yidD | membrane protein insertion efficiency factor YidD | -2.864873155 | 9.47E-115 | 1.93E-113 |
| FQU82_RS18265 | FQU82_RS18265 | TetR/AcrR family transcriptional regulator | 1.719221146 | 3.44E-114 | 6.98E-113 |
| FQU82_RS01690 | thrC | threonine synthase | -1.696232805 | 8.97E-114 | 1.81E-112 |
| FQU82_RS12610 | cyoC | cytochrome o ubiquinol oxidase subunit III | -1.487452327 | 2.67E-113 | 5.35E-112 |
| FQU82_RS05325 | lysS | lysine--tRNA ligase | -2.202973365 | 3.00E-113 | 5.99E-112 |
| FQU82_RS15875 | FQU82_RS15875 | rod shape-determining protein | -1.985377398 | 4.94E-113 | 9.79E-112 |
| FQU82_RS17815 | FQU82_RS17815 | amino acid permease | -2.413132665 | 1.05E-112 | 2.07E-111 |
| FQU82_RS18835 | FQU82_RS18835 | amino acid permease | 1.978038438 | 1.87E-112 | 3.68E-111 |
| FQU82_RS09755 | FQU82_RS09755 | Rrf2 family transcriptional regulator | 1.796268019 | 1.29E-111 | 2.52E-110 |
| FQU82_RS17365 | glyQ | glycine--tRNA ligase subunit alpha | -2.359640609 | 1.55E-111 | 3.01E-110 |
| FQU82_RS06895 | FQU82_RS06895 | adenylosuccinate synthase | -1.84490489 | 2.40E-111 | 4.62E-110 |
| FQU82_RS17565 | rplS | 50S ribosomal protein L19 | -1.624491337 | 2.50E-111 | 4.80E-110 |
| FQU82_RS14930 | FQU82_RS14930 | class 1 fructose-bisphosphatase | -2.847068012 | 8.02E-111 | 1.53E-109 |
| FQU82_RS17570 | trmD | tRNA (guanosine(37)-N1)-methyltransferase TrmD | -1.787717828 | 4.66E-109 | 8.85E-108 |
| FQU82_RS14825 | queC | 7-cyano-7-deazaguanine synthase QueC | -2.345139318 | 1.37E-108 | 2.59E-107 |
| FQU82_RS18260 | FQU82_RS18260 | zinc-binding dehydrogenase | 1.883458899 | 1.46E-108 | 2.75E-107 |
| FQU82_RS15470 | sucC | ADP-forming succinate--CoA ligase subunit beta | -1.902260827 | 1.51E-108 | 2.82E-107 |
| FQU82_RS06555 | FQU82_RS06555 | NCS2 family permease | -2.405367701 | 4.24E-108 | 7.89E-107 |
| FQU82_RS10780 | FQU82_RS10780 | AraC family transcriptional regulator | 3.113183662 | 8.37E-108 | 1.55E-106 |
| FQU82_RS14795 | cysW | sulfate ABC transporter permease subunit CysW | 2.533478744 | 8.83E-108 | 1.62E-106 |
| FQU82_RS15465 | sucD | succinate--CoA ligase subunit alpha | -1.609044676 | 5.17E-106 | 9.47E-105 |
| FQU82_RS01055 | FQU82_RS01055 | D-amino acid dehydrogenase | 1.847799853 | 3.29E-105 | 5.99E-104 |
| FQU82_RS17900 | FQU82_RS17900 | Tex family protein | -1.911240064 | 5.35E-105 | 9.69E-104 |
| FQU82_RS01925 | rpoB | DNA-directed RNA polymerase subunit beta | -1.572655582 | 2.00E-104 | 3.60E-103 |
| FQU82_RS03820 | FQU82_RS03820 | methionine synthase | -1.965584759 | 2.96E-104 | 5.31E-103 |
| FQU82_RS11305 | kdsA | 3-deoxy-8-phosphooctulonate synthase | -2.155373186 | 6.06E-104 | 1.08E-102 |
| FQU82_RS01060 | alr | alanine racemase | 2.128028782 | 1.93E-102 | 3.43E-101 |
| FQU82_RS09335 | putP | sodium/proline symporter PutP | -1.793650013 | 5.25E-102 | 9.28E-101 |
| FQU82_RS11710 | bamA | outer membrane protein assembly factor BamA | -1.64416347 | 3.57E-101 | 6.28E-100 |
| FQU82_RS14040 | FQU82_RS14040 | extended-spectrum class C beta-lactamase ADC-158 | -1.469784706 | 8.25E-101 | 1.44E-99 |
| FQU82_RS18030 | FQU82_RS18030 | hypothetical protein | -2.358703231 | 4.71E-100 | 8.20E-99 |
| FQU82_RS14870 | purL | phosphoribosylformylglycinamidine synthase | -1.834198681 | 7.01E-100 | 1.21E-98 |
| FQU82_RS18240 | FQU82_RS18240 | DUF475 domain-containing protein | -2.695815492 | 2.30E-99 | 3.97E-98 |
| FQU82_RS03390 | rplT | 50S ribosomal protein L20 | -1.55688201 | 4.70E-99 | 8.06E-98 |
| FQU82_RS02935 | FQU82_RS02935 | fumarate hydratase | -1.654344729 | 8.43E-99 | 1.44E-97 |
| FQU82_RS17295 | FQU82_RS17295 | DEAD/DEAH box helicase | -1.516530306 | 3.50E-98 | 5.95E-97 |
| FQU82_RS17425 | astD | succinylglutamate-semialdehyde dehydrogenase | -2.158053503 | 1.10E-96 | 1.87E-95 |
| FQU82_RS18280 | omp33-36 | porin Omp33-36 | -1.614962117 | 1.78E-96 | 3.00E-95 |
| FQU82_RS05475 | FQU82_RS05475 | META and DUF4377 domain-containing protein | -2.160196019 | 2.41E-96 | 4.04E-95 |
| FQU82_RS16215 | FQU82_RS16215 | nitrite/sulfite reductase | -1.866953962 | 3.44E-96 | 5.73E-95 |
| FQU82_RS12675 | mnmG | tRNA uridine-5-carboxymethylaminomethyl(34) synthesis enzyme MnmG | -1.792302647 | 8.59E-96 | 1.43E-94 |
| FQU82_RS07585 | FQU82_RS07585 | type I secretion system permease/ATPase | -2.197298767 | 2.22E-95 | 3.66E-94 |
| FQU82_RS14740 | lepA | translation elongation factor 4 | -1.972941502 | 3.60E-94 | 5.92E-93 |
| FQU82_RS01955 | FQU82_RS01955 | Na+/H+ antiporter NhaC family protein | -3.133064488 | 9.34E-94 | 1.53E-92 |
| FQU82_RS09040 | FQU82_RS09040 | SfnB family sulfur acquisition oxidoreductase | 2.647627032 | 1.23E-93 | 2.00E-92 |
| FQU82_RS19025 | FQU82_RS19025 | efflux RND transporter periplasmic adaptor subunit | 2.225436955 | 1.59E-93 | 2.57E-92 |
| FQU82_RS15610 | rpmA | 50S ribosomal protein L27 | -1.786105985 | 7.04E-93 | 1.14E-91 |
| FQU82_RS16560 | secF | protein translocase subunit SecF | -1.657863237 | 1.26E-92 | 2.03E-91 |
| FQU82_RS02170 | FQU82_RS02170 | ribosome-binding factor A | -1.772893095 | 1.39E-92 | 2.23E-91 |
| FQU82_RS15680 | FQU82_RS15680 | valine--tRNA ligase | -1.557511399 | 1.59E-92 | 2.54E-91 |
| FQU82_RS16160 | FQU82_RS16160 | uracil-xanthine permease family protein | -3.18215839 | 5.44E-92 | 8.62E-91 |
| Novel00122 | - | PF13356:Arm DNA-binding domain | 1.693745134 | 2.33E-91 | 3.68E-90 |
| FQU82_RS11940 | accC | acetyl-CoA carboxylase biotin carboxylase subunit | -1.864674027 | 7.74E-91 | 1.22E-89 |
| FQU82_RS13365 | adeL | multidrug efflux transcriptional repressor AdeL | 2.593964713 | 1.40E-90 | 2.20E-89 |
| FQU82_RS05230 | nfuA | Fe-S biogenesis protein NfuA | 1.545634189 | 1.50E-90 | 2.33E-89 |
| FQU82_RS01900 | nusG | transcription termination/antitermination protein NusG | -1.617102188 | 6.53E-90 | 1.01E-88 |
| FQU82_RS17370 | glyS | glycine--tRNA ligase subunit beta | -1.920321513 | 8.47E-90 | 1.31E-88 |
| FQU82_RS11790 | FQU82_RS11790 | D-amino acid dehydrogenase | 1.755354611 | 1.06E-89 | 1.63E-88 |
| FQU82_RS01370 | FQU82_RS01370 | TetR/AcrR family transcriptional regulator | 2.675197879 | 2.83E-89 | 4.33E-88 |
| FQU82_RS13385 | FQU82_RS13385 | serine hydroxymethyltransferase | -1.517815144 | 7.82E-89 | 1.19E-87 |
| FQU82_RS09750 | FQU82_RS09750 | IscS subfamily cysteine desulfurase | 1.309445893 | 1.16E-88 | 1.76E-87 |
| FQU82_RS09035 | FQU82_RS09035 | SfnB family sulfur acquisition oxidoreductase | 3.139207954 | 3.18E-88 | 4.81E-87 |
| FQU82_RS08785 | FQU82_RS08785 | monooxygenase | 2.486441477 | 2.15E-87 | 3.24E-86 |
| FQU82_RS10895 | FQU82_RS10895 | DUF637 domain-containing protein | -3.034113456 | 3.04E-87 | 4.56E-86 |
| FQU82_RS14470 | FQU82_RS14470 | L-cystine transporter | 1.492834386 | 1.61E-86 | 2.40E-85 |
| FQU82_RS14215 | efp | elongation factor P | -1.684399421 | 4.34E-86 | 6.46E-85 |
| FQU82_RS01075 | FQU82_RS01075 | amino acid permease | -2.253307789 | 7.57E-86 | 1.12E-84 |
| FQU82_RS10520 | adeA | multidrug efflux RND transporter periplasmic adaptor subunit AdeA | -2.348730922 | 1.42E-85 | 2.10E-84 |
| FQU82_RS11425 | FQU82_RS11425 | TonB-dependent siderophore receptor | -3.307637471 | 2.82E-84 | 4.14E-83 |
| FQU82_RS15160 | FQU82_RS15160 | FdhF/YdeP family oxidoreductase | 1.501758665 | 4.75E-84 | 6.94E-83 |
| FQU82_RS11740 | pyrH | UMP kinase | -2.557729449 | 2.95E-83 | 4.29E-82 |
| Novel00335 | - | PF00724:NADH:flavin oxidoreductase / NADH oxidase family | 4.466061517 | 5.54E-83 | 8.03E-82 |
| FQU82_RS14285 | dacC | D-alanyl-D-alanine carboxypeptidase PBP5/6 | -2.471666072 | 1.47E-82 | 2.13E-81 |
| FQU82_RS16550 | yajC | preprotein translocase subunit YajC | -2.52093057 | 2.04E-82 | 2.93E-81 |
| FQU82_RS10515 | adeB | multidrug efflux RND transporter permease subunit AdeB | -1.857777479 | 3.51E-82 | 5.02E-81 |
| FQU82_RS17760 | mraY | phospho-N-acetylmuramoyl-pentapeptide-transferase | -1.72723096 | 2.45E-81 | 3.49E-80 |
| FQU82_RS18760 | glmU | bifunctional UDP-N-acetylglucosamine diphosphorylase/glucosamine-1-phosphate N-acetyltransferase GlmU | -1.95120487 | 5.77E-81 | 8.20E-80 |
| FQU82_RS00125 | tyrS | tyrosine--tRNA ligase | -1.679102934 | 5.90E-81 | 8.34E-80 |
| FQU82_RS00895 | FQU82_RS00895 | phosphomannomutase/phosphoglucomutase | -2.039395586 | 3.26E-79 | 4.60E-78 |
| FQU82_RS14175 | FQU82_RS14175 | pirin-like bicupin family protein | 2.801593078 | 4.75E-79 | 6.66E-78 |
| FQU82_RS11365 | clpA | ATP-dependent Clp protease ATP-binding subunit ClpA | 1.156556068 | 9.07E-79 | 1.27E-77 |
| FQU82_RS16290 | FQU82_RS16290 | peroxiredoxin | 1.550376294 | 7.84E-78 | 1.09E-76 |
| FQU82_RS15890 | gatB | Asp-tRNA(Asn)/Glu-tRNA(Gln) amidotransferase subunit GatB | -1.795914234 | 8.65E-78 | 1.20E-76 |
| FQU82_RS03440 | trxA | thioredoxin | 1.596548605 | 2.04E-77 | 2.82E-76 |
| FQU82_RS06560 | FQU82_RS06560 | adenosine deaminase | -2.022709914 | 5.56E-77 | 7.65E-76 |
| FQU82_RS10055 | prmB | 50S ribosomal protein L3 N(5)-glutamine methyltransferase | -2.56211529 | 1.16E-76 | 1.59E-75 |
| FQU82_RS05935 | FQU82_RS05935 | cation diffusion facilitator family transporter | -2.06355719 | 6.48E-76 | 8.85E-75 |
| FQU82_RS18570 | FQU82_RS18570 | outer membrane protein OmpK | -3.742596243 | 1.09E-75 | 1.49E-74 |
| FQU82_RS17420 | astB | N-succinylarginine dihydrolase | -1.819173636 | 2.48E-75 | 3.36E-74 |
| FQU82_RS08810 | FQU82_RS08810 | ATP-binding cassette domain-containing protein | 1.491109804 | 3.52E-75 | 4.75E-74 |
| FQU82_RS04510 | FQU82_RS04510 | metal-dependent hydrolase | 2.523363166 | 7.51E-75 | 1.01E-73 |
| FQU82_RS03495 | FQU82_RS03495 | organic hydroperoxide resistance protein | 1.689656471 | 2.11E-74 | 2.83E-73 |
| FQU82_RS11310 | FQU82_RS11310 | CTP synthase | -1.339858839 | 2.63E-74 | 3.52E-73 |
| FQU82_RS05495 | ata | trimeric autotransporter adhesin Ata | -3.355161844 | 2.94E-74 | 3.91E-73 |
| FQU82_RS02950 | edd | phosphogluconate dehydratase | -1.67007619 | 9.93E-74 | 1.32E-72 |
| FQU82_RS15570 | FQU82_RS15570 | APC family permease | -1.652959796 | 2.72E-73 | 3.59E-72 |
| FQU82_RS15805 | FQU82_RS15805 | undecaprenyl-diphosphate phosphatase | -2.520413885 | 1.10E-72 | 1.45E-71 |
| FQU82_RS09745 | iscU | Fe-S cluster assembly scaffold IscU | 1.678693967 | 1.28E-72 | 1.67E-71 |
| FQU82_RS08035 | FQU82_RS08035 | aldehyde dehydrogenase (NADP(+)) | 4.475922279 | 2.09E-72 | 2.73E-71 |
| FQU82_RS14345 | FQU82_RS14345 | substrate-binding domain-containing protein | -2.774800577 | 2.55E-72 | 3.32E-71 |
| FQU82_RS14940 | FQU82_RS14940 | RNA polymerase sigma factor | 1.631649017 | 2.96E-72 | 3.83E-71 |
| FQU82_RS02305 | FQU82_RS02305 | Hsp33 family molecular chaperone HslO | 1.421435513 | 4.46E-72 | 5.76E-71 |
| FQU82_RS16915 | FQU82_RS16915 | xanthine phosphoribosyltransferase | -2.196065563 | 2.93E-71 | 3.77E-70 |
| FQU82_RS09935 | FQU82_RS09935 | TonB-dependent siderophore receptor | -3.423027373 | 8.37E-71 | 1.07E-69 |
| FQU82_RS17525 | serA | phosphoglycerate dehydrogenase | -1.604273545 | 2.13E-70 | 2.72E-69 |
| Novel00169 | - | PF16970:Type-1 fimbrial protein, A | 4.530738999 | 2.70E-70 | 3.44E-69 |
| FQU82_RS08040 | FQU82_RS08040 | APC family permease | 4.624891292 | 4.25E-70 | 5.39E-69 |
| FQU82_RS14735 | lepB | signal peptidase I | -1.717640281 | 4.51E-70 | 5.70E-69 |
| FQU82_RS18790 | hutI | imidazolonepropionase | 1.484014041 | 2.30E-69 | 2.89E-68 |
| FQU82_RS11595 | FQU82_RS11595 | universal stress protein | 1.691747711 | 2.75E-69 | 3.45E-68 |
| FQU82_RS15330 | folD | bifunctional methylenetetrahydrofolate dehydrogenase/methenyltetrahydrofolate cyclohydrolase FolD | -2.025293696 | 1.56E-68 | 1.95E-67 |
| FQU82_RS17890 | FQU82_RS17890 | SDR family NAD(P)-dependent oxidoreductase | -2.924863686 | 2.30E-68 | 2.86E-67 |
| FQU82_RS01895 | secE | preprotein translocase subunit SecE | -1.756746357 | 2.92E-68 | 3.62E-67 |
| FQU82_RS15550 | FQU82_RS15550 | DUF493 domain-containing protein | 1.769349538 | 1.25E-67 | 1.54E-66 |
| FQU82_RS00330 | grpE | nucleotide exchange factor GrpE | 1.51717347 | 9.82E-67 | 1.21E-65 |
| FQU82_RS09390 | FQU82_RS09390 | cupin domain-containing protein | -2.258829235 | 1.97E-66 | 2.42E-65 |
| FQU82_RS06880 | alaS | alanine--tRNA ligase | -1.452157212 | 9.17E-66 | 1.12E-64 |
| FQU82_RS11730 | uppS | polyprenyl diphosphate synthase | -2.197625286 | 1.24E-65 | 1.51E-64 |
| FQU82_RS15075 | gyrA | DNA gyrase subunit A | -1.266719186 | 1.30E-65 | 1.59E-64 |
| FQU82_RS03145 | ilvC | ketol-acid reductoisomerase | -1.177009888 | 3.54E-65 | 4.29E-64 |
| FQU82_RS00875 | galU | UTP--glucose-1-phosphate uridylyltransferase GalU | -1.527577329 | 3.62E-65 | 4.37E-64 |
| FQU82_RS05195 | FQU82_RS05195 | inorganic phosphate transporter | -1.391462347 | 5.17E-65 | 6.22E-64 |
| FQU82_RS14980 | purN | phosphoribosylglycinamide formyltransferase | -2.048524396 | 6.08E-65 | 7.29E-64 |
| FQU82_RS14820 | queE | 7-carboxy-7-deazaguanine synthase QueE | -2.229700658 | 1.28E-64 | 1.54E-63 |
| Novel00269 | - | PF00679:Elongation factor G C-terminus|PF00009:Elongation factor Tu GTP binding domain|PF03144:Elongation factor Tu domain 2|PF21018:TypA/BipA C-terminal domain | -2.690799665 | 3.44E-64 | 4.10E-63 |
| FQU82_RS03125 | leuS | leucine--tRNA ligase | -1.480380229 | 5.59E-64 | 6.64E-63 |
| FQU82_RS13245 | FQU82_RS13245 | dicarboxylate/amino acid:cation symporter | -1.560829564 | 7.26E-64 | 8.59E-63 |
| FQU82_RS03900 | bfmR | response regulator transcription factor BfmR | -1.337988378 | 1.48E-63 | 1.75E-62 |
| FQU82_RS17430 | astA | arginine N-succinyltransferase | -2.421406629 | 1.75E-63 | 2.06E-62 |
| FQU82_RS02275 | rpsO | 30S ribosomal protein S15 | -1.282711398 | 7.69E-63 | 9.01E-62 |
| FQU82_RS15085 | FQU82_RS15085 | electron transfer flavoprotein subunit beta/FixA family protein | -1.143734169 | 1.06E-62 | 1.24E-61 |
| FQU82_RS06570 | FQU82_RS06570 | hypothetical protein | -2.359612233 | 1.44E-62 | 1.68E-61 |
| FQU82_RS01930 | rpoC | DNA-directed RNA polymerase subunit beta | -1.021762272 | 3.07E-62 | 3.56E-61 |
| FQU82_RS15475 | lpdA | dihydrolipoyl dehydrogenase | -1.299870547 | 5.71E-62 | 6.61E-61 |
| Novel00114 | - | PF00164:Ribosomal protein S12/S23|PF10118:Predicted metal-dependent hydrolase|PF00177:Ribosomal protein S7p/S5e|PF03143:Elongation factor Tu C-terminal domain|PF03144:Elongation factor Tu domain 2|PF00009:Elongation factor Tu GTP binding domain|PF03764:Elongation factor G, domain IV|PF00679:Elongation factor G C-terminus|PF14492:Elongation Factor G, domain III | -1.693502673 | 6.50E-62 | 7.49E-61 |
| FQU82_RS11745 | rimO | 30S ribosomal protein S12 methylthiotransferase RimO | -1.822619952 | 1.86E-61 | 2.14E-60 |
| FQU82_RS04255 | fabG | 3-oxoacyl-ACP reductase FabG | -1.461818676 | 1.37E-60 | 1.57E-59 |
| FQU82_RS11545 | FQU82_RS11545 | hypothetical protein | 2.236226823 | 2.41E-60 | 2.75E-59 |
| FQU82_RS03940 | nuoF | NADH-quinone oxidoreductase subunit NuoF | -1.246323494 | 3.69E-60 | 4.20E-59 |
| FQU82_RS15080 | FQU82_RS15080 | FAD-binding protein | -1.050757673 | 6.23E-60 | 7.07E-59 |
| FQU82_RS13910 | FQU82_RS13910 | NADP-dependent malic enzyme | -1.287316571 | 6.96E-60 | 7.88E-59 |
| FQU82_RS02955 | eda | bifunctional 4-hydroxy-2-oxoglutarate aldolase/2-dehydro-3-deoxy-phosphogluconate aldolase | -1.676531154 | 8.80E-60 | 9.93E-59 |
| FQU82_RS12310 | FQU82_RS12310 | peptidylprolyl isomerase | -1.44590164 | 1.01E-59 | 1.14E-58 |
| FQU82_RS03260 | rsfS | ribosome silencing factor | 1.261227598 | 1.27E-59 | 1.42E-58 |
| FQU82_RS15870 | mreC | rod shape-determining protein MreC | -1.793586073 | 3.39E-59 | 3.78E-58 |
| FQU82_RS17965 | hisA | 1-(5-phosphoribosyl)-5-[(5-phosphoribosylamino)methylideneamino]imidazole-4-carboxamide isomerase | -1.513766953 | 3.86E-59 | 4.29E-58 |
| FQU82_RS02755 | msrA | peptide-methionine (S)-S-oxide reductase MsrA | -1.261905706 | 3.89E-59 | 4.32E-58 |
| FQU82_RS14335 | pstA | phosphate ABC transporter permease PstA | -1.843733598 | 1.23E-58 | 1.36E-57 |
| Novel00214 | - | PF00318:Ribosomal protein S2|PF00889:Elongation factor TS | -2.162314895 | 1.88E-58 | 2.07E-57 |
| FQU82_RS03190 | acnA | aconitate hydratase AcnA | 1.15681411 | 2.13E-58 | 2.34E-57 |
| FQU82_RS13845 | map | type I methionyl aminopeptidase | -1.653632081 | 4.04E-58 | 4.43E-57 |
| FQU82_RS17920 | FQU82_RS17920 | acetyl-CoA hydrolase/transferase family protein | -1.416981588 | 4.07E-58 | 4.44E-57 |
| FQU82_RS14460 | FQU82_RS14460 | pitrilysin family protein | -1.380489283 | 4.61E-58 | 5.02E-57 |
| FQU82_RS04580 | rep | DNA helicase Rep | -1.965958071 | 7.21E-58 | 7.84E-57 |
| FQU82_RS09030 | FQU82_RS09030 | LLM class flavin-dependent oxidoreductase | 3.808460152 | 1.86E-57 | 2.02E-56 |
| FQU82_RS11725 | FQU82_RS11725 | phosphatidate cytidylyltransferase | -1.956411203 | 2.11E-57 | 2.27E-56 |
| FQU82_RS12280 | FQU82_RS12280 | aldehyde dehydrogenase family protein | -2.22425009 | 2.19E-57 | 2.35E-56 |
| FQU82_RS16545 | tgt | tRNA guanosine(34) transglycosylase Tgt | -1.884966215 | 4.72E-57 | 5.07E-56 |
| FQU82_RS12715 | FQU82_RS12715 | methionine ABC transporter ATP-binding protein | -1.646357308 | 1.17E-56 | 1.25E-55 |
| FQU82_RS08370 | FQU82_RS08370 | acyl-CoA dehydrogenase family protein | -2.252738215 | 1.80E-56 | 1.92E-55 |
| FQU82_RS07865 | FQU82_RS07865 | type VI secretion system tube protein Hcp | 1.210573239 | 1.84E-56 | 1.95E-55 |
| FQU82_RS16515 | FQU82_RS16515 | HAMP domain-containing sensor histidine kinase | 1.498691279 | 3.10E-56 | 3.29E-55 |
| FQU82_RS03570 | murA | UDP-N-acetylglucosamine 1-carboxyvinyltransferase | -1.401466774 | 3.37E-56 | 3.56E-55 |
| FQU82_RS18895 | FQU82_RS18895 | phosphoribosylaminoimidazolesuccinocarboxamide synthase | -1.260868557 | 4.09E-56 | 4.32E-55 |
| FQU82_RS15010 | FQU82_RS15010 | lipoprotein-releasing ABC transporter permease subunit | -1.582479498 | 8.12E-56 | 8.53E-55 |
| FQU82_RS15995 | FQU82_RS15995 | tRNA-Asp | -2.62356873 | 8.39E-56 | 8.79E-55 |
| FQU82_RS13330 | yegQ | tRNA 5-hydroxyuridine modification protein YegQ | -2.732260056 | 1.09E-55 | 1.14E-54 |
| FQU82_RS02135 | tpiA | triose-phosphate isomerase | -1.849532062 | 1.66E-55 | 1.73E-54 |
| FQU82_RS02785 | lgt | prolipoprotein diacylglyceryl transferase | -1.868414069 | 3.75E-55 | 3.89E-54 |
| FQU82_RS02960 | FQU82_RS02960 | gluconate:H+ symporter | -1.626585276 | 7.78E-55 | 8.05E-54 |
| FQU82_RS17795 | FQU82_RS17795 | hypothetical protein | 1.200595417 | 8.84E-55 | 9.12E-54 |
| FQU82_RS16025 | dapE | succinyl-diaminopimelate desuccinylase | -2.053093151 | 9.42E-55 | 9.70E-54 |
| FQU82_RS00900 | lldP | L-lactate permease | 2.255126048 | 1.12E-54 | 1.15E-53 |
| FQU82_RS10060 | aroC | chorismate synthase | -1.753571362 | 1.17E-54 | 1.20E-53 |
| FQU82_RS18740 | ribE | 6,7-dimethyl-8-ribityllumazine synthase | -1.276359027 | 2.55E-54 | 2.60E-53 |
| FQU82_RS18310 | FQU82_RS18310 | hypothetical protein | 1.867637426 | 4.46E-54 | 4.54E-53 |
| FQU82_RS03975 | nuoM | NADH-quinone oxidoreductase subunit M | -1.119052528 | 9.75E-54 | 9.89E-53 |
| FQU82_RS14340 | pstC | phosphate ABC transporter permease subunit PstC | -2.429938701 | 3.62E-53 | 3.67E-52 |
| FQU82_RS08790 | FQU82_RS08790 | LLM class flavin-dependent oxidoreductase | 3.233768097 | 7.16E-53 | 7.22E-52 |
| FQU82_RS09220 | FQU82_RS09220 | hypothetical protein | 1.511535036 | 8.62E-53 | 8.68E-52 |
| FQU82_RS02020 | lpxO | lipid A hydroxylase LpxO | -1.666605802 | 1.26E-52 | 1.27E-51 |
| FQU82_RS04170 | bfr | bacterioferritin | 1.120637841 | 2.11E-52 | 2.11E-51 |
| FQU82_RS03970 | nuoL | NADH-quinone oxidoreductase subunit L | -1.063353327 | 3.91E-52 | 3.90E-51 |
| FQU82_RS06960 | FQU82_RS06960 | aspartate carbamoyltransferase catalytic subunit | -2.078355305 | 5.59E-52 | 5.56E-51 |
| FQU82_RS04960 | mqo | malate dehydrogenase (quinone) | -1.114154833 | 5.85E-52 | 5.81E-51 |
| FQU82_RS00140 | erpA | iron-sulfur cluster insertion protein ErpA | 1.529504671 | 1.85E-51 | 1.83E-50 |
| FQU82_RS18515 | FQU82_RS18515 | DUF2147 domain-containing protein | 1.210312954 | 1.93E-51 | 1.90E-50 |
| FQU82_RS04610 | FQU82_RS04610 | bacteriohemerythrin | -3.141332621 | 4.66E-51 | 4.58E-50 |
| FQU82_RS07000 | trmB | tRNA (guanosine(46)-N7)-methyltransferase TrmB | -2.900743597 | 8.61E-51 | 8.45E-50 |
| FQU82_RS18145 | FQU82_RS18145 | pirin family protein | 2.22428964 | 1.27E-50 | 1.24E-49 |
| FQU82_RS15235 | ttcA | tRNA 2-thiocytidine(32) synthetase TtcA | -2.230382911 | 1.96E-50 | 1.91E-49 |
| FQU82_RS13085 | rpsU | 30S ribosomal protein S21 | -1.023381299 | 3.25E-50 | 3.17E-49 |
| FQU82_RS18745 | nusB | transcription antitermination factor NusB | -1.32155081 | 3.34E-50 | 3.24E-49 |
| FQU82_RS17340 | FQU82_RS17340 | PspC domain-containing protein | 1.598194077 | 4.49E-50 | 4.34E-49 |
| FQU82_RS04335 | ispE | 4-(cytidine 5'-diphospho)-2-C-methyl-D-erythritol kinase | -1.478019239 | 7.92E-50 | 7.64E-49 |
| FQU82_RS13800 | mltB | lytic murein transglycosylase B | -2.366312806 | 8.17E-50 | 7.86E-49 |
| FQU82_RS06585 | FQU82_RS06585 | aspartate kinase | -1.418607143 | 3.62E-49 | 3.47E-48 |
| FQU82_RS03945 | nuoG | NADH-quinone oxidoreductase subunit NuoG | -0.94300379 | 4.26E-49 | 4.08E-48 |
| FQU82_RS15480 | odhB | 2-oxoglutarate dehydrogenase complex dihydrolipoyllysine-residue succinyltransferase | -0.974593914 | 6.74E-49 | 6.43E-48 |
| FQU82_RS01560 | ppa | inorganic diphosphatase | -1.577202033 | 1.60E-48 | 1.52E-47 |
| FQU82_RS03980 | nuoN | NADH-quinone oxidoreductase subunit NuoN | -1.063901543 | 4.93E-48 | 4.68E-47 |
| FQU82_RS13315 | FQU82_RS13315 | ferredoxin--NADP reductase | -1.395104783 | 1.30E-47 | 1.23E-46 |
| FQU82_RS12720 | FQU82_RS12720 | methionine ABC transporter permease | -1.595155474 | 2.26E-47 | 2.14E-46 |
| FQU82_RS17875 | FQU82_RS17875 | SEL1-like repeat protein | 1.388972458 | 2.85E-47 | 2.69E-46 |
| FQU82_RS14630 | FQU82_RS14630 | lipocalin family protein | 1.593666058 | 4.46E-47 | 4.19E-46 |
| FQU82_RS16990 | FQU82_RS16990 | M3 family metallopeptidase | 1.284444536 | 1.77E-46 | 1.66E-45 |
| FQU82_RS01660 | gpmI | 2,3-bisphosphoglycerate-independent phosphoglycerate mutase | -1.266820965 | 2.71E-46 | 2.54E-45 |
| FQU82_RS09080 | FQU82_RS09080 | YebC/PmpR family DNA-binding transcriptional regulator | -1.468039139 | 1.26E-45 | 1.18E-44 |
| Novel00014 | - | PF00296:Luciferase-like monooxygenase|PF00005:ABC transporter|PF00528:Binding-protein-dependent transport system inner membrane component|PF13379:NMT1-like family | 3.561074377 | 3.38E-45 | 3.15E-44 |
| FQU82_RS05450 | adk | adenylate kinase | -1.009311505 | 3.90E-45 | 3.62E-44 |
| FQU82_RS14945 | FQU82_RS14945 | hypothetical protein | 1.741702256 | 6.35E-45 | 5.88E-44 |
| FQU82_RS09775 | FQU82_RS09775 | SurA N-terminal domain-containing protein | -1.238874395 | 1.49E-44 | 1.38E-43 |
| Novel00170 | - | PF07883:Cupin domain|PF00107:Zinc-binding dehydrogenase|PF08240:Alcohol dehydrogenase GroES-like domain|PF02627:Carboxymuconolactone decarboxylase family | 3.285562775 | 1.87E-44 | 1.72E-43 |
| FQU82_RS18495 | FQU82_RS18495 | TonB-dependent siderophore receptor | -2.998578545 | 2.31E-44 | 2.12E-43 |
| FQU82_RS10355 | antA | anthranilate 1,2-dioxygenase large subunit | 7.754300776 | 3.48E-44 | 3.18E-43 |
| FQU82_RS16860 | FQU82_RS16860 | hypothetical protein | 1.365398794 | 5.01E-44 | 4.58E-43 |
| FQU82_RS15505 | sdhA | succinate dehydrogenase flavoprotein subunit | -1.030876007 | 7.68E-44 | 6.99E-43 |
| FQU82_RS17495 | FQU82_RS17495 | MFS transporter | -1.521811181 | 8.97E-44 | 8.15E-43 |
| FQU82_RS05025 | pgaB | poly-beta-1,6-N-acetyl-D-glucosamine N-deacetylase PgaB | -1.3464004 | 2.08E-43 | 1.88E-42 |
| FQU82_RS15460 | trpS | tryptophan--tRNA ligase | -1.537583249 | 5.47E-43 | 4.94E-42 |
| FQU82_RS12505 | FQU82_RS12505 | AMP-binding protein | 2.864496915 | 6.44E-43 | 5.81E-42 |
| FQU82_RS03170 | FQU82_RS03170 | peptide chain release factor 3 | -1.947186069 | 1.00E-42 | 9.00E-42 |
| FQU82_RS03555 | FQU82_RS03555 | RNA polymerase factor sigma-54 | 1.305828566 | 1.64E-42 | 1.47E-41 |
| FQU82_RS16165 | FQU82_RS16165 | class I SAM-dependent methyltransferase | -2.670183387 | 2.75E-42 | 2.46E-41 |
| FQU82_RS17300 | FQU82_RS17300 | inositol monophosphatase family protein | -1.370603368 | 4.10E-42 | 3.66E-41 |
| FQU82_RS09325 | putA | trifunctional transcriptional regulator/proline dehydrogenase/L-glutamate gamma-semialdehyde dehydrogenase | -1.115319756 | 5.26E-42 | 4.68E-41 |
| FQU82_RS02940 | pta | phosphate acetyltransferase | -1.040239631 | 6.84E-42 | 6.08E-41 |
| FQU82_RS01070 | FQU82_RS01070 | amino acid permease | 1.406585663 | 7.42E-42 | 6.57E-41 |
| FQU82_RS12550 | FQU82_RS12550 | hypothetical protein | 1.817182257 | 2.33E-41 | 2.06E-40 |
| FQU82_RS12685 | prmA | 50S ribosomal protein L11 methyltransferase | -1.471323338 | 2.61E-41 | 2.30E-40 |
| FQU82_RS11215 | FQU82_RS11215 | DUF6438 domain-containing protein | -1.968520018 | 2.70E-41 | 2.37E-40 |
| FQU82_RS07590 | FQU82_RS07590 | HlyD family type I secretion periplasmic adaptor subunit | -1.786084959 | 2.73E-41 | 2.40E-40 |
| FQU82_RS02770 | thyA | thymidylate synthase | -1.161956101 | 3.54E-41 | 3.10E-40 |
| FQU82_RS12615 | FQU82_RS12615 | cytochrome o ubiquinol oxidase subunit IV | -1.295417563 | 4.07E-41 | 3.55E-40 |
| FQU82_RS03135 | FQU82_RS03135 | acetolactate synthase 3 large subunit | -0.910874112 | 6.06E-41 | 5.27E-40 |
| Novel00193 | - | PF17432:Domain of unknown function (DUF3458_C) ARM repeats|PF11940:Domain of unknown function (DUF3458) Ig-like fold|PF01433:Peptidase family M1 domain|PF17900:Peptidase M1 N-terminal domain | 2.124122516 | 6.10E-41 | 5.30E-40 |
| FQU82_RS18430 | FQU82_RS18430 | 2-oxo acid dehydrogenase subunit E2 | -0.919662176 | 1.41E-40 | 1.22E-39 |
| FQU82_RS06950 | rlmKL | bifunctional 23S rRNA (guanine(2069)-N(7))-methyltransferase RlmK/23S rRNA (guanine(2445)-N(2))-methyltransferase RlmL | -1.459611864 | 1.51E-40 | 1.30E-39 |
| FQU82_RS01985 | FQU82_RS01985 | DsbA family oxidoreductase | 1.57833228 | 2.16E-40 | 1.86E-39 |
| FQU82_RS08900 | FQU82_RS08900 | hypothetical protein | -1.492294924 | 2.33E-40 | 2.00E-39 |
| Novel00061 | - | PF13602:Zinc-binding dehydrogenase | 4.198310009 | 3.32E-40 | 2.85E-39 |
| FQU82_RS04500 | tuf | elongation factor Tu | -2.357066684 | 5.04E-40 | 4.31E-39 |
| FQU82_RS02735 | purU | formyltetrahydrofolate deformylase | -1.603470472 | 6.21E-40 | 5.30E-39 |
| FQU82_RS04060 | metG | methionine--tRNA ligase | -1.134659756 | 9.16E-40 | 7.81E-39 |
| FQU82_RS18435 | aceE | pyruvate dehydrogenase (acetyl-transferring), homodimeric type | -0.987266925 | 1.18E-39 | 1.00E-38 |
| FQU82_RS00890 | galE | UDP-glucose 4-epimerase GalE | -1.356321608 | 1.30E-39 | 1.10E-38 |
| FQU82_RS03935 | nuoE | NADH-quinone oxidoreductase subunit NuoE | -1.118552764 | 1.98E-39 | 1.68E-38 |
| FQU82_RS18375 | prfB | peptide chain release factor 2 | -1.168385248 | 3.90E-39 | 3.29E-38 |
| FQU82_RS04560 | minC | septum site-determining protein MinC | -1.693325064 | 5.63E-39 | 4.74E-38 |
| FQU82_RS12115 | FQU82_RS12115 | universal stress protein | 1.059550836 | 6.57E-39 | 5.51E-38 |
| FQU82_RS14855 | FQU82_RS14855 | enoyl-CoA hydratase-related protein | 0.948339258 | 1.06E-38 | 8.86E-38 |
| FQU82_RS15405 | greA | transcription elongation factor GreA | -1.318886577 | 1.07E-38 | 8.94E-38 |
| FQU82_RS01790 | argH | argininosuccinate lyase | -1.221621632 | 1.25E-38 | 1.05E-37 |
| FQU82_RS03815 | FQU82_RS03815 | DUF1852 domain-containing protein | -1.510196417 | 1.44E-38 | 1.20E-37 |
| FQU82_RS04985 | FQU82_RS04985 | choline transporter | -1.51338179 | 4.02E-38 | 3.33E-37 |
| FQU82_RS04710 | ssrA | transfer-messenger RNA | 0.881186667 | 6.52E-38 | 5.40E-37 |
| FQU82_RS18720 | FQU82_RS18720 | hypothetical protein | 1.068178524 | 1.40E-37 | 1.16E-36 |
| FQU82_RS18090 | FQU82_RS18090 | helix-turn-helix transcriptional regulator | 2.270177737 | 1.60E-37 | 1.32E-36 |
| FQU82_RS03930 | nuoC | NADH-quinone oxidoreductase subunit C/D | -1.02791303 | 1.78E-37 | 1.47E-36 |
| FQU82_RS14060 | hemE | uroporphyrinogen decarboxylase | -2.245636617 | 2.09E-37 | 1.71E-36 |
| FQU82_RS02155 | rimP | ribosome maturation factor RimP | -1.381526696 | 3.08E-37 | 2.52E-36 |
| FQU82_RS02290 | FQU82_RS02290 | chaperone modulator CbpM | 1.474990813 | 3.32E-37 | 2.71E-36 |
| FQU82_RS09015 | FQU82_RS09015 | ATP-binding cassette domain-containing protein | 3.093431811 | 4.45E-37 | 3.62E-36 |
| FQU82_RS01065 | FQU82_RS01065 | RidA family protein | 1.606224131 | 4.91E-37 | 3.99E-36 |
| FQU82_RS10345 | antC | anthranilate 1,2-dioxygenase electron transfer component AntC | 4.656126646 | 5.12E-37 | 4.15E-36 |
| FQU82_RS03265 | FQU82_RS03265 | TIM barrel protein | 1.259428055 | 6.39E-37 | 5.17E-36 |
| FQU82_RS18400 | guaB | IMP dehydrogenase | -1.282980097 | 1.11E-36 | 8.95E-36 |
| FQU82_RS08365 | FQU82_RS08365 | enoyl-CoA hydratase | -2.321515803 | 1.18E-36 | 9.47E-36 |
| FQU82_RS18055 | FQU82_RS18055 | hypothetical protein | 4.172682916 | 1.18E-36 | 9.47E-36 |
| FQU82_RS17940 | FQU82_RS17940 | GNAT family N-acetyltransferase | 1.112649102 | 1.18E-36 | 9.47E-36 |
| FQU82_RS03055 | der | ribosome biogenesis GTPase Der | -1.190228277 | 1.73E-36 | 1.38E-35 |
| FQU82_RS05990 | FQU82_RS05990 | LysM peptidoglycan-binding domain-containing protein | -0.954549817 | 1.84E-36 | 1.47E-35 |
| FQU82_RS01680 | FQU82_RS01680 | response regulator | 0.998937375 | 2.72E-36 | 2.17E-35 |
| FQU82_RS06965 | FQU82_RS06965 | dihydroorotase | -1.530155663 | 3.24E-36 | 2.57E-35 |
| FQU82_RS04965 | betA | choline dehydrogenase | -1.207603818 | 3.81E-36 | 3.02E-35 |
| FQU82_RS18475 | murC | UDP-N-acetylmuramate--L-alanine ligase | -1.322615385 | 4.48E-36 | 3.54E-35 |
| FQU82_RS03920 | ndhC | NADH-quinone oxidoreductase subunit A | -0.981430533 | 4.88E-36 | 3.86E-35 |
| FQU82_RS11665 | FQU82_RS11665 | S4 domain-containing protein | 1.723565037 | 6.64E-36 | 5.23E-35 |
| FQU82_RS00345 | FQU82_RS00345 | hypothetical protein | -2.161878525 | 6.96E-36 | 5.47E-35 |
| FQU82_RS11475 | FQU82_RS11475 | nitroreductase family protein | 1.574350081 | 7.19E-36 | 5.64E-35 |
| FQU82_RS15965 | FQU82_RS15965 | PepSY domain-containing protein | -1.770400263 | 7.68E-36 | 6.01E-35 |
| FQU82_RS08000 | FQU82_RS08000 | GntR family transcriptional regulator | 2.124469903 | 1.10E-35 | 8.56E-35 |
| FQU82_RS10175 | FQU82_RS10175 | fumarate reductase/succinate dehydrogenase flavoprotein subunit | 2.995621295 | 1.15E-35 | 8.94E-35 |
| FQU82_RS07040 | ahpC | alkyl hydroperoxide reductase subunit C | 0.972074585 | 2.39E-35 | 1.86E-34 |
| FQU82_RS06530 | FQU82_RS06530 | pirin family protein | 3.204974129 | 3.90E-35 | 3.03E-34 |
| FQU82_RS03950 | nuoH | NADH-quinone oxidoreductase subunit NuoH | -0.891434793 | 4.74E-35 | 3.67E-34 |
| FQU82_RS08930 | FQU82_RS08930 | pyridoxal phosphate-dependent aminotransferase | 0.994298479 | 9.66E-35 | 7.46E-34 |
| FQU82_RS08635 | FQU82_RS08635 | serine acetyltransferase | 3.129682014 | 1.01E-34 | 7.82E-34 |
| FQU82_RS01265 | argS | arginine--tRNA ligase | -0.965640769 | 1.02E-34 | 7.83E-34 |
| FQU82_RS07605 | FQU82_RS07605 | universal stress protein | 0.975866924 | 1.17E-34 | 8.98E-34 |
| FQU82_RS09740 | iscA | iron-sulfur cluster assembly protein IscA | 1.371810001 | 1.36E-34 | 1.04E-33 |
| FQU82_RS12530 | prfA | peptide chain release factor 1 | -1.33628616 | 2.47E-34 | 1.88E-33 |
| FQU82_RS04250 | fabD | ACP S-malonyltransferase | -1.07871378 | 2.56E-34 | 1.95E-33 |
| FQU82_RS18270 | uvrA | excinuclease ABC subunit UvrA | 1.050921159 | 3.12E-34 | 2.38E-33 |
| FQU82_RS01695 | FQU82_RS01695 | homoserine dehydrogenase | -1.035360405 | 4.18E-34 | 3.17E-33 |
| FQU82_RS02590 | FQU82_RS02590 | AraC family transcriptional regulator | 1.093748868 | 6.77E-34 | 5.13E-33 |
| FQU82_RS04455 | FQU82_RS04455 | NAD+ synthase | -1.011955646 | 9.69E-34 | 7.33E-33 |
| Novel00043 | - | PF00562:RNA polymerase Rpb2, domain 6|PF04560:RNA polymerase Rpb2, domain 7|PF04565:RNA polymerase Rpb2, domain 3|PF04563:RNA polymerase beta subunit|PF04561:RNA polymerase Rpb2, domain 2|PF10385:RNA polymerase beta subunit external 1 domain|PF02357:Transcription termination factor nusG|PF03143:Elongation factor Tu C-terminal domain|PF00009:Elongation factor Tu GTP binding domain|PF03144:Elongation factor Tu domain 2|PF00687:Ribosomal protein L1p/L10e family|PF00584:SecE/Sec61-gamma subunits of protein translocation complex|PF00466:Ribosomal protein L10|PF04997:RNA polymerase Rpb1, domain 1|PF00623:RNA polymerase Rpb1, domain 2|PF04983:RNA polymerase Rpb1, domain 3|PF05000:RNA polymerase Rpb1, domain 4|PF04998:RNA polymerase Rpb1, domain 5 | -1.26737889 | 1.20E-33 | 9.04E-33 |
| FQU82_RS13975 | glnA | type I glutamate--ammonia ligase | -1.061424164 | 1.69E-33 | 1.27E-32 |
| FQU82_RS12730 | FQU82_RS12730 | hypothetical protein | 1.418953603 | 2.15E-33 | 1.61E-32 |
| FQU82_RS16220 | FQU82_RS16220 | glucose/quinate/shikimate family membrane-bound PQQ-dependent dehydrogenase | -0.813474947 | 3.52E-33 | 2.64E-32 |
| FQU82_RS08960 | glnD | [protein-PII] uridylyltransferase | 1.125967831 | 8.58E-33 | 6.41E-32 |
| FQU82_RS07510 | cysS | cysteine--tRNA ligase | -1.12442274 | 9.91E-33 | 7.40E-32 |
| FQU82_RS03070 | grxC | glutaredoxin 3 | 1.148094312 | 1.43E-32 | 1.06E-31 |
| FQU82_RS02645 | FQU82_RS02645 | ATP-binding cassette domain-containing protein | -1.289655245 | 1.43E-32 | 1.06E-31 |
| FQU82_RS14915 | tolB | Tol-Pal system beta propeller repeat protein TolB | -1.290698624 | 1.74E-32 | 1.29E-31 |
| FQU82_RS08920 | FQU82_RS08920 | type II asparaginase | -1.080888952 | 2.36E-32 | 1.75E-31 |
| FQU82_RS00200 | rpmH | 50S ribosomal protein L34 | -2.259063691 | 2.86E-32 | 2.11E-31 |
| FQU82_RS09815 | FQU82_RS09815 | IucA/IucC family protein | -2.5299218 | 4.14E-32 | 3.05E-31 |
| FQU82_RS18735 | ribBA | bifunctional 3,4-dihydroxy-2-butanone-4-phosphate synthase/GTP cyclohydrolase II | -0.919618758 | 5.47E-32 | 4.02E-31 |
| FQU82_RS04300 | pth | aminoacyl-tRNA hydrolase | -1.34282635 | 5.50E-32 | 4.04E-31 |
| FQU82_RS02035 | uvrC | excinuclease ABC subunit UvrC | 1.455883371 | 6.41E-32 | 4.69E-31 |
| FQU82_RS11465 | FQU82_RS11465 | cyd operon YbgE family protein | -1.39475469 | 6.88E-32 | 5.03E-31 |
| FQU82_RS13090 | FQU82_RS13090 | GatB/YqeY domain-containing protein | -0.894281949 | 7.37E-32 | 5.38E-31 |
| FQU82_RS15520 | gltA | citrate synthase | -1.075634792 | 7.95E-32 | 5.78E-31 |
| FQU82_RS14430 | hemL | glutamate-1-semialdehyde 2,1-aminomutase | -1.039239477 | 1.04E-31 | 7.57E-31 |
| FQU82_RS17435 | FQU82_RS17435 | aspartate aminotransferase family protein | -2.067691214 | 1.08E-31 | 7.85E-31 |
| FQU82_RS15790 | hchA | protein deglycase HchA | -1.327367566 | 1.91E-31 | 1.38E-30 |
| FQU82_RS03360 | thrS | threonine--tRNA ligase | -0.868686323 | 2.07E-31 | 1.49E-30 |
| FQU82_RS18545 | FQU82_RS18545 | HPF/RaiA family ribosome-associated protein | 0.83170872 | 2.47E-31 | 1.78E-30 |
| FQU82_RS17415 | astE | succinylglutamate desuccinylase | -1.377865164 | 3.96E-31 | 2.85E-30 |
| FQU82_RS11490 | FQU82_RS11490 | hypothetical protein | 1.238149485 | 4.92E-31 | 3.53E-30 |
| FQU82_RS14445 | FQU82_RS14445 | CYTH and CHAD domain-containing protein | 1.339526664 | 8.69E-31 | 6.22E-30 |
| FQU82_RS13080 | tsaD | tRNA (adenosine(37)-N6)-threonylcarbamoyltransferase complex transferase subunit TsaD | -1.772232056 | 1.08E-30 | 7.69E-30 |
| FQU82_RS18785 | hutG | formimidoylglutamase | 1.256336008 | 1.55E-30 | 1.11E-29 |
| FQU82_RS15855 | rng | ribonuclease G | -1.1446491 | 1.71E-30 | 1.22E-29 |
| FQU82_RS02470 | FQU82_RS02470 | long-chain-acyl-CoA synthetase | -1.086295222 | 1.96E-30 | 1.39E-29 |
| FQU82_RS02190 | FQU82_RS02190 | monovalent cation/H+ antiporter subunit D | -1.054230917 | 2.19E-30 | 1.55E-29 |
| FQU82_RS04075 | FQU82_RS04075 | 5'-methylthioadenosine/S-adenosylhomocysteine nucleosidase | -2.995043589 | 2.67E-30 | 1.89E-29 |
| FQU82_RS12400 | FQU82_RS12400 | MFS transporter | -2.411912371 | 2.79E-30 | 1.97E-29 |
| FQU82_RS10245 | FQU82_RS10245 | thiolase family protein | 1.261666529 | 3.06E-30 | 2.16E-29 |
| FQU82_RS09875 | FQU82_RS09875 | HAD family hydrolase | 1.30457186 | 3.84E-30 | 2.70E-29 |
| Novel00148 | - | PF20169:Family of unknown function (DUF6537)|PF01558:Pyruvate ferredoxin/flavodoxin oxidoreductase | 3.138371753 | 3.99E-30 | 2.80E-29 |
| FQU82_RS08355 | FQU82_RS08355 | 3-hydroxyacyl-CoA dehydrogenase | -2.622012571 | 4.33E-30 | 3.03E-29 |
| FQU82_RS04970 | betB | betaine-aldehyde dehydrogenase | -1.155091231 | 6.18E-30 | 4.32E-29 |
| FQU82_RS04715 | FQU82_RS04715 | tyrosine-type recombinase/integrase | 1.277064863 | 8.26E-30 | 5.76E-29 |
| FQU82_RS18620 | FQU82_RS18620 | type VI secretion system Vgr family protein | -2.724450917 | 9.68E-30 | 6.74E-29 |
| FQU82_RS11355 | FQU82_RS11355 | 3-deoxy-7-phosphoheptulonate synthase | 0.899790757 | 1.70E-29 | 1.18E-28 |
| FQU82_RS05880 | FQU82_RS05880 | hypothetical protein | 1.821515859 | 1.95E-29 | 1.36E-28 |
| FQU82_RS18415 | FQU82_RS18415 | TonB-dependent siderophore receptor | -1.529362805 | 2.19E-29 | 1.52E-28 |
| FQU82_RS04995 | FQU82_RS04995 | acyl-CoA thioesterase | 1.336495086 | 4.47E-29 | 3.09E-28 |
| FQU82_RS11850 | FQU82_RS11850 | winged helix-turn-helix transcriptional regulator | 1.358113163 | 5.23E-29 | 3.60E-28 |
| FQU82_RS17035 | FQU82_RS17035 | NAD(P)/FAD-dependent oxidoreductase | 0.82444982 | 5.86E-29 | 4.04E-28 |
| Novel00164 | - | PF01592:NifU-like N terminal domain|PF02082:Iron-dependent Transcriptional regulator|PF00266:Aminotransferase class-V|PF01521:Iron-sulphur cluster biosynthesis | 2.081401192 | 9.21E-29 | 6.33E-28 |
| FQU82_RS04585 | dut | dUTP diphosphatase | -2.846931609 | 1.06E-28 | 7.27E-28 |
| FQU82_RS05190 | metH | methionine synthase | -1.085063209 | 2.13E-28 | 1.46E-27 |
| FQU82_RS05680 | FQU82_RS05680 | hypothetical protein | 1.253538139 | 2.38E-28 | 1.63E-27 |
| FQU82_RS03040 | hisS | histidine--tRNA ligase | -1.224133212 | 2.43E-28 | 1.66E-27 |
| FQU82_RS11690 | lpxA | acyl-ACP--UDP-N-acetylglucosamine O-acyltransferase | -1.021563564 | 2.49E-28 | 1.70E-27 |
| FQU82_RS09565 | pyrF | orotidine-5'-phosphate decarboxylase | -1.965938106 | 6.03E-28 | 4.10E-27 |
| FQU82_RS17400 | FQU82_RS17400 | S8 family serine peptidase | 2.105882189 | 7.64E-28 | 5.18E-27 |
| Novel00010 | - | - | 4.173182836 | 9.00E-28 | 6.09E-27 |
| FQU82_RS15530 | FQU82_RS15530 | rhodanese-related sulfurtransferase | -1.583265474 | 9.54E-28 | 6.44E-27 |
| FQU82_RS18295 | FQU82_RS18295 | cation acetate symporter | -1.151378377 | 9.79E-28 | 6.60E-27 |
| FQU82_RS15485 | FQU82_RS15485 | 2-oxoglutarate dehydrogenase E1 component | -0.865644813 | 1.24E-27 | 8.36E-27 |
| FQU82_RS02980 | FQU82_RS02980 | alpha/beta hydrolase | 1.363621006 | 1.55E-27 | 1.04E-26 |
| FQU82_RS10255 | FQU82_RS10255 | 3-oxoacid CoA-transferase subunit B | 1.443324305 | 1.65E-27 | 1.11E-26 |
| FQU82_RS04215 | rluB | 23S rRNA pseudouridine(2605) synthase RluB | -0.89813208 | 4.51E-27 | 3.02E-26 |
| FQU82_RS11570 | FQU82_RS11570 | fructose-specific PTS transporter subunit EIIC | -1.301820612 | 7.00E-27 | 4.67E-26 |
| FQU82_RS05500 | FQU82_RS05500 | OmpA family protein | -2.679654223 | 7.19E-27 | 4.79E-26 |
| FQU82_RS15635 | FQU82_RS15635 | phosphatase PAP2 family protein | -0.963617114 | 1.23E-26 | 8.20E-26 |
| FQU82_RS01870 | FQU82_RS01870 | tRNA-Tyr | -1.29680647 | 2.67E-26 | 1.77E-25 |
| Novel00342 | - | PF09917:Uncharacterized protein conserved in bacteria (DUF2147) | 1.771166587 | 3.00E-26 | 1.99E-25 |
| FQU82_RS12415 | FQU82_RS12415 | bifunctional aconitate hydratase 2/2-methylisocitrate dehydratase | -0.835221838 | 3.05E-26 | 2.02E-25 |
| FQU82_RS08375 | FQU82_RS08375 | MFS transporter | -3.318739185 | 3.07E-26 | 2.03E-25 |
| FQU82_RS03955 | nuoI | NADH-quinone oxidoreductase subunit NuoI | -0.805058946 | 3.85E-26 | 2.54E-25 |
| Novel00356 | - | PF00903:Glyoxalase/Bleomycin resistance protein/Dioxygenase superfamily|PF14696:Hydroxyphenylpyruvate dioxygenase, HPPD, N-terminal | 3.356731845 | 7.00E-26 | 4.60E-25 |
| FQU82_RS09990 | FQU82_RS09990 | HIT family protein | 1.024417887 | 7.12E-26 | 4.67E-25 |
| FQU82_RS07775 | FQU82_RS07775 | SRPBCC family protein | 1.607073408 | 9.24E-26 | 6.06E-25 |
| FQU82_RS15515 | sdhC | succinate dehydrogenase, cytochrome b556 subunit | -0.864713727 | 1.23E-25 | 8.06E-25 |
| FQU82_RS03250 | FQU82_RS03250 | NAD(P)(+) transhydrogenase (Re/Si-specific) subunit beta | -0.851803678 | 1.30E-25 | 8.47E-25 |
| FQU82_RS16525 | queA | tRNA preQ1(34) S-adenosylmethionine ribosyltransferase-isomerase QueA | -1.818771197 | 1.50E-25 | 9.80E-25 |
| FQU82_RS10730 | FQU82_RS10730 | phosphoribosyltransferase family protein | -1.549150583 | 1.54E-25 | 1.00E-24 |
| FQU82_RS01765 | FQU82_RS01765 | TetR/AcrR family transcriptional regulator | 4.142918896 | 1.70E-25 | 1.10E-24 |
| FQU82_RS00285 | mpl | UDP-N-acetylmuramate:L-alanyl-gamma-D-glutamyl-meso-diaminopimelate ligase | -1.126203434 | 1.82E-25 | 1.18E-24 |
| FQU82_RS12580 | pgaA | poly-beta-1,6 N-acetyl-D-glucosamine exporter porin PgaA | 3.415533353 | 1.84E-25 | 1.19E-24 |
| FQU82_RS00880 | FQU82_RS00880 | nucleotide sugar dehydrogenase | -1.267332367 | 2.65E-25 | 1.71E-24 |
| FQU82_RS15795 | FQU82_RS15795 | NAD(P)H-quinone oxidoreductase | -1.107263767 | 3.08E-25 | 1.98E-24 |
| FQU82_RS02965 | FQU82_RS02965 | gluconokinase | -1.279634208 | 3.32E-25 | 2.14E-24 |
| FQU82_RS14415 | rapA | RNA polymerase-associated protein RapA | -0.781559105 | 3.64E-25 | 2.34E-24 |
| FQU82_RS17765 | murF | UDP-N-acetylmuramoyl-tripeptide--D-alanyl-D-alanine ligase | -1.249878977 | 4.72E-25 | 3.02E-24 |
| FQU82_RS09175 | FQU82_RS09175 | cytosine permease | 1.751487483 | 5.50E-25 | 3.52E-24 |
| FQU82_RS14005 | trpD | anthranilate phosphoribosyltransferase | -1.171807936 | 6.27E-25 | 4.01E-24 |
| FQU82_RS03960 | nuoJ | NADH-quinone oxidoreductase subunit J | -0.897124547 | 6.72E-25 | 4.29E-24 |
| FQU82_RS15735 | FQU82_RS15735 | DcaP family trimeric outer membrane transporter | -0.95025161 | 8.98E-25 | 5.71E-24 |
| FQU82_RS10295 | FQU82_RS10295 | GntP family permease | 1.437838333 | 9.83E-25 | 6.24E-24 |
| FQU82_RS11880 | FQU82_RS11880 | molybdopterin-dependent oxidoreductase | 2.363657459 | 1.12E-24 | 7.13E-24 |
| FQU82_RS04555 | minD | septum site-determining protein MinD | -0.815927363 | 1.16E-24 | 7.37E-24 |
| FQU82_RS02765 | FQU82_RS02765 | dihydrofolate reductase | -1.247168079 | 1.48E-24 | 9.33E-24 |
| FQU82_RS11720 | ispC | 1-deoxy-D-xylulose-5-phosphate reductoisomerase | -1.14386697 | 1.63E-24 | 1.03E-23 |
| FQU82_RS18035 | FQU82_RS18035 | potassium transporter Kup | -1.11992482 | 1.71E-24 | 1.08E-23 |
| FQU82_RS19035 | ppc | phosphoenolpyruvate carboxylase | 0.722939692 | 2.10E-24 | 1.32E-23 |
| FQU82_RS13860 | FQU82_RS13860 | MFS transporter | 1.499477168 | 2.22E-24 | 1.39E-23 |
| FQU82_RS03050 | bamB | outer membrane protein assembly factor BamB | -0.833841533 | 2.30E-24 | 1.44E-23 |
| FQU82_RS11560 | ptsP | phosphoenolpyruvate--protein phosphotransferase | -1.072657655 | 2.66E-24 | 1.66E-23 |
| FQU82_RS16505 | FQU82_RS16505 | branched-chain amino acid transaminase | -0.733271275 | 3.65E-24 | 2.28E-23 |
| FQU82_RS14265 | FQU82_RS14265 | peptidoglycan DD-metalloendopeptidase family protein | -1.239099913 | 4.51E-24 | 2.81E-23 |
| FQU82_RS15375 | rlmE | 23S rRNA (uridine(2552)-2'-O)-methyltransferase RlmE | 0.744492666 | 4.97E-24 | 3.09E-23 |
| FQU82_RS09430 | FQU82_RS09430 | phosphotransferase | -1.495623382 | 5.19E-24 | 3.23E-23 |
| FQU82_RS15005 | lolD | lipoprotein-releasing ABC transporter ATP-binding protein LolD | -1.325855552 | 6.05E-24 | 3.75E-23 |
| FQU82_RS11970 | FQU82_RS11970 | FMN-binding glutamate synthase family protein | -1.630363558 | 6.14E-24 | 3.80E-23 |
| FQU82_RS15510 | sdhD | succinate dehydrogenase, hydrophobic membrane anchor protein | -0.964094807 | 7.62E-24 | 4.71E-23 |
| FQU82_RS09820 | FQU82_RS09820 | SidA/IucD/PvdA family monooxygenase | -2.772377462 | 8.23E-24 | 5.08E-23 |
| FQU82_RS11935 | accB | acetyl-CoA carboxylase biotin carboxyl carrier protein | -1.554644434 | 1.07E-23 | 6.56E-23 |
| FQU82_RS07985 | FQU82_RS07985 | I78 family peptidase inhibitor | -1.060974246 | 1.12E-23 | 6.90E-23 |
| FQU82_RS13225 | FQU82_RS13225 | bifunctional prephenate dehydrogenase/3-phosphoshikimate 1-carboxyvinyltransferase | -0.924867452 | 1.25E-23 | 7.67E-23 |
| FQU82_RS15280 | FQU82_RS15280 | co-chaperone GroES | -1.224105258 | 1.27E-23 | 7.81E-23 |
| FQU82_RS03220 | dusA | tRNA dihydrouridine(20/20a) synthase DusA | -1.959038268 | 1.36E-23 | 8.31E-23 |
| FQU82_RS01970 | htpG | molecular chaperone HtpG | 1.070438722 | 1.44E-23 | 8.77E-23 |
| FQU82_RS04685 | hemB | porphobilinogen synthase | 0.848112529 | 1.48E-23 | 8.99E-23 |
| FQU82_RS11565 | pfkB | 1-phosphofructokinase | -1.412327605 | 1.48E-23 | 9.03E-23 |
| FQU82_RS06580 | csrA | carbon storage regulator CsrA | 1.063431396 | 1.54E-23 | 9.34E-23 |
| Novel00058 | - | PF00141:Peroxidase | 2.301048607 | 1.72E-23 | 1.04E-22 |
| FQU82_RS03410 | pheS | phenylalanine--tRNA ligase subunit alpha | -0.973101235 | 2.15E-23 | 1.30E-22 |
| FQU82_RS13190 | FQU82_RS13190 | thiazole synthase | -1.115741631 | 2.15E-23 | 1.30E-22 |
| FQU82_RS15305 | glpD | glycerol-3-phosphate dehydrogenase | 1.730030227 | 2.28E-23 | 1.38E-22 |
| FQU82_RS02295 | FQU82_RS02295 | DnaJ C-terminal domain-containing protein | 1.205158524 | 2.58E-23 | 1.56E-22 |
| FQU82_RS06020 | FQU82_RS06020 | TonB-dependent receptor | -2.005976575 | 2.63E-23 | 1.58E-22 |
| FQU82_RS18440 | FQU82_RS18440 | M23 family metallopeptidase | -1.289458426 | 3.07E-23 | 1.84E-22 |
| FQU82_RS01725 | murD | UDP-N-acetylmuramoyl-L-alanine--D-glutamate ligase | -1.311828638 | 3.47E-23 | 2.08E-22 |
| FQU82_RS14640 | FQU82_RS14640 | hypothetical protein | 1.256436361 | 3.92E-23 | 2.34E-22 |
| FQU82_RS11695 | fabZ | 3-hydroxyacyl-ACP dehydratase FabZ | -1.16089737 | 5.30E-23 | 3.16E-22 |
| FQU82_RS17625 | gmk | guanylate kinase | -1.096286694 | 5.38E-23 | 3.21E-22 |
| FQU82_RS13795 | rodA | rod shape-determining protein RodA | -2.431442816 | 5.65E-23 | 3.36E-22 |
| FQU82_RS11795 | FQU82_RS11795 | hypothetical protein | -3.148225088 | 6.83E-23 | 4.06E-22 |
| FQU82_RS04700 | FQU82_RS04700 | DHA2 family efflux MFS transporter permease subunit | 1.057910547 | 6.89E-23 | 4.08E-22 |
| FQU82_RS08805 | tauA | taurine ABC transporter substrate-binding protein | 0.70357682 | 7.08E-23 | 4.19E-22 |
| Novel00024 | - | PF01042:Endoribonuclease L-PSP|PF00842:Alanine racemase, C-terminal domain|PF01168:Alanine racemase, N-terminal domain|PF01266:FAD dependent oxidoreductase|PF00324:Amino acid permease | 2.072371229 | 1.08E-22 | 6.40E-22 |
| FQU82_RS05380 | FQU82_RS05380 | copper resistance protein NlpE | -1.209943351 | 1.49E-22 | 8.81E-22 |
| FQU82_RS12170 | FQU82_RS12170 | mechanosensitive ion channel | -1.424633217 | 1.63E-22 | 9.58E-22 |
| Novel00235 | - | PF12344:Ultra-violet resistance protein B|PF02151:UvrB/uvrC motif|PF04851:Type III restriction enzyme, res subunit|PF17757:UvrB interaction domain|PF00271:Helicase conserved C-terminal domain|PF08212:Lipocalin-like domain | 2.521099826 | 2.05E-22 | 1.21E-21 |
| FQU82_RS00795 | FQU82_RS00795 | FKBP-type peptidyl-prolyl cis-trans isomerase | -0.860071945 | 2.68E-22 | 1.57E-21 |
| Novel00230 | - | PF03466:LysR substrate binding domain|PF00126:Bacterial regulatory helix-turn-helix protein, lysR family|PF00375:Sodium:dicarboxylate symporter family | 2.144567702 | 3.24E-22 | 1.90E-21 |
| FQU82_RS10260 | FQU82_RS10260 | CoA transferase subunit A | 1.515065118 | 3.53E-22 | 2.07E-21 |
| FQU82_RS01490 | FQU82_RS01490 | DUF2726 domain-containing protein | 1.627658226 | 3.64E-22 | 2.13E-21 |
| FQU82_RS03415 | pheT | phenylalanine--tRNA ligase subunit beta | -0.779701173 | 6.02E-22 | 3.51E-21 |
| Novel00307 | - | PF01245:Ribosomal protein L19|PF01746:tRNA (Guanine-1)-methyltransferase|PF01782:RimM N-terminal domain|PF00886:Ribosomal protein S16 | -1.649780089 | 6.16E-22 | 3.59E-21 |
| FQU82_RS08360 | FQU82_RS08360 | SDR family oxidoreductase | -2.71731136 | 6.67E-22 | 3.88E-21 |
| FQU82_RS18500 | pyrE | orotate phosphoribosyltransferase | -1.522910389 | 7.29E-22 | 4.23E-21 |
| FQU82_RS12695 | fis | DNA-binding transcriptional regulator Fis | -1.427197606 | 8.83E-22 | 5.12E-21 |
| FQU82_RS09370 | FQU82_RS09370 | YoaK family protein | -1.935781373 | 9.12E-22 | 5.27E-21 |
| FQU82_RS09530 | FQU82_RS09530 | enoyl-CoA hydratase/isomerase family protein | -0.874953819 | 9.32E-22 | 5.38E-21 |
| FQU82_RS04090 | dcd | dCTP deaminase | -1.000734549 | 9.44E-22 | 5.44E-21 |
| FQU82_RS01715 | FQU82_RS01715 | ferrous iron transporter | -0.740688834 | 9.99E-22 | 5.75E-21 |
| FQU82_RS16185 | FQU82_RS16185 | OmpA family protein | -0.590718876 | 1.10E-21 | 6.32E-21 |
| FQU82_RS13875 | FQU82_RS13875 | acyl-CoA dehydrogenase | 0.652273139 | 1.11E-21 | 6.34E-21 |
| FQU82_RS09910 | FQU82_RS09910 | 3-deoxy-7-phosphoheptulonate synthase | -0.856482266 | 1.17E-21 | 6.67E-21 |
| FQU82_RS15545 | rpoD | RNA polymerase sigma factor RpoD | 0.608084929 | 1.24E-21 | 7.08E-21 |
| Novel00072 | - | PF05681:Fumarate hydratase (Fumerase)|PF05683:Fumarase C-terminus | -1.854239042 | 1.37E-21 | 7.84E-21 |
| FQU82_RS15230 | FQU82_RS15230 | SCP2 sterol-binding domain-containing protei | -0.907831255 | 2.35E-21 | 1.34E-20 |
| FQU82_RS12175 | FQU82_RS12175 | TonB-dependent receptor | -3.172751899 | 2.92E-21 | 1.66E-20 |
| FQU82_RS08540 | FQU82_RS08540 | hypothetical protein | 1.279778966 | 3.23E-21 | 1.83E-20 |
| FQU82_RS17445 | FQU82_RS17445 | amino acid permease | -0.972671484 | 3.47E-21 | 1.97E-20 |
| Novel00179 | - | PF10431:C-terminal, D2-small domain, of ClpB protein|PF02861:Clp amino terminal domain, pathogenicity island component|PF07724:AAA domain (Cdc48 subfamily)|PF17871:AAA lid domain|PF00004:ATPase family associated with various cellular activities (AAA) | 1.773800575 | 3.61E-21 | 2.05E-20 |
| Novel00068 | - | PF00682:HMGL-like|PF08502:LeuA allosteric (dimerisation) domain | 2.114466641 | 3.66E-21 | 2.07E-20 |
| FQU82_RS03095 | FQU82_RS03095 | enoyl-ACP reductase | -1.202558117 | 6.20E-21 | 3.50E-20 |
| FQU82_RS02080 | ruvX | Holliday junction resolvase RuvX | 0.847926785 | 6.42E-21 | 3.62E-20 |
| FQU82_RS14705 | pdxJ | pyridoxine 5'-phosphate synthase | -1.403569392 | 7.85E-21 | 4.42E-20 |
| FQU82_RS15285 | blp1 | biofilm-associated Ig-like repeat protein Blp1 | -0.95588446 | 8.89E-21 | 4.99E-20 |
| Novel00331 | - | PF00296:Luciferase-like monooxygenase | 3.059235207 | 1.02E-20 | 5.72E-20 |
| FQU82_RS00300 | FQU82_RS00300 | 5-(carboxyamino)imidazole ribonucleotide synthase | -1.019483525 | 1.57E-20 | 8.82E-20 |
| FQU82_RS14520 | FQU82_RS14520 | D-alanyl-D-alanine carboxypeptidase PBP6B | -1.164384485 | 1.83E-20 | 1.03E-19 |
| FQU82_RS14530 | FQU82_RS14530 | peptidylprolyl isomerase | -0.736446021 | 2.02E-20 | 1.13E-19 |
| FQU82_RS12575 | pgaB | poly-beta-1,6-N-acetyl-D-glucosamine N-deacetylase PgaB | 2.949180238 | 2.84E-20 | 1.59E-19 |
| FQU82_RS00410 | ileS | isoleucine--tRNA ligase | -0.666856925 | 2.90E-20 | 1.61E-19 |
| FQU82_RS14300 | mnmA | tRNA 2-thiouridine(34) synthase MnmA | -1.069901795 | 2.98E-20 | 1.66E-19 |
| FQU82_RS00785 | murJ | murein biosynthesis integral membrane protein MurJ | -1.153455305 | 3.03E-20 | 1.68E-19 |
| FQU82_RS03965 | nuoK | NADH-quinone oxidoreductase subunit NuoK | -0.959648972 | 3.47E-20 | 1.93E-19 |
| FQU82_RS15750 | FQU82_RS15750 | patatin-like phospholipase family protein | -1.664665968 | 3.70E-20 | 2.05E-19 |
| FQU82_RS03465 | FQU82_RS03465 | YggT family protein | -1.281939282 | 3.89E-20 | 2.15E-19 |
| FQU82_RS14065 | FQU82_RS14065 | L,D-transpeptidase | -0.710958048 | 4.73E-20 | 2.61E-19 |
| FQU82_RS00515 | rph | ribonuclease PH | -1.086428587 | 5.12E-20 | 2.82E-19 |
| FQU82_RS15500 | FQU82_RS15500 | succinate dehydrogenase iron-sulfur subunit | -0.655683968 | 5.13E-20 | 2.82E-19 |
| FQU82_RS14950 | FQU82_RS14950 | DUF3106 domain-containing protein | 1.907507984 | 5.31E-20 | 2.92E-19 |
| FQU82_RS01400 | FQU82_RS01400 | DUF2946 family protein | -1.170554413 | 7.65E-20 | 4.19E-19 |
| FQU82_RS18660 | FQU82_RS18660 | SDR family NAD(P)-dependent oxidoreductase | 0.979761253 | 8.25E-20 | 4.52E-19 |
| FQU82_RS08640 | FQU82_RS08640 | rhodanese-like domain-containing protein | 2.45836764 | 9.53E-20 | 5.21E-19 |
| Novel00262 | - | PF01016:Ribosomal L27 protein|PF00829:Ribosomal prokaryotic L21 protein | -1.08191544 | 9.85E-20 | 5.38E-19 |
| FQU82_RS09765 | FQU82_RS09765 | phasin family protein | 1.844236974 | 1.00E-19 | 5.45E-19 |
| FQU82_RS15625 | FQU82_RS15625 | polyprenyl synthetase family protein | -1.271714106 | 1.01E-19 | 5.48E-19 |
| FQU82_RS03075 | FQU82_RS03075 | rhodanese-like domain-containing protein | 0.819330501 | 1.29E-19 | 6.99E-19 |
| FQU82_RS04505 | FQU82_RS04505 | metal-dependent hydrolase | 2.140649496 | 1.34E-19 | 7.26E-19 |
| FQU82_RS02205 | FQU82_RS02205 | AAA family ATPase | 0.937301176 | 1.59E-19 | 8.61E-19 |
| FQU82_RS15205 | FQU82_RS15205 | non-ribosomal peptide synthetase | -0.863519532 | 1.62E-19 | 8.78E-19 |
| FQU82_RS02680 | FQU82_RS02680 | HdeD family acid-resistance protein | 1.386602472 | 1.64E-19 | 8.86E-19 |
| FQU82_RS09425 | FQU82_RS09425 | LPS-assembly protein LptD | -1.047124872 | 1.70E-19 | 9.19E-19 |
| FQU82_RS09995 | FQU82_RS09995 | YARHG domain-containing protein | 0.978789858 | 1.88E-19 | 1.01E-18 |
| FQU82_RS09945 | FQU82_RS09945 | CerR family C-terminal domain-containing protein | 2.387630289 | 2.11E-19 | 1.13E-18 |
| FQU82_RS14365 | FQU82_RS14365 | aldehyde dehydrogenase family protein | 1.041236659 | 2.94E-19 | 1.58E-18 |
| FQU82_RS16075 | FQU82_RS16075 | proline--tRNA ligase | -0.701122101 | 2.97E-19 | 1.59E-18 |
| FQU82_RS14900 | tolQ | protein TolQ | -1.582969385 | 3.82E-19 | 2.05E-18 |
| FQU82_RS11430 | FQU82_RS11430 | adenosine kinase | -0.900863438 | 4.34E-19 | 2.32E-18 |
| FQU82_RS16415 | FQU82_RS16415 | glutathione S-transferase family protein | 3.873779567 | 4.54E-19 | 2.42E-18 |
| FQU82_RS04065 | FQU82_RS04065 | hypothetical protein | 0.817793421 | 4.76E-19 | 2.53E-18 |
| FQU82_RS00870 | FQU82_RS00870 | sugar transferase | -1.279229577 | 6.82E-19 | 3.62E-18 |
| FQU82_RS12140 | FQU82_RS12140 | TonB-dependent siderophore receptor | -3.142820164 | 7.57E-19 | 4.02E-18 |
| FQU82_RS02970 | FQU82_RS02970 | NADP-dependent glyceraldehyde-3-phosphate dehydrogenase | -0.879208067 | 8.44E-19 | 4.48E-18 |
| FQU82_RS12595 | FQU82_RS12595 | RDD family protein | -1.272392907 | 1.15E-18 | 6.10E-18 |
| FQU82_RS15880 | gatC | Asp-tRNA(Asn)/Glu-tRNA(Gln) amidotransferase subunit GatC | -1.63978781 | 1.25E-18 | 6.60E-18 |
| FQU82_RS05375 | FQU82_RS05375 | isocitrate lyase | 0.722593051 | 1.33E-18 | 7.00E-18 |
| FQU82_RS09465 | msbA | lipid A export permease/ATP-binding protein MsbA | -0.79067516 | 1.83E-18 | 9.62E-18 |
| FQU82_RS02920 | clpX | ATP-dependent Clp protease ATP-binding subunit ClpX | 0.572525858 | 2.18E-18 | 1.14E-17 |
| FQU82_RS14205 | FQU82_RS14205 | YbdD/YjiX family protein | 1.373896726 | 2.52E-18 | 1.33E-17 |
| FQU82_RS18645 | FQU82_RS18645 | DUF445 domain-containing protein | 0.860116971 | 3.42E-18 | 1.79E-17 |
| FQU82_RS14510 | FQU82_RS14510 | NADP-dependent isocitrate dehydrogenase | -0.658750645 | 3.92E-18 | 2.05E-17 |
| FQU82_RS07720 | FQU82_RS07720 | divalent metal cation transporter | -1.277894349 | 4.41E-18 | 2.31E-17 |
| FQU82_RS17355 | FQU82_RS17355 | putative DNA modification/repair radical SAM protein | 1.904653484 | 4.92E-18 | 2.57E-17 |
| FQU82_RS09010 | FQU82_RS09010 | methionine ABC transporter permease | 2.738761447 | 5.02E-18 | 2.62E-17 |
| FQU82_RS09265 | FQU82_RS09265 | FxsA family protein | 0.889713562 | 5.43E-18 | 2.83E-17 |
| FQU82_RS10350 | antB | anthranilate 1,2-dioxygenase small subunit | 5.864451616 | 5.46E-18 | 2.84E-17 |
| Novel00134 | - | PF20979:Arginosuccinate synthase C-terminal domain|PF00764:Arginosuccinate synthase N-terminal HUP domain | -1.852933731 | 5.63E-18 | 2.92E-17 |
| FQU82_RS07630 | mutS | DNA mismatch repair protein MutS | -0.984274741 | 6.05E-18 | 3.14E-17 |
| FQU82_RS00885 | pgi | glucose-6-phosphate isomerase | -1.141797372 | 6.11E-18 | 3.16E-17 |
| FQU82_RS17790 | FQU82_RS17790 | sulfate ABC transporter substrate-binding protein | 0.937001972 | 6.20E-18 | 3.20E-17 |
| FQU82_RS07580 | FQU82_RS07580 | TolC family protein | -1.705821691 | 6.84E-18 | 3.53E-17 |
| FQU82_RS13790 | FQU82_RS13790 | hypothetical protein | -1.00307764 | 7.22E-18 | 3.72E-17 |
| FQU82_RS17660 | FQU82_RS17660 | O-antigen ligase family protein | -1.753404094 | 7.73E-18 | 3.98E-17 |
| FQU82_RS16210 | FQU82_RS16210 | DUF934 domain-containing protein | -1.022629135 | 8.01E-18 | 4.12E-17 |
| FQU82_RS11000 | FQU82_RS11000 | NADH:flavin oxidoreductase/NADH oxidase family protein | 2.27302122 | 8.32E-18 | 4.27E-17 |
| FQU82_RS02660 | FQU82_RS02660 | winged helix-turn-helix transcriptional regulator | 1.746272087 | 9.11E-18 | 4.67E-17 |
| FQU82_RS13065 | FQU82_RS13065 | DUF3336 domain-containing protein | 1.102460651 | 9.25E-18 | 4.74E-17 |
| FQU82_RS09785 | FQU82_RS09785 | alkane 1-monooxygenase | 0.709365844 | 9.32E-18 | 4.76E-17 |
| FQU82_RS05015 | FQU82_RS05015 | YaeQ family protein | -3.480416541 | 1.16E-17 | 5.93E-17 |
| FQU82_RS15030 | serC | 3-phosphoserine/phosphohydroxythreonine transaminase | -1.012614202 | 1.53E-17 | 7.78E-17 |
| FQU82_RS02005 | FQU82_RS02005 | hypothetical protein | 1.725596685 | 1.96E-17 | 9.99E-17 |
| FQU82_RS08310 | FQU82_RS08310 | MFS transporter | -2.758536731 | 2.02E-17 | 1.03E-16 |
| FQU82_RS02140 | secG | preprotein translocase subunit SecG | -1.007764455 | 2.25E-17 | 1.14E-16 |
| FQU82_RS13145 | FQU82_RS13145 | NAD(P)H-dependent glycerol-3-phosphate dehydrogenase | -1.10201015 | 2.51E-17 | 1.27E-16 |
| FQU82_RS16565 | coaBC | bifunctional phosphopantothenoylcysteine decarboxylase/phosphopantothenate--cysteine ligase CoaBC | -0.947805588 | 2.81E-17 | 1.42E-16 |
| FQU82_RS02260 | FQU82_RS02260 | UvrD-helicase domain-containing protein | 1.022719444 | 3.03E-17 | 1.53E-16 |
| FQU82_RS00965 | FQU82_RS00965 | DUF4126 domain-containing protein | 1.10532186 | 3.06E-17 | 1.54E-16 |
| FQU82_RS02740 | FQU82_RS02740 | energy transducer TonB | -1.259768453 | 3.30E-17 | 1.66E-16 |
| FQU82_RS12275 | FQU82_RS12275 | helix-turn-helix domain-containing protein | 1.356927656 | 3.86E-17 | 1.94E-16 |
| FQU82_RS14505 | FQU82_RS14505 | rRNA large subunit pseudouridine synthase E | -1.059237414 | 4.01E-17 | 2.01E-16 |
| FQU82_RS08350 | FQU82_RS08350 | 3-oxoadipyl-CoA thiolase | -2.241329401 | 4.50E-17 | 2.26E-16 |
| FQU82_RS15865 | mreD | rod shape-determining protein MreD | -1.508293709 | 4.54E-17 | 2.28E-16 |
| FQU82_RS09950 | FQU82_RS09950 | HlyD family efflux transporter periplasmic adaptor subunit | 2.457775967 | 4.58E-17 | 2.29E-16 |
| Novel00143 | - | PF00578:AhpC/TSA family | 1.633118451 | 5.26E-17 | 2.63E-16 |
| FQU82_RS01730 | ftsW | putative lipid II flippase FtsW | -0.858429784 | 5.30E-17 | 2.64E-16 |
| FQU82_RS17005 | FQU82_RS17005 | TRAP transporter large permease subunit | 0.790744359 | 6.13E-17 | 3.05E-16 |
| FQU82_RS05520 | FQU82_RS05520 | hypothetical protein | 3.549553091 | 6.14E-17 | 3.05E-16 |
| FQU82_RS01700 | FQU82_RS01700 | DsbC family protein | -1.088557305 | 7.55E-17 | 3.75E-16 |
| FQU82_RS05030 | pgaC | poly-beta-1,6-N-acetyl-D-glucosamine synthase | -1.029023906 | 8.72E-17 | 4.33E-16 |
| FQU82_RS03270 | FQU82_RS03270 | NAD(P)-dependent oxidoreductase | 0.885902285 | 9.06E-17 | 4.49E-16 |
| FQU82_RS05965 | FQU82_RS05965 | BON domain-containing protein | -0.872638954 | 1.16E-16 | 5.75E-16 |
| FQU82_RS13110 | FQU82_RS13110 | M48 family metalloprotease | 1.291660579 | 1.17E-16 | 5.76E-16 |
| FQU82_RS09365 | FQU82_RS09365 | DUF2058 domain-containing protein | -2.167343511 | 1.18E-16 | 5.84E-16 |
| FQU82_RS02750 | FQU82_RS02750 | biopolymer transporter ExbD | -0.966261919 | 1.25E-16 | 6.18E-16 |
| FQU82_RS08630 | FQU82_RS08630 | family 2A encapsulin nanocompartment shell protein | 2.11549524 | 1.28E-16 | 6.30E-16 |
| FQU82_RS16395 | FQU82_RS16395 | hypothetical protein | 1.847754896 | 1.41E-16 | 6.94E-16 |
| FQU82_RS09315 | trmA | tRNA (uridine(54)-C5)-methyltransferase TrmA | -2.206208357 | 1.62E-16 | 7.96E-16 |
| FQU82_RS17835 | FQU82_RS17835 | hypothetical protein | 1.276375596 | 1.64E-16 | 8.05E-16 |
| FQU82_RS17800 | gltX | glutamate--tRNA ligase | -0.804079056 | 1.74E-16 | 8.53E-16 |
| FQU82_RS15170 | FQU82_RS15170 | TetR/AcrR family transcriptional regulator | 1.655441779 | 1.76E-16 | 8.61E-16 |
| FQU82_RS00270 | FQU82_RS00270 | TerC family protein | 1.844713642 | 1.96E-16 | 9.56E-16 |
| FQU82_RS03005 | FQU82_RS03005 | phosphatase PAP2 family protein | 1.304762796 | 2.00E-16 | 9.76E-16 |
| FQU82_RS02185 | FQU82_RS02185 | Na+/H+ antiporter subunit E | -1.398157577 | 2.04E-16 | 9.93E-16 |
| FQU82_RS01375 | FQU82_RS01375 | DMT family transporter | 1.321847309 | 2.22E-16 | 1.08E-15 |
| FQU82_RS09555 | FQU82_RS09555 | integration host factor subunit beta | -0.680420436 | 2.30E-16 | 1.11E-15 |
| FQU82_RS03240 | FQU82_RS03240 | Re/Si-specific NAD(P)(+) transhydrogenase subunit alpha | -0.838508188 | 2.31E-16 | 1.12E-15 |
| FQU82_RS03335 | panC | pantoate--beta-alanine ligase | -0.854075533 | 2.85E-16 | 1.38E-15 |
| Novel00362 | - | PF00873:AcrB/AcrD/AcrF family|PF16576:Barrel-sandwich domain of CusB or HlyD membrane-fusion | 2.427743617 | 2.96E-16 | 1.43E-15 |
| FQU82_RS14235 | rpmE | 50S ribosomal protein L31 | -0.755719954 | 4.49E-16 | 2.17E-15 |
| Novel00289 | - | - | 2.45500757 | 4.52E-16 | 2.18E-15 |
| FQU82_RS09085 | FQU82_RS09085 | 1-acyl-sn-glycerol-3-phosphate acyltransferase | -3.159765091 | 4.64E-16 | 2.23E-15 |
| FQU82_RS09660 | FQU82_RS09660 | SOS response-associated peptidase family protein | 1.901044101 | 4.73E-16 | 2.27E-15 |
| FQU82_RS14325 | sohB | protease SohB | 0.680474997 | 5.34E-16 | 2.56E-15 |
| FQU82_RS04120 | FQU82_RS04120 | tRNA-dihydrouridine synthase | -2.255471837 | 6.06E-16 | 2.90E-15 |
| FQU82_RS15830 | FQU82_RS15830 | outer membrane protein transport protein | 0.650541261 | 6.89E-16 | 3.30E-15 |
| FQU82_RS15990 | FQU82_RS15990 | tRNA-Asp | -2.817884841 | 7.38E-16 | 3.53E-15 |
| FQU82_RS03750 | FQU82_RS03750 | acyl-CoA dehydrogenase | 1.224975562 | 8.24E-16 | 3.94E-15 |
| FQU82_RS01395 | def | peptide deformylase | 0.647336509 | 8.93E-16 | 4.26E-15 |
| FQU82_RS16330 | FQU82_RS16330 | metal-dependent hydrolase | 1.242270883 | 9.62E-16 | 4.58E-15 |
| FQU82_RS17210 | FQU82_RS17210 | metal-dependent hydrolase | 1.181146458 | 1.00E-15 | 4.77E-15 |
| FQU82_RS16995 | FQU82_RS16995 | YheV family putative zinc ribbon protein | 1.603839102 | 1.03E-15 | 4.89E-15 |
| FQU82_RS09540 | FQU82_RS09540 | SRPBCC family protein | -2.39234929 | 1.08E-15 | 5.10E-15 |
| FQU82_RS02085 | FQU82_RS02085 | YqgE/AlgH family protein | 0.578254118 | 1.31E-15 | 6.22E-15 |
| FQU82_RS15940 | FQU82_RS15940 | hypothetical protein | -2.039668543 | 1.42E-15 | 6.73E-15 |
| FQU82_RS02255 | FQU82_RS02255 | exodeoxyribonuclease V subunit gamma | 0.884371201 | 1.58E-15 | 7.46E-15 |
| FQU82_RS03330 | panB | 3-methyl-2-oxobutanoate hydroxymethyltransferase | -0.733344713 | 1.85E-15 | 8.71E-15 |
| FQU82_RS03150 | FQU82_RS03150 | GGDEF domain-containing phosphodiesterase | 1.20534487 | 2.15E-15 | 1.01E-14 |
| FQU82_RS14990 | sppA | signal peptide peptidase SppA | -0.826292634 | 2.66E-15 | 1.25E-14 |
| FQU82_RS09180 | FQU82_RS09180 | hypothetical protein | 2.034753373 | 3.29E-15 | 1.55E-14 |
| FQU82_RS11760 | glnG | nitrogen regulation protein NR(I) | -0.860492197 | 3.39E-15 | 1.59E-14 |
| FQU82_RS04070 | apbC | iron-sulfur cluster carrier protein ApbC | 0.873537814 | 3.57E-15 | 1.67E-14 |
| FQU82_RS04535 | FQU82_RS04535 | DNA translocase FtsK | 0.555382358 | 3.61E-15 | 1.69E-14 |
| Novel00301 | - | PF02812:Glu/Leu/Phe/Val dehydrogenase, dimerisation domain|PF00208:Glutamate/Leucine/Phenylalanine/Valine dehydrogenase | -1.81915308 | 3.92E-15 | 1.83E-14 |
| FQU82_RS03280 | FQU82_RS03280 | ATP-binding protein | 0.921979662 | 4.43E-15 | 2.07E-14 |
| FQU82_RS10635 | FQU82_RS10635 | hypothetical protein | -2.952013203 | 4.89E-15 | 2.28E-14 |
| FQU82_RS02520 | ptsP | phosphoenolpyruvate--protein phosphotransferase | 0.66811783 | 5.17E-15 | 2.40E-14 |
| FQU82_RS17545 | FQU82_RS17545 | TSUP family transporter | -1.774259515 | 5.65E-15 | 2.63E-14 |
| FQU82_RS19080 | FQU82_RS19080 | sodium-dependent transporter | 0.69989695 | 6.28E-15 | 2.92E-14 |
| FQU82_RS16625 | trxC | thioredoxin TrxC | 0.858493127 | 7.24E-15 | 3.36E-14 |
| FQU82_RS05305 | rubB | rubredoxin reductase RubB | -0.857053477 | 7.41E-15 | 3.43E-14 |
| FQU82_RS09020 | FQU82_RS09020 | MetQ/NlpA family ABC transporter substrate-binding protein | 3.383591621 | 7.67E-15 | 3.55E-14 |
| Novel00324 | - | PF02525:Flavodoxin-like fold | 9.228137635 | 8.18E-15 | 3.78E-14 |
| FQU82_RS02585 | infA | translation initiation factor IF-1 | -1.878728395 | 1.18E-14 | 5.43E-14 |
| FQU82_RS17550 | truB | tRNA pseudouridine(55) synthase TruB | -1.995079355 | 1.28E-14 | 5.91E-14 |
| FQU82_RS03345 | FQU82_RS03345 | HPr family phosphocarrier protein | 1.008328581 | 1.32E-14 | 6.06E-14 |
| FQU82_RS04230 | FQU82_RS04230 | L-threonylcarbamoyladenylate synthas | 0.821073706 | 1.53E-14 | 7.03E-14 |
| FQU82_RS16685 | FQU82_RS16685 | OmpA family protein | -1.297908216 | 1.69E-14 | 7.74E-14 |
| FQU82_RS10475 | FQU82_RS10475 | S-(hydroxymethyl)glutathione dehydrogenase/class III alcohol dehydrogenase | -0.78542098 | 1.74E-14 | 7.98E-14 |
| FQU82_RS14395 | FQU82_RS14395 | acyl-CoA desaturase | -1.29583149 | 1.80E-14 | 8.23E-14 |
| FQU82_RS18160 | FQU82_RS18160 | NAD-dependent succinate-semialdehyde dehydrogenase | 0.72670607 | 1.87E-14 | 8.57E-14 |
| FQU82_RS05005 | FQU82_RS05005 | GTP-binding protein | 1.642954784 | 1.89E-14 | 8.65E-14 |
| FQU82_RS14830 | FQU82_RS14830 | hypothetical protein | -0.596622392 | 3.36E-14 | 1.53E-13 |
| FQU82_RS04570 | FQU82_RS04570 | acyltransferase | -1.187580422 | 3.62E-14 | 1.65E-13 |
| FQU82_RS02580 | FQU82_RS02580 | lysozyme inhibitor LprI family protein | 1.326220208 | 3.89E-14 | 1.77E-13 |
| FQU82_RS05585 | FQU82_RS05585 | hypothetical protein | 0.677740109 | 4.00E-14 | 1.82E-13 |
| FQU82_RS11315 | FQU82_RS11315 | hypothetical protein | -0.636127376 | 4.27E-14 | 1.94E-13 |
| FQU82_RS04160 | FQU82_RS04160 | cell division protein ZipA C-terminal FtsZ-binding domain-containing protein | 0.699115382 | 4.75E-14 | 2.15E-13 |
| FQU82_RS06575 | rnhB | ribonuclease HII | -1.472312782 | 4.89E-14 | 2.21E-13 |
| FQU82_RS07970 | FQU82_RS07970 | epoxyqueuosine reductase QueH | -1.255054302 | 5.09E-14 | 2.30E-13 |
| FQU82_RS13780 | FQU82_RS13780 | ABC transporter permease | -1.273599619 | 5.30E-14 | 2.39E-13 |
| FQU82_RS12640 | dnaB | replicative DNA helicase | -1.140941336 | 5.75E-14 | 2.59E-13 |
| FQU82_RS16105 | FQU82_RS16105 | DsbC family protein | -1.063311628 | 5.78E-14 | 2.60E-13 |
| FQU82_RS17640 | FQU82_RS17640 | HD domain-containing protein | 0.715120124 | 5.90E-14 | 2.65E-13 |
| Novel00112 | - | - | 0.644460742 | 6.45E-14 | 2.89E-13 |
| FQU82_RS05435 | mrdA | penicillin-binding protein 2 | -1.093442981 | 6.98E-14 | 3.13E-13 |
| Novel00257 | - | PF00117:Glutamine amidotransferase class-I|PF02786:Carbamoyl-phosphate synthase L chain, ATP binding domain|PF02787:Carbamoyl-phosphate synthetase large chain, oligomerisation domain|PF02142:MGS-like domain|PF01272:Transcription elongation factor, GreA/GreB, C-term|PF03449:Transcription elongation factor, N-terminal | -1.221228823 | 8.38E-14 | 3.75E-13 |
| FQU82_RS07180 | FQU82_RS07180 | cold-shock protein | -1.055543697 | 8.51E-14 | 3.81E-13 |
| FQU82_RS18960 | FQU82_RS18960 | NAD(P)-dependent alcohol dehydrogenase | 0.783915904 | 8.54E-14 | 3.81E-13 |
| FQU82_RS02885 | metW | methionine biosynthesis protein MetW | 0.680126023 | 8.66E-14 | 3.86E-13 |
| FQU82_RS15115 | lysA | diaminopimelate decarboxylase | -0.714978968 | 9.60E-14 | 4.28E-13 |
| FQU82_RS16425 | FQU82_RS16425 | RcnB family protein | 2.203210031 | 1.05E-13 | 4.66E-13 |
| FQU82_RS12650 | FQU82_RS12650 | proteasome-type protease | 0.940650672 | 1.12E-13 | 5.00E-13 |
| FQU82_RS14920 | pal | peptidoglycan-associated lipoprotein Pal | -0.626365145 | 1.19E-13 | 5.29E-13 |
| FQU82_RS13915 | FQU82_RS13915 | multifunctional CCA addition/repair protein | 0.720773927 | 1.23E-13 | 5.45E-13 |
| FQU82_RS05595 | FQU82_RS05595 | LexA family transcriptional regulator | 0.967291221 | 1.55E-13 | 6.88E-13 |
| FQU82_RS12040 | FQU82_RS12040 | MaoC/PaaZ C-terminal domain-containing protein | -0.989617788 | 1.72E-13 | 7.59E-13 |
| FQU82_RS16890 | FQU82_RS16890 | PhoH family protein | 0.508143708 | 1.77E-13 | 7.80E-13 |
| FQU82_RS16435 | FQU82_RS16435 | phospholipase D family protein | 0.747514098 | 1.78E-13 | 7.87E-13 |
| FQU82_RS01245 | FQU82_RS01245 | pirin family protein | 0.770449129 | 1.87E-13 | 8.23E-13 |
| FQU82_RS09420 | FQU82_RS09420 | peptidylprolyl isomerase | -0.775139776 | 1.98E-13 | 8.71E-13 |
| FQU82_RS08940 | FQU82_RS08940 | glutathione peroxidase | 0.91211926 | 2.01E-13 | 8.82E-13 |
| Novel00047 | - | - | 5.149209387 | 2.07E-13 | 9.07E-13 |
| FQU82_RS07010 | FQU82_RS07010 | glutathione binding-like protein | -0.811612109 | 2.33E-13 | 1.02E-12 |
| FQU82_RS03020 | rlmN | 23S rRNA (adenine(2503)-C(2))-methyltransferase RlmN | 0.572458905 | 2.46E-13 | 1.08E-12 |
| FQU82_RS10305 | FQU82_RS10305 | AraC family transcriptional regulator | 1.815273203 | 2.55E-13 | 1.12E-12 |
| FQU82_RS01845 | gspD | type II secretion system secretin GspD | -0.993003366 | 2.64E-13 | 1.15E-12 |
| FQU82_RS08320 | FQU82_RS08320 | CaiB/BaiF CoA-transferase family protein | -1.039040569 | 2.78E-13 | 1.21E-12 |
| FQU82_RS04540 | rhtC | threonine export protein RhtC | 0.851467845 | 2.82E-13 | 1.23E-12 |
| FQU82_RS12980 | FQU82_RS12980 | hypothetical protein | -2.180475529 | 2.86E-13 | 1.24E-12 |
| FQU82_RS02310 | FQU82_RS02310 | monovalent cation:proton antiporter-2 (CPA2) family protein | 0.839013191 | 2.97E-13 | 1.29E-12 |
| FQU82_RS15930 | FQU82_RS15930 | alanine/glycine:cation symporter family protein | -2.835947998 | 3.05E-13 | 1.32E-12 |
| FQU82_RS14730 | FQU82_RS14730 | DUF4845 domain-containing protein | -1.530311586 | 3.33E-13 | 1.44E-12 |
| FQU82_RS16885 | miaB | tRNA (N6-isopentenyl adenosine(37)-C2)-methylthiotransferase MiaB | -0.649280635 | 3.41E-13 | 1.48E-12 |
| FQU82_RS01840 | FQU82_RS01840 | type II secretion system protein N | -1.151104548 | 3.48E-13 | 1.51E-12 |
| FQU82_RS14045 | FQU82_RS14045 | DUF4105 domain-containing protein | -1.409452689 | 4.30E-13 | 1.86E-12 |
| FQU82_RS18640 | FQU82_RS18640 | hypothetical protein | 0.824615645 | 4.33E-13 | 1.87E-12 |
| FQU82_RS03690 | FQU82_RS03690 | cation transporter | 1.975815445 | 4.36E-13 | 1.88E-12 |
| FQU82_RS05210 | FQU82_RS05210 | hypothetical protein | 1.846890234 | 4.41E-13 | 1.90E-12 |
| FQU82_RS11875 | moaA | GTP 3',8-cyclase MoaA | 1.208494496 | 4.41E-13 | 1.90E-12 |
| FQU82_RS09025 | FQU82_RS09025 | MetQ/NlpA family ABC transporter substrate-binding protein | 3.731857128 | 4.90E-13 | 2.11E-12 |
| FQU82_RS09925 | FQU82_RS09925 | PepSY-associated TM helix domain-containing protein | -2.602066276 | 5.47E-13 | 2.35E-12 |
| FQU82_RS16895 | ybeY | rRNA maturation RNase YbeY | 0.73267278 | 6.36E-13 | 2.73E-12 |
| FQU82_RS12525 | prmC | peptide chain release factor N(5)-glutamine methyltransferase | -1.684352991 | 7.02E-13 | 3.01E-12 |
| FQU82_RS10410 | pqqB | pyrroloquinoline quinone biosynthesis protein PqqB | -1.370421357 | 7.45E-13 | 3.19E-12 |
| FQU82_RS13305 | FQU82_RS13305 | DUF4010 domain-containing protein | 1.654060826 | 7.76E-13 | 3.31E-12 |
| FQU82_RS11510 | FQU82_RS11510 | aspartate aminotransferase family protein | -0.885832495 | 8.29E-13 | 3.54E-12 |
| FQU82_RS15370 | ftsH | ATP-dependent zinc metalloprotease FtsH | 0.424663112 | 8.59E-13 | 3.66E-12 |
| FQU82_RS15985 | FQU82_RS15985 | tRNA-Val | -2.847029223 | 8.71E-13 | 3.71E-12 |
| Novel00007 | - | PF14849:YidC periplasmic domain|PF02096:60Kd inner membrane protein|PF10396:GTP-binding protein TrmE N-terminus|PF01809:Putative membrane protein insertion efficiency factor | -1.279380327 | 9.03E-13 | 3.84E-12 |
| FQU82_RS17710 | aroB | 3-dehydroquinate synthase | -0.738706461 | 1.09E-12 | 4.63E-12 |
| FQU82_RS11045 | FQU82_RS11045 | LysR family transcriptional regulator | 1.331745607 | 1.10E-12 | 4.66E-12 |
| FQU82_RS16175 | FQU82_RS16175 | RtcB family protein | -1.607466977 | 1.17E-12 | 4.94E-12 |
| FQU82_RS05970 | FQU82_RS05970 | alpha/beta hydrolase | -1.36081658 | 1.25E-12 | 5.30E-12 |
| FQU82_RS02850 | FQU82_RS02850 | DUF839 domain-containing protein | -2.159903769 | 1.30E-12 | 5.50E-12 |
| FQU82_RS08315 | FQU82_RS08315 | acyl-CoA dehydrogenase family protein | -1.975895199 | 1.50E-12 | 6.33E-12 |
| FQU82_RS05940 | FQU82_RS05940 | LysE/ArgO family amino acid transporter | 1.666679536 | 1.57E-12 | 6.63E-12 |
| FQU82_RS18380 | FQU82_RS18380 | putative porin | 0.482498394 | 1.69E-12 | 7.12E-12 |
| FQU82_RS18595 | parE | DNA topoisomerase IV subunit B | 0.652859172 | 1.78E-12 | 7.50E-12 |
| FQU82_RS09435 | FQU82_RS09435 | nucleotidyltransferase family protein | -1.033772462 | 1.83E-12 | 7.70E-12 |
| FQU82_RS17620 | ispH | 4-hydroxy-3-methylbut-2-enyl diphosphate reductase | 0.685884521 | 1.90E-12 | 7.96E-12 |
| FQU82_RS09560 | FQU82_RS09560 | lipopolysaccharide assembly protein LapA domain-containing protein | -0.697253366 | 2.13E-12 | 8.92E-12 |
| FQU82_RS16135 | mutM | bifunctional DNA-formamidopyrimidine glycosylase/DNA-(apurinic or apyrimidinic site) lyase | 0.863338679 | 2.31E-12 | 9.65E-12 |
| Novel00360 | - | PF00873:AcrB/AcrD/AcrF family | 2.296617169 | 2.32E-12 | 9.70E-12 |
| Novel00200 | - | PF00510:Cytochrome c oxidase subunit III|PF01281:Ribosomal protein L9, N-terminal domain|PF03948:Ribosomal protein L9, C-terminal domain|PF00772:DnaB-like helicase N terminal domain|PF01040:UbiA prenyltransferase family|PF00115:Cytochrome C and Quinol oxidase polypeptide I|PF03626:Prokaryotic Cytochrome C oxidase subunit IV|PF01250:Ribosomal protein S6 | -1.447523689 | 2.34E-12 | 9.78E-12 |
| FQU82_RS12265 | FQU82_RS12265 | iron-containing alcohol dehydrogenase | -1.679106715 | 2.37E-12 | 9.86E-12 |
| FQU82_RS17465 | FQU82_RS17465 | hypothetical protein | 0.940108932 | 2.62E-12 | 1.09E-11 |
| FQU82_RS08100 | paaF | phenylacetate--CoA ligase | 1.39909266 | 2.66E-12 | 1.10E-11 |
| FQU82_RS03665 | rpe | ribulose-phosphate 3-epimerase | -0.689467628 | 2.68E-12 | 1.11E-11 |
| FQU82_RS07050 | FQU82_RS07050 | beta-ketoacyl-ACP synthase III | -0.590136032 | 2.69E-12 | 1.11E-11 |
| FQU82_RS18360 | FQU82_RS18360 | 3-deoxy-D-manno-octulosonic acid transferase | -1.062317402 | 3.23E-12 | 1.34E-11 |
| FQU82_RS15850 | FQU82_RS15850 | hypothetical protein | 1.358277641 | 3.67E-12 | 1.52E-11 |
| FQU82_RS09225 | FQU82_RS09225 | hypothetical protein | 1.540426929 | 3.90E-12 | 1.61E-11 |
| FQU82_RS00405 | lspA | signal peptidase II | -0.642871378 | 3.96E-12 | 1.64E-11 |
| FQU82_RS12560 | FQU82_RS12560 | hypothetical protein | 2.513472378 | 4.12E-12 | 1.70E-11 |
| FQU82_RS08935 | msrB | peptide-methionine (R)-S-oxide reductase MsrB | 0.762477119 | 4.22E-12 | 1.74E-11 |
| FQU82_RS04110 | FQU82_RS04110 | hypothetical protein | 1.074549389 | 4.69E-12 | 1.93E-11 |
| FQU82_RS07090 | FQU82_RS07090 | LysR family transcriptional regulator | 1.488862851 | 5.16E-12 | 2.12E-11 |
| FQU82_RS11460 | cydX | cytochrome bd-I oxidase subunit CydX | -1.283438957 | 5.51E-12 | 2.26E-11 |
| FQU82_RS13805 | FQU82_RS13805 | septal ring lytic transglycosylase RlpA family protein | -0.754746568 | 6.14E-12 | 2.52E-11 |
| FQU82_RS03720 | FQU82_RS03720 | tRNA-Arg | -2.157580792 | 6.40E-12 | 2.62E-11 |
| FQU82_RS08225 | FQU82_RS08225 | aldehyde dehydrogenase | 1.484670997 | 6.41E-12 | 2.62E-11 |
| FQU82_RS11685 | FQU82_RS11685 | YbgF trimerization domain-containing protein | -1.379507533 | 7.78E-12 | 3.18E-11 |
| FQU82_RS14210 | FQU82_RS14210 | carbon starvation CstA family protein | 0.601462185 | 7.92E-12 | 3.23E-11 |
| FQU82_RS01685 | pbpG | D-alanyl-D-alanine endopeptidase PBP7/8 | 0.50448571 | 8.21E-12 | 3.35E-11 |
| FQU82_RS04260 | acpP | acyl carrier protein | -1.097932316 | 9.44E-12 | 3.85E-11 |
| FQU82_RS12300 | FQU82_RS12300 | glutamine amidotransferase | -1.955027591 | 9.80E-12 | 3.99E-11 |
| FQU82_RS11865 | FQU82_RS11865 | molybdenum cofactor biosynthesis protein MoaE | 1.651703295 | 1.12E-11 | 4.57E-11 |
| FQU82_RS17775 | ftsI | penicillin-binding protein PBP3 | -0.666986394 | 1.15E-11 | 4.66E-11 |
| FQU82_RS17990 | FQU82_RS17990 | homoserine kinase | -0.988227532 | 1.15E-11 | 4.67E-11 |
| FQU82_RS08775 | FQU82_RS08775 | cytochrome ubiquinol oxidase subunit I | 1.002698593 | 1.27E-11 | 5.14E-11 |
| Novel00055 | - | PF08028:Acyl-CoA dehydrogenase, C-terminal domain | 2.276700395 | 1.33E-11 | 5.38E-11 |
| FQU82_RS16080 | FQU82_RS16080 | YegP family protein | 1.061194201 | 1.35E-11 | 5.48E-11 |
| FQU82_RS02105 | rlmB | 23S rRNA (guanosine(2251)-2'-O)-methyltransferase RlmB | -0.954921053 | 1.36E-11 | 5.50E-11 |
| FQU82_RS02715 | rpmG | 50S ribosomal protein L33 | -1.603578971 | 1.43E-11 | 5.77E-11 |
| FQU82_RS09790 | FQU82_RS09790 | acyl-CoA dehydrogenase family protein | 1.301493134 | 1.48E-11 | 5.95E-11 |
| FQU82_RS03140 | ilvN | acetolactate synthase small subunit | -0.633788794 | 1.66E-11 | 6.69E-11 |
| FQU82_RS10190 | FQU82_RS10190 | ABC transporter permease | 3.163233742 | 1.66E-11 | 6.69E-11 |
| FQU82_RS10415 | pqqC | pyrroloquinoline-quinone synthase PqqC | -1.033020799 | 1.75E-11 | 7.01E-11 |
| FQU82_RS12735 | FQU82_RS12735 | dicarboxylate/amino acid:cation symporter | -0.97061132 | 1.88E-11 | 7.55E-11 |
| FQU82_RS17905 | ompR | two-component system response regulator OmpR | 0.497530436 | 1.93E-11 | 7.73E-11 |
| FQU82_RS00295 | purE | 5-(carboxyamino)imidazole ribonucleotide mutase | -0.964956425 | 2.05E-11 | 8.22E-11 |
| FQU82_RS12305 | FQU82_RS12305 | glutamine--tRNA ligase/YqeY domain fusion protein | -0.553679907 | 2.09E-11 | 8.33E-11 |
| FQU82_RS14655 | FQU82_RS14655 | pyridoxal phosphate-dependent aminotransferase | -0.76496246 | 2.15E-11 | 8.56E-11 |
| FQU82_RS09585 | zapE | cell division protein ZapE | -0.743436287 | 2.18E-11 | 8.68E-11 |
| FQU82_RS11955 | FQU82_RS11955 | iron-containing redox enzyme family protein | 0.90662031 | 2.27E-11 | 9.02E-11 |
| FQU82_RS04695 | FQU82_RS04695 | EmrA/EmrK family multidrug efflux transporter periplasmic adaptor subunit | 0.688411296 | 2.39E-11 | 9.51E-11 |
| FQU82_RS01675 | FQU82_RS01675 | PAS domain-containing sensor histidine kinase | 1.596426022 | 2.46E-11 | 9.78E-11 |
| FQU82_RS16840 | FQU82_RS16840 | hypothetical protein | 1.774757169 | 2.50E-11 | 9.93E-11 |
| FQU82_RS02200 | FQU82_RS02200 | monovalent cation/H+ antiporter subunit A | -0.567541567 | 2.52E-11 | 9.98E-11 |
| FQU82_RS03065 | secB | protein-export chaperone SecB | 0.499219823 | 2.72E-11 | 1.08E-10 |
| FQU82_RS03580 | hisD | histidinol dehydrogenase | -0.619123126 | 4.04E-11 | 1.60E-10 |
| FQU82_RS18165 | gabT | 4-aminobutyrate--2-oxoglutarate transaminase | 1.439584788 | 4.17E-11 | 1.65E-10 |
| Novel00245 | - | PF02769:AIR synthase related protein, C-terminal domain|PF13507:CobB/CobQ-like glutamine amidotransferase domain | -1.366847039 | 4.79E-11 | 1.89E-10 |
| FQU82_RS14270 | surE | 5'/3'-nucleotidase SurE | -1.440361152 | 4.93E-11 | 1.94E-10 |
| FQU82_RS13900 | FQU82_RS13900 | 16S rRNA (uracil(1498)-N(3))-methyltransferase | -1.800544654 | 5.06E-11 | 1.99E-10 |
| FQU82_RS05235 | FQU82_RS05235 | TonB-dependent siderophore receptor | -1.284475183 | 5.07E-11 | 1.99E-10 |
| FQU82_RS14250 | FQU82_RS14250 | MFS transporter | -0.834338339 | 6.74E-11 | 2.65E-10 |
| FQU82_RS09545 | cmk | (d)CMP kinase | -0.579580522 | 6.77E-11 | 2.66E-10 |
| FQU82_RS10400 | ppk1 | polyphosphate kinase 1 | 0.925802606 | 6.94E-11 | 2.72E-10 |
| FQU82_RS17220 | FQU82_RS17220 | acyl-CoA thioesterase II | 0.619133696 | 7.20E-11 | 2.82E-10 |
| FQU82_RS12260 | FQU82_RS12260 | AAA family ATPase | -1.100600974 | 7.29E-11 | 2.85E-10 |
| Novel00203 | - | PF01808:AICARFT/IMPCHase bienzyme|PF02843:Phosphoribosylglycinamide synthetase, C domain|PF01071:Phosphoribosylglycinamide synthetase, ATP-grasp (A) domain|PF02844:Phosphoribosylglycinamide synthetase, N domain | -1.498817842 | 7.38E-11 | 2.89E-10 |
| Novel00286 | - | PF02866:lactate/malate dehydrogenase, alpha/beta C-terminal domain|PF00056:lactate/malate dehydrogenase, NAD binding domain | -1.661155705 | 7.67E-11 | 2.99E-10 |
| FQU82_RS01565 | FQU82_RS01565 | OprD family outer membrane porin | -0.503053348 | 8.82E-11 | 3.44E-10 |
| FQU82_RS14580 | FQU82_RS14580 | nitronate monooxygenase | 0.721226446 | 9.59E-11 | 3.73E-10 |
| FQU82_RS13785 | queF | NADPH-dependent 7-cyano-7-deazaguanine reductase QueF | -0.887422855 | 1.08E-10 | 4.21E-10 |
| FQU82_RS14700 | FQU82_RS14700 | tRNA-(ms[2]io[6]A)-hydroxylase | -1.022036681 | 1.34E-10 | 5.19E-10 |
| FQU82_RS12725 | gloB | hydroxyacylglutathione hydrolase | 0.920771948 | 1.35E-10 | 5.26E-10 |
| FQU82_RS09795 | FQU82_RS09795 | acyl-CoA dehydrogenase family protein | 1.103901005 | 1.42E-10 | 5.49E-10 |
| FQU82_RS03925 | FQU82_RS03925 | NADH-quinone oxidoreductase subunit B | -0.549962219 | 1.46E-10 | 5.65E-10 |
| FQU82_RS12570 | pgaC | poly-beta-1,6-N-acetyl-D-glucosamine synthase | 2.173252735 | 1.65E-10 | 6.37E-10 |
| FQU82_RS17000 | FQU82_RS17000 | hypothetical protein | 1.853706024 | 1.74E-10 | 6.73E-10 |
| FQU82_RS09700 | FQU82_RS09700 | Dyp-type peroxidase | -0.766241109 | 1.93E-10 | 7.45E-10 |
| FQU82_RS02600 | FQU82_RS02600 | asparaginase | -0.957749934 | 2.12E-10 | 8.16E-10 |
| FQU82_RS16605 | FQU82_RS16605 | rhodanese-like domain-containing protein | 0.736027415 | 2.39E-10 | 9.21E-10 |
| FQU82_RS05220 | FQU82_RS05220 | arylsulfatase | 1.613315125 | 2.73E-10 | 1.05E-09 |
| FQU82_RS11715 | rseP | RIP metalloprotease RseP | -0.65308137 | 2.79E-10 | 1.07E-09 |
| FQU82_RS12045 | FQU82_RS12045 | 3-oxoacyl-ACP reductase | -0.486725036 | 3.00E-10 | 1.15E-09 |
| FQU82_RS06035 | pyrC | dihydroorotase | -0.586077514 | 3.11E-10 | 1.19E-09 |
| FQU82_RS09300 | FQU82_RS09300 | lecithin retinol acyltransferase family protein | 1.292506441 | 3.14E-10 | 1.20E-09 |
| FQU82_RS01885 | tuf | elongation factor Tu | -2.803778395 | 3.23E-10 | 1.23E-09 |
| FQU82_RS07515 | FQU82_RS07515 | KpsF/GutQ family sugar-phosphate isomerase | -0.677774089 | 3.25E-10 | 1.24E-09 |
| Novel00079 | - | PF02556:Preprotein translocase subunit SecB|PF00581:Rhodanese-like domain|PF00462:Glutaredoxin | 1.856265584 | 3.27E-10 | 1.25E-09 |
| FQU82_RS08645 | FQU82_RS08645 | SDR family oxidoreductase | 2.49903698 | 3.32E-10 | 1.27E-09 |
| FQU82_RS13230 | pheA | prephenate dehydratase | -0.553343558 | 3.47E-10 | 1.32E-09 |
| FQU82_RS01250 | FQU82_RS01250 | OsmC family protein | 0.964600821 | 3.54E-10 | 1.35E-09 |
| FQU82_RS05360 | FQU82_RS05360 | CitMHS family transporter | -0.582784649 | 3.54E-10 | 1.35E-09 |
| FQU82_RS00910 | lldD | FMN-dependent L-lactate dehydrogenase LldD | 1.065961201 | 3.93E-10 | 1.49E-09 |
| FQU82_RS18450 | lpxC | UDP-3-O-acyl-N-acetylglucosamine deacetylase | -1.039683165 | 4.40E-10 | 1.67E-09 |
| FQU82_RS11765 | adeN | multidrug efflux transcriptional repressor AdeN | 1.210900707 | 4.68E-10 | 1.77E-09 |
| FQU82_RS14675 | FQU82_RS14675 | PaaI family thioesterase | 1.519341987 | 4.72E-10 | 1.79E-09 |
| FQU82_RS15325 | FQU82_RS15325 | 50S ribosomal protein L11 methyltransferase | -1.86365397 | 4.72E-10 | 1.79E-09 |
| FQU82_RS18255 | FQU82_RS18255 | DUF1304 domain-containing protein | 0.7574793 | 5.30E-10 | 2.00E-09 |
| FQU82_RS12535 | FQU82_RS12535 | type 1 glutamine amidotransferase domain-containing protein | 2.522717026 | 5.35E-10 | 2.02E-09 |
| FQU82_RS09645 | FQU82_RS09645 | ABC transporter permease | -1.37159069 | 5.43E-10 | 2.05E-09 |
| FQU82_RS11415 | FQU82_RS11415 | phospholipase A | -0.714086485 | 5.78E-10 | 2.18E-09 |
| FQU82_RS07740 | FQU82_RS07740 | biotin carboxylase N-terminal domain-containing protein | -0.679982068 | 5.78E-10 | 2.18E-09 |
| FQU82_RS17015 | FQU82_RS17015 | hypothetical protein | 0.651102881 | 6.17E-10 | 2.32E-09 |
| Novel00174 | - | PF03952:Enolase, N-terminal domain|PF00113:Enolase, C-terminal TIM barrel domain|PF00793:DAHP synthetase I family | -1.764318561 | 6.34E-10 | 2.38E-09 |
| FQU82_RS02340 | lnt | apolipoprotein N-acyltransferase | -0.825017329 | 6.54E-10 | 2.45E-09 |
| FQU82_RS16790 | FQU82_RS16790 | DUF1446 domain-containing protein | 1.285151284 | 6.55E-10 | 2.45E-09 |
| FQU82_RS08650 | FQU82_RS08650 | LysR family transcriptional regulator | 1.584612487 | 6.59E-10 | 2.47E-09 |
| FQU82_RS05070 | FQU82_RS05070 | aromatic ring-hydroxylating dioxygenase subunit alpha | 1.714055513 | 6.61E-10 | 2.47E-09 |
| FQU82_RS15125 | FQU82_RS15125 | amino acid permease | -0.508837542 | 6.63E-10 | 2.47E-09 |
| FQU82_RS16235 | FQU82_RS16235 | AraC family transcriptional regulator | 0.611934978 | 7.51E-10 | 2.80E-09 |
| FQU82_RS11810 | fumC | class II fumarate hydratase | 0.554564693 | 7.66E-10 | 2.86E-09 |
| FQU82_RS13120 | FQU82_RS13120 | CvpA family protein | 1.468916699 | 7.94E-10 | 2.96E-09 |
| FQU82_RS14935 | FQU82_RS14935 | RNA methyltransferase | -1.999027209 | 8.28E-10 | 3.08E-09 |
| FQU82_RS15180 | FQU82_RS15180 | nitroreductase family protein | 0.689740382 | 8.36E-10 | 3.11E-09 |
| FQU82_RS11470 | FQU82_RS11470 | DUF2057 domain-containing protein | -2.385602227 | 8.47E-10 | 3.15E-09 |
| FQU82_RS10185 | FQU82_RS10185 | ABC transporter substrate-binding protein | 1.990831164 | 9.39E-10 | 3.48E-09 |
| FQU82_RS18300 | FQU82_RS18300 | DUF485 domain-containing protein | -0.783326481 | 1.03E-09 | 3.82E-09 |
| FQU82_RS09985 | FQU82_RS09985 | porin | -2.312920863 | 1.05E-09 | 3.90E-09 |
| FQU82_RS16280 | FQU82_RS16280 | hypothetical protein | -0.602793992 | 1.05E-09 | 3.90E-09 |
| FQU82_RS12470 | kdpB | potassium-transporting ATPase subunit KdpB | 1.644906272 | 1.18E-09 | 4.34E-09 |
| Novel00287 | - | PF09526:Probable metal-binding protein (DUF2387)|PF01432:Peptidase family M3 | 1.602158018 | 1.24E-09 | 4.56E-09 |
| FQU82_RS13165 | FQU82_RS13165 | cold-shock protein | -0.604553241 | 1.26E-09 | 4.63E-09 |
| FQU82_RS18460 | ftsA | cell division protein FtsA | -0.484394592 | 1.29E-09 | 4.73E-09 |
| FQU82_RS15980 | FQU82_RS15980 | tRNA-Asp | -3.022090167 | 1.36E-09 | 4.99E-09 |
| FQU82_RS04085 | FQU82_RS04085 | LysE family transporter | 0.725182944 | 1.38E-09 | 5.06E-09 |
| FQU82_RS13995 | FQU82_RS13995 | M1 family metallopeptidase | -1.050175416 | 1.43E-09 | 5.26E-09 |
| FQU82_RS01185 | FQU82_RS01185 | BolA family protein | 0.90588401 | 1.45E-09 | 5.31E-09 |
| Novel00071 | - | PF11008:Protein of unknown function (DUF2846)|PF00574:Clp protease|PF07724:AAA domain (Cdc48 subfamily)|PF10431:C-terminal, D2-small domain, of ClpB protein|PF06689:ClpX C4-type zinc finger | 1.003419827 | 1.45E-09 | 5.31E-09 |
| FQU82_RS09715 | FQU82_RS09715 | HIT domain-containing protein | -0.571744554 | 1.47E-09 | 5.37E-09 |
| Novel00026 | - | PF00958:GMP synthase C terminal domain | -1.400420477 | 1.54E-09 | 5.63E-09 |
| FQU82_RS16690 | FQU82_RS16690 | metallophosphoesterase | 0.756975757 | 1.83E-09 | 6.70E-09 |
| FQU82_RS17275 | FQU82_RS17275 | outer membrane lipid asymmetry maintenance protein MlaD | -0.508200472 | 1.85E-09 | 6.74E-09 |
| FQU82_RS09955 | FQU82_RS09955 | ATP-binding cassette domain-containing protein | 1.768275533 | 1.86E-09 | 6.77E-09 |
| FQU82_RS00930 | prpB | methylisocitrate lyase | -0.724717277 | 1.86E-09 | 6.77E-09 |
| FQU82_RS14480 | FQU82_RS14480 | YbfB/YjiJ family MFS transporte | 3.395054214 | 2.00E-09 | 7.25E-09 |
| FQU82_RS15595 | serS | serine--tRNA ligase | -0.745084789 | 2.05E-09 | 7.45E-09 |
| FQU82_RS11700 | lpxD | UDP-3-O-(3-hydroxymyristoyl)glucosamine N-acyltransferase | -0.753841007 | 2.28E-09 | 8.28E-09 |
| FQU82_RS00010 | FQU82_RS00010 | plasmid replication DNA-binding protein | 1.129531126 | 2.41E-09 | 8.73E-09 |
| FQU82_RS18470 | FQU82_RS18470 | D-alanine--D-alanine ligase | -0.630254065 | 2.43E-09 | 8.79E-09 |
| FQU82_RS00145 | FQU82_RS00145 | DUF6091 family protein | 1.392394515 | 2.44E-09 | 8.84E-09 |
| FQU82_RS17695 | FQU82_RS17695 | glutamate synthase subunit beta | 0.477073639 | 2.48E-09 | 8.96E-09 |
| FQU82_RS10005 | FQU82_RS10005 | hypothetical protein | 1.268754352 | 2.56E-09 | 9.26E-09 |
| FQU82_RS15025 | FQU82_RS15025 | hypothetical protein | 0.630012465 | 2.58E-09 | 9.31E-09 |
| FQU82_RS01530 | FQU82_RS01530 | trimeric intracellular cation channel family protein | -1.607305019 | 2.75E-09 | 9.89E-09 |
| FQU82_RS03045 | FQU82_RS03045 | tetratricopeptide repeat protein | -0.666490877 | 2.80E-09 | 1.01E-08 |
| FQU82_RS14450 | FQU82_RS14450 | hypothetical protein | 0.762250601 | 2.85E-09 | 1.02E-08 |
| FQU82_RS01880 | FQU82_RS01880 | tRNA-Thr | -3.30967936 | 2.85E-09 | 1.02E-08 |
| FQU82_RS13135 | gspL | type II secretion system protein GspL | -0.886462021 | 2.87E-09 | 1.03E-08 |
| FQU82_RS00495 | FQU82_RS00495 | TetR/AcrR family transcriptional regulator | 0.923520624 | 3.04E-09 | 1.09E-08 |
| FQU82_RS00915 | dld | D-lactate dehydrogenase | 1.63283831 | 3.14E-09 | 1.12E-08 |
| FQU82_RS07870 | tssE | type VI secretion system baseplate subunit TssE | 2.008156549 | 3.36E-09 | 1.20E-08 |
| FQU82_RS18505 | FQU82_RS18505 | exodeoxyribonuclease III | -0.73596535 | 3.39E-09 | 1.21E-08 |
| Novel00101 | - | PF00317:Ribonucleotide reductase, all-alpha domain|PF02867:Ribonucleotide reductase, barrel domain|PF03477:ATP cone domain | 0.869125965 | 3.56E-09 | 1.27E-08 |
| FQU82_RS13250 | FQU82_RS13250 | glycosyl transferase family protein | 0.767161619 | 3.84E-09 | 1.37E-08 |
| FQU82_RS10630 | FQU82_RS10630 | GH3 auxin-responsive promoter family protein | -2.409444625 | 3.91E-09 | 1.39E-08 |
| FQU82_RS04475 | FQU82_RS04475 | beta-ketoacyl-ACP synthase II | -0.556455274 | 4.00E-09 | 1.42E-08 |
| FQU82_RS00275 | FQU82_RS00275 | phosphatase PAP2 family protein | -2.806168511 | 4.09E-09 | 1.46E-08 |
| FQU82_RS00465 | FQU82_RS00465 | RcnB family protein | 1.993714505 | 4.14E-09 | 1.47E-08 |
| FQU82_RS03515 | gigB | anti-anti-sigma factor GigB | 0.566491406 | 4.24E-09 | 1.51E-08 |
| FQU82_RS05580 | FQU82_RS05580 | hypothetical protein | 0.915763488 | 4.26E-09 | 1.51E-08 |
| FQU82_RS14845 | FQU82_RS14845 | CAP domain-containing protein | 2.106057348 | 4.38E-09 | 1.55E-08 |
| Novel00274 | - | PF10417:C-terminal domain of 1-Cys peroxiredoxin|PF00753:Metallo-beta-lactamase superfamily | 1.756501675 | 4.40E-09 | 1.56E-08 |
| FQU82_RS05265 | FQU82_RS05265 | FtsX-like permease family protein | 0.893743739 | 4.41E-09 | 1.56E-08 |
| FQU82_RS03705 | argJ | bifunctional glutamate N-acetyltransferase/amino-acid acetyltransferase ArgJ | -0.550230994 | 4.49E-09 | 1.58E-08 |
| FQU82_RS05055 | FQU82_RS05055 | IclR family transcriptional regulator | 1.298689158 | 4.50E-09 | 1.59E-08 |
| FQU82_RS12895 | proP | glycine betaine/L-proline transporter ProP | -1.499224119 | 4.66E-09 | 1.64E-08 |
| Novel00294 | - | PF00271:Helicase conserved C-terminal domain|PF00270:DEAD/DEAH box helicase | -0.967996364 | 4.86E-09 | 1.71E-08 |
| FQU82_RS10250 | FQU82_RS10250 | TIGR00366 family protein | 1.176980108 | 5.19E-09 | 1.83E-08 |
| FQU82_RS04355 | FQU82_RS04355 | DNA primase | 0.755084923 | 5.47E-09 | 1.92E-08 |
| FQU82_RS03575 | hisG | ATP phosphoribosyltransferase | -0.83755591 | 5.59E-09 | 1.96E-08 |
| FQU82_RS02410 | FQU82_RS02410 | HopJ type III effector protein | -1.081031334 | 5.82E-09 | 2.04E-08 |
| FQU82_RS16245 | FQU82_RS16245 | 3-hydroxyacyl-CoA dehydrogenase NAD-binding domain-containing protein | 0.774736598 | 6.15E-09 | 2.15E-08 |
| Novel00212 | - | - | 2.786091042 | 6.40E-09 | 2.24E-08 |
| FQU82_RS14905 | tolR | protein TolR | -2.068093861 | 6.45E-09 | 2.26E-08 |
| FQU82_RS03120 | FQU82_RS03120 | hypothetical protein | -0.512788887 | 6.56E-09 | 2.29E-08 |
| FQU82_RS15925 | FQU82_RS15925 | patatin-like phospholipase family protein | 1.162157282 | 6.65E-09 | 2.32E-08 |
| FQU82_RS11495 | FQU82_RS11495 | hypothetical protein | 1.180396698 | 6.70E-09 | 2.34E-08 |
| FQU82_RS01750 | FQU82_RS01750 | NUDIX domain-containing protein | 0.871869696 | 7.04E-09 | 2.45E-08 |
| FQU82_RS02450 | FQU82_RS02450 | SDR family oxidoreductase | 1.916870983 | 7.05E-09 | 2.45E-08 |
| FQU82_RS08200 | FQU82_RS08200 | GntR family transcriptional regulator | 1.11691721 | 7.35E-09 | 2.56E-08 |
| sRNA00046 | - | - | 2.515622814 | 7.93E-09 | 2.75E-08 |
| FQU82_RS00310 | FQU82_RS00310 | hypothetical protein | 0.655598177 | 8.48E-09 | 2.94E-08 |
| FQU82_RS11860 | moaCB | bifunctional molybdenum cofactor biosynthesis protein MoaC/MoaB | 0.795294681 | 8.56E-09 | 2.97E-08 |
| Novel00227 | - | PF00206:Lyase|PF08328:Adenylosuccinate lyase C-terminal|PF09997:Predicted membrane protein (DUF2238) | -1.737977045 | 9.08E-09 | 3.14E-08 |
| FQU82_RS14775 | pabC | aminodeoxychorismate lyase | 1.181309112 | 9.32E-09 | 3.22E-08 |
| FQU82_RS16390 | baeS | sensor histidine kinase efflux regulator BaeS | 0.65811311 | 9.45E-09 | 3.26E-08 |
| FQU82_RS10075 | FQU82_RS10075 | helix-turn-helix domain-containing protein | 0.926034821 | 9.88E-09 | 3.41E-08 |
| FQU82_RS09860 | FQU82_RS09860 | PepSY-associated TM helix domain-containing protein | -1.621195227 | 1.01E-08 | 3.47E-08 |
| FQU82_RS00185 | dnaN | DNA polymerase III subunit beta | -0.500866812 | 1.02E-08 | 3.53E-08 |
| FQU82_RS18600 | FQU82_RS18600 | YqiA/YcfP family alpha/beta fold hydrolase | 0.859693776 | 1.05E-08 | 3.61E-08 |
| FQU82_RS10385 | FQU82_RS10385 | isochorismatase family protein | -2.406057715 | 1.05E-08 | 3.61E-08 |
| FQU82_RS13125 | FQU82_RS13125 | quinone-dependent dihydroorotate dehydrogenase | -0.686516943 | 1.12E-08 | 3.85E-08 |
| FQU82_RS05590 | FQU82_RS05590 | hypothetical protein | 1.27600681 | 1.15E-08 | 3.96E-08 |
| FQU82_RS12315 | FQU82_RS12315 | UDP-2,3-diacylglucosamine diphosphatase | -2.244800475 | 1.21E-08 | 4.14E-08 |
| Novel00234 | - | PF02800:Glyceraldehyde 3-phosphate dehydrogenase, C-terminal domain|PF00044:Glyceraldehyde 3-phosphate dehydrogenase, NAD binding domain | -1.38983327 | 1.25E-08 | 4.28E-08 |
| Novel00355 | - | PF00324:Amino acid permease | 1.961639077 | 1.27E-08 | 4.34E-08 |
| FQU82_RS14440 | FQU82_RS14440 | DUF962 domain-containing protein | 1.242638556 | 1.27E-08 | 4.35E-08 |
| FQU82_RS02945 | FQU82_RS02945 | acetate kinase | -0.520832072 | 1.28E-08 | 4.36E-08 |
| FQU82_RS04520 | FQU82_RS04520 | arginyltransferase | 0.627227685 | 1.36E-08 | 4.63E-08 |
| FQU82_RS02435 | abeM | multidrug efflux MATE transporter AbeM | -0.876667189 | 1.36E-08 | 4.65E-08 |
| FQU82_RS00905 | lldR | transcriptional regulator LldR | 1.277854415 | 1.43E-08 | 4.87E-08 |
| FQU82_RS15415 | FQU82_RS15415 | universal stress protein | 0.848856236 | 1.44E-08 | 4.89E-08 |
| FQU82_RS08450 | FQU82_RS08450 | TetR/AcrR family transcriptional regulator | 0.686465353 | 1.47E-08 | 5.00E-08 |
| FQU82_RS18965 | FQU82_RS18965 | PLP-dependent aminotransferase family protein | 0.887678454 | 1.52E-08 | 5.18E-08 |
| FQU82_RS03350 | yjgA | ribosome biogenesis factor YjgA | 0.806482223 | 1.58E-08 | 5.36E-08 |
| FQU82_RS17325 | FQU82_RS17325 | hypothetical protein | -0.598263965 | 1.76E-08 | 5.95E-08 |
| FQU82_RS04280 | ribB | 3,4-dihydroxy-2-butanone-4-phosphate synthase | -0.529119428 | 1.95E-08 | 6.60E-08 |
| Novel00277 | - | - | 2.069730971 | 1.98E-08 | 6.70E-08 |
| FQU82_RS07520 | FQU82_RS07520 | HAD-IIIA family hydrolase | -0.933202639 | 1.99E-08 | 6.73E-08 |
| FQU82_RS09385 | FQU82_RS09385 | TetR/AcrR family transcriptional regulator | -1.567119418 | 2.17E-08 | 7.32E-08 |
| FQU82_RS11705 | FQU82_RS11705 | OmpH family outer membrane protein | -0.463237136 | 2.27E-08 | 7.67E-08 |
| FQU82_RS13240 | FQU82_RS13240 | acyl-CoA dehydrogenase family protein | -0.852364712 | 2.32E-08 | 7.82E-08 |
| FQU82_RS15525 | FQU82_RS15525 | DUF1289 domain-containing protein | 1.15297499 | 2.32E-08 | 7.82E-08 |
| Novel00256 | - | PF01434:Peptidase family M41|PF06480:FtsH Extracellular|PF00004:ATPase family associated with various cellular activities (AAA)|PF17862:AAA+ lid domain|PF01728:FtsJ-like methyltransferase | 0.802904792 | 2.43E-08 | 8.16E-08 |
| FQU82_RS10220 | FQU82_RS10220 | aspartate ammonia-lyase | -0.892804348 | 2.48E-08 | 8.33E-08 |
| FQU82_RS17910 | FQU82_RS17910 | ATP-binding protein | 0.559210423 | 2.56E-08 | 8.59E-08 |
| Novel00108 | - | PF01386:Ribosomal L25p family|PF13793:N-terminal domain of ribose phosphate pyrophosphokinase|PF14572:Phosphoribosyl synthetase-associated domain | -1.325013518 | 2.59E-08 | 8.69E-08 |
| FQU82_RS08770 | cydB | cytochrome d ubiquinol oxidase subunit II | 1.106906875 | 2.62E-08 | 8.77E-08 |
| FQU82_RS19000 | dapB | 4-hydroxy-tetrahydrodipicolinate reductase | -0.735196439 | 2.70E-08 | 9.02E-08 |
| FQU82_RS13260 | FQU82_RS13260 | NAD(+) kinase | 0.512937469 | 2.75E-08 | 9.21E-08 |
| FQU82_RS12665 | FQU82_RS12665 | circularly permuted type 2 ATP-grasp protein | 0.914933831 | 2.86E-08 | 9.56E-08 |
| FQU82_RS19070 | rsmB | 16S rRNA (cytosine(967)-C(5))-methyltransferase RsmB | -0.657305693 | 2.90E-08 | 9.68E-08 |
| FQU82_RS09730 | hscA | Fe-S protein assembly chaperone HscA | -0.48222856 | 2.91E-08 | 9.69E-08 |
| FQU82_RS04390 | FQU82_RS04390 | YfhL family 4Fe-4S dicluster ferredoxin | 0.704073063 | 2.98E-08 | 9.94E-08 |
| FQU82_RS07030 | FQU82_RS07030 | O-methyltransferase | 1.004585436 | 3.00E-08 | 9.97E-08 |
| FQU82_RS16630 | FQU82_RS16630 | alpha/beta hydrolase | 1.107715867 | 3.04E-08 | 1.01E-07 |
| Novel00085 | - | PF02410:Ribosomal silencing factor during starvation | 2.410194323 | 3.12E-08 | 1.04E-07 |
| FQU82_RS15450 | FQU82_RS15450 | neutral zinc metallopeptidase | 0.708679878 | 3.33E-08 | 1.11E-07 |
| FQU82_RS10045 | recR | recombination mediator RecR | -0.850491188 | 3.37E-08 | 1.12E-07 |
| FQU82_RS16735 | pdxA | 4-hydroxythreonine-4-phosphate dehydrogenase PdxA | 0.504850043 | 3.38E-08 | 1.12E-07 |
| FQU82_RS02925 | FQU82_RS02925 | DUF2846 domain-containing protein | 1.239762723 | 3.50E-08 | 1.16E-07 |
| FQU82_RS16195 | FQU82_RS16195 | thiolase family protein | -0.569839333 | 3.52E-08 | 1.16E-07 |
| FQU82_RS14435 | thiE | thiamine phosphate synthase | -1.40609627 | 3.62E-08 | 1.20E-07 |
| FQU82_RS04385 | coaD | pantetheine-phosphate adenylyltransferase | 0.671022663 | 3.70E-08 | 1.22E-07 |
| FQU82_RS10960 | FQU82_RS10960 | TetR/AcrR family transcriptional regulator | 1.823447567 | 3.73E-08 | 1.23E-07 |
| FQU82_RS15155 | fdhD | formate dehydrogenase accessory sulfurtransferase FdhD | 1.410612622 | 3.88E-08 | 1.28E-07 |
| FQU82_RS13810 | FQU82_RS13810 | DUF962 domain-containing protein | 1.570968857 | 4.14E-08 | 1.36E-07 |
| FQU82_RS14055 | FQU82_RS14055 | DUF817 family protein | -2.895344448 | 4.38E-08 | 1.44E-07 |
| FQU82_RS19055 | FQU82_RS19055 | hypothetical protein | 0.765381051 | 4.39E-08 | 1.44E-07 |
| FQU82_RS00245 | hpt | hypoxanthine phosphoribosyltransferase | -0.812069209 | 4.50E-08 | 1.48E-07 |
| FQU82_RS06890 | FQU82_RS06890 | ATP phosphoribosyltransferase regulatory subunit | -0.649828529 | 4.53E-08 | 1.48E-07 |
| FQU82_RS11400 | ilvA | threonine ammonia-lyase, biosynthetic | -0.562549645 | 4.56E-08 | 1.50E-07 |
| FQU82_RS09835 | FQU82_RS09835 | IucA/IucC family protein | -1.116837777 | 4.60E-08 | 1.51E-07 |
| FQU82_RS13180 | FQU82_RS13180 | DUF423 domain-containing protein | -0.998661885 | 4.89E-08 | 1.60E-07 |
| FQU82_RS02365 | murI | glutamate racemase | 0.796977685 | 5.17E-08 | 1.69E-07 |
| Novel00069 | - | PF00593:TonB dependent receptor | -1.611273585 | 5.34E-08 | 1.74E-07 |
| FQU82_RS00160 | FQU82_RS00160 | ATP-binding cassette domain-containing protein | -0.673528655 | 5.37E-08 | 1.75E-07 |
| FQU82_RS17975 | FQU82_RS17975 | DMT family transporter | 1.427935823 | 5.38E-08 | 1.75E-07 |
| FQU82_RS10970 | FQU82_RS10970 | Rrf2 family transcriptional regulator | 0.955479722 | 5.49E-08 | 1.79E-07 |
| FQU82_RS03585 | hisC | histidinol-phosphate transaminase | -0.667184891 | 5.59E-08 | 1.82E-07 |
| FQU82_RS17405 | FQU82_RS17405 | hypothetical protein | 2.382729085 | 6.35E-08 | 2.06E-07 |
| FQU82_RS15380 | yhbY | ribosome assembly RNA-binding protein YhbY | -1.070005411 | 6.54E-08 | 2.12E-07 |
| FQU82_RS02195 | FQU82_RS02195 | Na+/H+ antiporter subunit C | -0.852853484 | 6.99E-08 | 2.27E-07 |
| FQU82_RS10170 | FQU82_RS10170 | GntR family transcriptional regulator | 3.007107171 | 7.03E-08 | 2.28E-07 |
| FQU82_RS04990 | FQU82_RS04990 | MarC family protein | -1.57303209 | 7.44E-08 | 2.41E-07 |
| FQU82_RS15860 | FQU82_RS15860 | Maf-like protein | -1.165616443 | 7.93E-08 | 2.56E-07 |
| FQU82_RS17315 | hemF | oxygen-dependent coproporphyrinogen oxidase | 0.384319165 | 7.94E-08 | 2.56E-07 |
| FQU82_RS01545 | FQU82_RS01545 | long-chain-fatty-acid--CoA ligase | -0.392148265 | 8.32E-08 | 2.69E-07 |
| FQU82_RS07615 | FQU82_RS07615 | D-Ala-D-Ala carboxypeptidase family metallohydrolase | -1.064957017 | 9.10E-08 | 2.94E-07 |
| FQU82_RS18810 | FQU82_RS18810 | HutD family protein | 1.169172387 | 9.14E-08 | 2.95E-07 |
| FQU82_RS01200 | FQU82_RS01200 | hypothetical protein | 0.973888895 | 9.39E-08 | 3.02E-07 |
| Novel00344 | - | - | 1.448946155 | 9.41E-08 | 3.03E-07 |
| FQU82_RS02285 | FQU82_RS02285 | magnesium transporter CorA family protein | 0.86594441 | 9.66E-08 | 3.11E-07 |
| FQU82_RS17655 | bfr | bacterioferritin | 0.411076738 | 9.70E-08 | 3.11E-07 |
| FQU82_RS04115 | FQU82_RS04115 | hypothetical protein | 1.562073167 | 1.01E-07 | 3.25E-07 |
| FQU82_RS02525 | FQU82_RS02525 | RNA pyrophosphohydrolase | 0.668721692 | 1.08E-07 | 3.45E-07 |
| FQU82_RS18915 | FQU82_RS18915 | PQQ-dependent sugar dehydrogenase | 0.972691054 | 1.14E-07 | 3.65E-07 |
| FQU82_RS01940 | FQU82_RS01940 | YMGG-like glycine zipper-containing protein | 2.561972021 | 1.14E-07 | 3.66E-07 |
| FQU82_RS04345 | FQU82_RS04345 | tetratricopeptide repeat protein | 0.768089157 | 1.22E-07 | 3.89E-07 |
| FQU82_RS18480 | murG | undecaprenyldiphospho-muramoylpentapeptide beta-N-acetylglucosaminyltransferase | -0.65916325 | 1.26E-07 | 4.02E-07 |
| FQU82_RS02235 | ubiE | bifunctional demethylmenaquinone methyltransferase/2-methoxy-6-polyprenyl-1,4-benzoquinol methylase UbiE | -0.464586454 | 1.33E-07 | 4.23E-07 |
| FQU82_RS17475 | FQU82_RS17475 | ParA family protein | 0.681887866 | 1.35E-07 | 4.31E-07 |
| FQU82_RS05925 | FQU82_RS05925 | hypothetical protein | 0.588615944 | 1.36E-07 | 4.33E-07 |
| FQU82_RS04650 | FQU82_RS04650 | SbcC/MukB-like Walker B domain-containing protein | 0.843000217 | 1.37E-07 | 4.35E-07 |
| FQU82_RS18120 | FQU82_RS18120 | MFS transporter | 1.554968465 | 1.38E-07 | 4.37E-07 |
| FQU82_RS15210 | FQU82_RS15210 | SgcJ/EcaC family oxidoreductase | 1.647397652 | 1.38E-07 | 4.38E-07 |
| FQU82_RS10215 | FQU82_RS10215 | TonB-dependent siderophore receptor | -1.405021185 | 1.41E-07 | 4.48E-07 |
| FQU82_RS07540 | FQU82_RS07540 | TolC family protein | -1.225192092 | 1.44E-07 | 4.56E-07 |
| FQU82_RS11325 | FQU82_RS11325 | alpha/beta hydrolase | 0.705533409 | 1.44E-07 | 4.57E-07 |
| FQU82_RS00225 | FQU82_RS00225 | metal/formaldehyde-sensitive transcriptional repressor | 1.734572241 | 1.45E-07 | 4.60E-07 |
| FQU82_RS03285 | cysM | cysteine synthase CysM | -0.704351996 | 1.48E-07 | 4.70E-07 |
| FQU82_RS17235 | FQU82_RS17235 | NAD(P)H-binding protein | 0.83073749 | 1.51E-07 | 4.76E-07 |
| FQU82_RS05315 | FQU82_RS05315 | SRPBCC domain-containing protein | 1.568566148 | 1.78E-07 | 5.62E-07 |
| FQU82_RS18350 | FQU82_RS18350 | mechanosensitive ion channel family protein | -0.667198765 | 1.82E-07 | 5.75E-07 |
| FQU82_RS13255 | FQU82_RS13255 | YeaC family protein | 1.118139945 | 1.87E-07 | 5.90E-07 |
| FQU82_RS18065 | FQU82_RS18065 | helix-turn-helix domain-containing protein | 1.991303859 | 1.89E-07 | 5.96E-07 |
| FQU82_RS06015 | FQU82_RS06015 | NADH:flavin oxidoreductase/NADH oxidase family protein | 0.472478849 | 1.91E-07 | 6.02E-07 |
| Novel00318 | - | PF00005:ABC transporter|PF12848:ABC transporter | -0.824220264 | 1.99E-07 | 6.26E-07 |
| Novel00188 | - | PF01266:FAD dependent oxidoreductase | 2.202297233 | 2.17E-07 | 6.82E-07 |
| FQU82_RS15275 | groL | chaperonin GroEL | -0.4327512 | 2.34E-07 | 7.33E-07 |
| FQU82_RS03660 | FQU82_RS03660 | DUF2218 domain-containing protein | 1.493483819 | 2.34E-07 | 7.34E-07 |
| FQU82_RS04940 | FQU82_RS04940 | alpha/beta fold hydrolase | 1.156141074 | 2.38E-07 | 7.45E-07 |
| FQU82_RS19050 | FQU82_RS19050 | sulfite exporter TauE/SafE family protein | -1.247389329 | 2.38E-07 | 7.46E-07 |
| FQU82_RS09825 | FQU82_RS09825 | DHA2 family efflux MFS transporter permease subunit | -1.889137117 | 2.50E-07 | 7.82E-07 |
| FQU82_RS04140 | FQU82_RS04140 | biotin--[acetyl-CoA-carboxylase] ligase | -0.718486453 | 2.61E-07 | 8.15E-07 |
| FQU82_RS01410 | FQU82_RS01410 | hypothetical protein | -2.237581184 | 2.69E-07 | 8.39E-07 |
| FQU82_RS08245 | FQU82_RS08245 | NAD-dependent succinate-semialdehyde dehydrogenase | 1.677011545 | 2.69E-07 | 8.39E-07 |
| FQU82_RS04595 | argB | acetylglutamate kinase | -0.867039145 | 2.73E-07 | 8.50E-07 |
| FQU82_RS11385 | FQU82_RS11385 | hypothetical protein | 0.909765256 | 2.74E-07 | 8.52E-07 |
| FQU82_RS18130 | FQU82_RS18130 | GFA family protein | 0.78446231 | 2.81E-07 | 8.74E-07 |
| FQU82_RS06915 | crp | cAMP-activated global transcriptional regulator CRP | -0.638582416 | 2.92E-07 | 9.07E-07 |
| FQU82_RS09230 | bioB | biotin synthase BioB | -0.533829413 | 3.06E-07 | 9.51E-07 |
| Novel00311 | - | PF01493:GXGXG motif|PF01645:Conserved region in glutamate synthase|PF00310:Glutamine amidotransferases class-II|PF04898:Glutamate synthase central domain|PF07992:Pyridine nucleotide-disulphide oxidoreductase|PF14691:Dihydroprymidine dehydrogenase domain II, 4Fe-4S cluster | 0.681093656 | 3.22E-07 | 1.00E-06 |
| FQU82_RS11340 | FQU82_RS11340 | NADPH-dependent 2,4-dienoyl-CoA reductase | -0.604816862 | 3.23E-07 | 1.00E-06 |
| FQU82_RS13930 | FQU82_RS13930 | superoxide dismutase | 0.322633946 | 3.24E-07 | 1.00E-06 |
| FQU82_RS01270 | FQU82_RS01270 | NAD-dependent malic enzyme | 0.479525876 | 3.46E-07 | 1.07E-06 |
| Novel00120 | - | PF00490:Delta-aminolevulinic acid dehydratase | 1.317607196 | 3.51E-07 | 1.08E-06 |
| Novel00155 | - | - | 3.334165862 | 3.78E-07 | 1.17E-06 |
| FQU82_RS14995 | FQU82_RS14995 | lysophospholipid acyltransferase family protein | -0.66143885 | 3.79E-07 | 1.17E-06 |
| FQU82_RS14770 | mltG | endolytic transglycosylase MltG | -0.855008822 | 3.81E-07 | 1.18E-06 |
| Novel00282 | - | PF00380:Ribosomal protein S9/S16|PF02798:Glutathione S-transferase, N-terminal domain | -0.937972162 | 3.99E-07 | 1.23E-06 |
| FQU82_RS13265 | mrcB | penicillin-binding protein 1B | -0.40937488 | 4.00E-07 | 1.23E-06 |
| FQU82_RS01760 | FQU82_RS01760 | hypothetical protein | 1.404639237 | 4.33E-07 | 1.33E-06 |
| FQU82_RS02650 | FQU82_RS02650 | SlyX family protein | 0.951872379 | 4.34E-07 | 1.33E-06 |
| Novel00337 | - | PF00478:IMP dehydrogenase / GMP reductase domain|PF00571:CBS domain | -0.934221942 | 4.41E-07 | 1.35E-06 |
| FQU82_RS04000 | FQU82_RS04000 | ferredoxin--NADP reductase | -0.870837276 | 4.65E-07 | 1.43E-06 |
| FQU82_RS01515 | FQU82_RS01515 | TIGR00730 family Rossman fold protein | 0.729244749 | 4.70E-07 | 1.44E-06 |
| FQU82_RS17385 | FQU82_RS17385 | LysR family transcriptional regulator | 1.628177917 | 4.73E-07 | 1.45E-06 |
| FQU82_RS10150 | FQU82_RS10150 | AraC family transcriptional regulator | 1.360802251 | 5.42E-07 | 1.66E-06 |
| FQU82_RS10300 | FQU82_RS10300 | 3-hydroxybutyrate dehydrogenase | 1.060263059 | 5.56E-07 | 1.70E-06 |
| FQU82_RS04145 | FQU82_RS04145 | sulfite exporter TauE/SafE family protein | -0.766546696 | 6.12E-07 | 1.87E-06 |
| FQU82_RS16310 | folC | bifunctional tetrahydrofolate synthase/dihydrofolate synthase | -0.613985967 | 6.21E-07 | 1.90E-06 |
| FQU82_RS02840 | FQU82_RS02840 | NRDE family protein | 0.843625231 | 6.22E-07 | 1.90E-06 |
| FQU82_RS16940 | FQU82_RS16940 | fumarylacetoacetate hydrolase family protein | -0.720307152 | 6.36E-07 | 1.94E-06 |
| FQU82_RS18635 | FQU82_RS18635 | DUF924 family protein | 0.836625682 | 6.37E-07 | 1.94E-06 |
| FQU82_RS01195 | FQU82_RS01195 | ParA family protein | 0.638817398 | 6.55E-07 | 1.99E-06 |
| FQU82_RS05060 | FQU82_RS05060 | non-heme iron oxygenase ferredoxin subunit | 3.787171365 | 6.60E-07 | 2.00E-06 |
| Novel00198 | - | PF00296:Luciferase-like monooxygenase | 1.326094349 | 6.78E-07 | 2.06E-06 |
| FQU82_RS02640 | FQU82_RS02640 | LysE family translocator | 0.986202933 | 7.11E-07 | 2.16E-06 |
| FQU82_RS05330 | FQU82_RS05330 | capsule assembly Wzi family protein | -0.550487027 | 7.71E-07 | 2.34E-06 |
| FQU82_RS15365 | folP | dihydropteroate synthase | -1.724907709 | 7.73E-07 | 2.34E-06 |
| Novel00338 | - | PF17831:Pyruvate dehydrogenase E1 component middle domain|PF00364:Biotin-requiring enzyme|PF00198:2-oxoacid dehydrogenases acyltransferase (catalytic domain)|PF02817:e3 binding domain | -0.686391534 | 7.74E-07 | 2.34E-06 |
| FQU82_RS04105 | FQU82_RS04105 | hypothetical protein | 0.998941042 | 8.17E-07 | 2.47E-06 |
| FQU82_RS01605 | FQU82_RS01605 | ammonium transporter | 0.537245984 | 8.87E-07 | 2.68E-06 |
| FQU82_RS04165 | ligA | NAD-dependent DNA ligase LigA | -0.666484119 | 9.14E-07 | 2.76E-06 |
| FQU82_RS18305 | FQU82_RS18305 | PAS domain-containing hybrid sensor histidine kinase/response regulator | 0.481890207 | 9.26E-07 | 2.79E-06 |
| FQU82_RS04020 | FQU82_RS04020 | hypothetical protein | 0.636700946 | 9.36E-07 | 2.82E-06 |
| FQU82_RS04055 | FQU82_RS04055 | NF038215 family lipoprotein | 3.025384929 | 1.01E-06 | 3.03E-06 |
| FQU82_RS14115 | basD | acinetobactin non-ribosomal peptide synthetase subunit BasD | -1.927877776 | 1.03E-06 | 3.10E-06 |
| FQU82_RS10900 | FQU82_RS10900 | ShlB/FhaC/HecB family hemolysin secretion/activation protein | -1.172239976 | 1.06E-06 | 3.18E-06 |
| FQU82_RS00090 | FQU82_RS00090 | tetratricopeptide repeat protein | 0.487291807 | 1.07E-06 | 3.20E-06 |
| FQU82_RS14925 | FQU82_RS14925 | NF038105 family protein | 1.643781208 | 1.07E-06 | 3.21E-06 |
| FQU82_RS04900 | FQU82_RS04900 | LysR family transcriptional regulator | 1.82667063 | 1.11E-06 | 3.34E-06 |
| Novel00217 | - | PF02777:Iron/manganese superoxide dismutases, C-terminal domain | 1.1964535 | 1.12E-06 | 3.37E-06 |
| FQU82_RS11420 | FQU82_RS11420 | insulinase family protein | 0.332840944 | 1.13E-06 | 3.37E-06 |
| FQU82_RS06980 | FQU82_RS06980 | glutathione S-transferase | 0.715515493 | 1.13E-06 | 3.38E-06 |
| Novel00315 | - | PF00749:tRNA synthetases class I (E and Q), catalytic domain | 1.293695312 | 1.21E-06 | 3.60E-06 |
| FQU82_RS11040 | FQU82_RS11040 | TorF family putative porin | 0.46131513 | 1.21E-06 | 3.62E-06 |
| FQU82_RS03340 | rapZ | RNase adapter RapZ | 0.525262233 | 1.24E-06 | 3.69E-06 |
| FQU82_RS05575 | FQU82_RS05575 | hypothetical protein | 0.854585215 | 1.27E-06 | 3.78E-06 |
| FQU82_RS11975 | FQU82_RS11975 | alpha/beta hydrolase | 0.733146409 | 1.32E-06 | 3.92E-06 |
| FQU82_RS12680 | FQU82_RS12680 | hypothetical protein | 0.64697483 | 1.38E-06 | 4.10E-06 |
| FQU82_RS04945 | FQU82_RS04945 | basic amino acid/polyamine antiporter | -0.926882491 | 1.48E-06 | 4.39E-06 |
| FQU82_RS14130 | bauB | siderophore-binding periplasmic lipoprotein BauB | -2.58326421 | 1.50E-06 | 4.44E-06 |
| FQU82_RS05565 | FQU82_RS05565 | hypothetical protein | 1.417232918 | 1.51E-06 | 4.49E-06 |
| FQU82_RS11950 | FQU82_RS11950 | MFS transporter | -2.362677563 | 1.52E-06 | 4.50E-06 |
| FQU82_RS04575 | FQU82_RS04575 | OmpA family protein | -0.360546746 | 1.52E-06 | 4.52E-06 |
| FQU82_RS04680 | FQU82_RS04680 | FAD-dependent oxidoreductase | 0.686418864 | 1.57E-06 | 4.65E-06 |
| FQU82_RS14725 | rnc | ribonuclease III | -1.059776813 | 1.62E-06 | 4.79E-06 |
| Novel00048 | - | PF00378:Enoyl-CoA hydratase/isomerase|PF02737:3-hydroxyacyl-CoA dehydrogenase, NAD binding domain|PF00725:3-hydroxyacyl-CoA dehydrogenase, C-terminal domain|PF00108:Thiolase, N-terminal domain|PF02803:Thiolase, C-terminal domain | 0.534194323 | 1.64E-06 | 4.84E-06 |
| FQU82_RS17945 | hisB | imidazoleglycerol-phosphate dehydratase HisB | -0.490337427 | 1.64E-06 | 4.84E-06 |
| FQU82_RS09685 | FQU82_RS09685 | nucleoside-diphosphate sugar epimerase | 1.319672275 | 1.65E-06 | 4.87E-06 |
| FQU82_RS12510 | FQU82_RS12510 | acyl-CoA dehydrogenase family protein | 1.799465613 | 1.68E-06 | 4.95E-06 |
| FQU82_RS05470 | gdhA | NADP-specific glutamate dehydrogenase | -0.593224822 | 1.73E-06 | 5.09E-06 |
| FQU82_RS04245 | rpmF | 50S ribosomal protein L32 | -0.724667949 | 1.77E-06 | 5.20E-06 |
| FQU82_RS03460 | proC | pyrroline-5-carboxylate reductase | -0.518060502 | 1.78E-06 | 5.23E-06 |
| FQU82_RS15935 | FQU82_RS15935 | hypothetical protein | -2.958134772 | 1.87E-06 | 5.49E-06 |
| FQU82_RS11815 | galE | UDP-glucose 4-epimerase GalE | 0.447311404 | 1.89E-06 | 5.56E-06 |
| FQU82_RS04630 | fur | ferric iron uptake transcriptional regulator | 0.580929267 | 1.92E-06 | 5.63E-06 |
| FQU82_RS03245 | FQU82_RS03245 | proton-translocating transhydrogenase family protein | -0.758225767 | 1.96E-06 | 5.73E-06 |
| FQU82_RS16950 | FQU82_RS16950 | nitroreductase family protein | 2.312509886 | 1.96E-06 | 5.73E-06 |
| FQU82_RS02990 | nagZ | beta-N-acetylhexosaminidase | -0.668338353 | 2.00E-06 | 5.85E-06 |
| FQU82_RS09050 | FQU82_RS09050 | amino acid ABC transporter substrate-binding protein | 0.461457913 | 2.08E-06 | 6.07E-06 |
| FQU82_RS03085 | orn | oligoribonuclease | -0.739121412 | 2.13E-06 | 6.24E-06 |
| FQU82_RS19085 | FQU82_RS19085 | TIGR04219 family outer membrane beta-barrel protein | -1.101279366 | 2.14E-06 | 6.25E-06 |
| FQU82_RS16355 | FQU82_RS16355 | TonB-dependent receptor | -1.248295531 | 2.15E-06 | 6.26E-06 |
| FQU82_RS11770 | argF | ornithine carbamoyltransferase | -0.474188601 | 2.19E-06 | 6.39E-06 |
| FQU82_RS04155 | smc | chromosome segregation protein SMC | -0.69341999 | 2.21E-06 | 6.44E-06 |
| FQU82_RS18315 | FQU82_RS18315 | response regulator transcription factor | 0.658546438 | 2.25E-06 | 6.54E-06 |
| FQU82_RS11785 | FQU82_RS11785 | hypothetical protein | 1.233026561 | 2.33E-06 | 6.77E-06 |
| FQU82_RS10975 | FQU82_RS10975 | FAD-dependent oxidoreductase | 1.157443833 | 2.51E-06 | 7.28E-06 |
| FQU82_RS00220 | mnmE | tRNA uridine-5-carboxymethylaminomethyl(34) synthesis GTPase MnmE | -0.694728531 | 2.51E-06 | 7.29E-06 |
| FQU82_RS17770 | FQU82_RS17770 | UDP-N-acetylmuramoyl-L-alanyl-D-glutamate--2,6-diaminopimelate ligase | -0.541231764 | 2.53E-06 | 7.34E-06 |
| FQU82_RS05020 | FQU82_RS05020 | FKBP-type peptidyl-prolyl cis-trans isomerase | -1.144390737 | 2.59E-06 | 7.51E-06 |
| FQU82_RS13455 | FQU82_RS13455 | tape measure protein | 0.958788431 | 2.64E-06 | 7.64E-06 |
| FQU82_RS14035 | folE | GTP cyclohydrolase I FolE | -0.914010132 | 2.72E-06 | 7.86E-06 |
| FQU82_RS17010 | rrtA | rhombosortase | 0.917745571 | 2.80E-06 | 8.10E-06 |
| FQU82_RS16360 | FQU82_RS16360 | cob(I)yrinic acid a,c-diamide adenosyltransferase | -1.468663803 | 2.87E-06 | 8.29E-06 |
| FQU82_RS16985 | rnr | ribonuclease R | 0.357572952 | 2.92E-06 | 8.43E-06 |
| FQU82_RS01610 | nrdR | transcriptional regulator NrdR | 0.7352379 | 3.01E-06 | 8.69E-06 |
| FQU82_RS12515 | FQU82_RS12515 | 3-hydroxyacyl-CoA dehydrogenase | 1.488382632 | 3.15E-06 | 9.08E-06 |
| FQU82_RS09410 | FQU82_RS09410 | hypothetical protein | -2.01831429 | 3.20E-06 | 9.22E-06 |
| FQU82_RS00305 | FQU82_RS00305 | lytic murein transglycosylase | -0.444164261 | 3.26E-06 | 9.37E-06 |
| FQU82_RS16400 | FQU82_RS16400 | acyl-CoA dehydrogenase C-terminal domain-containing protein | 0.312752718 | 3.29E-06 | 9.47E-06 |
| FQU82_RS11505 | FQU82_RS11505 | CBS domain-containing protein | 0.497491687 | 3.39E-06 | 9.74E-06 |
| FQU82_RS12390 | FQU82_RS12390 | DUF4198 domain-containing protein | -1.729675567 | 3.40E-06 | 9.75E-06 |
| FQU82_RS02315 | FQU82_RS02315 | primosomal protein N | 0.655481076 | 3.46E-06 | 9.91E-06 |
| FQU82_RS16700 | FQU82_RS16700 | lysophospholipid acyltransferase family protein | -0.694830767 | 3.52E-06 | 1.01E-05 |
| FQU82_RS11580 | FQU82_RS11580 | MarR family transcriptional regulator | 0.555073213 | 3.64E-06 | 1.04E-05 |
| FQU82_RS01990 | FQU82_RS01990 | hypothetical protein | 1.109349115 | 3.65E-06 | 1.04E-05 |
| FQU82_RS07660 | FQU82_RS07660 | MFS transporter | 1.817977027 | 3.69E-06 | 1.05E-05 |
| FQU82_RS03420 | FQU82_RS03420 | integration host factor subunit alpha | 0.617556886 | 3.76E-06 | 1.07E-05 |
| FQU82_RS02725 | FQU82_RS02725 | coniferyl aldehyde dehydrogenase | 0.944329178 | 3.83E-06 | 1.09E-05 |
| Novel00002 | - | PF01521:Iron-sulphur cluster biosynthesis | 3.066421685 | 4.01E-06 | 1.14E-05 |
| FQU82_RS00335 | FQU82_RS00335 | LysR family transcriptional regulator | 1.495813865 | 4.03E-06 | 1.15E-05 |
| FQU82_RS17675 | FQU82_RS17675 | TPM domain-containing protein | 1.040360386 | 4.05E-06 | 1.15E-05 |
| FQU82_RS01705 | xerD | site-specific tyrosine recombinase XerD | -0.98653475 | 4.12E-06 | 1.17E-05 |
| FQU82_RS16305 | FQU82_RS16305 | SPOR domain-containing protein | 0.461003535 | 4.15E-06 | 1.18E-05 |
| FQU82_RS03880 | FQU82_RS03880 | ribonucleotide-diphosphate reductase subunit beta | -0.325719597 | 4.21E-06 | 1.19E-05 |
| FQU82_RS14500 | icd | NADP-dependent isocitrate dehydrogenase | 0.610035946 | 4.23E-06 | 1.20E-05 |
| FQU82_RS00790 | FQU82_RS00790 | FKBP-type peptidyl-prolyl cis-trans isomerase | -0.491767988 | 4.23E-06 | 1.20E-05 |
| FQU82_RS02180 | FQU82_RS02180 | monovalent cation/H+ antiporter subunit F | -1.279956275 | 4.41E-06 | 1.25E-05 |
| Novel00249 | - | PF01012:Electron transfer flavoprotein domain|PF00766:Electron transfer flavoprotein FAD-binding domain | -0.634340217 | 4.44E-06 | 1.26E-05 |
| FQU82_RS09570 | FQU82_RS09570 | lysine exporter LysO family protein | -1.544632441 | 4.46E-06 | 1.26E-05 |
| FQU82_RS02700 | FQU82_RS02700 | hypothetical protein | 0.44253406 | 4.50E-06 | 1.27E-05 |
| FQU82_RS10625 | FQU82_RS10625 | TolC family protein | -2.223661497 | 4.62E-06 | 1.30E-05 |
| FQU82_RS11105 | feaR | transcriptional regulator FeaR | 1.902414718 | 4.70E-06 | 1.33E-05 |
| FQU82_RS03035 | ispG | flavodoxin-dependent (E)-4-hydroxy-3-methylbut-2-enyl-diphosphate synthase | -0.402390566 | 4.90E-06 | 1.38E-05 |
| FQU82_RS14985 | FQU82_RS14985 | alpha/beta hydrolase | -0.574690791 | 5.01E-06 | 1.41E-05 |
| FQU82_RS09345 | gcvH | glycine cleavage system protein GcvH | -0.99411951 | 5.14E-06 | 1.45E-05 |
| FQU82_RS03565 | ibaG | BolA family iron metabolism protein IbaG | -0.885634241 | 5.16E-06 | 1.45E-05 |
| FQU82_RS15535 | FQU82_RS15535 | YecA family protein | -0.562697135 | 5.34E-06 | 1.50E-05 |
| FQU82_RS01875 | FQU82_RS01875 | tRNA-Gly | -1.497124476 | 5.44E-06 | 1.53E-05 |
| FQU82_RS12430 | FQU82_RS12430 | outer membrane protein assembly factor | 0.467706349 | 5.50E-06 | 1.54E-05 |
| Novel00260 | - | PF01127:Succinate dehydrogenase/Fumarate reductase transmembrane subunit|PF13183:4Fe-4S dicluster domain|PF13085:2Fe-2S iron-sulfur cluster binding domain|PF02910:Fumarate reductase flavoprotein C-term|PF00890:FAD binding domain | -0.565113783 | 5.56E-06 | 1.56E-05 |
| FQU82_RS02360 | FQU82_RS02360 | DUF1285 domain-containing protein | 0.664055919 | 5.62E-06 | 1.58E-05 |
| FQU82_RS15970 | FQU82_RS15970 | YdcF family protein | -2.579632362 | 5.82E-06 | 1.63E-05 |
| FQU82_RS08710 | mdcA | malonate decarboxylase subunit alpha | 2.746327009 | 6.09E-06 | 1.70E-05 |
| FQU82_RS16385 | FQU82_RS16385 | response regulator | 0.559297834 | 6.28E-06 | 1.75E-05 |
| FQU82_RS10195 | FQU82_RS10195 | ABC transporter ATP-binding protein | 2.431880889 | 6.30E-06 | 1.76E-05 |
| FQU82_RS03785 | FQU82_RS03785 | LysR substrate-binding domain-containing protein | 1.277532449 | 6.31E-06 | 1.76E-05 |
| FQU82_RS11525 | FQU82_RS11525 | LysR family transcriptional regulator | 0.745663972 | 6.50E-06 | 1.81E-05 |
| FQU82_RS02270 | FQU82_RS02270 | EstA family serine hydrolase | 1.193320558 | 6.54E-06 | 1.82E-05 |
| FQU82_RS02060 | FQU82_RS02060 | IclR family transcriptional regulator C-terminal domain-containing protein | 0.690753677 | 6.85E-06 | 1.91E-05 |
| FQU82_RS07125 | FQU82_RS07125 | LysE family transporter | -1.415387011 | 6.96E-06 | 1.94E-05 |
| FQU82_RS14420 | FQU82_RS14420 | RluA family pseudouridine synthase | -0.866237728 | 7.13E-06 | 1.98E-05 |
| FQU82_RS09620 | FQU82_RS09620 | TonB-dependent receptor | 0.993941153 | 7.19E-06 | 2.00E-05 |
| FQU82_RS14765 | tmk | dTMP kinase | -1.093638876 | 7.32E-06 | 2.03E-05 |
| FQU82_RS17865 | FQU82_RS17865 | hypothetical protein | -0.737372873 | 7.41E-06 | 2.05E-05 |
| FQU82_RS04530 | trxB | thioredoxin-disulfide reductase | -0.373161728 | 7.51E-06 | 2.08E-05 |
| FQU82_RS17925 | FQU82_RS17925 | GNAT family acetyltransferase | 1.344512772 | 7.70E-06 | 2.13E-05 |
| Novel00157 | - | PF02773:S-adenosylmethionine synthetase, C-terminal domain|PF02772:S-adenosylmethionine synthetase, central domain|PF00438:S-adenosylmethionine synthetase, N-terminal domain | -1.772483446 | 8.05E-06 | 2.23E-05 |
| FQU82_RS01585 | FQU82_RS01585 | PLP-dependent aminotransferase family protein | 0.854870681 | 8.18E-06 | 2.26E-05 |
| Novel00246 | - | PF08281:Sigma-70, region 4 | 2.08537663 | 8.51E-06 | 2.35E-05 |
| Novel00186 | - | PF01765:Ribosome recycling factor | -1.643054638 | 8.54E-06 | 2.36E-05 |
| FQU82_RS08520 | katE | catalase HPII | -0.511676813 | 8.73E-06 | 2.41E-05 |
| FQU82_RS09640 | FQU82_RS09640 | M3 family metallopeptidase | -0.670211427 | 8.86E-06 | 2.44E-05 |
| FQU82_RS01295 | FQU82_RS01295 | transcriptional repressor | 0.791983814 | 8.87E-06 | 2.44E-05 |
| FQU82_RS15560 | FQU82_RS15560 | hypothetical protein | 0.427224565 | 9.06E-06 | 2.49E-05 |
| FQU82_RS13150 | FQU82_RS13150 | nitroreductase | -0.765056162 | 9.07E-06 | 2.49E-05 |
| FQU82_RS09965 | FQU82_RS09965 | ABC transporter permease | 1.735391805 | 9.09E-06 | 2.50E-05 |
| FQU82_RS16765 | FQU82_RS16765 | acetyl/propionyl/methylcrotonyl-CoA carboxylase subunit alpha | 0.898677073 | 9.33E-06 | 2.56E-05 |
| FQU82_RS16485 | FQU82_RS16485 | glycosyltransferase family 2 protein | -0.828413065 | 9.66E-06 | 2.65E-05 |
| FQU82_RS16140 | FQU82_RS16140 | peptidylprolyl isomerase | 0.870707009 | 9.94E-06 | 2.72E-05 |
| FQU82_RS02445 | hemW | radical SAM family heme chaperone HemW | 0.597348683 | 1.04E-05 | 2.84E-05 |
| FQU82_RS02880 | FQU82_RS02880 | tetratricopeptide repeat protein | 0.327834696 | 1.13E-05 | 3.10E-05 |
| FQU82_RS09075 | FQU82_RS09075 | Lrp/AsnC family transcriptional regulator | 0.784914625 | 1.15E-05 | 3.14E-05 |
| Novel00053 | - | PF13591:MerR HTH family regulatory protein | 2.29989009 | 1.17E-05 | 3.21E-05 |
| FQU82_RS08220 | FQU82_RS08220 | LLM class flavin-dependent oxidoreductase | 1.294485313 | 1.21E-05 | 3.31E-05 |
| FQU82_RS17745 | ponA | penicillin-binding protein PBP1a | -0.36152332 | 1.22E-05 | 3.34E-05 |
| FQU82_RS16755 | FQU82_RS16755 | ClpXP protease specificity-enhancing factor | 0.482748514 | 1.27E-05 | 3.47E-05 |
| FQU82_RS08380 | FQU82_RS08380 | 3-oxoacid CoA-transferase subunit A | -1.542060311 | 1.31E-05 | 3.58E-05 |
| FQU82_RS00475 | FQU82_RS00475 | YciK family oxidoreductase | 0.316325669 | 1.35E-05 | 3.68E-05 |
| FQU82_RS12865 | FQU82_RS12865 | paraquat-inducible protein A | 1.122999324 | 1.38E-05 | 3.76E-05 |
| FQU82_RS08455 | FQU82_RS08455 | AMP-binding protein | 0.544840502 | 1.40E-05 | 3.80E-05 |
| FQU82_RS02530 | FQU82_RS02530 | HAD family hydrolase | -0.776970383 | 1.40E-05 | 3.81E-05 |
| FQU82_RS10200 | FQU82_RS10200 | HEAT repeat domain-containing protein | 2.294319846 | 1.40E-05 | 3.81E-05 |
| FQU82_RS03525 | FQU82_RS03525 | VacJ family lipoprotein | 0.438295351 | 1.47E-05 | 3.99E-05 |
| FQU82_RS10340 | FQU82_RS10340 | AraC family transcriptional regulator | 1.75022376 | 1.49E-05 | 4.03E-05 |
| FQU82_RS08925 | FQU82_RS08925 | dicarboxylate/amino acid:cation symporter | -0.830951796 | 1.52E-05 | 4.11E-05 |
| Novel00092 | - | PF00085:Thioredoxin | 1.334452506 | 1.54E-05 | 4.17E-05 |
| FQU82_RS16935 | FQU82_RS16935 | helix-turn-helix transcriptional regulator | 1.357419474 | 1.56E-05 | 4.22E-05 |
| FQU82_RS04180 | FQU82_RS04180 | trehalose-6-phosphate synthase | -0.706117231 | 1.57E-05 | 4.24E-05 |
| FQU82_RS17995 | hisF | imidazole glycerol phosphate synthase subunit HisF | -0.446541816 | 1.59E-05 | 4.30E-05 |
| FQU82_RS15780 | FQU82_RS15780 | amino acid permease | -0.302318777 | 1.60E-05 | 4.31E-05 |
| FQU82_RS13870 | lipA | lipoyl synthase | 0.332384281 | 1.62E-05 | 4.37E-05 |
| Novel00213 | - | PF00464:Serine hydroxymethyltransferase | -0.97886156 | 1.64E-05 | 4.43E-05 |
| FQU82_RS02875 | rdgB | RdgB/HAM1 family non-canonical purine NTP pyrophosphatase | -1.045148738 | 1.65E-05 | 4.45E-05 |
| FQU82_RS03640 | tsaB | tRNA (adenosine(37)-N6)-threonylcarbamoyltransferase complex dimerization subunit type 1 TsaB | -1.030738009 | 1.67E-05 | 4.50E-05 |
| FQU82_RS06030 | rnt | ribonuclease T | -0.497301788 | 1.68E-05 | 4.52E-05 |
| FQU82_RS18185 | FQU82_RS18185 | AzlC family ABC transporter permease | -0.682847013 | 1.70E-05 | 4.56E-05 |
| Novel00364 | - | PF00311:Phosphoenolpyruvate carboxylase | 1.276982884 | 1.73E-05 | 4.63E-05 |
| FQU82_RS15090 | FQU82_RS15090 | tyrosine recombinase XerC | 0.661317577 | 1.73E-05 | 4.63E-05 |
| FQU82_RS08030 | abaF | fosfomycin efflux MFS transporter AbaF | 5.502304979 | 1.76E-05 | 4.71E-05 |
| FQU82_RS04480 | FQU82_RS04480 | EcsC family protein | 0.362730396 | 1.78E-05 | 4.76E-05 |
| FQU82_RS03595 | FQU82_RS03595 | hypothetical protein | 1.639005778 | 1.82E-05 | 4.85E-05 |
| FQU82_RS10540 | FQU82_RS10540 | AraC family transcriptional regulator ligand-binding domain-containing protein | 1.80552528 | 1.83E-05 | 4.88E-05 |
| FQU82_RS08255 | FQU82_RS08255 | LysR family transcriptional regulator | 1.060950599 | 1.85E-05 | 4.94E-05 |
| Novel00050 | - | PF02622:Uncharacterized ACR, COG1678|PF03652:Holliday junction resolvase | 1.140730723 | 1.95E-05 | 5.21E-05 |
| FQU82_RS17270 | FQU82_RS17270 | ABC transporter substrate-binding protein | -0.347809119 | 2.01E-05 | 5.35E-05 |
| FQU82_RS17700 | gltB | glutamate synthase large subunit | 0.355924268 | 2.02E-05 | 5.39E-05 |
| FQU82_RS11480 | ftsY | signal recognition particle-docking protein FtsY | -0.485588102 | 2.04E-05 | 5.44E-05 |
| FQU82_RS03650 | FQU82_RS03650 | C13 family peptidase | -0.535588112 | 2.07E-05 | 5.51E-05 |
| FQU82_RS09635 | FQU82_RS09635 | ABC transporter substrate-binding protein | -0.798159577 | 2.28E-05 | 6.07E-05 |
| FQU82_RS05035 | pgaD | poly-beta-1,6-N-acetyl-D-glucosamine biosynthesis protein PgaD | -0.97372034 | 2.31E-05 | 6.13E-05 |
| FQU82_RS11870 | FQU82_RS11870 | MoaD/ThiS family protein | 1.069721999 | 2.34E-05 | 6.22E-05 |
| FQU82_RS16620 | FQU82_RS16620 | acyl-CoA thioesterase | 0.515155679 | 2.37E-05 | 6.28E-05 |
| FQU82_RS00865 | FQU82_RS00865 | glycosyltransferase | -2.130582685 | 2.43E-05 | 6.44E-05 |
| Novel00320 | - | PF12836:Helix-hairpin-helix motif | -2.027314161 | 2.50E-05 | 6.63E-05 |
| Novel00172 | - | PF02082:Iron-dependent Transcriptional regulator|PF12706:Beta-lactamase superfamily domain | 2.432140153 | 2.52E-05 | 6.68E-05 |
| FQU82_RS12465 | kdpC | potassium-transporting ATPase subunit KdpC | 1.616869011 | 2.54E-05 | 6.73E-05 |
| FQU82_RS14570 | FQU82_RS14570 | hypothetical protein | 0.599408643 | 2.61E-05 | 6.89E-05 |
| FQU82_RS04125 | ccsA | cytochrome c biogenesis protein CcsA | 0.766687249 | 2.61E-05 | 6.90E-05 |
| FQU82_RS03010 | iscX | Fe-S cluster assembly protein IscX | -0.638912425 | 2.62E-05 | 6.91E-05 |
| Novel00181 | - | PF00881:Nitroreductase family | 2.984676311 | 2.62E-05 | 6.91E-05 |
| FQU82_RS01860 | trpE | anthranilate synthase component I | 0.471472091 | 2.64E-05 | 6.95E-05 |
| FQU82_RS16780 | FQU82_RS16780 | carboxyl transferase domain-containing protein | 1.127468819 | 2.69E-05 | 7.07E-05 |
| FQU82_RS12165 | FQU82_RS12165 | hypothetical protein | 1.273907256 | 2.69E-05 | 7.09E-05 |
| FQU82_RS16225 | FQU82_RS16225 | carbohydrate porin | -0.302929416 | 2.74E-05 | 7.21E-05 |
| FQU82_RS03995 | gigC | LysR family transcriptional regulator GigC | -0.588632899 | 2.82E-05 | 7.40E-05 |
| FQU82_RS04035 | FQU82_RS04035 | HlyD family secretion protein | 0.666219092 | 2.83E-05 | 7.44E-05 |
| FQU82_RS08110 | FQU82_RS08110 | DapH/DapD/GlmU-related protein | 1.060490217 | 3.10E-05 | 8.14E-05 |
| FQU82_RS17265 | FQU82_RS17265 | STAS domain-containing protein | -0.4871231 | 3.14E-05 | 8.24E-05 |
| FQU82_RS18920 | FQU82_RS18920 | YcgN family cysteine cluster protein | 0.95821448 | 3.15E-05 | 8.25E-05 |
| FQU82_RS08475 | FQU82_RS08475 | LysR family transcriptional regulator | 1.58819549 | 3.15E-05 | 8.25E-05 |
| Novel00161 | - | PF00162:Phosphoglycerate kinase | -2.499973437 | 3.23E-05 | 8.46E-05 |
| FQU82_RS04010 | FQU82_RS04010 | hypothetical protein | 0.615265393 | 3.28E-05 | 8.57E-05 |
| Novel00211 | - | - | 3.564898012 | 3.60E-05 | 9.38E-05 |
| FQU82_RS10640 | FQU82_RS10640 | DHA2 family efflux MFS transporter permease subunit | -2.042471244 | 3.60E-05 | 9.38E-05 |
| Novel00124 | - | PF01106:NifU-like domain|PF07715:TonB-dependent Receptor Plug Domain | 1.807499401 | 3.60E-05 | 9.39E-05 |
| FQU82_RS08605 | FQU82_RS08605 | cysteine ABC transporter substrate-binding protein | 0.597422482 | 3.66E-05 | 9.54E-05 |
| FQU82_RS04295 | FQU82_RS04295 | aspartate 1-decarboxylase | 0.441315314 | 3.83E-05 | 9.98E-05 |
| FQU82_RS18465 | FQU82_RS18465 | cell division protein FtsQ/DivIB | -0.451187329 | 3.88E-05 | 0.0001009 |
| FQU82_RS03255 | FQU82_RS03255 | SDR family NAD(P)-dependent oxidoreductase | 0.768502743 | 3.95E-05 | 0.0001027 |
| Novel00131 | - | - | 1.450754729 | 4.05E-05 | 0.0001053 |
| FQU82_RS15755 | FQU82_RS15755 | NfeD family protein | 0.552604359 | 4.08E-05 | 0.0001059 |
| FQU82_RS09650 | FQU82_RS09650 | ABC transporter permease | -1.094431574 | 4.13E-05 | 0.0001073 |
| FQU82_RS15715 | FQU82_RS15715 | phosphatase PAP2 family protein | -1.995498151 | 4.18E-05 | 0.0001084 |
| FQU82_RS10025 | FQU82_RS10025 | alpha/beta fold hydrolase | 0.847346545 | 4.32E-05 | 0.0001119 |
| FQU82_RS05675 | FQU82_RS05675 | hypothetical protein | 1.511759251 | 4.36E-05 | 0.0001129 |
| FQU82_RS16925 | FQU82_RS16925 | YihY family inner membrane protein | -0.506404592 | 4.59E-05 | 0.0001187 |
| FQU82_RS13990 | FQU82_RS13990 | aminodeoxychorismate/anthranilate synthase component II | 0.529230796 | 4.63E-05 | 0.0001196 |
| FQU82_RS16345 | trpB | tryptophan synthase subunit beta | -0.488605277 | 4.74E-05 | 0.0001224 |
| Novel00086 | - | PF01261:Xylose isomerase-like TIM barrel | 2.741800663 | 4.77E-05 | 0.0001232 |
| Novel00029 | - | PF07690:Major Facilitator Superfamily | 1.461445122 | 4.80E-05 | 0.0001237 |
| FQU82_RS03800 | FQU82_RS03800 | LysR family transcriptional regulator | 1.34444 | 4.97E-05 | 0.0001281 |
| FQU82_RS04130 | ffh | signal recognition particle protein | -0.334487322 | 4.99E-05 | 0.0001286 |
| FQU82_RS04375 | FQU82_RS04375 | flavodoxin family protein | 0.7027637 | 5.10E-05 | 0.0001312 |
| FQU82_RS01210 | FQU82_RS01210 | DedA family protein | -0.90571098 | 5.11E-05 | 0.0001315 |
| FQU82_RS01220 | FQU82_RS01220 | DUF445 family protein | 0.527789988 | 5.15E-05 | 0.0001324 |
| FQU82_RS05340 | cysD | sulfate adenylyltransferase subunit CysD | 0.269720786 | 5.21E-05 | 0.0001338 |
| Novel00035 | - | PF00072:Response regulator receiver domain | 1.56688839 | 5.22E-05 | 0.0001339 |
| FQU82_RS09980 | FQU82_RS09980 | YidB family protein | -0.508454985 | 5.24E-05 | 0.0001343 |
| FQU82_RS01190 | FQU82_RS01190 | SirB2 family protein | 0.865757228 | 5.24E-05 | 0.0001344 |
| Novel00070 | - | PF00574:Clp protease|PF00254:FKBP-type peptidyl-prolyl cis-trans isomerase|PF05698:Bacterial trigger factor protein (TF) C-terminus | -0.916444532 | 5.27E-05 | 0.000135 |
| FQU82_RS12855 | FQU82_RS12855 | MlaD family protein | 0.502913277 | 5.44E-05 | 0.0001392 |
| FQU82_RS03090 | FQU82_RS03090 | Bax inhibitor-1/YccA family protein | -0.403869238 | 5.45E-05 | 0.0001394 |
| FQU82_RS11485 | FQU82_RS11485 | hypothetical protein | 1.000841995 | 5.48E-05 | 0.0001401 |
| FQU82_RS18910 | FQU82_RS18910 | replication-associated recombination protein A | 0.580022533 | 5.58E-05 | 0.0001425 |
| Novel00252 | - | - | 2.943041148 | 5.62E-05 | 0.0001434 |
| FQU82_RS08440 | FQU82_RS08440 | carboxyl transferase domain-containing protein | -0.417682219 | 5.65E-05 | 0.000144 |
| FQU82_RS06945 | FQU82_RS06945 | hypothetical protein | 1.21862725 | 5.68E-05 | 0.0001447 |
| FQU82_RS09445 | FQU82_RS09445 | ParA family protein | -0.707792377 | 5.69E-05 | 0.000145 |
| FQU82_RS13130 | gspM | type II secretion system protein GspM | -1.252268512 | 6.06E-05 | 0.0001541 |
| FQU82_RS04525 | aat | leucyl/phenylalanyl-tRNA--protein transferase | 0.959966087 | 6.30E-05 | 0.0001601 |
| FQU82_RS13185 | thiS | sulfur carrier protein ThiS | -1.067716494 | 6.50E-05 | 0.0001651 |
| FQU82_RS14400 | FQU82_RS14400 | ferredoxin reductase | -0.851723107 | 6.62E-05 | 0.0001681 |
| FQU82_RS11335 | FQU82_RS11335 | nuclear transport factor 2 family protein | 1.148693255 | 6.86E-05 | 0.0001741 |
| FQU82_RS02395 | FQU82_RS02395 | TIGR03862 family flavoprotein | -1.39884511 | 6.90E-05 | 0.000175 |
| FQU82_RS14105 | basF | acinetobactin biosynthesis bifunctional isochorismatase/aryl carrier protein BasF | -2.22388081 | 6.99E-05 | 0.000177 |
| FQU82_RS03470 | polA | DNA polymerase I | -0.307740667 | 6.99E-05 | 0.000177 |
| FQU82_RS10980 | FQU82_RS10980 | type 1 glutamine amidotransferase domain-containing protein | 0.655263213 | 7.12E-05 | 0.00018 |
| FQU82_RS07015 | FQU82_RS07015 | hypothetical protein | 0.426928303 | 7.23E-05 | 0.0001828 |
| FQU82_RS10365 | modA | molybdate ABC transporter substrate-binding protein | 1.173973502 | 7.25E-05 | 0.000183 |
| FQU82_RS07530 | lptA | lipopolysaccharide transport periplasmic protein LptA | -0.415438227 | 7.40E-05 | 0.0001868 |
| FQU82_RS00155 | FQU82_RS00155 | DUF6091 family protein | 1.863473419 | 7.75E-05 | 0.0001955 |
| Novel00090 | - | PF00453:Ribosomal protein L20|PF08719:NADAR domain | -1.220665997 | 7.85E-05 | 0.0001978 |
| FQU82_RS18730 | FQU82_RS18730 | hypothetical protein | 0.310034736 | 7.89E-05 | 0.0001986 |
| FQU82_RS08390 | dcaP | outer membrane trimeric porin-like protein DcaP | -2.071562117 | 7.96E-05 | 0.0002005 |
| Novel00261 | - | - | 3.479186248 | 8.07E-05 | 0.0002031 |
| FQU82_RS18990 | FQU82_RS18990 | MFS transporter | 0.57805108 | 8.26E-05 | 0.0002077 |
| FQU82_RS07665 | FQU82_RS07665 | DUF2147 domain-containing protein | 1.496999257 | 8.30E-05 | 0.0002086 |
| FQU82_RS17950 | hisH | imidazole glycerol phosphate synthase subunit HisH | -0.61217406 | 8.38E-05 | 0.0002104 |
| FQU82_RS03615 | filE | putative pilus assembly protein FilE | 1.145338572 | 8.39E-05 | 0.0002105 |
| FQU82_RS01800 | FQU82_RS01800 | LytTR family DNA-binding domain-containing protein | 0.605837947 | 8.45E-05 | 0.0002119 |
| FQU82_RS14595 | FQU82_RS14595 | HAD-IB family hydrolase | 0.972451395 | 8.48E-05 | 0.0002124 |
| FQU82_RS08105 | paaX | phenylacetic acid degradation operon negative regulatory protein PaaX | 0.587672468 | 8.64E-05 | 0.0002163 |
| FQU82_RS12565 | pgaD | poly-beta-1,6-N-acetyl-D-glucosamine biosynthesis protein PgaD | 2.248618478 | 8.71E-05 | 0.0002179 |
| FQU82_RS18355 | FQU82_RS18355 | 16S rRNA (uracil(1498)-N(3))-methyltransferase | -1.093569574 | 8.72E-05 | 0.0002181 |
| FQU82_RS04015 | FQU82_RS04015 | mechanosensitive ion channel | 0.8508794 | 8.83E-05 | 0.0002207 |
| FQU82_RS09520 | FQU82_RS09520 | 6-carboxytetrahydropterin synthase | 0.438959689 | 8.87E-05 | 0.0002215 |
| FQU82_RS16095 | FQU82_RS16095 | SRPBCC family protein | 0.929483091 | 8.99E-05 | 0.0002243 |
| FQU82_RS18535 | FQU82_RS18535 | transporter | -0.377024897 | 9.03E-05 | 0.000225 |
| FQU82_RS12090 | mgtA | magnesium-translocating P-type ATPase | -0.910658565 | 9.18E-05 | 0.0002286 |
| FQU82_RS14880 | ruvA | Holliday junction branch migration protein RuvA | -0.512052061 | 9.19E-05 | 0.0002287 |
| FQU82_RS07725 | FQU82_RS07725 | 5-oxoprolinase subunit PxpA | -0.440357527 | 9.19E-05 | 0.0002287 |
| FQU82_RS07680 | FQU82_RS07680 | 3-hydroxyacyl-CoA dehydrogenase NAD-binding domain-containing protein | 0.987278965 | 9.29E-05 | 0.000231 |
| FQU82_RS02100 | FQU82_RS02100 | pyrimidine/purine nucleoside phosphorylase | -0.556815256 | 9.34E-05 | 0.0002321 |
| FQU82_RS01415 | FQU82_RS01415 | oxygenase MpaB family protein | -1.304922943 | 9.44E-05 | 0.0002343 |
| FQU82_RS11985 | murB | UDP-N-acetylmuramate dehydrogenase | -0.791404336 | 9.57E-05 | 0.0002374 |
| FQU82_RS02665 | FQU82_RS02665 | hypothetical protein | 0.432692942 | 9.61E-05 | 0.0002382 |
| Novel00216 | - | PF05221:S-adenosyl-L-homocysteine hydrolase|PF00670:S-adenosyl-L-homocysteine hydrolase, NAD binding domain|PF02219:Methylenetetrahydrofolate reductase | -1.001558486 | 9.67E-05 | 0.0002396 |
| FQU82_RS16845 | pssA | CDP-diacylglycerol--serine O-phosphatidyltransferase | -0.412834089 | 9.72E-05 | 0.0002407 |
| FQU82_RS18175 | FQU82_RS18175 | amino acid permease | 0.918451403 | 9.94E-05 | 0.000246 |
| FQU82_RS11590 | FQU82_RS11590 | GGDEF domain-containing protein | 0.639473553 | 0.0001019 | 0.0002519 |
| FQU82_RS13360 | FQU82_RS13360 | ABC transporter substrate-binding protein | 0.972698157 | 0.0001029 | 0.0002542 |
| FQU82_RS14600 | proB | glutamate 5-kinase | -0.397141597 | 0.0001062 | 0.0002621 |
| FQU82_RS16405 | FQU82_RS16405 | acyl-CoA dehydrogenase C-terminal domain-containing protein | -0.285452573 | 0.0001124 | 0.0002771 |
| FQU82_RS08005 | FQU82_RS08005 | 4-hydroxyproline epimerase | 2.211354352 | 0.0001149 | 0.0002833 |
| FQU82_RS10030 | FQU82_RS10030 | O-succinylhomoserine sulfhydrylase | -0.452788059 | 0.0001185 | 0.0002919 |
| Novel00177 | - | PF00793:DAHP synthetase I family | 1.85573126 | 0.0001196 | 0.0002943 |
| FQU82_RS11390 | FQU82_RS11390 | SprT family zinc-dependent metalloprotease | -0.843099206 | 0.0001224 | 0.0003011 |
| FQU82_RS17285 | FQU82_RS17285 | ABC transporter ATP-binding protein | -0.422976214 | 0.0001241 | 0.0003051 |
| FQU82_RS07140 | xseA | exodeoxyribonuclease VII large subunit | 0.766627805 | 0.000125 | 0.000307 |
| FQU82_RS12385 | FQU82_RS12385 | LysR family transcriptional regulator | 0.636567346 | 0.0001252 | 0.0003073 |
| FQU82_RS09275 | FQU82_RS09275 | hypothetical protein | 0.580224682 | 0.0001274 | 0.0003125 |
| FQU82_RS11170 | FQU82_RS11170 | SDR family oxidoreductase | 0.633077783 | 0.0001308 | 0.0003206 |
| FQU82_RS02245 | FQU82_RS02245 | polyphosphate--AMP phosphotransferase | 0.622434432 | 0.0001311 | 0.0003212 |
| FQU82_RS08345 | FQU82_RS08345 | IclR family transcriptional regulator | -1.170060155 | 0.0001327 | 0.0003248 |
| FQU82_RS01355 | FQU82_RS01355 | glutathione peroxidase | 0.399047034 | 0.0001333 | 0.0003261 |
| FQU82_RS00775 | nadC | carboxylating nicotinate-nucleotide diphosphorylase | -0.703863731 | 0.0001335 | 0.0003262 |
| FQU82_RS00400 | FQU82_RS00400 | peptidylprolyl isomerase | -0.394151776 | 0.0001336 | 0.0003262 |
| FQU82_RS00415 | ribF | bifunctional riboflavin kinase/FAD synthetase | 0.491738628 | 0.0001336 | 0.0003262 |
| FQU82_RS15445 | FQU82_RS15445 | cation:proton antiporter | -0.681047542 | 0.0001386 | 0.0003382 |
| FQU82_RS02250 | FQU82_RS02250 | hypothetical protein | 0.403199263 | 0.0001407 | 0.0003431 |
| FQU82_RS13175 | rpoH | RNA polymerase sigma factor RpoH | 0.294099546 | 0.000141 | 0.0003436 |
| FQU82_RS04225 | FQU82_RS04225 | segregation/condensation protein A | -0.774460681 | 0.0001452 | 0.0003533 |
| FQU82_RS06905 | FQU82_RS06905 | M48 family metallopeptidase | -0.358474689 | 0.0001452 | 0.0003533 |
| FQU82_RS01770 | FQU82_RS01770 | EamA family transporter | 2.936489693 | 0.0001453 | 0.0003533 |
| Novel00016 | - | - | 5.004715781 | 0.0001458 | 0.0003543 |
| FQU82_RS08905 | cysE | serine O-acetyltransferase | 0.479301928 | 0.0001482 | 0.0003599 |
| Novel00121 | - | PF13437:HlyD family secretion protein|PF07690:Major Facilitator Superfamily | 1.108704804 | 0.0001486 | 0.0003605 |
| FQU82_RS13395 | FQU82_RS13395 | alpha/beta fold hydrolase | 1.145970491 | 0.0001488 | 0.0003607 |
| FQU82_RS02390 | FQU82_RS02390 | SAM-dependent methyltransferase | -1.828840213 | 0.0001512 | 0.0003664 |
| FQU82_RS14050 | FQU82_RS14050 | DUF3015 family protein | -0.332354611 | 0.0001523 | 0.0003687 |
| FQU82_RS15675 | FQU82_RS15675 | hypothetical protein | 0.926944301 | 0.0001529 | 0.0003698 |
| FQU82_RS07910 | tssH | type VI secretion system ATPase TssH | 0.681267922 | 0.000153 | 0.0003698 |
| FQU82_RS03905 | bfmS | sensor histidine kinase BfmS | -0.322340767 | 0.0001539 | 0.0003718 |
| FQU82_RS13400 | FQU82_RS13400 | DUF3820 family protein | -1.475961577 | 0.0001612 | 0.0003891 |
| FQU82_RS02055 | FQU82_RS02055 | alpha-ketoglutarate-dependent dioxygenase AlkB | 0.786090315 | 0.0001639 | 0.0003955 |
| FQU82_RS04790 | FQU82_RS04790 | nucleoid-associated protein | -0.686874724 | 0.000166 | 0.0004003 |
| FQU82_RS02385 | FQU82_RS02385 | DNA gyrase inhibitor YacG | 0.583983296 | 0.0001688 | 0.0004069 |
| Novel00167 | - | PF02525:Flavodoxin-like fold | 3.712041565 | 0.0001747 | 0.0004207 |
| FQU82_RS00470 | FQU82_RS00470 | RcnB family protein | 0.457770474 | 0.0001761 | 0.0004237 |
| FQU82_RS14965 | hda | DnaA regulatory inactivator Had | -0.471152822 | 0.0001793 | 0.0004313 |
| FQU82_RS14135 | bauE | ferric acinetobactin ABC transporter ATP-binding protein BauE | -3.326784998 | 0.0001818 | 0.0004368 |
| FQU82_RS18110 | FQU82_RS18110 | DMT family transporter | -1.857587939 | 0.0001867 | 0.0004485 |
| FQU82_RS00150 | FQU82_RS00150 | DUF6091 family protein | 0.398930008 | 0.000187 | 0.0004489 |
| FQU82_RS17190 | FQU82_RS17190 | DUF853 family protein | 0.474893825 | 0.0001897 | 0.0004549 |
| FQU82_RS08010 | FQU82_RS08010 | FAD-dependent oxidoreductase | 3.066871082 | 0.0001905 | 0.0004565 |
| FQU82_RS05215 | FQU82_RS05215 | formylglycine-generating enzyme family protein | 2.033859217 | 0.0001954 | 0.0004681 |
| FQU82_RS17535 | FQU82_RS17535 | EamA family transporter | -0.956555128 | 0.0001958 | 0.0004686 |
| FQU82_RS01965 | FQU82_RS01965 | lysozyme inhibitor LprI family protein | -0.701122402 | 0.0002012 | 0.0004812 |
| Novel00299 | - | PF02092:Glycyl-tRNA synthetase beta subunit | -1.531957875 | 0.0002041 | 0.0004879 |
| FQU82_RS11540 | FQU82_RS11540 | heme-binding protein | 0.47637204 | 0.0002068 | 0.0004939 |
| FQU82_RS15845 | FQU82_RS15845 | wax ester/triacylglycerol synthase family O-acyltransferase | 0.362023628 | 0.000212 | 0.0005061 |
| Novel00067 | - | PF08238:Sel1 repeat|PF07021:Methionine biosynthesis protein MetW | 0.765875943 | 0.0002145 | 0.0005117 |
| Novel00152 | - | PF00155:Aminotransferase class I and II | 1.342958586 | 0.0002187 | 0.0005214 |
| FQU82_RS11330 | FQU82_RS11330 | SGNH/GDSL hydrolase family protein | 0.820245493 | 0.0002243 | 0.0005343 |
| FQU82_RS07535 | lptB | LPS export ABC transporter ATP-binding protein | -0.430886495 | 0.0002282 | 0.0005434 |
| FQU82_RS15175 | FQU82_RS15175 | SDR family oxidoreductase | 0.698028125 | 0.0002298 | 0.0005467 |
| FQU82_RS07875 | tssF | type VI secretion system baseplate subunit TssF | 1.483252276 | 0.00023 | 0.0005469 |
| FQU82_RS12745 | FQU82_RS12745 | MFS transporter | -1.30606746 | 0.0002364 | 0.0005616 |
| FQU82_RS16540 | FQU82_RS16540 | LemA family protein | -1.007664297 | 0.0002381 | 0.0005653 |
| FQU82_RS14885 | ruvB | Holliday junction branch migration DNA helicase RuvB | -0.403753171 | 0.0002384 | 0.0005657 |
| FQU82_RS07115 | FQU82_RS07115 | four-helix bundle copper-binding protein | 0.878823239 | 0.0002423 | 0.0005745 |
| Novel00123 | - | PF06039:Malate:quinone oxidoreductase (Mqo) | -0.864520089 | 0.0002429 | 0.0005755 |
| FQU82_RS08990 | FQU82_RS08990 | hypothetical protein | 0.859166853 | 0.0002467 | 0.0005843 |
| FQU82_RS05080 | FQU82_RS05080 | VOC family protein | 1.846005545 | 0.0002506 | 0.000593 |
| FQU82_RS12120 | FQU82_RS12120 | ATP-binding cassette domain-containing protein | 0.953595593 | 0.0002574 | 0.0006088 |
| FQU82_RS10380 | FQU82_RS10380 | SDR family oxidoreductase | -1.565879279 | 0.0002643 | 0.0006246 |
| FQU82_RS15700 | FQU82_RS15700 | LysR family transcriptional regulator | 1.750398879 | 0.0002667 | 0.0006298 |
| FQU82_RS18865 | FQU82_RS18865 | DUF523 domain-containing protein | 2.181536074 | 0.0002727 | 0.0006437 |
| FQU82_RS19140 | FQU82_RS19140 | hypothetical protein | 0.695980378 | 0.0002739 | 0.0006461 |
| FQU82_RS04380 | smpB | SsrA-binding protein SmpB | 0.488553812 | 0.000276 | 0.0006505 |
| FQU82_RS10230 | FQU82_RS10230 | hypothetical protein | 2.679797502 | 0.0002834 | 0.0006676 |
| FQU82_RS14895 | ybgC | tol-pal system-associated acyl-CoA thioesterase | -1.24905929 | 0.0002841 | 0.0006687 |
| FQU82_RS08405 | FQU82_RS08405 | acyl-CoA dehydrogenase C-terminal domain-containing protein | -0.503780855 | 0.0002862 | 0.0006733 |
| FQU82_RS13100 | FQU82_RS13100 | D-glycerate dehydrogenase | -0.450040056 | 0.0002895 | 0.0006805 |
| FQU82_RS14120 | basC | putative histamine N-monooxygenase | -1.680444794 | 0.0002992 | 0.0007029 |
| FQU82_RS16585 | FQU82_RS16585 | septation protein IspZ | -0.816864602 | 0.0003096 | 0.0007267 |
| Novel00046 | - | PF01323:DSBA-like thioredoxin domain | 2.269920289 | 0.0003104 | 0.0007283 |
| Novel00348 | - | PF00106:short chain dehydrogenase | 4.218976511 | 0.0003108 | 0.0007284 |
| Novel00251 | - | - | 1.56499321 | 0.0003109 | 0.0007284 |
| FQU82_RS19010 | dnaJ | molecular chaperone DnaJ | 0.391082495 | 0.0003121 | 0.0007307 |
| FQU82_RS10225 | FQU82_RS10225 | hypothetical protein | 5.136737649 | 0.0003127 | 0.0007314 |
| FQU82_RS01035 | FQU82_RS01035 | nuclear transport factor 2 family protein | 1.263029064 | 0.0003128 | 0.0007314 |
| FQU82_RS01785 | FQU82_RS01785 | oxidative damage protection protein | -0.712971071 | 0.0003138 | 0.0007332 |
| FQU82_RS13325 | FQU82_RS13325 | PLP-dependent aminotransferase family protein | 0.575697718 | 0.0003316 | 0.0007744 |
| FQU82_RS11060 | catA | catechol 1,2-dioxygenase | 0.703331666 | 0.0003394 | 0.0007921 |
| Novel00158 | - | PF00171:Aldehyde dehydrogenase family|PF01619:Proline dehydrogenase|PF14850:DNA-binding domain of Proline dehydrogenase | -0.64100558 | 0.0003468 | 0.0008087 |
| FQU82_RS16010 | FQU82_RS16010 | YbaN family protein | 0.835921464 | 0.0003579 | 0.0008341 |
| FQU82_RS02330 | FQU82_RS02330 | hypothetical protein | 0.699254606 | 0.0003602 | 0.000839 |
| FQU82_RS07670 | FQU82_RS07670 | MerR family transcriptional regulator | 1.173227436 | 0.000366 | 0.0008519 |
| FQU82_RS14695 | FQU82_RS14695 | SDR family oxidoreductase | 0.507853307 | 0.0003665 | 0.0008524 |
| FQU82_RS05465 | FQU82_RS05465 | RnfABCDGE type electron transport complex subunit B | -1.056084378 | 0.0003691 | 0.0008579 |
| FQU82_RS01025 | FQU82_RS01025 | RluA family pseudouridine synthase | -1.839668688 | 0.0003693 | 0.000858 |
| FQU82_RS16230 | FQU82_RS16230 | CaiB/BaiF CoA-transferase family protein | 0.799047724 | 0.0003718 | 0.0008631 |
| FQU82_RS09475 | kdsB | 3-deoxy-manno-octulosonate cytidylyltransferase | -0.565033702 | 0.0003768 | 0.0008743 |
| FQU82_RS02420 | FQU82_RS02420 | AarF/ABC1/UbiB kinase family protein | 0.406759764 | 0.0003802 | 0.0008814 |
| FQU82_RS17530 | FQU82_RS17530 | EamA family transporter | -0.781680307 | 0.0003805 | 0.0008816 |
| FQU82_RS18395 | glmM | phosphoglucosamine mutase | -0.261710005 | 0.0003818 | 0.000884 |
| FQU82_RS14355 | FQU82_RS14355 | thiamine pyrophosphate-binding protein | 0.717020675 | 0.0003826 | 0.0008853 |
| FQU82_RS05065 | FQU82_RS05065 | hypothetical protein | 1.583050095 | 0.0003848 | 0.0008898 |
| FQU82_RS17630 | rpoZ | DNA-directed RNA polymerase subunit omega | -0.426790817 | 0.0003852 | 0.0008902 |
| Novel00015 | - | PF13379:NMT1-like family | 2.32391464 | 0.0003949 | 0.000912 |
| FQU82_RS13775 | FQU82_RS13775 | ABC transporter ATP-binding protein | -0.541801748 | 0.000396 | 0.0009139 |
| Novel00218 | - | PF00120:Glutamine synthetase, catalytic domain|PF03951:Glutamine synthetase, beta-Grasp domain | -0.66373413 | 0.0003992 | 0.0009207 |
| FQU82_RS18655 | FQU82_RS18655 | MATE family efflux transporter | 0.562029591 | 0.000402 | 0.0009267 |
| FQU82_RS11600 | FQU82_RS11600 | PQQ-dependent sugar dehydrogenase | -0.368994296 | 0.0004027 | 0.0009275 |
| FQU82_RS11380 | argC | N-acetyl-gamma-glutamyl-phosphate reductase | -0.362835196 | 0.0004059 | 0.0009344 |
| FQU82_RS08760 | FQU82_RS08760 | PLP-dependent aminotransferase family protein | 1.065130624 | 0.0004072 | 0.0009367 |
| FQU82_RS11205 | FQU82_RS11205 | YeeE/YedE thiosulfate transporter family protein | 1.847375498 | 0.0004167 | 0.000958 |
| FQU82_RS05300 | estB | esterase EstB | 0.479997286 | 0.0004202 | 0.0009654 |
| FQU82_RS12850 | FQU82_RS12850 | PqiC family protein | 0.684739437 | 0.0004338 | 0.000996 |
| FQU82_RS16065 | FQU82_RS16065 | efflux RND transporter periplasmic adaptor subunit | -0.742190726 | 0.000443 | 0.0010166 |
| Novel00135 | - | PF02599:Global regulator protein family | 1.761954958 | 0.0004456 | 0.0010218 |
| FQU82_RS11395 | rpiA | ribose-5-phosphate isomerase RpiA | -0.524986173 | 0.0004526 | 0.0010372 |
| FQU82_RS14150 | basB | acinetobactin non-ribosomal peptide synthetase subunit BasB | -1.134046501 | 0.0004539 | 0.0010396 |
| FQU82_RS09350 | FQU82_RS09350 | AraC family transcriptional regulator | 0.577325267 | 0.0004563 | 0.0010442 |
| Novel00149 | - | - | 5.037607394 | 0.0004606 | 0.0010535 |
| FQU82_RS08420 | FQU82_RS08420 | LysR family transcriptional regulator | 0.847946357 | 0.0004612 | 0.0010543 |
| FQU82_RS12340 | miaA | tRNA (adenosine(37)-N6)-dimethylallyltransferase MiaA | 0.573121514 | 0.0004653 | 0.0010629 |
| FQU82_RS05955 | rsmI | 16S rRNA (cytidine(1402)-2'-O)-methyltransferase | -0.968480824 | 0.0004695 | 0.0010719 |
| FQU82_RS17200 | FQU82_RS17200 | Rrf2 family transcriptional regulator | -0.928344243 | 0.0004725 | 0.001078 |
| FQU82_RS08430 | FQU82_RS08430 | acetyl/propionyl/methylcrotonyl-CoA carboxylase subunit alpha | -0.372350003 | 0.0004889 | 0.0011146 |
| FQU82_RS10545 | FQU82_RS10545 | alpha/beta fold hydrolase | 2.338228451 | 0.0004963 | 0.0011307 |
| FQU82_RS02030 | FQU82_RS02030 | CHAP domain-containing protein | -2.478123497 | 0.000499 | 0.0011362 |
| FQU82_RS02230 | FQU82_RS02230 | hypothetical protein | -0.458993612 | 0.0005018 | 0.0011418 |
| FQU82_RS15110 | dapF | diaminopimelate epimerase | -0.343823289 | 0.0005038 | 0.0011457 |
| FQU82_RS12960 | gltS | sodium/glutamate symporter | -0.389215106 | 0.0005128 | 0.0011653 |
| FQU82_RS18715 | FQU82_RS18715 | hypothetical protein | -0.306599811 | 0.0005175 | 0.0011752 |
| FQU82_RS16130 | FQU82_RS16130 | DNA-3-methyladenine glycosylase | 0.83703998 | 0.0005189 | 0.0011778 |
| FQU82_RS07045 | hrpA | ATP-dependent RNA helicase HrpA | 0.258808376 | 0.0005494 | 0.0012462 |
| FQU82_RS08945 | FQU82_RS08945 | AraC family transcriptional regulator | 0.76952655 | 0.0005583 | 0.0012655 |
| FQU82_RS02900 | FQU82_RS02900 | Fe2+-dependent dioxygenase | -2.007255466 | 0.0005593 | 0.001267 |
| FQU82_RS10155 | FQU82_RS10155 | helix-turn-helix domain-containing protein | 0.792529467 | 0.0005683 | 0.0012865 |
| FQU82_RS05920 | FQU82_RS05920 | FAD-dependent monooxygenase | -0.427410983 | 0.0005834 | 0.00132 |
| FQU82_RS15240 | FQU82_RS15240 | lytic transglycosylase domain-containing protein | -0.85961259 | 0.0005936 | 0.0013421 |
| FQU82_RS17250 | FQU82_RS17250 | NUDIX domain-containing protein | 0.618349714 | 0.0005968 | 0.0013486 |
| FQU82_RS09295 | FQU82_RS09295 | YceI family protein | 0.339793741 | 0.0005985 | 0.0013515 |
| FQU82_RS18245 | tenA | thiaminase II | -0.713676577 | 0.0005997 | 0.0013534 |
| FQU82_RS03405 | FQU82_RS03405 | hypothetical protein | 0.76653048 | 0.0006006 | 0.0013545 |
| FQU82_RS16785 | FQU82_RS16785 | SDR family oxidoreductase | 1.308402421 | 0.0006051 | 0.0013638 |
| FQU82_RS15770 | FQU82_RS15770 | polyprenyl synthetase family protein | -0.646961019 | 0.0006222 | 0.0014015 |
| FQU82_RS06040 | FQU82_RS06040 | diguanylate cyclase | 1.062205546 | 0.0006226 | 0.0014015 |
| FQU82_RS15385 | FQU82_RS15385 | DOMON-like domain-containing protein | -0.676914302 | 0.0006393 | 0.0014381 |
| FQU82_RS07085 | benA | benzoate 1,2-dioxygenase large subunit | 1.432579085 | 0.0006427 | 0.0014449 |
| FQU82_RS14645 | FQU82_RS14645 | diguanylate cyclase | 1.077236564 | 0.0006466 | 0.0014526 |
| FQU82_RS09150 | FQU82_RS09150 | hypothetical protein | 0.875748684 | 0.0006514 | 0.0014626 |
| FQU82_RS16775 | FQU82_RS16775 | acyl-CoA dehydrogenase family protein | 0.775591646 | 0.0006523 | 0.0014636 |
| FQU82_RS09485 | FQU82_RS09485 | PilZ domain-containing protein | -0.85298 | 0.0006603 | 0.0014806 |
| FQU82_RS15585 | FQU82_RS15585 | tRNA (cytidine(34)-2'-O)-methyltransferase | -0.738124702 | 0.0006686 | 0.0014984 |
| FQU82_RS11410 | FQU82_RS11410 | TrkH family potassium uptake protein | -0.773797408 | 0.0006696 | 0.0014996 |
| FQU82_RS05830 | FQU82_RS05830 | tape measure protein | 0.800817357 | 0.0006855 | 0.0015343 |
| FQU82_RS09575 | FQU82_RS09575 | NAD(P)/FAD-dependent oxidoreductase | 0.706839984 | 0.0006962 | 0.0015572 |
| FQU82_RS00955 | FQU82_RS00955 | hypothetical protein | 0.75598139 | 0.000701 | 0.001567 |
| FQU82_RS06910 | FQU82_RS06910 | NADP(H)-dependent aldo-keto reductase | 0.367817744 | 0.0007028 | 0.00157 |
| FQU82_RS05930 | FQU82_RS05930 | MerR family transcriptional regulator | 1.421590763 | 0.0007034 | 0.0015703 |
| FQU82_RS06930 | FQU82_RS06930 | peptidoglycan DD-metalloendopeptidase family protein | -1.5030597 | 0.0007069 | 0.0015772 |
| FQU82_RS12655 | FQU82_RS12655 | transglutaminase family protein | 0.848220452 | 0.0007101 | 0.0015834 |
| FQU82_RS18820 | FQU82_RS18820 | acyltransferase | -1.463967032 | 0.0007232 | 0.0016116 |
| FQU82_RS02345 | FQU82_RS02345 | CBS domain-containing protein | -0.400394337 | 0.0007341 | 0.0016349 |
| FQU82_RS00995 | FQU82_RS00995 | type VI secretion system Vgr family protein | 0.393810959 | 0.0007382 | 0.0016428 |
| FQU82_RS17215 | FQU82_RS17215 | dicarboxylate/amino acid:cation symporter | 0.420770332 | 0.0007401 | 0.0016458 |
| Novel00094 | - | PF02566:OsmC-like protein | 1.554242848 | 0.0007404 | 0.0016458 |
| Novel00150 | - | - | 2.407041982 | 0.0007543 | 0.0016756 |
| Novel00351 | - | - | 2.568198786 | 0.0007574 | 0.0016814 |
| FQU82_RS04795 | FQU82_RS04795 | hypothetical protein | 0.804650977 | 0.0007787 | 0.001727 |
| FQU82_RS17410 | FQU82_RS17410 | hypothetical protein | 0.319942465 | 0.0007789 | 0.001727 |
| FQU82_RS11210 | FQU82_RS11210 | YeeE/YedE family protein | 1.425485759 | 0.0007822 | 0.0017332 |
| FQU82_RS00170 | cybC | cytochrome b562 | 0.370853188 | 0.0007837 | 0.0017354 |
| FQU82_RS16730 | rsmA | 16S rRNA (adenine(1518)-N(6)/adenine(1519)-N(6))-dimethyltransferase RsmA | -0.318768264 | 0.0007863 | 0.0017401 |
| FQU82_RS05165 | FQU82_RS05165 | LysR substrate-binding domain-containing protein | 1.056469508 | 0.0008162 | 0.0018053 |
| FQU82_RS12290 | FQU82_RS12290 | ethanolamine ammonia-lyase subunit EutB | 2.096833217 | 0.0008359 | 0.0018477 |
| FQU82_RS17885 | FQU82_RS17885 | aminotransferase class I/II-fold pyridoxal phosphate-dependent enzyme | 0.39803751 | 0.000837 | 0.0018488 |
| FQU82_RS01050 | FQU82_RS01050 | Lrp/AsnC ligand binding domain-containing protein | 0.99004534 | 0.0008513 | 0.0018793 |
| FQU82_RS18605 | FQU82_RS18605 | hypothetical protein | 0.302376972 | 0.0008552 | 0.0018867 |
| FQU82_RS04360 | FQU82_RS04360 | outer membrane protein assembly factor BamD | -0.259956792 | 0.0008656 | 0.0019079 |
| FQU82_RS13105 | FQU82_RS13105 | hypothetical protein | 0.907202195 | 0.0008659 | 0.0019079 |
| FQU82_RS16255 | FQU82_RS16255 | patatin family protein | -0.816977977 | 0.0008737 | 0.0019241 |
| Novel00240 | - | - | -1.721873197 | 0.0008952 | 0.0019702 |
| FQU82_RS09215 | FQU82_RS09215 | fimbrial protein | -1.284322182 | 0.000917 | 0.0020169 |
| FQU82_RS00355 | FQU82_RS00355 | alpha/beta hydrolase | 0.360385223 | 0.0009193 | 0.0020207 |
| FQU82_RS01655 | lptG | LPS export ABC transporter permease LptG | -0.371817031 | 0.0009329 | 0.002048 |
| FQU82_RS16430 | FQU82_RS16430 | hypothetical protein | -0.624009041 | 0.0009329 | 0.002048 |
| FQU82_RS09490 | FQU82_RS09490 | TatD family hydrolase | -0.428256476 | 0.0009335 | 0.0020481 |
| Novel00210 | - | - | 3.032052024 | 0.0009429 | 0.0020675 |
| Novel00054 | - | PF01430:Hsp33 protein | 1.144205849 | 0.0009464 | 0.0020738 |
| Novel00147 | - | - | 4.879246497 | 0.0009571 | 0.0020959 |
| FQU82_RS11630 | kynU | kynureninase | 1.336525734 | 0.0009668 | 0.0021158 |
| FQU82_RS09900 | FQU82_RS09900 | histidine-type phosphatase | -1.481222085 | 0.0010068 | 0.0022022 |
| FQU82_RS10645 | FQU82_RS10645 | HlyD family secretion protein | -2.040595137 | 0.0010116 | 0.0022112 |
| FQU82_RS08660 | yfcF | glutathione transferase | 0.674415293 | 0.0010541 | 0.0023027 |
| FQU82_RS04950 | FQU82_RS04950 | homocysteine S-methyltransferase family protein | -0.7229417 | 0.0010627 | 0.00232 |
| FQU82_RS14110 | basE | (2,3-dihydroxybenzoyl)adenylate synthase BasE | -1.514241117 | 0.0010764 | 0.0023485 |
| FQU82_RS08955 | dapC | succinyldiaminopimelate transaminase | -0.389310773 | 0.0010841 | 0.0023639 |
| FQU82_RS03985 | upp | uracil phosphoribosyltransferase | -0.297665888 | 0.0010935 | 0.002383 |
| FQU82_RS08795 | FQU82_RS08795 | MFS transporter | 2.762085096 | 0.0011028 | 0.0024017 |
| FQU82_RS16110 | FQU82_RS16110 | tetratricopeptide repeat protein | 0.509555108 | 0.0011062 | 0.0024077 |
| Novel00322 | - | PF00486:Transcriptional regulatory protein, C terminal | 0.889861397 | 0.0011182 | 0.0024323 |
| FQU82_RS14100 | basG | acinetobactin biosynthesis histidine decarboxylase BasG | -1.639523841 | 0.001122 | 0.0024391 |
| FQU82_RS03290 | FQU82_RS03290 | 3'-5' exonuclease | -0.628785395 | 0.0011384 | 0.0024731 |
| FQU82_RS18285 | betT | choline BCCT transporter BetT | -0.31315542 | 0.0011496 | 0.002496 |
| FQU82_RS05650 | FQU82_RS05650 | hypothetical protein | 0.506349039 | 0.0011538 | 0.0025036 |
| Novel00145 | - | PF04965:Baseplate wedge protein gp25|PF05638:Type VI secretion system effector, Hcp | 1.448296045 | 0.0011628 | 0.0025216 |
| FQU82_RS01500 | dinB | DNA polymerase IV | 1.191017787 | 0.001182 | 0.0025616 |
| FQU82_RS11155 | FQU82_RS11155 | MarR family winged helix-turn-helix transcriptional regulator | -0.995291068 | 0.0011881 | 0.0025734 |
| FQU82_RS06535 | FQU82_RS06535 | metal-dependent hydrolase | 1.659438094 | 0.0012183 | 0.002637 |
| Novel00229 | - | PF00202:Aminotransferase class-III|PF00282:Pyridoxal-dependent decarboxylase conserved domain | 0.476274867 | 0.0012531 | 0.0027107 |
| FQU82_RS14255 | FQU82_RS14255 | ATP-binding protein | -0.249509795 | 0.0012572 | 0.0027179 |
| FQU82_RS18930 | FQU82_RS18930 | dienelactone hydrolase family protein | -0.373139441 | 0.0012616 | 0.0027259 |
| FQU82_RS10205 | FQU82_RS10205 | gamma-butyrobetaine hydroxylase-like domain-containing protein | 3.022609445 | 0.0012788 | 0.0027613 |
| FQU82_RS00920 | FQU82_RS00920 | amino acid aminotransferase | -0.255165927 | 0.0012929 | 0.00279 |
| FQU82_RS04240 | FQU82_RS04240 | DUF177 domain-containing protein | -0.238122667 | 0.0012984 | 0.0028001 |
| FQU82_RS01555 | FQU82_RS01555 | MAPEG family protein | -0.788210422 | 0.0013093 | 0.0028221 |
| FQU82_RS07885 | FQU82_RS07885 | hypothetical protein | 1.673951879 | 0.0013423 | 0.0028913 |
| FQU82_RS13885 | FQU82_RS13885 | cysteine peptidase family C39 domain-containing protein | -1.747054316 | 0.0013467 | 0.0028991 |
| FQU82_RS19090 | FQU82_RS19090 | glutathione S-transferase family protein | 0.695802619 | 0.0013538 | 0.0029126 |
| FQU82_RS01205 | FQU82_RS01205 | LysE family transporter | -1.085336146 | 0.0014351 | 0.0030855 |
| FQU82_RS07935 | FQU82_RS07935 | M15 family metallopeptidase | 1.471924112 | 0.0014375 | 0.003089 |
| FQU82_RS09940 | lpxB | lipid-A-disaccharide synthase | -0.901382912 | 0.001453 | 0.0031203 |
| FQU82_RS05545 | FQU82_RS05545 | hypothetical protein | 0.769307275 | 0.0014573 | 0.0031277 |
| FQU82_RS18275 | FQU82_RS18275 | GGDEF domain-containing protein | 1.016908703 | 0.0014757 | 0.0031653 |
| FQU82_RS01615 | ribD | bifunctional diaminohydroxyphosphoribosylaminopyrimidine deaminase/5-amino-6-(5-phosphoribosylamino)uracil reductase RibD | 0.413557077 | 0.0014827 | 0.0031779 |
| FQU82_RS09855 | FQU82_RS09855 | hypothetical protein | -4.603500782 | 0.0014834 | 0.0031779 |
| FQU82_RS15725 | FQU82_RS15725 | DNA-binding response regulator PmrA | -0.428373957 | 0.0014875 | 0.0031849 |
| Novel00187 | - | - | -1.985322238 | 0.0014928 | 0.0031943 |
| FQU82_RS03910 | FQU82_RS03910 | hypothetical protein | 0.316440674 | 0.0014969 | 0.003201 |
| FQU82_RS00770 | FQU82_RS00770 | hypothetical protein | 1.460359746 | 0.0015159 | 0.0032398 |
| FQU82_RS12405 | FQU82_RS12405 | ion transporter | 0.941104829 | 0.0015182 | 0.0032427 |
| FQU82_RS15600 | FQU82_RS15600 | RsiV family protein | 0.490222044 | 0.0015253 | 0.0032559 |
| FQU82_RS17540 | FQU82_RS17540 | hemerythrin domain-containing protein | 0.550614188 | 0.0015283 | 0.0032605 |
| FQU82_RS15815 | FQU82_RS15815 | fatty acid desaturase family protein | -0.946573041 | 0.0016129 | 0.0034389 |
| FQU82_RS09655 | FQU82_RS09655 | ABC transporter ATP-binding protein | -0.66119958 | 0.0016351 | 0.003484 |
| FQU82_RS10995 | FQU82_RS10995 | saccharopine dehydrogenase NADP-binding domain-containing protein | 0.678017273 | 0.0016872 | 0.0035928 |
| FQU82_RS08850 | FQU82_RS08850 | amidohydrolase | 0.708406273 | 0.001694 | 0.0036052 |
| FQU82_RS11405 | FQU82_RS11405 | TrkA family potassium uptake protein | -0.668452384 | 0.0017006 | 0.003617 |
| Novel00084 | - | PF00330:Aconitase family (aconitate hydratase)|PF00694:Aconitase C-terminal domain | 1.353379934 | 0.0017169 | 0.0036495 |
| FQU82_RS13235 | FQU82_RS13235 | alpha/beta hydrolase-fold protein | -1.30170887 | 0.0017494 | 0.0037164 |
| Novel00117 | - | PF17854:FtsK alpha domain|PF01580:FtsK/SpoIIIE family | 0.799077613 | 0.0017548 | 0.0037256 |
| FQU82_RS01535 | FQU82_RS01535 | MFS transporter | 0.397135533 | 0.0017958 | 0.0038104 |
| FQU82_RS03025 | pilW | type IV pilus biogenesis/stability protein PilW | 0.406473173 | 0.0018041 | 0.0038257 |
| FQU82_RS04025 | FQU82_RS04025 | NAD(P)/FAD-dependent oxidoreductase | -0.82624456 | 0.001829 | 0.0038753 |
| FQU82_RS15150 | FQU82_RS15150 | YkvA family protein | 1.454153843 | 0.0018296 | 0.0038753 |
| FQU82_RS02115 | coaE | dephospho-CoA kinase | 0.777918189 | 0.001842 | 0.0038992 |
| FQU82_RS01290 | znuC | zinc ABC transporter ATP-binding protein ZnuC | 0.430594767 | 0.001863 | 0.0039413 |
| Novel00361 | - | PF00873:AcrB/AcrD/AcrF family | 3.763286081 | 0.001874 | 0.0039623 |
| FQU82_RS16495 | FQU82_RS16495 | glycosyltransferase | -0.554338659 | 0.0018808 | 0.0039741 |
| FQU82_RS13830 | FQU82_RS13830 | DUF3465 domain-containing protein | -2.171497627 | 0.0018826 | 0.0039757 |
| FQU82_RS05985 | dnaQ | DNA polymerase III subunit epsilon | -0.495193846 | 0.0018848 | 0.0039779 |
| FQU82_RS11855 | FQU82_RS11855 | molybdopterin molybdotransferase MoeA | 0.685094801 | 0.0018897 | 0.0039858 |
| FQU82_RS02240 | FQU82_RS02240 | hypothetical protein | -0.569685563 | 0.0019087 | 0.0040235 |
| FQU82_RS15630 | FQU82_RS15630 | site-specific recombinase | -0.329255595 | 0.0019295 | 0.0040651 |
| FQU82_RS08910 | FQU82_RS08910 | RNA methyltransferase | -0.572636928 | 0.0019535 | 0.0041132 |
| FQU82_RS16380 | FQU82_RS16380 | YdiU family protein | 0.332721572 | 0.0019569 | 0.0041179 |
| FQU82_RS03225 | FQU82_RS03225 | MFS transporter | -0.680424714 | 0.002022 | 0.0042502 |
| FQU82_RS16770 | FQU82_RS16770 | enoyl-CoA hydratase-related protein | 0.893140965 | 0.0020222 | 0.0042502 |
| FQU82_RS11180 | FQU82_RS11180 | phosphotransferase family protein | 0.412703745 | 0.0020422 | 0.0042897 |
| FQU82_RS16285 | secA | preprotein translocase subunit SecA | -0.237765223 | 0.0020451 | 0.0042933 |
| FQU82_RS10420 | pqqD | pyrroloquinoline quinone biosynthesis peptide chaperone PqqD | -0.985928701 | 0.0020486 | 0.004298 |
| FQU82_RS02775 | FQU82_RS02775 | hypothetical protein | -1.457099815 | 0.0020615 | 0.0043227 |
| FQU82_RS18750 | thiL | thiamine-phosphate kinase | -0.484758228 | 0.0020668 | 0.0043309 |
| FQU82_RS04550 | minE | cell division topological specificity factor MinE | -0.326482428 | 0.0020679 | 0.0043309 |
| FQU82_RS18540 | FQU82_RS18540 | ankyrin repeat domain-containing protein | -0.397624235 | 0.0020769 | 0.0043471 |
| FQU82_RS08730 | mdcE | biotin-independent malonate decarboxylase subunit gamma | 1.448883047 | 0.0020794 | 0.0043499 |
| FQU82_RS18955 | FQU82_RS18955 | DNA-3-methyladenine glycosylase I | 0.614007406 | 0.0020995 | 0.0043892 |
| FQU82_RS07120 | FQU82_RS07120 | hypothetical protein | 2.689118877 | 0.0021267 | 0.0044435 |
| FQU82_RS11375 | FQU82_RS11375 | hypothetical protein | -0.467636932 | 0.0021363 | 0.004461 |
| FQU82_RS01085 | FQU82_RS01085 | CoA-acylating methylmalonate-semialdehyde dehydrogenase | 0.689423564 | 0.0021571 | 0.0045018 |
| FQU82_RS15840 | FQU82_RS15840 | phosphoadenylyl-sulfate reductase | 0.284469555 | 0.0021655 | 0.0045166 |
| Novel00171 | - | PF12833:Helix-turn-helix domain | 4.624351297 | 0.0021825 | 0.004547 |
| FQU82_RS12440 | coq7 | 2-polyprenyl-3-methyl-6-methoxy-1,4-benzoquinone monooxygenase | 0.333972349 | 0.0021836 | 0.004547 |
| FQU82_RS02615 | asd | aspartate-semialdehyde dehydrogenase | -0.316036431 | 0.0021839 | 0.004547 |
| FQU82_RS08725 | FQU82_RS08725 | biotin-independent malonate decarboxylase subunit beta | 1.952700735 | 0.002213 | 0.0046048 |
| FQU82_RS04095 | FQU82_RS04095 | hypothetical protein | 1.60078541 | 0.0022422 | 0.0046629 |
| FQU82_RS14455 | FQU82_RS14455 | DUF3144 domain-containing protein | -0.491948308 | 0.0022457 | 0.0046675 |
| Novel00326 | - | PF00171:Aldehyde dehydrogenase family | 2.047054656 | 0.0022471 | 0.0046677 |
| FQU82_RS15605 | lolA | outer membrane lipoprotein chaperone LolA | -0.327018008 | 0.0023065 | 0.0047882 |
| FQU82_RS18725 | serB | phosphoserine phosphatase SerB | -0.313861828 | 0.0023367 | 0.004848 |
| Novel00082 | - | PF13603:Leucyl-tRNA synthetase, editing domain | -1.68211105 | 0.0023571 | 0.0048876 |
| FQU82_RS05460 | nth | endonuclease III | -0.66306015 | 0.0024097 | 0.0049937 |
| FQU82_RS10180 | FQU82_RS10180 | ferredoxin family protein | 2.484092457 | 0.0024358 | 0.005045 |
| FQU82_RS14240 | FQU82_RS14240 | bile acid:sodium symporter family protein | -0.482493709 | 0.0024911 | 0.0051565 |
| FQU82_RS16610 | FQU82_RS16610 | YajQ family cyclic di-GMP-binding protein | -0.583987448 | 0.0025034 | 0.0051788 |
| FQU82_RS09675 | FQU82_RS09675 | phosphoglycerate mutase family protein | 0.715700466 | 0.0025456 | 0.0052631 |
| FQU82_RS16050 | FQU82_RS16050 | response regulator | 0.582614653 | 0.0025531 | 0.0052755 |
| FQU82_RS08435 | FQU82_RS08435 | enoyl-CoA hydratase/isomerase family protein | -0.446924534 | 0.0025888 | 0.0053462 |
| FQU82_RS09000 | FQU82_RS09000 | DUF962 domain-containing protein | 2.483025047 | 0.00267 | 0.0055107 |
| FQU82_RS09440 | rsmG | 16S rRNA (guanine(527)-N(7))-methyltransferase RsmG | -0.492984187 | 0.0026852 | 0.0055389 |
| FQU82_RS14085 | barB | acinetobactin export ABC transporter permease/ATP-binding subunit BarB | -1.117743073 | 0.0027079 | 0.0055823 |
| FQU82_RS12025 | amvA | multidrug efflux MFS transporter AmvA | -0.493595583 | 0.0027178 | 0.0055995 |
| FQU82_RS18140 | FQU82_RS18140 | FAD-dependent oxidoreductase | 0.690919888 | 0.002729 | 0.0056194 |
| FQU82_RS07730 | FQU82_RS07730 | putative hydro-lyase | -0.493008225 | 0.0027346 | 0.0056276 |
| FQU82_RS10455 | FQU82_RS10455 | hypothetical protein | -0.627461588 | 0.0027617 | 0.0056801 |
| FQU82_RS09400 | FQU82_RS09400 | DUF2237 domain-containing protein | 0.560683765 | 0.0028325 | 0.0058224 |
| FQU82_RS02265 | recD | exodeoxyribonuclease V subunit alpha | 0.642421124 | 0.0028458 | 0.0058436 |
| FQU82_RS16635 | FQU82_RS16635 | TIGR01244 family sulfur transferase | 0.493021362 | 0.0028461 | 0.0058436 |
| FQU82_RS14605 | cgtA | Obg family GTPase CgtA | -0.877339568 | 0.0028553 | 0.0058591 |
| FQU82_RS01455 | cysK | cysteine synthase A | 0.338887363 | 0.0028969 | 0.0059411 |
| FQU82_RS03180 | FQU82_RS03180 | RsiV family protein | 0.336309459 | 0.0029761 | 0.0060999 |
| FQU82_RS01350 | FQU82_RS01350 | hypothetical protein | 0.693865115 | 0.0030267 | 0.0062001 |
| FQU82_RS02065 | FQU82_RS02065 | FUSC family protein | 1.146506933 | 0.0031576 | 0.0064644 |
| FQU82_RS08270 | pobA | 4-hydroxybenzoate 3-monooxygenase | -1.423649119 | 0.0031829 | 0.0065124 |
| Novel00166 | - | PF01230:HIT domain | 1.315848783 | 0.003207 | 0.0065579 |
| FQU82_RS02475 | FQU82_RS02475 | Rne/Rng family ribonuclease | -0.22466267 | 0.0032339 | 0.0066092 |
| FQU82_RS14750 | FQU82_RS14750 | Do family serine endopeptidase | 0.234788396 | 0.0033683 | 0.0068799 |
| FQU82_RS09930 | FQU82_RS09930 | hypothetical protein | -3.854851507 | 0.0033815 | 0.0069029 |
| FQU82_RS11200 | FQU82_RS11200 | MBL fold metallo-hydrolase | 0.862122942 | 0.0034435 | 0.0070254 |
| FQU82_RS08505 | FQU82_RS08505 | hypothetical protein | -0.546981187 | 0.0034492 | 0.007029 |
| FQU82_RS02375 | hemH | ferrochelatase | 0.414320903 | 0.0034492 | 0.007029 |
| FQU82_RS12585 | FQU82_RS12585 | pyruvate, water dikinase regulatory protein | -0.312232588 | 0.0034664 | 0.0070601 |
| FQU82_RS07795 | uca | urea carboxylase | 1.593571041 | 0.0034698 | 0.0070629 |
| FQU82_RS19045 | FQU82_RS19045 | LysR family transcriptional regulatorr | 0.68624385 | 0.0035163 | 0.0071533 |
| Novel00173 | - | PF09694:Bacterial protein of unknown function (Gcw_chp) | 2.419291122 | 0.0035519 | 0.0072218 |
| FQU82_RS03520 | gigA | RsbU family protein phosphatase GigA | 0.344731724 | 0.003585 | 0.0072849 |
| FQU82_RS12710 | FQU82_RS12710 | MetQ/NlpA family ABC transporter substrate-binding protein | 0.230865427 | 0.0035957 | 0.0073025 |
| FQU82_RS09915 | FQU82_RS09915 | sulfite exporter TauE/SafE family protein | -0.463637786 | 0.0036465 | 0.0074014 |
| FQU82_RS01645 | FQU82_RS01645 | leucyl aminopeptidase | -0.213716739 | 0.0036735 | 0.0074519 |
| FQU82_RS10620 | FQU82_RS10620 | hypothetical protein | -1.61865555 | 0.0036778 | 0.0074563 |
| FQU82_RS05095 | FQU82_RS05095 | cupin domain-containing protein | -0.312055025 | 0.0037336 | 0.0075652 |
| FQU82_RS04565 | FQU82_RS04565 | hypothetical protein | -0.76391692 | 0.0037605 | 0.0076152 |
| FQU82_RS08875 | FQU82_RS08875 | LysR family transcriptional regulator | 0.949698956 | 0.0037782 | 0.0076469 |
| FQU82_RS17225 | plsB | glycerol-3-phosphate 1-O-acyltransferase PlsB | -0.219250065 | 0.0037835 | 0.0076531 |
| FQU82_RS05735 | FQU82_RS05735 | major capsid protein | -0.851999913 | 0.0038355 | 0.007754 |
| FQU82_RS06900 | FQU82_RS06900 | hypothetical protein | -1.202213852 | 0.0038491 | 0.0077759 |
| FQU82_RS03685 | FQU82_RS03685 | copper resistance protein B | 0.778067209 | 0.0038515 | 0.0077759 |
| Novel00266 | - | - | -2.152069465 | 0.0038529 | 0.0077759 |
| FQU82_RS17205 | yihA | ribosome biogenesis GTP-binding protein YihA/YsxC | -0.468226762 | 0.0038688 | 0.0078035 |
| FQU82_RS17895 | FQU82_RS17895 | SulP family inorganic anion transporter | -0.833979496 | 0.0038825 | 0.0078266 |
| Novel00044 | - | - | -4.778702347 | 0.0038934 | 0.0078443 |
| Novel00247 | - | PF00521:DNA gyrase/topoisomerase IV, subunit A|PF03989:DNA gyrase C-terminal domain, beta-propeller | -0.72124973 | 0.0039279 | 0.0079092 |
| FQU82_RS04605 | FQU82_RS04605 | class II glutamine amidotransferase | 0.484895504 | 0.0039333 | 0.0079157 |
| Novel00066 | - | PF00303:Thymidylate synthase | -0.734088139 | 0.0039571 | 0.0079591 |
| FQU82_RS04435 | FQU82_RS04435 | NAD-dependent succinate-semialdehyde dehydrogenase | 0.72238967 | 0.0040191 | 0.0080791 |
| FQU82_RS00935 | prpC | 2-methylcitrate synthase | -0.332819881 | 0.0040271 | 0.0080906 |
| FQU82_RS08195 | FQU82_RS08195 | DUF1330 domain-containing protein | 1.215634447 | 0.004058 | 0.0081467 |
| FQU82_RS17505 | FQU82_RS17505 | NAD(P)H-dependent oxidoreductase | 0.879474111 | 0.0040596 | 0.0081467 |
| FQU82_RS18940 | mutY | A/G-specific adenine glycosylase | -0.754525257 | 0.0040621 | 0.0081472 |
| FQU82_RS15245 | htpX | protease HtpX | 0.211693548 | 0.004084 | 0.0081864 |
| FQU82_RS09960 | FQU82_RS09960 | ABC transporter permease | 1.148193662 | 0.0040924 | 0.0081986 |
| FQU82_RS18580 | alc | allantoicase | 0.67222618 | 0.0041197 | 0.0082486 |
| FQU82_RS05975 | nudC | NAD(+) diphosphatase | -0.485766627 | 0.0041446 | 0.008294 |
| FQU82_RS03745 | FQU82_RS03745 | LysR substrate-binding domain-containing protein | 1.261663255 | 0.0041512 | 0.0083017 |
| FQU82_RS10240 | FQU82_RS10240 | LysR family transcriptional regulator | -0.612999082 | 0.0041532 | 0.0083017 |
| FQU82_RS09455 | FQU82_RS09455 | MotA/TolQ/ExbB proton channel family protein | -1.042834487 | 0.0042467 | 0.0084838 |
| FQU82_RS01720 | FQU82_RS01720 | hypothetical protein | 0.402554659 | 0.0042921 | 0.0085696 |
| Novel00004 | - | PF01751:Toprim domain|PF00986:DNA gyrase B subunit, carboxyl terminus|PF21249:GyrB, hook|PF00204:DNA gyrase B|PF18053:DNA gyrase B subunit insert domain|PF07361:Cytochrome b562 | 0.59797557 | 0.0043053 | 0.0085913 |
| FQU82_RS16510 | glnE | bifunctional [glutamate--ammonia ligase]-adenylyl-L-tyrosine phosphorylase/[glutamate--ammonia-ligase] adenylyltransferase | 0.249302886 | 0.0043219 | 0.0086194 |
| FQU82_RS13855 | FQU82_RS13855 | MFS transporter | 1.137552309 | 0.0043435 | 0.0086578 |
| FQU82_RS08065 | paaC | phenylacetate-CoA oxygenase subunit PaaC | -0.491110322 | 0.004374 | 0.0087136 |
| FQU82_RS02995 | FQU82_RS02995 | carboxy terminal-processing peptidase | -0.213004058 | 0.0043974 | 0.0087554 |
| FQU82_RS17870 | FQU82_RS17870 | hypothetical protein | -1.638728659 | 0.0044302 | 0.0088158 |
| FQU82_RS08895 | FQU82_RS08895 | alpha/beta fold hydrolase | -0.461369356 | 0.0044333 | 0.0088169 |
| FQU82_RS01095 | FQU82_RS01095 | AMP-binding protein | 0.819106356 | 0.0044406 | 0.0088264 |
| FQU82_RS04895 | FQU82_RS04895 | dihydrodipicolinate synthase family protein | 1.348741274 | 0.0046232 | 0.0091843 |
| FQU82_RS10460 | FQU82_RS10460 | hypothetical protein | -2.305235576 | 0.0046804 | 0.0092927 |
| Novel00349 | - | - | 2.705807122 | 0.0047643 | 0.0094498 |
| FQU82_RS08575 | FQU82_RS08575 | GNAT family N-acetyltransferase | 1.295869057 | 0.0047648 | 0.0094498 |
| FQU82_RS09470 | lpxK | tetraacyldisaccharide 4'-kinase | -0.655335537 | 0.0048582 | 0.0096278 |
| FQU82_RS00130 | FQU82_RS00130 | anhydro-N-acetylmuramic acid kinase | 0.282036458 | 0.00486 | 0.0096278 |
| FQU82_RS07855 | tssB | type VI secretion system contractile sheath small subunit | -0.304765667 | 0.0048852 | 0.0096713 |
| FQU82_RS07890 | tssM | type VI secretion system membrane subunit TssM | 0.391851708 | 0.0048874 | 0.0096713 |
| FQU82_RS11555 | FQU82_RS11555 | alpha/beta fold hydrolase | 0.488036393 | 0.0049174 | 0.0097253 |
| FQU82_RS01235 | FQU82_RS01235 | PadR family transcriptional regulator | 0.884330356 | 0.0049407 | 0.0097659 |
| FQU82_RS05570 | FQU82_RS05570 | hypothetical protein | 1.293528358 | 0.0049506 | 0.00978 |
| FQU82_RS02760 | FQU82_RS02760 | DUF4105 domain-containing protein | -0.315892023 | 0.0051365 | 0.0101416 |
| FQU82_RS11435 | FQU82_RS11435 | Rieske (2Fe-2S) protein | -0.643394255 | 0.0051561 | 0.0101748 |
| FQU82_RS17330 | FQU82_RS17330 | acyl-CoA dehydrogenase C-terminal domain-containing protein | -0.207969447 | 0.0051774 | 0.0102111 |
| FQU82_RS09600 | FQU82_RS09600 | biopolymer transporter ExbD | 1.502557218 | 0.005246 | 0.0103406 |
| Novel00192 | - | PF00582:Universal stress protein family | 1.330443807 | 0.0052544 | 0.0103513 |
| FQU82_RS15760 | FQU82_RS15760 | SPFH domain-containing protein | 0.265482211 | 0.0053468 | 0.0105277 |
| FQU82_RS09770 | FQU82_RS09770 | HU family DNA-binding protein | -0.327221285 | 0.0053772 | 0.0105817 |
| FQU82_RS16760 | FQU82_RS16760 | hypothetical protein | -0.283719265 | 0.0054919 | 0.0108014 |
| FQU82_RS18190 | FQU82_RS18190 | AzlD domain-containing protein | -1.032298761 | 0.0055914 | 0.010991 |
| FQU82_RS15810 | FQU82_RS15810 | PaaX family transcriptional regulator C-terminal domain-containing protein | 0.793749531 | 0.0056331 | 0.0110669 |
| FQU82_RS09890 | cobT | nicotinate-nucleotide--dimethylbenzimidazole phosphoribosyltransferase | -0.59633587 | 0.005666 | 0.0111253 |
| FQU82_RS18890 | FQU82_RS18890 | hypothetical protein | -0.213305206 | 0.0057187 | 0.0112226 |
| FQU82_RS08265 | pobR | IclR family transcriptional regulator PobR | 1.19581043 | 0.0057878 | 0.0113518 |
| Novel00104 | - | - | 1.77010103 | 0.0058011 | 0.0113716 |
| FQU82_RS13980 | FQU82_RS13980 | peptide MFS transporter | -0.469304391 | 0.0058211 | 0.0114046 |
| FQU82_RS11320 | FQU82_RS11320 | metal-dependent hydrolase | 1.690599923 | 0.005889 | 0.0115312 |
| FQU82_RS03295 | rlmD | 23S rRNA (uracil(1939)-C(5))-methyltransferase RlmD | -0.350724333 | 0.0059813 | 0.0117056 |
| Novel00175 | - | PF06418:CTP synthase N-terminus|PF00117:Glutamine amidotransferase class-I | -0.752870147 | 0.0059966 | 0.0117291 |
| FQU82_RS16070 | FQU82_RS16070 | efflux RND transporter permease subunit | -0.327900023 | 0.0060076 | 0.011744 |
| FQU82_RS01625 | FQU82_RS01625 | riboflavin synthase | 0.324975953 | 0.0060313 | 0.0117797 |
| FQU82_RS11615 | FQU82_RS11615 | S8 family serine peptidase | -0.724050683 | 0.0060324 | 0.0117797 |
| FQU82_RS12210 | FQU82_RS12210 | glutathione S-transferase family protein | 0.82467424 | 0.006082 | 0.0118699 |
| FQU82_RS10505 | FQU82_RS10505 | hypothetical protein | -1.445443083 | 0.0061144 | 0.0119266 |
| FQU82_RS17705 | FQU82_RS17705 | hypothetical protein | -0.42117395 | 0.0061398 | 0.0119695 |
| FQU82_RS01390 | FQU82_RS01390 | LysM peptidoglycan-binding domain-containing protein | -0.60508703 | 0.0061602 | 0.0120028 |
| Novel00347 | - | PF04286:Protein of unknown function (DUF445) | 1.160274595 | 0.0061904 | 0.0120551 |
| FQU82_RS15950 | FQU82_RS15950 | multidrug effflux MFS transporter | -0.367561379 | 0.0063037 | 0.012269 |
| FQU82_RS12360 | FQU82_RS12360 | type II toxin-antitoxin system RelB/DinJ family antitoxin | -1.305361805 | 0.0063351 | 0.0123233 |
| Novel00154 | - | PF02311:AraC-like ligand binding domain|PF00255:Glutathione peroxidase | 1.66849125 | 0.0064229 | 0.0124846 |
| Novel00194 | - | - | 2.409841979 | 0.0064251 | 0.0124846 |
| FQU82_RS14720 | era | GTPase Era | -0.228349252 | 0.0064391 | 0.0125049 |
| FQU82_RS05280 | ppk1 | polyphosphate kinase 1 | -0.270102363 | 0.006473 | 0.012564 |
| Novel00057 | - | - | 1.361040087 | 0.006501 | 0.0126115 |
| FQU82_RS02175 | FQU82_RS02175 | Na+/H+ antiporter subunit G | -0.718766031 | 0.0065223 | 0.0126459 |
| FQU82_RS05480 | FQU82_RS05480 | class III extradiol ring-cleavage dioxygenase | 0.534219642 | 0.0065547 | 0.0126977 |
| FQU82_RS03890 | FQU82_RS03890 | ribonucleoside-diphosphate reductase subunit alpha | 0.191023318 | 0.0065562 | 0.0126977 |
| FQU82_RS02010 | fadA | acetyl-CoA C-acyltransferase FadA | 0.178809577 | 0.0066276 | 0.012829 |
| FQU82_RS02465 | FQU82_RS02465 | tRNA-Lys | -1.305279674 | 0.0067306 | 0.0130213 |
| FQU82_RS18650 | FQU82_RS18650 | pyridoxal phosphate-dependent aminotransferase | 0.363905142 | 0.0069413 | 0.0134216 |
| Novel00160 | - | PF00375:Sodium:dicarboxylate symporter family | -0.950769098 | 0.0069672 | 0.0134644 |
| FQU82_RS02550 | FQU82_RS02550 | DUF2061 domain-containing protei | 0.876339459 | 0.0069865 | 0.0134943 |
| FQU82_RS03175 | FQU82_RS03175 | hypothetical protein | -0.766119439 | 0.0071132 | 0.0137315 |
| FQU82_RS00940 | acnD | Fe/S-dependent 2-methylisocitrate dehydratase AcnD | -0.271312752 | 0.0071487 | 0.0137925 |
| FQU82_RS15960 | FQU82_RS15960 | SdpI family protein | -0.246131399 | 0.007178 | 0.0138417 |
| FQU82_RS13925 | FQU82_RS13925 | Nif3-like dinuclear metal center hexameric protein | -0.439005137 | 0.0072981 | 0.0140656 |
| FQU82_RS16200 | FQU82_RS16200 | VOC family protein | 0.429340242 | 0.0074374 | 0.0143262 |
| FQU82_RS16410 | FQU82_RS16410 | LysR substrate-binding domain-containing protein | 1.180963792 | 0.0074665 | 0.0143746 |
| Novel00278 | - | PF00581:Rhodanese-like domain | 2.234472886 | 0.0075172 | 0.0144643 |
| Novel00159 | - | PF00474:Sodium:solute symporter family | -1.491521922 | 0.0076226 | 0.0146592 |
| FQU82_RS14850 | FQU82_RS14850 | nicotinate-nicotinamide nucleotide adenylyltransferase | -0.726850067 | 0.0076907 | 0.0147821 |
| FQU82_RS09680 | rpsT | 30S ribosomal protein S20 | -0.35714101 | 0.0076954 | 0.0147832 |
| FQU82_RS04395 | rnpB | RNase P RNA component class A | 0.190193941 | 0.0077548 | 0.0148892 |
| Novel00060 | - | PF02774:Semialdehyde dehydrogenase, dimerisation domain|PF01118:Semialdehyde dehydrogenase, NAD binding domain | 0.588890803 | 0.0077651 | 0.0148978 |
| FQU82_RS10050 | FQU82_RS10050 | ribonuclease D | -0.591322346 | 0.0077677 | 0.0148978 |
| FQU82_RS17240 | recG | ATP-dependent DNA helicase RecG | 0.487146208 | 0.0078025 | 0.0149566 |
| FQU82_RS10070 | FQU82_RS10070 | iron-containing redox enzyme family protein | 0.579547105 | 0.0078187 | 0.0149794 |
| FQU82_RS04615 | FQU82_RS04615 | hypothetical protein | -0.247009434 | 0.0078728 | 0.015075 |
| FQU82_RS12690 | FQU82_RS12690 | zinc-ribbon and DUF3426 domain-containing protein | -0.760908597 | 0.0079165 | 0.0151505 |
| FQU82_RS12545 | FQU82_RS12545 | hypothetical protein | 0.382379142 | 0.0079313 | 0.0151707 |
| FQU82_RS13350 | FQU82_RS13350 | ABC transporter permease | 1.415149684 | 0.0080484 | 0.0153863 |
| Novel00368 | - | PF00209:Sodium:neurotransmitter symporter family | 1.179289334 | 0.008087 | 0.0154519 |
| FQU82_RS16315 | accD | acetyl-CoA carboxylase, carboxyltransferase subunit beta | -0.236068902 | 0.008126 | 0.0155181 |
| FQU82_RS05040 | FQU82_RS05040 | crotonase/enoyl-CoA hydratase family protein | 0.601666411 | 0.0082082 | 0.0156666 |
| FQU82_RS11350 | FQU82_RS11350 | alpha/beta hydrolase | 0.444377454 | 0.0082431 | 0.0157248 |
| FQU82_RS15320 | FQU82_RS15320 | hypothetical protein | 0.273659224 | 0.008275 | 0.0157771 |
| Novel00224 | - | PF08207:Elongation factor P (EF-P) KOW-like domain|PF09285:Elongation factor P, C-terminal|PF01132:Elongation factor P (EF-P) OB domain | -0.939340665 | 0.0082855 | 0.0157887 |
| FQU82_RS15800 | FQU82_RS15800 | AraC family transcriptional regulator | -0.610756299 | 0.0083263 | 0.015858 |
| FQU82_RS18485 | gshB | glutathione synthase | -0.325731046 | 0.0085115 | 0.0162019 |
| FQU82_RS09290 | FQU82_RS09290 | OsmC family protein | 0.612044808 | 0.0086047 | 0.0163706 |
| FQU82_RS18685 | asd | archaetidylserine decarboxylase | -0.62271803 | 0.0086219 | 0.0163945 |
| FQU82_RS10040 | FQU82_RS10040 | YbaB/EbfC family nucleoid-associated protein | 0.455093873 | 0.0087134 | 0.0165596 |
| FQU82_RS09590 | FQU82_RS09590 | malate synthase G | 0.181382407 | 0.0087381 | 0.0165977 |
| FQU82_RS02545 | FQU82_RS02545 | LysR family transcriptional regulator | 0.512832443 | 0.0087757 | 0.0166602 |
| Novel00109 | - | PF08544:GHMP kinases C terminal | -1.578590808 | 0.0089546 | 0.0169908 |
| FQU82_RS05175 | FQU82_RS05175 | MFS transporter | 3.851153268 | 0.0089921 | 0.0170528 |
| Novel00040 | - | PF21157:DnaK suppressor protein DksA, coiled-coil domain|PF01258:Prokaryotic dksA/traR C4-type zinc finger | 0.870251805 | 0.00901 | 0.0170777 |
| FQU82_RS13035 | FQU82_RS13035 | LysR family transcriptional regulator | 0.878494263 | 0.0091623 | 0.017357 |
| FQU82_RS01080 | FQU82_RS01080 | LysR family transcriptional regulator | 0.905283594 | 0.0091735 | 0.017369 |
| FQU82_RS03355 | FQU82_RS03355 | acyl-CoA synthetase | -0.408071788 | 0.0092518 | 0.017508 |
| FQU82_RS14405 | fabR | HTH-type transcriptional repressor FabR | -0.835934755 | 0.0093069 | 0.0176029 |
| Novel00065 | - | PF01625:Peptide methionine sulfoxide reductase | -0.739034798 | 0.0094216 | 0.0178102 |
| FQU82_RS09615 | FQU82_RS09615 | energy transducer TonB | 1.804304418 | 0.0095435 | 0.0180312 |
| Novel00243 | - | PF14602:Hexapeptide repeat of succinyl-transferase|PF04055:Radical SAM superfamily | -1.145500744 | 0.0095595 | 0.0180517 |
| FQU82_RS12065 | tal | transaldolase | -0.249086802 | 0.0096531 | 0.0182169 |
| FQU82_RS14015 | FQU82_RS14015 | Smr/MutS family protein | -0.407722352 | 0.0096572 | 0.0182169 |
| Novel00197 | - | PF00296:Luciferase-like monooxygenase | 2.795259869 | 0.0098281 | 0.0185294 |
| FQU82_RS02500 | FQU82_RS02500 | DUF1345 domain-containing protein | -0.736310288 | 0.0099181 | 0.0186892 |
| FQU82_RS17750 | FQU82_RS17750 | methyltransferase domain-containing protein | -0.477793769 | 0.0099724 | 0.0187816 |
| FQU82_RS07100 | FQU82_RS07100 | heavy metal translocating P-type ATPase | 0.238180206 | 0.0100872 | 0.0189876 |
| FQU82_RS17635 | FQU82_RS17635 | hypothetical protein | -1.012373067 | 0.0101189 | 0.0190373 |
| FQU82_RS08020 | FQU82_RS08020 | FAD/NAD(P)-binding oxidoreductase | 0.9747102 | 0.010125 | 0.0190387 |
| FQU82_RS10235 | FQU82_RS10235 | LysR substrate-binding domain-containing protein | -0.93592061 | 0.010158 | 0.0190906 |
| FQU82_RS04635 | FQU82_RS04635 | PilT/PilU family type 4a pilus ATPase | -0.994158017 | 0.0103594 | 0.0194589 |
| FQU82_RS13170 | FQU82_RS13170 | sulfurtransferase TusA family protein | 0.76739742 | 0.010608 | 0.0199152 |
| FQU82_RS05485 | rlmH | 23S rRNA (pseudouridine(1915)-N(3))-methyltransferase RlmH | -1.212913473 | 0.0106235 | 0.0199338 |
| Novel00346 | - | - | 1.182841961 | 0.0106408 | 0.0199557 |
| FQU82_RS07715 | mumR | LysR family transcriptional regulator MumR | 0.583264009 | 0.0107839 | 0.0202135 |
| FQU82_RS03710 | nadA | quinolinate synthase NadA | -0.248168792 | 0.0108294 | 0.020288 |
| Novel00018 | - | PF00106:short chain dehydrogenase | 0.648346398 | 0.0109154 | 0.0204383 |
| FQU82_RS14350 | FQU82_RS14350 | amino acid permease | 1.094468522 | 0.0111912 | 0.0209437 |
| FQU82_RS14330 | pstB | phosphate ABC transporter ATP-binding protein PstB | -0.278893646 | 0.0113577 | 0.0212441 |
| FQU82_RS09450 | FQU82_RS09450 | ParB/RepB/Spo0J family partition protein | -0.422098486 | 0.0113727 | 0.021261 |
| FQU82_RS11115 | FQU82_RS11115 | PDR/VanB family oxidoreductase | 1.677177569 | 0.0114902 | 0.0214693 |
| FQU82_RS03680 | FQU82_RS03680 | copper resistance system multicopper oxidase | 0.399124215 | 0.0115315 | 0.0215351 |
| FQU82_RS09760 | FQU82_RS09760 | hypothetical protein | 0.487425241 | 0.011548 | 0.0215546 |
| FQU82_RS03275 | FQU82_RS03275 | crotonase/enoyl-CoA hydratase family protein | 0.286083921 | 0.0116543 | 0.0217416 |
| FQU82_RS04365 | rluD | 23S rRNA pseudouridine(1911/1915/1917) synthase RluD | -0.286821124 | 0.0116766 | 0.0217718 |
| FQU82_RS03030 | FQU82_RS03030 | DUF4115 domain-containing protein | -0.25181783 | 0.0117011 | 0.021806 |
| FQU82_RS16335 | FQU82_RS16335 | DUF2804 domain-containing protein | 0.860502534 | 0.011862 | 0.0220943 |
| FQU82_RS05895 | FQU82_RS05895 | cell division protein ZapA | 0.437188226 | 0.0119571 | 0.0222598 |
| FQU82_RS05115 | FQU82_RS05115 | aldehyde dehydrogenase | 0.498878442 | 0.0120004 | 0.0223182 |
| FQU82_RS18025 | FQU82_RS18025 | DNA/RNA non-specific endonuclease | -0.403054546 | 0.0120011 | 0.0223182 |
| FQU82_RS12080 | FQU82_RS12080 | benzoate/H(+) symporter BenE family transporter | 0.577742888 | 0.0120719 | 0.0224383 |
| FQU82_RS08625 | FQU82_RS08625 | family 2A encapsulin nanocompartment cargo protein cysteine desulfurase | 1.97840966 | 0.0120971 | 0.0224732 |
| FQU82_RS00640 | FQU82_RS00640 | hypothetical protein | -0.632789573 | 0.0121984 | 0.0226496 |
| FQU82_RS09805 | FQU82_RS09805 | hypothetical protein | 0.701008801 | 0.0122544 | 0.0227417 |
| FQU82_RS17845 | FQU82_RS17845 | cation diffusion facilitator family transporter | -0.29720992 | 0.0122668 | 0.0227528 |
| FQU82_RS00665 | FQU82_RS00665 | hypothetical protein | 1.191702988 | 0.012285 | 0.0227747 |
| FQU82_RS18690 | FQU82_RS18690 | rhodanese-like domain-containing protein | 0.341363702 | 0.0123374 | 0.0228599 |
| FQU82_RS01485 | FQU82_RS01485 | YgiQ family radical SAM protein | -0.241700593 | 0.0123632 | 0.0228958 |
| FQU82_RS15820 | FQU82_RS15820 | AraC family transcriptional regulator | 0.674313762 | 0.0124455 | 0.0230362 |
| FQU82_RS04195 | bioA | adenosylmethionine--8-amino-7-oxononanoate transaminase | 0.384890489 | 0.0127249 | 0.0235411 |
| FQU82_RS14225 | FQU82_RS14225 | hypothetical protein | 0.442425163 | 0.0127432 | 0.0235626 |
| FQU82_RS18855 | FQU82_RS18855 | IclR family transcriptional regulator | 0.372891325 | 0.0128659 | 0.0237772 |
| FQU82_RS00500 | FQU82_RS00500 | TetR family transcriptional regulator | -0.470917216 | 0.0130802 | 0.0241607 |
| FQU82_RS14280 | FQU82_RS14280 | hypothetical protein | -0.46390034 | 0.0130948 | 0.0241749 |
| FQU82_RS07155 | FQU82_RS07155 | LexA family transcriptional regulator | 0.356630217 | 0.0131816 | 0.0243227 |
| FQU82_RS09665 | FQU82_RS09665 | hypothetical protein | 0.455143488 | 0.0132295 | 0.0243984 |
| FQU82_RS12145 | FQU82_RS12145 | N-acetyltransferase | 0.990775519 | 0.0132671 | 0.024455 |
| Novel00228 | - | PF01343:Peptidase family S49|PF08496:Peptidase family S49 N-terminal | 0.944286953 | 0.0132895 | 0.0244836 |
| FQU82_RS17390 | FQU82_RS17390 | hypothetical protein | -0.5335198 | 0.0133241 | 0.0245346 |
| FQU82_RS08735 | FQU82_RS08735 | malonate decarboxylase holo-ACP synthase | 1.882847974 | 0.0133993 | 0.0246602 |
| FQU82_RS10105 | FQU82_RS10105 | acetoin reductase | 1.404664276 | 0.013473 | 0.024783 |
| FQU82_RS03885 | FQU82_RS03885 | hypothetical protein | 1.425011628 | 0.0138384 | 0.0254421 |
| FQU82_RS02685 | FQU82_RS02685 | ATP-dependent helicase | 0.330338709 | 0.0138529 | 0.0254556 |
| FQU82_RS18925 | FQU82_RS18925 | DUF1328 domain-containing protein | 0.790786092 | 0.0139058 | 0.0255395 |
| FQU82_RS00015 | FQU82_RS00015 | hypothetical protein | 1.207131812 | 0.0139441 | 0.0255966 |
| FQU82_RS10375 | FQU82_RS10375 | ATP-binding cassette domain-containing protein | 1.740995949 | 0.0140842 | 0.0258404 |
| Novel00036 | - | PF00291:Pyridoxal-phosphate dependent enzyme | -0.845378586 | 0.0141976 | 0.026035 |
| FQU82_RS18665 | FQU82_RS18665 | TetR/AcrR family transcriptional regulator | 0.48809321 | 0.0142437 | 0.0261061 |
| FQU82_RS14810 | carO | ornithine uptake porin CarO type 1 | 0.229227173 | 0.0142805 | 0.0261601 |
| FQU82_RS02710 | FQU82_RS02710 | cation:proton antiporter | -0.353136329 | 0.0144046 | 0.0263737 |
| FQU82_RS03380 | nreB | nickel resistance MFS transporter NreB | -0.528767737 | 0.0145915 | 0.0267022 |
| FQU82_RS18445 | FQU82_RS18445 | DciA family protein | 0.786832507 | 0.0146718 | 0.0268354 |
| FQU82_RS12060 | FQU82_RS12060 | LysR family transcriptional regulator | 0.904036239 | 0.0146963 | 0.0268662 |
| FQU82_RS10135 | FQU82_RS10135 | sulfite exporter TauE/SafE family protein | -2.215420841 | 0.0147588 | 0.0269577 |
| FQU82_RS11050 | FQU82_RS11050 | muconate/chloromuconate family cycloisomerase | 1.460018596 | 0.0147615 | 0.0269577 |
| FQU82_RS12540 | FQU82_RS12540 | LysR family transcriptional regulator | 0.717294836 | 0.0148509 | 0.0271071 |
| FQU82_RS13285 | FQU82_RS13285 | ATP-binding protein | 0.64541399 | 0.0149951 | 0.0273563 |
| FQU82_RS16150 | mscL | large conductance mechanosensitive channel protein MscL | 0.200228718 | 0.0150984 | 0.0275305 |
| FQU82_RS11660 | FQU82_RS11660 | HAD-IA family hydrolase | -0.702710963 | 0.0155811 | 0.0283962 |
| FQU82_RS09355 | FQU82_RS09355 | MaoC family dehydratase | -0.614225571 | 0.015614 | 0.0284415 |
| FQU82_RS01420 | FQU82_RS01420 | TetR/AcrR family transcriptional regulator | 1.447278777 | 0.0158378 | 0.0288343 |
| Novel00345 | - | - | 2.447610063 | 0.0159211 | 0.0289712 |
| FQU82_RS17485 | sodC | superoxide dismutase family protein | -0.241480754 | 0.0159772 | 0.0290584 |
| FQU82_RS11960 | FQU82_RS11960 | TetR/AcrR family transcriptional regulator | 0.734847108 | 0.0160387 | 0.0291553 |
| Novel00083 | - | PF13710:ACT domain|PF10369:Small subunit of acetolactate synthase|PF01450:Acetohydroxy acid isomeroreductase, catalytic domain|PF07991:Acetohydroxy acid isomeroreductase, NADPH-binding domain|PF00205:Thiamine pyrophosphate enzyme, central domain|PF02775:Thiamine pyrophosphate enzyme, C-terminal TPP binding domain | -0.407043624 | 0.0161398 | 0.0293241 |
| FQU82_RS12475 | kdpA | potassium-transporting ATPase subunit KdpA | 1.237604593 | 0.0161565 | 0.0293394 |
| FQU82_RS15915 | sbmA | peptide antibiotic transporter SbmA | -0.428428958 | 0.0165182 | 0.0299809 |
| FQU82_RS15055 | FQU82_RS15055 | ChbG/HpnK family deacetylase | 1.270568832 | 0.016616 | 0.0301429 |
| FQU82_RS09830 | FQU82_RS09830 | IucA/IucC family protein | -0.399254789 | 0.0166285 | 0.0301503 |
| Novel00096 | - | PF04333:MlaA lipoprotein | 0.875812833 | 0.0168289 | 0.0304982 |
| FQU82_RS16055 | pilG | twitching motility response regulator PilG | -0.527436805 | 0.0168903 | 0.0305937 |
| FQU82_RS18340 | acs | acetate--CoA ligase | 0.210241219 | 0.0170174 | 0.0308082 |
| FQU82_RS17305 | dxs | 1-deoxy-D-xylulose-5-phosphate synthase | 0.171951341 | 0.0171921 | 0.0311087 |
| FQU82_RS07930 | FQU82_RS07930 | hypothetical protein | 0.851553713 | 0.0174002 | 0.0314693 |
| FQU82_RS11995 | FQU82_RS11995 | lactonase family protein | -0.374792586 | 0.0175809 | 0.0317798 |
| FQU82_RS15720 | FQU82_RS15720 | two-component system sensor histidine kinase PmrB | -0.373177863 | 0.0175961 | 0.0317912 |
| FQU82_RS07620 | FQU82_RS07620 | ferredoxin family protein | -0.557967247 | 0.0176712 | 0.0319105 |
| FQU82_RS05085 | FQU82_RS05085 | FAD-dependent oxidoreductase | 1.439951334 | 0.0176857 | 0.0319205 |
| FQU82_RS14090 | barA | acinetobactin export ABC transporter permease/ATP-binding subunit BarA | -0.863682204 | 0.0177583 | 0.0320354 |
| FQU82_RS16705 | hflX | GTPase HflX | 0.319461845 | 0.0178701 | 0.0322206 |
| FQU82_RS16960 | FQU82_RS16960 | hypothetical protein | -0.539808239 | 0.0179199 | 0.032294 |
| FQU82_RS13215 | FQU82_RS13215 | aminoacyl-histidine dipeptidase | 0.279902732 | 0.0180386 | 0.0324915 |
| FQU82_RS10425 | pqqE | pyrroloquinoline quinone biosynthesis protein PqqE | -0.460332711 | 0.0182008 | 0.0327669 |
| FQU82_RS17480 | FQU82_RS17480 | AI-2E family transporter | -0.310730327 | 0.0182331 | 0.0328086 |
| FQU82_RS07110 | FQU82_RS07110 | GFA family protein | 0.576593747 | 0.0182886 | 0.0328916 |
| FQU82_RS12970 | FQU82_RS12970 | AraC family transcriptional regulator | 1.686159746 | 0.0183471 | 0.0329802 |
| FQU82_RS12860 | FQU82_RS12860 | paraquat-inducible protein A | 0.649964702 | 0.0183707 | 0.033006 |
| FQU82_RS07220 | FQU82_RS07220 | helix-turn-helix domain-containing protein | 1.322714834 | 0.0184442 | 0.0331213 |
| Novel00288 | - | - | 1.594222956 | 0.0186705 | 0.0335107 |
| FQU82_RS13140 | FQU82_RS13140 | phosphoglycerate mutase family protein | -0.585853712 | 0.0186913 | 0.0335311 |
| FQU82_RS16440 | znuD | zinc piracy TonB-dependent receptor ZnuD | 0.502688227 | 0.0187536 | 0.0336259 |
| FQU82_RS05615 | FQU82_RS05615 | hypothetical protein | 1.19054083 | 0.0188204 | 0.0337165 |
| FQU82_RS14025 | xdhB | xanthine dehydrogenase molybdopterin binding subunit | 0.508020824 | 0.0188231 | 0.0337165 |
| FQU82_RS06050 | FQU82_RS06050 | NADPH-dependent 2,4-dienoyl-CoA reductase | -0.280387763 | 0.0188433 | 0.0337355 |
| FQU82_RS18155 | FQU82_RS18155 | LysR substrate-binding domain-containing protein | 0.499380351 | 0.0190278 | 0.0340488 |
| FQU82_RS18385 | recJ | single-stranded-DNA-specific exonuclease RecJ | -0.370944415 | 0.0191526 | 0.0342547 |
| FQU82_RS12645 | alr | alanine racemase | -0.467324085 | 0.0192112 | 0.0343424 |
| Novel00115 | - | PF04377:Arginine-tRNA-protein transferase, C terminus|PF04376:Arginine-tRNA-protein transferase, N terminus | 1.217093816 | 0.0192897 | 0.0344652 |
| FQU82_RS05730 | FQU82_RS05730 | hypothetical protein | -0.983609113 | 0.0198013 | 0.0353481 |
| FQU82_RS01215 | FQU82_RS01215 | DoxX family protein | 0.782700989 | 0.0198037 | 0.0353481 |
| Novel00316 | - | PF19269:Anticodon binding domain | -0.964053253 | 0.0199603 | 0.0356097 |
| FQU82_RS17555 | FQU82_RS17555 | lipase secretion chaperone | 1.595229249 | 0.0199767 | 0.0356211 |
| FQU82_RS15060 | FQU82_RS15060 | glycosyltransferase family 2 protein | 1.040798966 | 0.0200009 | 0.0356463 |
| FQU82_RS14525 | FQU82_RS14525 | NAD(P)/FAD-dependent oxidoreductase | -0.376989537 | 0.0200711 | 0.0357535 |
| FQU82_RS02595 | truA | tRNA pseudouridine(38-40) synthase TruA | -0.495125827 | 0.0200919 | 0.0357726 |
| FQU82_RS15655 | FQU82_RS15655 | YbjQ family protein | -0.828926013 | 0.020128 | 0.035819 |
| FQU82_RS05630 | FQU82_RS05630 | hypothetical protein | 3.515119426 | 0.0203163 | 0.0361359 |
| FQU82_RS04135 | FQU82_RS04135 | pantothenate kinase | -0.66094364 | 0.0204765 | 0.0364027 |
| FQU82_RS03590 | FQU82_RS03590 | anthranilate synthase component I family protein | -1.758258582 | 0.0205007 | 0.0364275 |
| FQU82_RS04625 | bamE | outer membrane protein assembly factor BamE | -0.354986001 | 0.0206006 | 0.0365867 |
| FQU82_RS04235 | FQU82_RS04235 | elongation factor P hydroxylase | 0.861931288 | 0.0208839 | 0.0370713 |
| FQU82_RS18015 | FQU82_RS18015 | hypothetical protein | 1.091837054 | 0.0209705 | 0.0372064 |
| FQU82_RS16480 | FQU82_RS16480 | glycosyltransferase | -0.406194962 | 0.0211456 | 0.0374984 |
| FQU82_RS01620 | FQU82_RS01620 | DNA adenine methylase | 0.227264403 | 0.0213757 | 0.0378874 |
| FQU82_RS17310 | ribA | GTP cyclohydrolase II | 0.596004186 | 0.0214389 | 0.0379805 |
| Novel00208 | - | - | 2.623631559 | 0.0214559 | 0.0379917 |
| Novel00153 | - | - | 3.042318133 | 0.0216565 | 0.0383277 |
| Novel00102 | - | - | 1.781434957 | 0.0217159 | 0.0384137 |
| FQU82_RS14070 | basJ | acinetobactin biosynthesis isochorismate synthase BasJ | -1.572678144 | 0.0217839 | 0.0385148 |
| Novel00310 | - | PF04011:LemA family | 0.886655121 | 0.0218101 | 0.038542 |
| FQU82_RS09240 | FQU82_RS09240 | bifunctional ADP-dependent NAD(P)H-hydrate dehydratase/NAD(P)H-hydrate epimerase | 0.637716531 | 0.0220815 | 0.0390022 |
| FQU82_RS15745 | FQU82_RS15745 | lysophospholipid acyltransferase family protein | 0.748461897 | 0.022117 | 0.0390412 |
| FQU82_RS03000 | FQU82_RS03000 | glycosyltransferase family 1 protein | 0.838797962 | 0.0221255 | 0.0390412 |
| FQU82_RS18170 | FQU82_RS18170 | PLP-dependent aminotransferase family protein | 0.636493847 | 0.0222879 | 0.0393082 |
| FQU82_RS12420 | FQU82_RS12420 | hypothetical protein | -0.718248094 | 0.0223123 | 0.0393317 |
| Novel00221 | - | PF04328:Selenoprotein, putative|PF13722:5TM C-terminal transporter carbon starvation CstA | 1.226810354 | 0.0223692 | 0.0394125 |
| FQU82_RS09480 | FQU82_RS09480 | DNA polymerase III subunit delta | -0.873770107 | 0.0224993 | 0.039622 |
| FQU82_RS09535 | tadA | tRNA adenosine(34) deaminase TadA | -0.700859081 | 0.0226699 | 0.0399026 |
| FQU82_RS05345 | cysN | sulfate adenylyltransferase subunit CysN | -0.184286645 | 0.0228064 | 0.040123 |
| FQU82_RS07200 | FQU82_RS07200 | hypothetical protein | -0.49493697 | 0.0229753 | 0.0404002 |
| FQU82_RS12205 | FQU82_RS12205 | SDR family NAD(P)-dependent oxidoreductase | 1.355759484 | 0.0233072 | 0.0409635 |
| FQU82_RS10110 | FQU82_RS10110 | 2,3-butanediol dehydrogenase | 0.568332801 | 0.0234185 | 0.0411387 |
| FQU82_RS06970 | FQU82_RS06970 | OmpA family protein | -0.234920303 | 0.0235119 | 0.0412823 |
| FQU82_RS00490 | FQU82_RS00490 | thiol:disulfide interchange protein DsbA/DsbL | 0.256367024 | 0.0236516 | 0.0415071 |
| FQU82_RS08210 | FQU82_RS08210 | alpha/beta hydrolase | 0.781969713 | 0.0237545 | 0.0416671 |
| FQU82_RS09610 | FQU82_RS09610 | MotA/TolQ/ExbB proton channel family protein | 0.688203796 | 0.0239388 | 0.0419698 |
| Novel00205 | - | PF00156:Phosphoribosyl transferase domain | 1.056549051 | 0.0240278 | 0.042105 |
| Novel00279 | - | PF03061:Thioesterase superfamily|PF00085:Thioredoxin|PF21352:Thioredoxin 2, N-terminal | 0.937568542 | 0.0242888 | 0.0425413 |
| FQU82_RS08845 | FQU82_RS08845 | hydrolase | 0.662935566 | 0.0243022 | 0.0425438 |
| FQU82_RS19305 | FQU82_RS19305 | KGW motif small protein | 1.837617647 | 0.0243458 | 0.0425991 |
| FQU82_RS08965 | purT | formate-dependent phosphoribosylglycinamide formyltransferase | 0.218613922 | 0.0246579 | 0.0431239 |
| Novel00141 | - | - | 1.046377105 | 0.0246756 | 0.0431337 |
| Novel00091 | - | PF00006:ATP synthase alpha/beta family, nucleotide-binding domain|PF07497:Rho termination factor, RNA-binding domain | -0.666083142 | 0.0247079 | 0.0431689 |
| FQU82_RS00175 | gyrB | DNA topoisomerase (ATP-hydrolyzing) subunit B | -0.163821995 | 0.0250946 | 0.0438154 |
| FQU82_RS13290 | FQU82_RS13290 | response regulator transcription factor | 0.798506699 | 0.0251025 | 0.0438154 |
| FQU82_RS16205 | FQU82_RS16205 | SMR family transporter | -1.816638075 | 0.0252846 | 0.0441115 |
| FQU82_RS00350 | FQU82_RS00350 | hypothetical protein | 0.665119346 | 0.0253877 | 0.0442696 |
| Novel00329 | - | PF04341:Protein of unknown function, DUF485|PF00474:Sodium:solute symporter family | -0.593901106 | 0.0254313 | 0.0443239 |
| FQU82_RS10850 | FQU82_RS10850 | MFS transporter | 1.494617702 | 0.0254886 | 0.044402 |
| FQU82_RS05275 | mtgA | monofunctional biosynthetic peptidoglycan transglycosylase | -0.489857683 | 0.02565 | 0.0446611 |
| FQU82_RS13275 | FQU82_RS13275 | thiamine phosphate synthase | -0.650888537 | 0.0258027 | 0.044905 |
| FQU82_RS05610 | FQU82_RS05610 | hypothetical protein | 0.921282156 | 0.025973 | 0.0451793 |
| FQU82_RS16865 | FQU82_RS16865 | DUF2789 family protein | 0.533392931 | 0.0260571 | 0.0453035 |
| FQU82_RS05100 | FQU82_RS05100 | alpha/beta hydrolase | 0.442026312 | 0.0261658 | 0.0454701 |
| FQU82_RS10165 | chrA | chromate efflux transporter | 0.641790388 | 0.0262088 | 0.0455226 |
| FQU82_RS11030 | FQU82_RS11030 | IlvD/Edd family dehydratase | 1.341159983 | 0.0263529 | 0.0457505 |
| FQU82_RS05645 | FQU82_RS05645 | hypothetical protein | 1.144584084 | 0.026644 | 0.0462333 |
| FQU82_RS05900 | FQU82_RS05900 | hypothetical protein | 0.285396699 | 0.0267282 | 0.0463568 |
| FQU82_RS04350 | hemA | glutamyl-tRNA reductase | 0.260401416 | 0.0268762 | 0.0465725 |
| FQU82_RS05045 | pnuC | nicotinamide riboside transporter PnuC | -0.819588662 | 0.0268788 | 0.0465725 |
| FQU82_RS10860 | FQU82_RS10860 | GntR family transcriptional regulatorfamily|PF07702:UTRA domain | 0.578681687 | 0.0270128 | 0.0467819 |
| FQU82_RS11895 | nirD | nitrite reductase small subunit NirD | 0.945294523 | 0.0270851 | 0.0468842 |
| FQU82_RS18585 | FQU82_RS18585 | ureidoglycolate lyase | 0.63483682 | 0.0271654 | 0.0470002 |
| FQU82_RS07025 | FQU82_RS07025 | DUF1653 domain-containing protein | 0.821597247 | 0.0271898 | 0.0470196 |
| FQU82_RS17185 | FQU82_RS17185 | PLP-dependent transferase | -0.308561693 | 0.0272332 | 0.0470718 |
| FQU82_RS03490 | gspE | type II secretion system ATPase GspE | 0.346047916 | 0.027776 | 0.0479865 |
| FQU82_RS13345 | FQU82_RS13345 | ATP-binding cassette domain-containing protein | 0.827866807 | 0.0278022 | 0.048001 |
| FQU82_RS12010 | FQU82_RS12010 | phospholipase C, phosphocholine-specific | 0.894379355 | 0.0278114 | 0.048001 |
| Novel00327 | - | PF00436:Single-strand binding protein family | 0.785072183 | 0.0281811 | 0.0486155 |
| FQU82_RS13970 | FQU82_RS13970 | chorismate lyase | -0.729952102 | 0.0282042 | 0.0486316 |
| FQU82_RS16325 | FQU82_RS16325 | AraC family transcriptional regulator | 0.870044644 | 0.0284895 | 0.0490999 |
| FQU82_RS13095 | FQU82_RS13095 | M48 family metalloprotease | 0.311383917 | 0.0285548 | 0.0491885 |
| FQU82_RS02040 | FQU82_RS02040 | thioesterase family protein | 0.427994388 | 0.0286073 | 0.0492549 |
| FQU82_RS01995 | FQU82_RS01995 | MerR family transcriptional regulator | 1.02317705 | 0.0286827 | 0.049361 |
| Novel00273 | - | PF07517:SecA DEAD-like domain|PF21090:SecA P-loop domain|PF01043:SecA preprotein cross-linking domain|PF07516:SecA Wing and Scaffold domain | 0.440365195 | 0.0288305 | 0.0495912 |
| FQU82_RS00290 | FQU82_RS00290 | DMT family protein | 0.646193202 | 0.0291514 | 0.0501189 |
| FQU82_RS03455 | tilS | tRNA lysidine(34) synthetase TilS | 0.640016595 | 0.0292169 | 0.0502073 |
| FQU82_RS07915 | tssA | type VI secretion system protein TssA | 0.85354218 | 0.0292758 | 0.0502765 |
| FQU82_RS12445 | FQU82_RS12445 | DUF2799 domain-containing protein | 1.441679377 | 0.0292855 | 0.0502765 |
| FQU82_RS18825 | zigA | zinc metallochaperone GTPase ZigA | 1.177771873 | 0.0293509 | 0.0503644 |
| FQU82_RS06885 | gap | type I glyceraldehyde-3-phosphate dehydrogenase | -0.376405328 | 0.0293663 | 0.0503666 |
| FQU82_RS15775 | prpF | 2-methylaconitate cis-trans isomerase PrpF | 0.213905213 | 0.029428 | 0.050448 |
| FQU82_RS10550 | FQU82_RS10550 | SDR family NAD(P)-dependent oxidoreductase | 1.446799693 | 0.029487 | 0.0505248 |
| FQU82_RS01300 | FQU82_RS01300 | zinc ABC transporter substrate-binding protein | -0.472849771 | 0.0296485 | 0.050777 |
| FQU82_RS05075 | FQU82_RS05075 | SDR family oxidoreductase | 1.083339576 | 0.0298636 | 0.0511208 |
| FQU82_RS09270 | FQU82_RS09270 | hypothetical protein | 3.364292861 | 0.0299132 | 0.0511809 |
| FQU82_RS15130 | radA | DNA repair protein RadA | 0.234831422 | 0.03008 | 0.0514416 |
| FQU82_RS14910 | tolA | cell envelope integrity protein TolA | -0.42615696 | 0.0302794 | 0.0517577 |
| FQU82_RS07610 | FQU82_RS07610 | O-methyltransferase | -0.620175186 | 0.0307703 | 0.0525714 |
| FQU82_RS12325 | nfsB | oxygen-insensitive NAD(P)H nitroreductase | 0.533602426 | 0.0311574 | 0.0532072 |
| FQU82_RS10490 | FQU82_RS10490 | fimbria/pilus outer membrane usher protein | 0.686572218 | 0.0314733 | 0.0537208 |
| FQU82_RS14680 | FQU82_RS14680 | hypothetical protein | 0.471630198 | 0.0315376 | 0.0538047 |
| FQU82_RS01775 | FQU82_RS01775 | TolC family outer membrane protein | 1.117284729 | 0.0317807 | 0.0541934 |
| FQU82_RS18630 | gshA | glutamate--cysteine ligase | -0.272172701 | 0.031808 | 0.0542141 |
| FQU82_RS11225 | FQU82_RS11225 | carbohydrate porin | 0.807840654 | 0.0319782 | 0.0544779 |
| FQU82_RS05145 | FQU82_RS05145 | helix-turn-helix transcriptional regulator | 2.219840865 | 0.0322679 | 0.0549452 |
| FQU82_RS00165 | FQU82_RS00165 | VTT domain-containing protein | -0.753431764 | 0.032305 | 0.0549819 |
| FQU82_RS01090 | mmsB | 3-hydroxyisobutyrate dehydrogenase | 0.79575346 | 0.0323396 | 0.0550144 |
| FQU82_RS06955 | FQU82_RS06955 | hypothetical protein | 2.876274414 | 0.032484 | 0.0552337 |
| FQU82_RS18005 | FQU82_RS18005 | AraC family transcriptional regulator | 0.932949734 | 0.0325477 | 0.0553156 |
| Novel00038 | - | PF03447:Homoserine dehydrogenase, NAD binding domain | -0.885171027 | 0.0325849 | 0.0553522 |
| FQU82_RS16365 | FQU82_RS16365 | glycerophosphodiester phosphodiesterase | -0.383275299 | 0.0329837 | 0.0560029 |
| FQU82_RS14010 | trpC | indole-3-glycerol phosphate synthase TrpC | -0.302217159 | 0.0335825 | 0.0569924 |
| FQU82_RS05135 | def | peptide deformylase | 0.476381296 | 0.0336682 | 0.0571105 |
| FQU82_RS11900 | nirB | nitrite reductase large subunit NirB | 0.947277169 | 0.0338861 | 0.0574526 |
| FQU82_RS08595 | FQU82_RS08595 | amino acid ABC transporter ATP-binding protein | 0.65058275 | 0.0342079 | 0.0579707 |
| FQU82_RS03395 | FQU82_RS03395 | NADAR family protein | -0.95974717 | 0.0343339 | 0.0581564 |
| FQU82_RS05255 | FQU82_RS05255 | arylesterase | 0.519389329 | 0.0344786 | 0.0583628 |
| FQU82_RS16945 | FQU82_RS16945 | YcxB family protein | -0.603062398 | 0.0345006 | 0.0583628 |
| FQU82_RS12660 | FQU82_RS12660 | alpha-E domain-containing protein | 0.929406889 | 0.034505 | 0.0583628 |
| FQU82_RS10085 | FQU82_RS10085 | thiamine pyrophosphate-dependent dehydrogenase E1 component subunit alpha | -1.078323242 | 0.0345955 | 0.058488 |
| FQU82_RS08680 | FQU82_RS08680 | PLP-dependent aminotransferase family protein | 0.644411478 | 0.0346489 | 0.0585503 |
| FQU82_RS18935 | FQU82_RS18935 | HIT family protein | 0.245108187 | 0.0347934 | 0.0587666 |
| FQU82_RS08250 | FQU82_RS08250 | carboxymuconolactone decarboxylase family protein | 0.802121482 | 0.0348327 | 0.058805 |
| FQU82_RS02350 | tusA | sulfurtransferase TusA | 0.826656823 | 0.0348538 | 0.0588127 |
| FQU82_RS14565 | wecB | UDP-N-acetylglucosamine 2-epimerase (non-hydrolyzing) | 0.336168021 | 0.0349007 | 0.0588639 |
| FQU82_RS08385 | FQU82_RS08385 | 3-oxoacid CoA-transferase subunit B | -0.749182993 | 0.0351959 | 0.0593335 |
| FQU82_RS16120 | FQU82_RS16120 | NADH:flavin oxidoreductase/NADH oxidase | 0.397464358 | 0.035323 | 0.0595197 |
| Novel00268 | - | PF01741:Large-conductance mechanosensitive channel, MscL | 1.069520763 | 0.0353775 | 0.0595831 |
| FQU82_RS15045 | FQU82_RS15045 | ABC transporter permease | -0.56320493 | 0.0355588 | 0.0598602 |
| FQU82_RS11290 | ftsB | cell division protein FtsB | 0.353341641 | 0.03571 | 0.0600862 |
| FQU82_RS12930 | csuAB | Csu fimbrial major subunit CsuAB | -1.514295388 | 0.035787 | 0.0601873 |
| FQU82_RS16725 | FQU82_RS16725 | symmetrical bis(5'-nucleosyl)-tetraphosphatase | -0.240960689 | 0.0358626 | 0.0602859 |
| FQU82_RS03300 | FQU82_RS03300 | bifunctional (p)ppGpp synthetase/guanosine-3',5'-bis(diphosphate) 3'-pyrophosphohydrolase | 0.151294446 | 0.0359314 | 0.0603729 |
| FQU82_RS02110 | FQU82_RS02110 | DMT family transporter | -0.465044869 | 0.0359828 | 0.0604306 |
| FQU82_RS10370 | modB | molybdate ABC transporter permease subunit | 1.056426059 | 0.0361919 | 0.0607531 |
| FQU82_RS15270 | FQU82_RS15270 | diacylglycerol kinase | 1.12418394 | 0.0366346 | 0.0614673 |
| FQU82_RS14760 | FQU82_RS14760 | PaaI family thioesterase | 0.894121423 | 0.0367817 | 0.0616819 |
| Novel00003 | - | PF19582:Solute-binding protein AdeT 1/2 | 0.672259884 | 0.0367973 | 0.0616819 |
| FQU82_RS05130 | FQU82_RS05130 | MFS transporter | 0.624789073 | 0.0369314 | 0.0618775 |
| Novel00180 | - | PF05193:Peptidase M16 inactive domain|PF08367:Peptidase M16C associated | 0.5304492 | 0.0369735 | 0.0619189 |
| FQU82_RS01855 | FQU82_RS01855 | phosphoglycolate phosphatase | 0.339331718 | 0.0374502 | 0.0626876 |
| FQU82_RS17335 | FQU82_RS17335 | hypothetical protein | -0.467690383 | 0.0375686 | 0.0628562 |
| Novel00334 | - | PF00501:AMP-binding enzyme|PF16177:Acetyl-coenzyme A synthetase N-terminus | 0.814378223 | 0.0376984 | 0.0630436 |
| FQU82_RS18625 | FQU82_RS18625 | disulfide bond formation protein B | -0.447321619 | 0.0380683 | 0.0636324 |
| FQU82_RS07225 | FQU82_RS07225 | hypothetical protein | -1.417304903 | 0.0384212 | 0.064192 |
| FQU82_RS05445 | FQU82_RS05445 | membrane protein | 0.232883102 | 0.0384401 | 0.0641933 |
| FQU82_RS17290 | FQU82_RS17290 | hypothetical protein | 0.938329757 | 0.0385303 | 0.0643137 |
| FQU82_RS16800 | FQU82_RS16800 | acyl-CoA dehydrogenase family protein | 0.414650664 | 0.0393494 | 0.0656501 |
| FQU82_RS17740 | FQU82_RS17740 | pilus assembly protein PilM | 0.33354067 | 0.039447 | 0.065782 |
| FQU82_RS05355 | FQU82_RS05355 | hypothetical protein | -0.753633297 | 0.0397176 | 0.0662023 |
| FQU82_RS05200 | FQU82_RS05200 | D-serine ammonia-lyase | 0.856340504 | 0.0397436 | 0.0662145 |
| FQU82_RS06005 | FQU82_RS06005 | ABC transporter permease | -0.825808633 | 0.0399699 | 0.0665603 |
| FQU82_RS05995 | FQU82_RS05995 | extracellular solute-binding protein | -0.589195171 | 0.0400583 | 0.0666763 |
| Novel00242 | - | - | 1.05742695 | 0.0402533 | 0.0669695 |
| FQU82_RS10605 | FQU82_RS10605 | HD domain-containing protein | 2.173632074 | 0.0405231 | 0.0673868 |
| FQU82_RS10725 | FQU82_RS10725 | nicotinate phosphoribosyltransferase | -0.67807173 | 0.0406301 | 0.067533 |
| FQU82_RS10785 | FQU82_RS10785 | NAD(P)-dependent oxidoreductase | 1.135010439 | 0.0407916 | 0.0677697 |
| FQU82_RS09310 | FQU82_RS09310 | hypothetical protein | 1.431372962 | 0.0408106 | 0.0677697 |
| FQU82_RS11110 | FQU82_RS11110 | flavin reductase family protein | 1.248199488 | 0.0408373 | 0.0677824 |
| FQU82_RS08490 | FQU82_RS08490 | hypothetical protein | 0.937497965 | 0.0415327 | 0.0689043 |
| FQU82_RS02215 | hisIE | bifunctional phosphoribosyl-AMP cyclohydrolase/phosphoribosyl-ATP diphosphatase HisIE | -0.235963465 | 0.0415527 | 0.0689053 |
| FQU82_RS02915 | clpP | ATP-dependent Clp endopeptidase proteolytic subunit ClpP | 0.169575076 | 0.0416745 | 0.069075 |
| FQU82_RS01820 | FQU82_RS01820 | membrane protein | -0.349802197 | 0.0421814 | 0.0698827 |
| FQU82_RS11120 | FQU82_RS11120 | SDR family oxidoreductase | 1.286649399 | 0.0422581 | 0.0699771 |
| Novel00099 | - | PF01613:Flavin reductase like domain|PF01717:Cobalamin-independent synthase, Catalytic domain | -0.524563086 | 0.0425227 | 0.0703824 |
| FQU82_RS05525 | FQU82_RS05525 | hypothetical protein | 0.812477694 | 0.042835 | 0.0708663 |
| Novel00367 | - | - | 1.133233507 | 0.0429652 | 0.0710486 |
| FQU82_RS11610 | FQU82_RS11610 | SulP family inorganic anion transporter | -0.412516338 | 0.0432546 | 0.0714938 |
| FQU82_RS03200 | FQU82_RS03200 | tRNA threonylcarbamoyladenosine dehydratase | -0.479979103 | 0.0433919 | 0.0716811 |
| FQU82_RS02210 | FQU82_RS02210 | AAA family ATPase | 0.246713991 | 0.0434082 | 0.0716811 |
| FQU82_RS18815 | hutC | histidine utilization repressor | 0.420733492 | 0.0434403 | 0.0716903 |
| FQU82_RS14865 | FQU82_RS14865 | multidrug effflux MFS transporter | 0.367268835 | 0.0434542 | 0.0716903 |
| FQU82_RS17195 | FQU82_RS17195 | globin domain-containing protein | 0.231136294 | 0.0435343 | 0.0717892 |
| FQU82_RS02015 | fadB | fatty acid oxidation complex subunit alpha FadB | -0.145137394 | 0.0436307 | 0.0719147 |
| FQU82_RS13825 | FQU82_RS13825 | AraC family transcriptional regulator | 0.858372441 | 0.0436928 | 0.0719837 |
| Novel00030 | - | PF00892:EamA-like transporter family | 1.448989431 | 0.0438007 | 0.0721112 |
| Novel00363 | - | PF00311:Phosphoenolpyruvate carboxylase | 0.547056883 | 0.0438108 | 0.0721112 |
| FQU82_RS03165 | FQU82_RS03165 | iron-containing redox enzyme family protein | 0.579448283 | 0.0438807 | 0.0721928 |
| FQU82_RS13195 | FQU82_RS13195 | metal-dependent hydrolase | 1.316370402 | 0.0439146 | 0.0722151 |
| FQU82_RS09395 | FQU82_RS09395 | Maf family protein | -0.683096787 | 0.0439634 | 0.072262 |
| FQU82_RS14140 | bauC | ferric acinetobactin ABC transporter permease subunit BauC | -1.879014968 | 0.0442567 | 0.0727104 |
| FQU82_RS06925 | FQU82_RS06925 | GNAT family N-acetyltransferase | 0.807417468 | 0.044738 | 0.0734671 |
| FQU82_RS04325 | FQU82_RS04325 | tRNA-Gln | -1.070800009 | 0.0452226 | 0.0742287 |
| FQU82_RS13200 | yaaA | peroxide stress protein YaaA | 0.288138289 | 0.0456345 | 0.0748701 |
| FQU82_RS06540 | FQU82_RS06540 | SDR family NAD(P)-dependent oxidoreductase | 0.612042323 | 0.0457807 | 0.0750752 |
| FQU82_RS04955 | FQU82_RS04955 | hypothetical protein | -1.535669473 | 0.0461099 | 0.0755803 |
| FQU82_RS05885 | FQU82_RS05885 | tyrosine-type recombinase/integrase | 0.483305179 | 0.046392 | 0.0760075 |
| FQU82_RS07805 | FQU82_RS07805 | helix-turn-helix domain-containing protein | 1.003941327 | 0.0465613 | 0.0762497 |
| FQU82_RS03060 | FQU82_RS03060 | 4'-phosphopantetheinyl transferase superfamily protein | -1.783450324 | 0.0466457 | 0.0763528 |
| FQU82_RS03875 | FQU82_RS03875 | hypothetical protein | -0.398059062 | 0.0467373 | 0.0764674 |
| FQU82_RS03645 | FQU82_RS03645 | class I SAM-dependent methyltransferase | -0.591450485 | 0.046785 | 0.0765103 |
| Novel00088 | - | - | -1.20561673 | 0.0468081 | 0.0765128 |
| FQU82_RS16880 | FQU82_RS16880 | lytic transglycosylase domain-containing protein | 0.167966826 | 0.0470829 | 0.0769266 |
| Novel00241 | - | PF13365:Trypsin-like peptidase domain | 0.744642233 | 0.0478639 | 0.0781667 |
| FQU82_RS13205 | FQU82_RS13205 | hydrolase | -0.340938622 | 0.047898 | 0.0781864 |
| FQU82_RS01010 | FQU82_RS01010 | hypothetical protein | 0.574659026 | 0.0483697 | 0.0789202 |
| FQU82_RS16470 | FQU82_RS16470 | glycosyltransferase family 25 protein | 0.45131446 | 0.0489713 | 0.0798651 |
| FQU82_RS17280 | mlaE | lipid asymmetry maintenance ABC transporter permease subunit MlaE | -1.29945113 | 0.0491208 | 0.0800721 |
| FQU82_RS07735 | FQU82_RS07735 | urea amidolyase family protein | -0.300555494 | 0.0491551 | 0.0800913 |
| FQU82_RS04050 | FQU82_RS04050 | LysE family translocator | -0.714976648 | 0.0494431 | 0.0805016 |
| FQU82_RS02400 | FQU82_RS02400 | GFA family protein | 0.3030582 | 0.0494523 | 0.0805016 |
| FQU82_RS08550 | FQU82_RS08550 | LysE family translocator | 1.047058039 | 0.0497566 | 0.0809599 |
| FQU82_RS04200 | FQU82_RS04200 | 8-amino-7-oxononanoate synthase | 0.426645885 | 0.0499032 | 0.0811612 |

**Table S3 The DEGs identified in *A. baumannii* after IPM treatment**

| **Gene_id** | **Gene name** | **Gene description** | **log2FC (IPM/Control)** | **pvalue** | **padj** |
| --- | --- | --- | --- | --- | --- |
| FQU82_RS04710 | ssrA | transfer-messenger RNA | 0.558383234 | 1.25E-21 | 2.01E-18 |
| FQU82_RS18515 | FQU82_RS18515 | DUF2147 domain-containing protein | 0.488530208 | 6.28E-14 | 5.07E-11 |
| FQU82_RS04395 | rnpB | RNase P RNA component class A | 0.479704807 | 1.81E-12 | 9.75E-10 |
| FQU82_RS01970 | htpG | molecular chaperone HtpG | 0.624638493 | 1.80E-11 | 7.25E-09 |
| FQU82_RS11495 | FQU82_RS11495 | hypothetical protein | 0.783183872 | 4.16E-11 | 1.34E-08 |
| FQU82_RS06935 | clpB | ATP-dependent chaperone ClpB | 0.468650362 | 1.52E-10 | 3.61E-08 |
| FQU82_RS09850 | FQU82_RS09850 | TonB-dependent receptor | -0.487064933 | 1.56E-10 | 3.61E-08 |
| FQU82_RS14810 | carO | ornithine uptake porin CarO type 1 | 0.54749239 | 2.64E-10 | 5.34E-08 |
| FQU82_RS02705 | FQU82_RS02705 | hypothetical protein | 0.467165772 | 1.78E-09 | 3.20E-07 |
| FQU82_RS02905 | FQU82_RS02905 | TonB-dependent siderophore receptor | -0.410458269 | 2.06E-09 | 3.32E-07 |
| FQU82_RS01405 | FQU82_RS01405 | TonB-dependent copper receptor | -0.47998492 | 2.73E-09 | 4.01E-07 |
| FQU82_RS14620 | FQU82_RS14620 | glyceraldehyde-3-phosphate dehydrogenase | -0.321798105 | 2.19E-08 | 2.94E-06 |
| FQU82_RS02725 | FQU82_RS02725 | coniferyl aldehyde dehydrogenase | 0.34810569 | 7.51E-08 | 9.33E-06 |
| FQU82_RS03250 | FQU82_RS03250 | NAD(P)(+) transhydrogenase (Re/Si-specific) subunit beta | -0.390286028 | 1.27E-07 | 1.46E-05 |
| FQU82_RS16605 | FQU82_RS16605 | rhodanese-like domain-containing protein | 0.641826042 | 1.49E-07 | 1.53E-05 |
| FQU82_RS10500 | FQU82_RS10500 | fimbrial protein | 0.392486426 | 1.51E-07 | 1.53E-05 |
| FQU82_RS04010 | FQU82_RS04010 | hypothetical protein | 0.653016593 | 1.90E-07 | 1.80E-05 |
| FQU82_RS04335 | ispE | 4-(cytidine 5'-diphospho)-2-C-methyl-D-erythritol kinase | 0.425634195 | 2.12E-07 | 1.90E-05 |
| FQU82_RS13165 | FQU82_RS13165 | cold-shock protein | 0.465536933 | 2.36E-07 | 2.01E-05 |
| FQU82_RS15080 | FQU82_RS15080 | FAD-binding protein | -0.271270345 | 4.23E-07 | 3.42E-05 |
| FQU82_RS03385 | rpmI | 50S ribosomal protein L35 | 0.289567978 | 5.47E-07 | 4.21E-05 |
| FQU82_RS09545 | cmk | (d)CMP kinase | 0.458274122 | 8.30E-07 | 6.10E-05 |
| FQU82_RS18265 | FQU82_RS18265 | TetR/AcrR family transcriptional regulator | 0.326412375 | 9.63E-07 | 6.76E-05 |
| FQU82_RS02275 | rpsO | 30S ribosomal protein S15 | 0.315068081 | 1.25E-06 | 8.44E-05 |
| FQU82_RS14815 | dapD | 2,3,4,5-tetrahydropyridine-2,6-dicarboxylate N-succinyltransferase | -0.297135385 | 1.37E-06 | 8.82E-05 |
| FQU82_RS18255 | FQU82_RS18255 | DUF1304 domain-containing protein | 0.624227773 | 1.61E-06 | 0.0001001 |
| FQU82_RS18260 | FQU82_RS18260 | zinc-binding dehydrogenase | 0.341368905 | 1.83E-06 | 0.0001067 |
| FQU82_RS02745 | FQU82_RS02745 | MotA/TolQ/ExbB proton channel family protein | -0.381382836 | 1.85E-06 | 0.0001067 |
| FQU82_RS09325 | putA | trifunctional transcriptional regulator/proline dehydrogenase/L-glutamate gamma-semialdehyde dehydrogenase | -0.30170026 | 2.76E-06 | 0.0001537 |
| FQU82_RS18380 | FQU82_RS18380 | putative porin | 0.245338552 | 3.64E-06 | 0.0001957 |
| FQU82_RS15965 | FQU82_RS15965 | PepSY domain-containing protein | -0.501562501 | 4.03E-06 | 0.00021 |
| FQU82_RS15300 | FQU82_RS15300 | phosphoenolpyruvate carboxykinase (GTP) | -0.281321611 | 4.26E-06 | 0.0002149 |
| FQU82_RS15610 | rpmA | 50S ribosomal protein L27 | 0.342242487 | 5.07E-06 | 0.0002483 |
| FQU82_RS03420 | FQU82_RS03420 | integration host factor subunit alpha | 0.606247483 | 7.98E-06 | 0.0003788 |
| FQU82_RS11300 | eno | phosphopyruvate hydratase | -0.252246966 | 8.32E-06 | 0.0003838 |
| FQU82_RS15085 | FQU82_RS15085 | electron transfer flavoprotein subunit beta/FixA family protein | -0.239977757 | 9.49E-06 | 0.0004256 |
| FQU82_RS13375 | adeG | multidrug efflux RND transporter permease subunit AdeG | 0.593086085 | 9.91E-06 | 0.0004324 |
| FQU82_RS09935 | FQU82_RS09935 | TonB-dependent siderophore receptor | -0.613384849 | 1.07E-05 | 0.0004548 |
| FQU82_RS17330 | FQU82_RS17330 | acyl-CoA dehydrogenase C-terminal domain-containing protein | -0.289939501 | 1.12E-05 | 0.0004649 |
| FQU82_RS03390 | rplT | 50S ribosomal protein L20 | 0.264855392 | 1.59E-05 | 0.0006406 |
| FQU82_RS09770 | FQU82_RS09770 | HU family DNA-binding protein | 0.450545304 | 1.67E-05 | 0.0006566 |
| FQU82_RS12045 | FQU82_RS12045 | 3-oxoacyl-ACP reductase | -0.266160246 | 1.98E-05 | 0.0007605 |
| FQU82_RS05925 | FQU82_RS05925 | hypothetical protein | 0.497808008 | 2.35E-05 | 0.0008845 |
| FQU82_RS09815 | FQU82_RS09815 | IucA/IucC family protein | -0.765629743 | 3.14E-05 | 0.0011536 |
| FQU82_RS00410 | ileS | isoleucine--tRNA ligase | -0.249968165 | 3.50E-05 | 0.0012569 |
| FQU82_RS15885 | gatA | Asp-tRNA(Asn)/Glu-tRNA(Gln) amidotransferase subunit GatA | -0.281879598 | 4.23E-05 | 0.0014867 |
| FQU82_RS05935 | FQU82_RS05935 | cation diffusion facilitator family transporter | 0.343486981 | 4.34E-05 | 0.0014924 |
| FQU82_RS09550 | rpsA | 30S ribosomal protein S1 | -0.223305166 | 4.87E-05 | 0.0016385 |
| FQU82_RS09335 | putP | sodium/proline symporter PutP | -0.291329942 | 5.23E-05 | 0.0017254 |
| FQU82_RS01830 | FQU82_RS01830 | H-NS histone family protein | 0.39490728 | 6.38E-05 | 0.0020236 |
| Novel00262 | - | PF01016:Ribosomal L27 protein|PF00829:Ribosomal prokaryotic L21 protein | -0.598043339 | 6.39E-05 | 0.0020236 |
| FQU82_RS14370 | FQU82_RS14370 | aspartate aminotransferase family protein | -0.225974195 | 6.62E-05 | 0.0020566 |
| FQU82_RS17795 | FQU82_RS17795 | hypothetical protein | 0.331269077 | 7.44E-05 | 0.0022663 |
| FQU82_RS03495 | FQU82_RS03495 | organic hydroperoxide resistance protein | 0.425313644 | 8.58E-05 | 0.0025288 |
| FQU82_RS14375 | FQU82_RS14375 | diaminobutyrate--2-oxoglutarate transaminase | -0.226768025 | 8.87E-05 | 0.0025288 |
| FQU82_RS01400 | FQU82_RS01400 | DUF2946 family protein | -0.474128433 | 8.91E-05 | 0.0025288 |
| FQU82_RS17875 | FQU82_RS17875 | SEL1-like repeat protein | 0.332348913 | 8.93E-05 | 0.0025288 |
| FQU82_RS15505 | sdhA | succinate dehydrogenase flavoprotein subunit | -0.227759423 | 0.0001034 | 0.0028349 |
| FQU82_RS17655 | bfr | bacterioferritin | 0.324554106 | 0.0001036 | 0.0028349 |
| FQU82_RS18030 | FQU82_RS18030 | hypothetical protein | -0.356505994 | 0.0001117 | 0.0030078 |
| FQU82_RS07180 | FQU82_RS07180 | cold-shock protein | 0.483517567 | 0.0001202 | 0.0031812 |
| FQU82_RS00125 | tyrS | tyrosine--tRNA ligase | -0.285193447 | 0.0001292 | 0.0033522 |
| FQU82_RS04260 | acpP | acyl carrier protein | 0.544259241 | 0.0001308 | 0.0033522 |
| FQU82_RS17525 | serA | phosphoglycerate dehydrogenase | -0.243566792 | 0.0001667 | 0.0042054 |
| FQU82_RS02470 | FQU82_RS02470 | long-chain-acyl-CoA synthetase | -0.30042877 | 0.0001715 | 0.0042619 |
| FQU82_RS12680 | FQU82_RS12680 | hypothetical protein | 0.46371885 | 0.0001984 | 0.0048538 |
| FQU82_RS01715 | FQU82_RS01715 | ferrous iron transporter B | -0.25247049 | 0.0002477 | 0.0059701 |
| FQU82_RS00140 | erpA | iron-sulfur cluster insertion protein ErpA | 0.458383105 | 0.0002561 | 0.0060812 |
| FQU82_RS16870 | FQU82_RS16870 | malate dehydrogenase | -0.239379804 | 0.0002749 | 0.0064344 |
| FQU82_RS17065 | rpmJ | 50S ribosomal protein L36 | 0.269083574 | 0.0002887 | 0.0066315 |
| FQU82_RS15550 | FQU82_RS15550 | DUF493 domain-containing protein | 0.446694905 | 0.0002915 | 0.0066315 |
| FQU82_RS17630 | rpoZ | DNA-directed RNA polymerase subunit omega | 0.42883929 | 0.0003214 | 0.0072101 |
| FQU82_RS14475 | FQU82_RS14475 | LysR family transcriptional regulator | 0.368082372 | 0.0003486 | 0.0076216 |
| Novel00158 | - | PF00171:Aldehyde dehydrogenase family|PF01619:Proline dehydrogenase|PF14850:DNA-binding domain of Proline dehydrogenase | -0.793399071 | 0.000351 | 0.0076216 |
| FQU82_RS18840 | fahA | fumarylacetoacetase | 0.319563377 | 0.0003541 | 0.0076216 |
| FQU82_RS00920 | FQU82_RS00920 | amino acid aminotransferase | -0.271262346 | 0.0003587 | 0.0076216 |
| FQU82_RS12415 | FQU82_RS12415 | bifunctional aconitate hydratase 2/2-methylisocitrate dehydratase | -0.19721882 | 0.0003893 | 0.0081653 |
| FQU82_RS03910 | FQU82_RS03910 | hypothetical protein | 0.378342458 | 0.0004094 | 0.0084709 |
| FQU82_RS18765 | glmS | glutamine--fructose-6-phosphate transaminase (isomerizing) | -0.240685235 | 0.0004144 | 0.0084709 |
| FQU82_RS13370 | adeF | multidrug efflux RND transporter periplasmic adaptor subunit AdeF | 1.028242899 | 0.000418 | 1 |
| FQU82_RS01680 | FQU82_RS01680 | response regulator | 0.319710139 | 0.0004303 | 0.0086865 |
| FQU82_RS15805 | FQU82_RS15805 | undecaprenyl-diphosphate phosphatase | -0.395248178 | 0.0004397 | 0.0087677 |
| FQU82_RS12700 | purH | bifunctional phosphoribosylaminoimidazolecarboxamide formyltransferase/IMP cyclohydrolase | -0.258321682 | 0.0004722 | 0.0092997 |
| FQU82_RS13910 | FQU82_RS13910 | NADP-dependent malic enzyme | -0.229702692 | 0.0005028 | 0.0097835 |
| FQU82_RS15485 | FQU82_RS15485 | 2-oxoglutarate dehydrogenase E1 component | -0.201553677 | 0.0005209 | 0.010015 |
| FQU82_RS19140 | FQU82_RS19140 | hypothetical protein | 0.715259565 | 0.0005379 | 0.0101161 |
| FQU82_RS03240 | FQU82_RS03240 | Re/Si-specific NAD(P)(+) transhydrogenase subunit alpha | -0.327072036 | 0.0005387 | 0.0101161 |
| FQU82_RS11710 | bamA | outer membrane protein assembly factor BamA | -0.206783927 | 0.0005769 | 0.01071 |
| FQU82_RS17920 | FQU82_RS17920 | acetyl-CoA hydrolase/transferase family protein | -0.25327758 | 0.0005983 | 0.0109805 |
| FQU82_RS03145 | ilvC | ketol-acid reductoisomerase | -0.211358867 | 0.0006134 | 0.0111316 |
| FQU82_RS15460 | trpS | tryptophan--tRNA ligase | -0.335676467 | 0.0006317 | 0.011335 |
| FQU82_RS09405 | FQU82_RS09405 | phosphoglycerate kinase | -0.241990176 | 0.0006573 | 0.0116656 |
| FQU82_RS06045 | argG | argininosuccinate synthase | -0.25470269 | 0.000685 | 0.0120241 |
| FQU82_RS11545 | FQU82_RS11545 | hypothetical protein | 0.50906185 | 0.0007376 | 0.012809 |
| FQU82_RS16215 | FQU82_RS16215 | nitrite/sulfite reductase | -0.299739016 | 0.0007603 | 0.0130628 |
| FQU82_RS03930 | nuoC | NADH-quinone oxidoreductase subunit C/D | -0.22321829 | 0.0008057 | 0.0136967 |
| FQU82_RS03890 | FQU82_RS03890 | ribonucleoside-diphosphate reductase subunit alpha | -0.193775374 | 0.00085 | 0.0142988 |
| FQU82_RS01225 | guaA | glutamine-hydrolyzing GMP synthase | -0.219298934 | 0.000893 | 0.0148465 |
| FQU82_RS03425 | FQU82_RS03425 | hypothetical protein | 0.289855023 | 0.0009053 | 0.0148465 |
| FQU82_RS15480 | odhB | 2-oxoglutarate dehydrogenase complex dihydrolipoyllysine-residue succinyltransferase | -0.181161312 | 0.0009101 | 0.0148465 |
| FQU82_RS04065 | FQU82_RS04065 | hypothetical protein | 0.318104178 | 0.0009223 | 0.0148725 |
| FQU82_RS12310 | FQU82_RS12310 | peptidylprolyl isomerase | -0.241864002 | 0.0009361 | 0.0148725 |
| FQU82_RS00310 | FQU82_RS00310 | hypothetical protein | 0.449016651 | 0.0009393 | 0.0148725 |
| FQU82_RS09875 | FQU82_RS09875 | HAD family hydrolase | 0.425470849 | 0.000955 | 0.0149743 |
| Novel00199 | - | PF02896:PEP-utilising enzyme, PEP-binding domain|PF00391:PEP-utilising enzyme, mobile domain|PF01326:Pyruvate phosphate dikinase, AMP/ATP-binding domain|PF14237:GYF domain 2 | -0.711513356 | 0.0009838 | 0.0152772 |
| FQU82_RS17440 | FQU82_RS17440 | Glu/Leu/Phe/Val dehydrogenase | -0.194874418 | 0.0010308 | 0.015855 |
| Novel00282 | - | PF00380:Ribosomal protein S9/S16|PF02798:Glutathione S-transferase, N-terminal domain | -0.76689161 | 0.0010601 | 0.0161521 |
| FQU82_RS17880 | ettA | energy-dependent translational throttle protein EttA | -0.210891647 | 0.0011055 | 0.0166865 |
| FQU82_RS18545 | FQU82_RS18545 | HPF/RaiA family ribosome-associated protein | 0.1911677 | 0.0011322 | 0.016847 |
| FQU82_RS14635 | uvrB | excinuclease ABC subunit UvrB | 0.293469608 | 0.001137 | 0.016847 |
| FQU82_RS13975 | glnA | type I glutamate--ammonia ligase | -0.222788922 | 0.0011536 | 0.0169364 |
| FQU82_RS01925 | rpoB | DNA-directed RNA polymerase subunit beta | -0.165024857 | 0.0011741 | 0.0169838 |
| FQU82_RS07720 | FQU82_RS07720 | divalent metal cation transporter | -0.448050757 | 0.0011778 | 0.0169838 |
| FQU82_RS17700 | gltB | glutamate synthase large subunit | -0.196070032 | 0.0011912 | 0.0170245 |
| FQU82_RS18295 | FQU82_RS18295 | cation acetate symporter | -0.288211166 | 0.001256 | 0.0177936 |
| FQU82_RS00895 | FQU82_RS00895 | phosphomannomutase/phosphoglucomutase | -0.293716815 | 0.0013178 | 0.0185067 |
| FQU82_RS07585 | FQU82_RS07585 | type I secretion system permease/ATPase | -0.32941051 | 0.001349 | 0.0187813 |
| FQU82_RS09945 | FQU82_RS09945 | CerR family C-terminal domain-containing protein | 1.127708555 | 0.0013742 | 1 |
| Novel00129 | - | PF02190:ATP-dependent protease La (LON) substrate-binding domain|PF05362:Lon protease (S16) C-terminal proteolytic domain|PF00004:ATPase family associated with various cellular activities (AAA) | -0.71369364 | 0.0014161 | 0.0195467 |
| FQU82_RS01960 | FQU82_RS01960 | OmpW family outer membrane protein | -0.369456824 | 0.0014524 | 0.0198783 |
| FQU82_RS16155 | typA | translational GTPase TypA | -0.195114979 | 0.0015117 | 0.0205158 |
| FQU82_RS02910 | tig | trigger factor | -0.198594187 | 0.0016392 | 0.0220606 |
| FQU82_RS14235 | rpmE | 50S ribosomal protein L31 | 0.254760031 | 0.0017448 | 0.0232877 |
| FQU82_RS00465 | FQU82_RS00465 | RcnB family protein | 1.298683517 | 0.0017778 | 1 |
| Novel00143 | - | PF00578:AhpC/TSA family | -1.066662452 | 0.0018023 | 1 |
| FQU82_RS14115 | basD | acinetobactin non-ribosomal peptide synthetase subunit BasD | -1.298700211 | 0.0018287 | 1 |
| FQU82_RS18160 | FQU82_RS18160 | NAD-dependent succinate-semialdehyde dehydrogenase | -0.326887077 | 0.0019751 | 0.0261461 |
| FQU82_RS15520 | gltA | citrate synthase | -0.202713261 | 0.0020137 | 0.0264398 |
| Novel00234 | - | PF02800:Glyceraldehyde 3-phosphate dehydrogenase, C-terminal domain|PF00044:Glyceraldehyde 3-phosphate dehydrogenase, NAD binding domain | -0.89152879 | 0.0020519 | 1 |
| FQU82_RS12605 | cyoB | cytochrome o ubiquinol oxidase subunit I | -0.178135185 | 0.0020528 | 0.0267359 |
| Novel00231 | - | PF03971:Monomeric isocitrate dehydrogenase | -0.611944513 | 0.0020817 | 0.0268285 |
| Novel00056 | - | PF20833:RNase E/G, Thioredoxin-like domain|PF10150:Ribonuclease E/G family | -0.61824819 | 0.0020931 | 0.0268285 |
| FQU82_RS17965 | hisA | 1-(5-phosphoribosyl)-5-[(5-phosphoribosylamino)methylideneamino]imidazole-4-carboxamide isomerase | -0.256188428 | 0.0021882 | 0.0278263 |
| FQU82_RS15850 | FQU82_RS15850 | hypothetical protein | 0.56957615 | 0.0023041 | 0.0290717 |
| FQU82_RS13890 | ahcY | adenosylhomocysteinase | -0.216117394 | 0.0023866 | 0.029302 |
| FQU82_RS07730 | FQU82_RS07730 | putative hydro-lyase | -0.565695517 | 0.0023885 | 0.029302 |
| FQU82_RS18935 | FQU82_RS18935 | HIT family protein | 0.338727128 | 0.0024038 | 0.029302 |
| FQU82_RS10475 | FQU82_RS10475 | S-(hydroxymethyl)glutathione dehydrogenase/class III alcohol dehydrogenase | -0.299773651 | 0.0024108 | 0.029302 |
| FQU82_RS13330 | yegQ | tRNA 5-hydroxyuridine modification protein YegQ | -0.405226595 | 0.0024131 | 0.029302 |
| FQU82_RS00215 | yidC | membrane protein insertase YidC | -0.178586944 | 0.0024896 | 0.0300053 |
| FQU82_RS15075 | gyrA | DNA gyrase subunit A | -0.196424496 | 0.00254 | 0.0303864 |
| FQU82_RS18280 | omp33-36 | porin Omp33-36 | -0.175181742 | 0.0025905 | 0.0305525 |
| FQU82_RS16555 | secD | protein translocase subunit SecD | -0.186742994 | 0.0025922 | 0.0305525 |
| FQU82_RS05305 | rubB | rubredoxin reductase RubB | -0.325305443 | 0.0026107 | 0.0305525 |
| FQU82_RS00090 | FQU82_RS00090 | tetratricopeptide repeat protein | 0.330651243 | 0.0026978 | 0.0311276 |
| FQU82_RS10220 | FQU82_RS10220 | aspartate ammonia-lyase | -0.492738709 | 0.0027057 | 0.0311276 |
| FQU82_RS14335 | pstA | phosphate ABC transporter permease PstA | -0.323037135 | 0.0027176 | 0.0311276 |
| FQU82_RS09680 | rpsT | 30S ribosomal protein S20 | 0.401108229 | 0.0029388 | 0.0334232 |
| FQU82_RS07620 | FQU82_RS07620 | ferredoxin family protein | 0.64460016 | 0.0030007 | 1 |
| FQU82_RS07095 | FQU82_RS07095 | heavy-metal-associated domain-containing protein | 0.387118402 | 0.0030363 | 0.0341074 |
| FQU82_RS17840 | FQU82_RS17840 | hypothetical protein | 0.48020718 | 0.0030412 | 0.0341074 |
| FQU82_RS13385 | FQU82_RS13385 | serine hydroxymethyltransferase | -0.184870232 | 0.0030861 | 0.0343729 |
| FQU82_RS08605 | FQU82_RS08605 | cysteine ABC transporter substrate-binding protein | -0.567654314 | 0.0031259 | 0.0344606 |
| FQU82_RS12730 | FQU82_RS12730 | hypothetical protein | 0.443757081 | 0.0031379 | 0.0344606 |
| FQU82_RS01690 | thrC | threonine synthase | -0.213279448 | 0.003158 | 0.0344606 |
| FQU82_RS01490 | FQU82_RS01490 | DUF2726 domain-containing protein | 0.616773586 | 0.0032053 | 0.0347416 |
| FQU82_RS03975 | nuoM | NADH-quinone oxidoreductase subunit M | -0.1744527 | 0.0032517 | 0.03501 |
| FQU82_RS06020 | FQU82_RS06020 | TonB-dependent receptor | -0.567546715 | 0.0034581 | 0.036986 |
| FQU82_RS03260 | rsfS | ribosome silencing factor | 0.237347656 | 0.0034871 | 0.0370501 |
| Novel00100 | - | PF00268:Ribonucleotide reductase, small chain | -0.814708642 | 0.0034984 | 1 |
| Novel00257 | - | PF00117:Glutamine amidotransferase class-I|PF02786:Carbamoyl-phosphate synthase L chain, ATP binding domain|PF02787:Carbamoyl-phosphate synthetase large chain, oligomerisation domain|PF02142:MGS-like domain|PF01272:Transcription elongation factor, GreA/GreB, C-term|PF03449:Transcription elongation factor, N-terminal | -0.536673613 | 0.0036399 | 0.0383058 |
| FQU82_RS08430 | FQU82_RS08430 | acetyl/propionyl/methylcrotonyl-CoA carboxylase subunit alpha | -0.325547429 | 0.0036536 | 0.0383058 |
| FQU82_RS12050 | FQU82_RS12050 | acetyl-CoA C-acetyltransferase | -0.201115022 | 0.0036764 | 0.0383058 |
| FQU82_RS16450 | aspS | aspartate--tRNA ligase | -0.181383174 | 0.0037442 | 0.0385307 |
| FQU82_RS13895 | metF | methylenetetrahydrofolate reductase [NAD(P)H] | -0.271613356 | 0.0037457 | 0.0385307 |
| FQU82_RS18415 | FQU82_RS18415 | TonB-dependent siderophore receptor | -0.374668559 | 0.0038569 | 0.039221 |
| FQU82_RS17005 | FQU82_RS17005 | TRAP transporter large permease subunit | -0.304038592 | 0.0038772 | 0.039221 |
| FQU82_RS13245 | FQU82_RS13245 | dicarboxylate/amino acid:cation symporter | -0.238514079 | 0.0038857 | 0.039221 |
| FQU82_RS02935 | FQU82_RS02935 | fumarate hydratase | -0.189966418 | 0.003924 | 0.039362 |
| FQU82_RS18365 | FQU82_RS18365 | alkene reductase | 0.247853767 | 0.0039783 | 0.0396599 |
| FQU82_RS05450 | adk | adenylate kinase | -0.174192041 | 0.0040449 | 0.0400771 |
| FQU82_RS08360 | FQU82_RS08360 | SDR family oxidoreductase | -0.663053887 | 0.0041193 | 1 |
| FQU82_RS08465 | FQU82_RS08465 | DcaP family trimeric outer membrane transporter | -0.642531279 | 0.004166 | 1 |
| FQU82_RS11425 | FQU82_RS11425 | TonB-dependent siderophore receptor | -0.345960828 | 0.0041736 | 0.040856 |
| FQU82_RS01345 | FQU82_RS01345 | F0F1 ATP synthase subunit epsilon | 0.185966055 | 0.0041741 | 0.040856 |
| Novel00226 | - | PF00903:Glyoxalase/Bleomycin resistance protein/Dioxygenase superfamily | -2.280708682 | 0.00419 | 1 |
| FQU82_RS07725 | FQU82_RS07725 | 5-oxoprolinase subunit PxpA | -0.358322797 | 0.0043996 | 0.0424622 |
| FQU82_RS02235 | ubiE | bifunctional demethylmenaquinone methyltransferase/2-methoxy-6-polyprenyl-1,4-benzoquinol methylase UbiE | -0.229340274 | 0.0044124 | 0.0424622 |
| FQU82_RS08805 | tauA | taurine ABC transporter substrate-binding protein | 0.194353147 | 0.0044391 | 0.0424622 |
| FQU82_RS15010 | FQU82_RS15010 | lipoprotein-releasing ABC transporter permease subunit | -0.241815914 | 0.0044434 | 0.0424622 |
| FQU82_RS04180 | FQU82_RS04180 | trehalose-6-phosphate synthase | -0.503484794 | 0.0045693 | 0.0434085 |
| Novel00174 | - | PF03952:Enolase, N-terminal domain|PF00113:Enolase, C-terminal TIM barrel domain|PF00793:DAHP synthetase I family | -0.852633439 | 0.0045697 | 1 |
| Novel00216 | - | PF05221:S-adenosyl-L-homocysteine hydrolase|PF00670:S-adenosyl-L-homocysteine hydrolase, NAD binding domain|PF02219:Methylenetetrahydrofolate reductase | -0.881584118 | 0.0046037 | 1 |
| FQU82_RS17900 | FQU82_RS17900 | Tex family protein | -0.193501188 | 0.0047094 | 0.0443657 |
| FQU82_RS06585 | FQU82_RS06585 | aspartate kinase | -0.19762953 | 0.004734 | 0.0443657 |
| FQU82_RS15640 | adeI | multidrug efflux RND transporter periplasmic adaptor subunit AdeI | -0.172682416 | 0.0047525 | 0.0443657 |
| FQU82_RS04970 | betB | betaine-aldehyde dehydrogenase | -0.269815791 | 0.0047844 | 0.0444069 |
| FQU82_RS18570 | FQU82_RS18570 | outer membrane protein OmpK | -0.407164816 | 0.0048591 | 0.0448427 |
| FQU82_RS03015 | ndk | nucleoside-diphosphate kinase | -0.155554805 | 0.0049209 | 0.0448449 |
| FQU82_RS03625 | FQU82_RS03625 | NUDIX hydrolase | 0.468086842 | 0.0049375 | 0.0448449 |
| FQU82_RS04245 | rpmF | 50S ribosomal protein L32 | 0.368466367 | 0.0049427 | 0.0448449 |
| FQU82_RS05195 | FQU82_RS05195 | inorganic phosphate transporter | -0.19886248 | 0.0049873 | 0.0449971 |
| FQU82_RS03265 | FQU82_RS03265 | TIM barrel protein | 0.318048887 | 0.0051549 | 0.0462507 |
| FQU82_RS14150 | basB | acinetobactin non-ribosomal peptide synthetase subunit BasB | -1.033402224 | 0.0051553 | 1 |
| FQU82_RS18805 | hutU | urocanate hydratase | 0.201839669 | 0.005299 | 0.0471341 |
| Novel00116 | - | PF07992:Pyridine nucleotide-disulphide oxidoreductase | -1.555732486 | 0.0053168 | 1 |
| FQU82_RS01545 | FQU82_RS01545 | long-chain-fatty-acid--CoA ligase | -0.171426612 | 0.0053223 | 0.0471341 |
| FQU82_RS12115 | FQU82_RS12115 | universal stress protein | 0.212622367 | 0.0053409 | 0.0471341 |
| FQU82_RS14940 | FQU82_RS14940 | RNA polymerase sigma factor | 0.301567784 | 0.0053845 | 0.0472606 |
| FQU82_RS04985 | FQU82_RS04985 | choline transporter | -0.306155092 | 0.0054761 | 0.047805 |
| FQU82_RS10725 | FQU82_RS10725 | nicotinate phosphoribosyltransferase | 0.802083212 | 0.0055821 | 1 |
| FQU82_RS01310 | atpB | F0F1 ATP synthase subunit A | -0.171836321 | 0.0058205 | 0.0505382 |
| FQU82_RS11940 | accC | acetyl-CoA carboxylase biotin carboxylase subunit | -0.22730588 | 0.0059316 | 0.0511478 |
| FQU82_RS15125 | FQU82_RS15125 | amino acid permease | -0.202714649 | 0.005954 | 0.0511478 |
| FQU82_RS06580 | csrA | carbon storage regulator CsrA | 0.298821513 | 0.0060505 | 0.0515656 |
| FQU82_RS01685 | pbpG | D-alanyl-D-alanine endopeptidase PBP7/8 | 0.194566432 | 0.0060665 | 0.0515656 |
| FQU82_RS05475 | FQU82_RS05475 | META and DUF4377 domain-containing protein | -0.25504157 | 0.0061886 | 0.0521432 |
| FQU82_RS09585 | zapE | cell division protein ZapE | -0.284901793 | 0.0061991 | 0.0521432 |
| FQU82_RS14215 | efp | elongation factor P | -0.17490413 | 0.0062857 | 0.0525983 |
| FQU82_RS16865 | FQU82_RS16865 | DUF2789 family protein | 0.628416182 | 0.006329 | 1 |
| FQU82_RS16195 | FQU82_RS16195 | thiolase family protein | -0.198379411 | 0.0064016 | 0.0532919 |
| FQU82_RS03880 | FQU82_RS03880 | ribonucleotide-diphosphate reductase subunit beta | -0.166445591 | 0.0064917 | 0.0534679 |
| FQU82_RS05325 | lysS | lysine--tRNA ligase | -0.223089081 | 0.0065003 | 0.0534679 |
| FQU82_RS11305 | kdsA | 3-deoxy-8-phosphooctulonate synthase | -0.205326112 | 0.0065221 | 0.0534679 |
| FQU82_RS10965 | FQU82_RS10965 | MBL fold metallo-hydrolase | 0.440269806 | 0.0068076 | 0.0555265 |
| FQU82_RS16975 | FQU82_RS16975 | tRNA-Arg | 0.610053878 | 0.0071322 | 1 |
| FQU82_RS18495 | FQU82_RS18495 | TonB-dependent siderophore receptor | -0.442702504 | 0.0071801 | 0.0582707 |
| FQU82_RS07050 | FQU82_RS07050 | beta-ketoacyl-ACP synthase III | -0.197954458 | 0.0072675 | 0.0586851 |
| FQU82_RS19055 | FQU82_RS19055 | hypothetical protein | 0.408386364 | 0.0073492 | 0.0590497 |
| FQU82_RS14510 | FQU82_RS14510 | NADP-dependent isocitrate dehydrogenase | -0.167441736 | 0.0074643 | 0.0596775 |
| FQU82_RS15560 | FQU82_RS15560 | hypothetical protein | 0.266369077 | 0.0075933 | 0.0604097 |
| FQU82_RS16160 | FQU82_RS16160 | uracil-xanthine permease family protein | -0.312384663 | 0.0079866 | 0.063125 |
| FQU82_RS13255 | FQU82_RS13255 | YeaC family protein | 0.676740003 | 0.0079867 | 1 |
| Novel00286 | - | PF02866:lactate/malate dehydrogenase, alpha/beta C-terminal domain|PF00056:lactate/malate dehydrogenase, NAD binding domain | -0.721723031 | 0.0080113 | 1 |
| FQU82_RS00300 | FQU82_RS00300 | 5-(carboxyamino)imidazole ribonucleotide synthase | -0.258883929 | 0.0080128 | 0.063125 |
| FQU82_RS16135 | mutM | bifunctional DNA-formamidopyrimidine glycosylase/DNA-(apurinic or apyrimidinic site) lyase | 0.36317452 | 0.0080971 | 0.06328 |
| FQU82_RS02020 | lpxO | lipid A hydroxylase LpxO | -0.24412464 | 0.0081108 | 0.06328 |
| FQU82_RS03440 | trxA | thioredoxin | 0.244153302 | 0.0081918 | 0.0636043 |
| FQU82_RS08925 | FQU82_RS08925 | dicarboxylate/amino acid:cation symporter | -0.544683977 | 0.0083018 | 0.0641499 |
| FQU82_RS00325 | dnaK | molecular chaperone DnaK | 0.144615018 | 0.0084334 | 0.0648572 |
| FQU82_RS01955 | FQU82_RS01955 | Na+/H+ antiporter NhaC family protein | -0.290160898 | 0.0088247 | 0.0675444 |
| FQU82_RS14310 | purB | adenylosuccinate lyase | -0.186353373 | 0.0088807 | 0.0676525 |
| FQU82_RS15540 | FQU82_RS15540 | hypothetical protein | 0.699943456 | 0.0091552 | 1 |
| FQU82_RS04555 | minD | septum site-determining protein MinD | -0.168833053 | 0.0091992 | 0.0690939 |
| Novel00072 | - | PF05681:Fumarate hydratase (Fumerase)|PF05683:Fumarase C-terminus | -0.541640135 | 0.0092029 | 0.0690939 |
| FQU82_RS04715 | FQU82_RS04715 | tyrosine-type recombinase/integrase | 0.336117922 | 0.0092052 | 0.0690939 |
| FQU82_RS03565 | ibaG | BolA family iron metabolism protein IbaG | 0.453423409 | 0.009241 | 0.0690939 |
| FQU82_RS00225 | FQU82_RS00225 | metal/formaldehyde-sensitive transcriptional repressor | 1.049248709 | 0.0093697 | 1 |
| FQU82_RS13145 | FQU82_RS13145 | NAD(P)H-dependent glycerol-3-phosphate dehydrogenase | -0.339089792 | 0.0098618 | 0.0732158 |
| FQU82_RS05470 | gdhA | NADP-specific glutamate dehydrogenase | -0.288351143 | 0.009883 | 0.0732158 |
| FQU82_RS15360 | FQU82_RS15360 | DUF5713 family protein | 1.514734215 | 0.0099317 | 1 |
| FQU82_RS11945 | FQU82_RS11945 | hypothetical protein | 0.535638251 | 0.0099733 | 0.0735474 |
| FQU82_RS12950 | FQU82_RS12950 | GntR family transcriptional regulator | 1.381266675 | 0.0101382 | 1 |
| FQU82_RS01695 | FQU82_RS01695 | homoserine dehydrogenase | -0.199816485 | 0.0104658 | 0.0768282 |
| FQU82_RS17225 | plsB | glycerol-3-phosphate 1-O-acyltransferase PlsB | -0.165638018 | 0.0105601 | 0.0771699 |
| FQU82_RS04310 | FQU82_RS04310 | ribose-phosphate pyrophosphokinase | -0.147762066 | 0.0107155 | 0.0779528 |
| FQU82_RS17340 | FQU82_RS17340 | PspC domain-containing protein | 0.708627177 | 0.0107813 | 0.0780799 |
| FQU82_RS09755 | FQU82_RS09755 | Rrf2 family transcriptional regulator | 0.241933644 | 0.0108533 | 0.0782503 |
| FQU82_RS11335 | FQU82_RS11335 | nuclear transport factor 2 family protein | 0.859669388 | 0.0109863 | 1 |
| FQU82_RS02280 | pnp | polyribonucleotide nucleotidyltransferase | -0.156690253 | 0.0112481 | 0.0807362 |
| FQU82_RS15475 | lpdA | dihydrolipoyl dehydrogenase | -0.148605859 | 0.0113227 | 0.0809124 |
| FQU82_RS03595 | FQU82_RS03595 | hypothetical protein | 1.083147743 | 0.0113394 | 1 |
| FQU82_RS03820 | FQU82_RS03820 | methionine synthase | -0.172879892 | 0.0116309 | 0.0824221 |
| FQU82_RS11565 | pfkB | 1-phosphofructokinase | -0.3714709 | 0.0116361 | 0.0824221 |
| FQU82_RS09280 | metK | methionine adenosyltransferase | -0.189775555 | 0.0117487 | 0.0828563 |
| FQU82_RS03950 | nuoH | NADH-quinone oxidoreductase subunit NuoH | -0.163524824 | 0.0120102 | 0.0837024 |
| FQU82_RS11535 | brnQ | branched-chain amino acid transport system II carrier protein | -0.219014421 | 0.0120276 | 0.0837024 |
| FQU82_RS16405 | FQU82_RS16405 | acyl-CoA dehydrogenase C-terminal domain-containing protein | -0.142695167 | 0.0120491 | 0.0837024 |
| FQU82_RS15395 | carA | glutamine-hydrolyzing carbamoyl-phosphate synthase small subunit | -0.185821121 | 0.0120759 | 0.0837024 |
| FQU82_RS09910 | FQU82_RS09910 | 3-deoxy-7-phosphoheptulonate synthase | -0.202995789 | 0.0122111 | 0.0842776 |
| FQU82_RS04550 | minE | cell division topological specificity factor MinE | 0.260454953 | 0.0126082 | 0.0866393 |
| FQU82_RS16290 | FQU82_RS16290 | peroxiredoxin | 0.191829267 | 0.0127014 | 0.0866393 |
| FQU82_RS02355 | FQU82_RS02355 | electron transfer flavoprotein-ubiquinone oxidoreductase | -0.146766826 | 0.0127143 | 0.0866393 |
| FQU82_RS13225 | FQU82_RS13225 | bifunctional prephenate dehydrogenase/3-phosphoshikimate 1-carboxyvinyltransferase | -0.182096642 | 0.0127944 | 0.0868188 |
| FQU82_RS01885 | tuf | elongation factor Tu | -0.842479489 | 0.0130911 | 1 |
| FQU82_RS02785 | lgt | prolipoprotein diacylglyceryl transferase | -0.276431889 | 0.0134665 | 0.0909977 |
| FQU82_RS13915 | FQU82_RS13915 | multifunctional CCA addition/repair protein | 0.276516437 | 0.013573 | 0.0910956 |
| FQU82_RS18740 | ribE | 6,7-dimethyl-8-ribityllumazine synthase | -0.179267847 | 0.0135938 | 0.0910956 |
| FQU82_RS08455 | FQU82_RS08455 | AMP-binding protein | -0.350054456 | 0.0139655 | 0.0931996 |
| FQU82_RS04235 | FQU82_RS04235 | elongation factor P hydroxylase | 0.998291586 | 0.0140227 | 1 |
| FQU82_RS00795 | FQU82_RS00795 | FKBP-type peptidyl-prolyl cis-trans isomerase | -0.212686016 | 0.0141299 | 0.0937022 |
| FQU82_RS11220 | FQU82_RS11220 | glucose/quinate/shikimate family membrane-bound PQQ-dependent dehydrogenase | -0.401881652 | 0.0141569 | 0.0937022 |
| FQU82_RS17445 | FQU82_RS17445 | amino acid permease | -0.245624824 | 0.0144315 | 0.0951304 |
| FQU82_RS02715 | rpmG | 50S ribosomal protein L33 | 0.477828574 | 0.0145779 | 0.0957045 |
| FQU82_RS13260 | FQU82_RS13260 | NAD(+) kinase | 0.240363528 | 0.0149339 | 0.0974378 |
| FQU82_RS03125 | leuS | leucine--tRNA ligase | -0.169280695 | 0.0149733 | 0.0974378 |
| FQU82_RS03150 | FQU82_RS03150 | GGDEF domain-containing phosphodiesterase | 0.44883435 | 0.0150229 | 0.0974378 |
| FQU82_RS15500 | FQU82_RS15500 | succinate dehydrogenase iron-sulfur subunit | -0.14372978 | 0.0151022 | 0.09756 |
| FQU82_RS05575 | FQU82_RS05575 | hypothetical protein | 0.478619129 | 0.0153123 | 0.0981716 |
| FQU82_RS00935 | prpC | 2-methylcitrate synthase | -0.308499287 | 0.0153184 | 0.0981716 |
| FQU82_RS14655 | FQU82_RS14655 | pyridoxal phosphate-dependent aminotransferase | -0.177430039 | 0.0154873 | 0.0985295 |
| FQU82_RS17835 | FQU82_RS17835 | hypothetical protein | 0.463715055 | 0.0155368 | 0.0985295 |
| FQU82_RS07650 | FQU82_RS07650 | lauroyl acyltransferase | 0.390868782 | 0.0155863 | 0.0985295 |
| FQU82_RS08955 | dapC | succinyldiaminopimelate transaminase | -0.271800651 | 0.0156183 | 0.0985295 |
| FQU82_RS12705 | purD | phosphoribosylamine--glycine ligase | -0.185104643 | 0.015775 | 0.0987168 |
| FQU82_RS11515 | grxD | Grx4 family monothiol glutaredoxin | 0.265549163 | 0.0157766 | 0.0987168 |
| FQU82_RS00195 | dnaA | chromosomal replication initiator protein DnaA | 0.250820064 | 0.0158942 | 0.0987168 |
| FQU82_RS15830 | FQU82_RS15830 | outer membrane protein transport protein | 0.163345391 | 0.0159154 | 0.0987168 |
| FQU82_RS17695 | FQU82_RS17695 | glutamate synthase subunit beta | -0.175873332 | 0.0159826 | 0.0987168 |
| FQU82_RS07155 | FQU82_RS07155 | LexA family transcriptional regulator | 0.413621485 | 0.0160147 | 0.0987168 |
| Novel00245 | - | PF02769:AIR synthase related protein, C-terminal domain|PF13507:CobB/CobQ-like glutamine amidotransferase domain | -0.612541076 | 0.0165428 | 0.1015843 |
| FQU82_RS02625 | gltP | glutamate/aspartate:proton symporter GltP | -0.163749938 | 0.0167386 | 0.1023972 |
| FQU82_RS01060 | alr | alanine racemase | 0.301385365 | 0.0169509 | 0.1033048 |
| FQU82_RS01910 | rplA | 50S ribosomal protein L1 | -0.132720575 | 0.0171694 | 0.104243 |
| FQU82_RS05675 | FQU82_RS05675 | hypothetical protein | 1.064309865 | 0.0172374 | 1 |
| FQU82_RS15130 | radA | DNA repair protein RadA | 0.264303095 | 0.0175264 | 0.1060119 |
| FQU82_RS11725 | FQU82_RS11725 | phosphatidate cytidylyltransferase | -0.243059839 | 0.0175931 | 0.1060183 |
| FQU82_RS05990 | FQU82_RS05990 | LysM peptidoglycan-binding domain-containing protein | -0.154275088 | 0.0178508 | 0.1071709 |
| Novel00203 | - | PF01808:AICARFT/IMPCHase bienzyme|PF02843:Phosphoribosylglycinamide synthetase, C domain|PF01071:Phosphoribosylglycinamide synthetase, ATP-grasp (A) domain|PF02844:Phosphoribosylglycinamide synthetase, N domain | -0.557253729 | 0.0178972 | 1 |
| FQU82_RS09275 | FQU82_RS09275 | hypothetical protein | 0.336527102 | 0.0179455 | 0.1071728 |
| FQU82_RS07605 | FQU82_RS07605 | universal stress protein | 0.202384065 | 0.0179838 | 0.1071728 |
| FQU82_RS01330 | atpA | F0F1 ATP synthase subunit alpha | -0.142926533 | 0.0181479 | 0.1077532 |
| FQU82_RS03590 | FQU82_RS03590 | anthranilate synthase component I family protein | -2.484764223 | 0.0185715 | 1 |
| FQU82_RS18895 | FQU82_RS18895 | phosphoribosylaminoimidazolesuccinocarboxamide synthase | -0.154486395 | 0.0186182 | 0.1101404 |
| FQU82_RS14430 | hemL | glutamate-1-semialdehyde 2,1-aminomutase | -0.185136718 | 0.0188982 | 0.1106632 |
| FQU82_RS16510 | glnE | bifunctional [glutamate--ammonia ligase]-adenylyl-L-tyrosine phosphorylase/[glutamate--ammonia-ligase] adenylyltransferase | 0.208606544 | 0.0189092 | 0.1106632 |
| FQU82_RS05585 | FQU82_RS05585 | hypothetical protein | 0.210554564 | 0.0189121 | 0.1106632 |
| FQU82_RS15235 | ttcA | tRNA 2-thiocytidine(32) synthetase TtcA | -0.285711595 | 0.0191484 | 0.1113952 |
| FQU82_RS13295 | FQU82_RS13295 | NirD/YgiW/YdeI family stress tolerance protein | 0.423148396 | 0.0191751 | 0.1113952 |
| Novel00070 | - | PF00574:Clp protease|PF00254:FKBP-type peptidyl-prolyl cis-trans isomerase|PF05698:Bacterial trigger factor protein (TF) C-terminus | -0.643381725 | 0.019687 | 1 |
| Novel00009 | - | PF00012:Hsp70 protein|PF01025:GrpE | -0.465067612 | 0.0196881 | 0.1138953 |
| Novel00064 | - | PF00171:Aldehyde dehydrogenase family|PF00830:Ribosomal L28 family | -0.517462501 | 0.0197465 | 0.1138953 |
| FQU82_RS12335 | hfq | RNA chaperone Hfq | 0.473161021 | 0.020007 | 0.1147217 |
| FQU82_RS04945 | FQU82_RS04945 | basic amino acid/polyamine antiporter | -0.452270081 | 0.0200319 | 0.1147217 |
| Novel00227 | - | PF00206:Lyase|PF08328:Adenylosuccinate lyase C-terminal|PF09997:Predicted membrane protein (DUF2238) | -0.688056993 | 0.0203684 | 1 |
| FQU82_RS10640 | FQU82_RS10640 | DHA2 family efflux MFS transporter permease subunit | -1.13476082 | 0.0205416 | 1 |
| FQU82_RS15090 | FQU82_RS15090 | tyrosine recombinase XerC | 0.362865821 | 0.0206087 | 0.1172281 |
| FQU82_RS10055 | prmB | 50S ribosomal protein L3 N(5)-glutamine methyltransferase | -0.255562312 | 0.0206147 | 0.1172281 |
| Novel00075 | - | PF04055:Radical SAM superfamily|PF21016:Ribosomal RNA large subunit methyltransferase N-terminal domain | -1.01272867 | 0.0206752 | 1 |
| FQU82_RS00200 | rpmH | 50S ribosomal protein L34 | 0.328553167 | 0.0206896 | 0.1172411 |
| FQU82_RS03095 | FQU82_RS03095 | enoyl-ACP reductase | -0.271144723 | 0.0207807 | 0.1173457 |
| FQU82_RS02010 | fadA | acetyl-CoA C-acyltransferase FadA | -0.15627137 | 0.0209667 | 0.1176481 |
| FQU82_RS08900 | FQU82_RS08900 | hypothetical protein | -0.203360279 | 0.02098 | 0.1176481 |
| FQU82_RS12865 | FQU82_RS12865 | paraquat-inducible protein A | 0.720671193 | 0.0210591 | 1 |
| FQU82_RS02940 | pta | phosphate acetyltransferase | -0.177317922 | 0.0210835 | 0.1178193 |
| FQU82_RS07735 | FQU82_RS07735 | urea amidolyase family protein | -0.367546419 | 0.0215099 | 0.1197879 |
| FQU82_RS09390 | FQU82_RS09390 | cupin domain-containing protein | -0.267294375 | 0.0218434 | 0.1212271 |
| FQU82_RS04455 | FQU82_RS04455 | NAD+ synthase | -0.167686168 | 0.0222922 | 0.1230997 |
| FQU82_RS15400 | carB | carbamoyl-phosphate synthase large subunit | -0.131925459 | 0.0223386 | 0.1230997 |
| FQU82_RS04960 | mqo | malate dehydrogenase (quinone) | -0.147706697 | 0.0224717 | 0.1230997 |
| FQU82_RS03555 | FQU82_RS03555 | RNA polymerase factor sigma-54 | 0.278424468 | 0.0224857 | 0.1230997 |
| FQU82_RS18810 | FQU82_RS18810 | HutD family protein | 0.608403089 | 0.0229574 | 1 |
| FQU82_RS05370 | FQU82_RS05370 | LysR family transcriptional regulator | 0.263350774 | 0.0230844 | 0.1259506 |
| FQU82_RS17000 | FQU82_RS17000 | hypothetical protein | 0.873512765 | 0.0232667 | 1 |
| FQU82_RS13860 | FQU82_RS13860 | MFS transporter | 0.394092416 | 0.0233567 | 0.1270068 |
| FQU82_RS06555 | FQU82_RS06555 | NCS2 family permease | -0.188870418 | 0.0237127 | 0.1283165 |
| FQU82_RS12140 | FQU82_RS12140 | TonB-dependent siderophore receptor | -0.596515157 | 0.0237556 | 1 |
| FQU82_RS16845 | pssA | CDP-diacylglycerol--serine O-phosphatidyltransferase | -0.236673681 | 0.0237564 | 0.1283165 |
| FQU82_RS01335 | atpG | F0F1 ATP synthase subunit gamma | -0.136559067 | 0.0238714 | 0.1285077 |
| FQU82_RS05915 | FQU82_RS05915 | FAD-dependent monooxygenase | -0.349953338 | 0.0239944 | 0.1287409 |
| FQU82_RS18760 | glmU | bifunctional UDP-N-acetylglucosamine diphosphorylase/glucosamine-1-phosphate N-acetyltransferase GlmU | -0.181608434 | 0.024286 | 0.1298738 |
| FQU82_RS09775 | FQU82_RS09775 | SurA N-terminal domain-containing protein | -0.194639519 | 0.0248032 | 0.1312823 |
| FQU82_RS08440 | FQU82_RS08440 | carboxyl transferase domain-containing protein | -0.204658669 | 0.0248447 | 0.1312823 |
| FQU82_RS02565 | FQU82_RS02565 | hypothetical protein | 0.230952063 | 0.0248559 | 0.1312823 |
| FQU82_RS09425 | FQU82_RS09425 | LPS-assembly protein LptD | -0.19315418 | 0.0249163 | 0.1312823 |
| FQU82_RS18720 | FQU82_RS18720 | hypothetical protein | 0.208436437 | 0.024989 | 0.1312823 |
| FQU82_RS17110 | rplE | 50S ribosomal protein L5 | -0.124148353 | 0.0250371 | 0.1312823 |
| FQU82_RS12365 | FQU82_RS12365 | GNAT family N-acetyltransferase | -1.033978434 | 0.0253479 | 1 |
| FQU82_RS12650 | FQU82_RS12650 | proteasome-type protease | 0.35887006 | 0.0255079 | 0.1332836 |
| FQU82_RS14980 | purN | phosphoribosylglycinamide formyltransferase | -0.217891955 | 0.0255838 | 0.1332836 |
| FQU82_RS01790 | argH | argininosuccinate lyase | -0.190084252 | 0.0257853 | 0.1339013 |
| FQU82_RS10060 | aroC | chorismate synthase | -0.216545191 | 0.0259221 | 0.1341802 |
| FQU82_RS10010 | FQU82_RS10010 | hypothetical protein | 3.143378022 | 0.0261843 | 1 |
| FQU82_RS02660 | FQU82_RS02660 | winged helix-turn-helix transcriptional regulator | 0.594072603 | 0.0264596 | 1 |
| Novel00036 | - | PF00291:Pyridoxal-phosphate dependent enzyme | -0.882803653 | 0.0265559 | 1 |
| FQU82_RS18860 | hppD | 4-hydroxyphenylpyruvate dioxygenase | 0.165831968 | 0.0270211 | 0.1394219 |
| FQU82_RS11315 | FQU82_RS11315 | hypothetical protein | -0.179277028 | 0.0272514 | 0.1401626 |
| FQU82_RS05345 | cysN | sulfate adenylyltransferase subunit CysN | -0.141540181 | 0.0279599 | 0.1432549 |
| FQU82_RS17905 | ompR | two-component system response regulator OmpR | 0.134982277 | 0.0280301 | 0.1432549 |
| FQU82_RS15120 | FQU82_RS15120 | lipoprotein | 0.939273575 | 0.0282884 | 1 |
| FQU82_RS08920 | FQU82_RS08920 | type II asparaginase | -0.183302103 | 0.0285641 | 0.1455235 |
| FQU82_RS15680 | FQU82_RS15680 | valine--tRNA ligase | -0.149466147 | 0.0292425 | 0.1485112 |
| FQU82_RS17425 | astD | succinylglutamate-semialdehyde dehydrogenase | -0.166237712 | 0.0296908 | 0.1502682 |
| FQU82_RS18885 | dapA | 4-hydroxy-tetrahydrodipicolinate synthase | 0.136813404 | 0.0297745 | 0.1502682 |
| FQU82_RS15115 | lysA | diaminopimelate decarboxylase | -0.192196226 | 0.0300645 | 0.1508882 |
| FQU82_RS03090 | FQU82_RS03090 | Bax inhibitor-1/YccA family protein | 0.189329617 | 0.0303557 | 0.1508882 |
| FQU82_RS09285 | tkt | transketolase | -0.152924186 | 0.0303935 | 0.1508882 |
| FQU82_RS04305 | rplY | 50S ribosomal protein L25 | 0.136711361 | 0.0304858 | 0.1508882 |
| FQU82_RS17685 | FQU82_RS17685 | LemA family protein | 0.164777164 | 0.0304945 | 0.1508882 |
| FQU82_RS01340 | atpD | F0F1 ATP synthase subunit beta | -0.128370713 | 0.030506 | 0.1508882 |
| FQU82_RS01730 | ftsW | putative lipid II flippase FtsW | -0.184888349 | 0.0305514 | 0.1508882 |
| FQU82_RS04060 | metG | methionine--tRNA ligase | -0.153465982 | 0.0307754 | 0.1515311 |
| FQU82_RS16735 | pdxA | 4-hydroxythreonine-4-phosphate dehydrogenase PdxA | 0.212187475 | 0.0310073 | 0.152209 |
| FQU82_RS10380 | FQU82_RS10380 | SDR family oxidoreductase | -0.968721801 | 0.031074 | 1 |
| Novel00089 | - | PF03129:Anticodon binding domain|PF00587:tRNA synthetase class II core domain (G, H, P, S and T)|PF00707:Translation initiation factor IF-3, C-terminal domain|PF05198:Translation initiation factor IF-3, N-terminal domain|PF02798:Glutathione S-transferase, N-terminal domain | -0.926845594 | 0.0314132 | 0.1537343 |
| FQU82_RS01185 | FQU82_RS01185 | BolA family protein | 0.379309775 | 0.0321529 | 0.1568791 |
| FQU82_RS11765 | adeN | multidrug efflux transcriptional repressor AdeN | 0.553505553 | 0.0326512 | 1 |
| FQU82_RS14580 | FQU82_RS14580 | nitronate monooxygenase | 0.212830842 | 0.0329203 | 0.1598438 |
| Novel00071 | - | PF11008:Protein of unknown function (DUF2846)|PF00574:Clp protease|PF07724:AAA domain (Cdc48 subfamily)|PF10431:C-terminal, D2-small domain, of ClpB protein|PF06689:ClpX C4-type zinc finger | -0.478915994 | 0.0329585 | 0.1598438 |
| FQU82_RS03945 | nuoG | NADH-quinone oxidoreductase subunit NuoG | -0.139213438 | 0.0333925 | 0.1614639 |
| FQU82_RS00645 | FQU82_RS00645 | IS91-like element ISVsa3 family transposase | 0.320040967 | 0.0336435 | 0.1621919 |
| FQU82_RS17045 | rpoA | DNA-directed RNA polymerase subunit alpha | -0.131393297 | 0.0340922 | 0.1638656 |
| FQU82_RS02445 | hemW | radical SAM family heme chaperone HemW | 0.30672313 | 0.03443 | 0.1649986 |
| FQU82_RS18035 | FQU82_RS18035 | potassium transporter Kup | -0.219154578 | 0.0346931 | 0.1657671 |
| FQU82_RS05270 | FQU82_RS05270 | rhomboid family intramembrane serine protease | 1.143631361 | 0.0354356 | 1 |
| FQU82_RS14830 | FQU82_RS14830 | hypothetical protein | 0.15425412 | 0.0356272 | 0.1695122 |
| FQU82_RS09400 | FQU82_RS09400 | DUF2237 domain-containing protein | 0.463524527 | 0.0357575 | 0.1695122 |
| FQU82_RS04220 | scpB | SMC-Scp complex subunit ScpB | 0.395365087 | 0.0358343 | 0.1695122 |
| FQU82_RS13170 | FQU82_RS13170 | sulfurtransferase TusA family protein | 0.71771438 | 0.0358406 | 1 |
| FQU82_RS01065 | FQU82_RS01065 | RidA family protein | 0.336219015 | 0.0358967 | 0.1695122 |
| FQU82_RS01710 | FQU82_RS01710 | FeoA family protein | 0.467877558 | 0.0361052 | 1 |
| FQU82_RS08840 | FQU82_RS08840 | LysR family transcriptional regulator | 0.881846421 | 0.0362742 | 1 |
| FQU82_RS10385 | FQU82_RS10385 | isochorismatase family protein | -0.775868049 | 0.0363542 | 1 |
| FQU82_RS17365 | glyQ | glycine--tRNA ligase subunit alpha | -0.166568145 | 0.0366306 | 0.1724734 |
| FQU82_RS02115 | coaE | dephospho-CoA kinase | 0.602156871 | 0.036951 | 1 |
| FQU82_RS07000 | trmB | tRNA (guanosine(46)-N7)-methyltransferase TrmB | -0.314940364 | 0.0369746 | 0.1735871 |
| FQU82_RS17895 | FQU82_RS17895 | SulP family inorganic anion transporter | -0.668325408 | 0.0373443 | 1 |
| FQU82_RS02645 | FQU82_RS02645 | ATP-binding cassette domain-containing protein | -0.196352941 | 0.0375119 | 0.175515 |
| FQU82_RS06050 | FQU82_RS06050 | NADPH-dependent 2,4-dienoyl-CoA reductase | -0.267564369 | 0.0376026 | 0.175515 |
| FQU82_RS18665 | FQU82_RS18665 | TetR/AcrR family transcriptional regulator | 0.454273201 | 0.037853 | 1 |
| FQU82_RS11745 | rimO | 30S ribosomal protein S12 methylthiotransferase RimO | -0.190746613 | 0.0379207 | 0.1764853 |
| FQU82_RS14805 | FQU82_RS14805 | CysB family HTH-type transcriptional regulator | -0.234582085 | 0.038029 | 0.1764853 |
| FQU82_RS11970 | FQU82_RS11970 | FMN-binding glutamate synthase family protein | -0.288669487 | 0.0381909 | 0.1767172 |
| Novel00224 | - | PF08207:Elongation factor P (EF-P) KOW-like domain|PF09285:Elongation factor P, C-terminal|PF01132:Elongation factor P (EF-P) OB domain | -0.955483321 | 0.0382224 | 1 |
| FQU82_RS04495 | fusA | elongation factor G | -0.116418337 | 0.0382978 | 0.1767172 |
| FQU82_RS02575 | FQU82_RS02575 | hypothetical protein | 1.119224389 | 0.0386662 | 1 |
| FQU82_RS09925 | FQU82_RS09925 | PepSY-associated TM helix domain-containing protein | -0.615077213 | 0.0390323 | 1 |
| FQU82_RS06965 | FQU82_RS06965 | dihydroorotase | -0.189991038 | 0.0391151 | 0.1799742 |
| FQU82_RS15570 | FQU82_RS15570 | APC family permease | -0.172367532 | 0.0393685 | 0.1806253 |
| FQU82_RS09820 | FQU82_RS09820 | SidA/IucD/PvdA family monooxygenase | -0.434505439 | 0.0396251 | 1 |
| FQU82_RS11560 | ptsP | phosphoenolpyruvate--protein phosphotransferase | -0.214795948 | 0.039785 | 0.182019 |
| FQU82_RS02085 | FQU82_RS02085 | YqgE/AlgH family protein | 0.166905196 | 0.040192 | 0.183362 |
| FQU82_RS09590 | FQU82_RS09590 | malate synthase G | -0.128530331 | 0.0403429 | 0.183532 |
| FQU82_RS12715 | FQU82_RS12715 | methionine ABC transporter ATP-binding protein | -0.185197466 | 0.0405137 | 0.183791 |
| FQU82_RS09765 | FQU82_RS09765 | phasin family protein | 0.561756146 | 0.0406533 | 1 |
| FQU82_RS13900 | FQU82_RS13900 | 16S rRNA (uracil(1498)-N(3))-methyltransferase | -0.535548144 | 0.0409065 | 1 |
| FQU82_RS09830 | FQU82_RS09830 | IucA/IucC family protein | 0.346094036 | 0.0410235 | 0.18511 |
| FQU82_RS17140 | rpsC | 30S ribosomal protein S3 | -0.127533958 | 0.0410337 | 0.18511 |
| FQU82_RS01930 | rpoC | DNA-directed RNA polymerase subunit beta | -0.124747211 | 0.0420032 | 0.1889559 |
| FQU82_RS15150 | FQU82_RS15150 | YkvA family protein | 1.112213852 | 0.0420686 | 1 |
| FQU82_RS15375 | rlmE | 23S rRNA (uridine(2552)-2'-O)-methyltransferase RlmE | 0.157252224 | 0.0428311 | 0.192125 |
| FQU82_RS06880 | alaS | alanine--tRNA ligase | -0.137390787 | 0.0429456 | 0.192125 |
| FQU82_RS00270 | FQU82_RS00270 | TerC family protein | 0.606919413 | 0.043218 | 1 |
| FQU82_RS04055 | FQU82_RS04055 | NF038215 family lipoprotein | 1.677442346 | 0.0433616 | 1 |
| FQU82_RS08575 | FQU82_RS08575 | GNAT family N-acetyltransferase | 1.063120811 | 0.0435854 | 1 |
| FQU82_RS16390 | baeS | sensor histidine kinase efflux regulator BaeS | 0.272946264 | 0.0436055 | 0.1942168 |
| Novel00137 | - | PF02272:DHHA1 domain | -1.584815105 | 0.0436073 | 1 |
| FQU82_RS03335 | panC | pantoate--beta-alanine ligase | -0.186666103 | 0.043748 | 0.1942168 |
| FQU82_RS17945 | hisB | imidazoleglycerol-phosphate dehydratase HisB | -0.207300033 | 0.0438746 | 0.1942168 |
| FQU82_RS03940 | nuoF | NADH-quinone oxidoreductase subunit NuoF | -0.150297168 | 0.0438942 | 0.1942168 |
| FQU82_RS09995 | FQU82_RS09995 | YARHG domain-containing protein | 0.244533598 | 0.0440348 | 0.1943065 |
| FQU82_RS08200 | FQU82_RS08200 | GntR family transcriptional regulator | 0.472388047 | 0.0442556 | 1 |
| FQU82_RS01375 | FQU82_RS01375 | DMT family transporter | -0.455020437 | 0.0443764 | 1 |
| FQU82_RS16180 | FQU82_RS16180 | hypothetical protein | 1.14370912 | 0.0445787 | 1 |
| FQU82_RS12175 | FQU82_RS12175 | TonB-dependent receptor | -0.475984961 | 0.0448787 | 1 |
| FQU82_RS15180 | FQU82_RS15180 | nitroreductase family protein | -0.228122316 | 0.0448827 | 0.1972866 |
| FQU82_RS17400 | FQU82_RS17400 | S8 family serine peptidase | 0.536435834 | 0.0449543 | 1 |
| FQU82_RS17080 | rpmD | 50S ribosomal protein L30 | 0.112531141 | 0.0449545 | 0.1972866 |
| FQU82_RS03310 | mazG | nucleoside triphosphate pyrophosphohydrolase | 0.869412619 | 0.0450705 | 1 |
| Novel00230 | - | PF03466:LysR substrate binding domain|PF00126:Bacterial regulatory helix-turn-helix protein, lysR family|PF00375:Sodium:dicarboxylate symporter family | -0.748547229 | 0.0452461 | 1 |
| FQU82_RS14450 | FQU82_RS14450 | hypothetical protein | 0.290121964 | 0.0453371 | 0.1984265 |
| Novel00105 | - | PF01479:S4 domain|PF00849:RNA pseudouridylate synthase | -0.615989652 | 0.0454904 | 1 |
| FQU82_RS13195 | FQU82_RS13195 | metal-dependent hydrolase | 1.403544697 | 0.0455102 | 1 |
| FQU82_RS10225 | FQU82_RS10225 | hypothetical protein | 3.794230926 | 0.0455971 | 1 |
| FQU82_RS08320 | FQU82_RS08320 | CaiB/BaiF CoA-transferase family protein | -0.260175959 | 0.0457047 | 0.1994456 |
| FQU82_RS13080 | tsaD | tRNA (adenosine(37)-N6)-threonylcarbamoyltransferase complex transferase subunit TsaD | -0.277464092 | 0.0458169 | 0.1994456 |
| FQU82_RS05535 | FQU82_RS05535 | hypothetical protein | 1.131153065 | 0.0465942 | 1 |
| FQU82_RS15995 | FQU82_RS15995 | tRNA-Asp | 0.247554052 | 0.0466102 | 0.2012213 |
| FQU82_RS15470 | sucC | ADP-forming succinate--CoA ligase subunit beta | -0.134470307 | 0.046691 | 0.2012213 |
| FQU82_RS19060 | ilvD | dihydroxy-acid dehydratase | -0.133516432 | 0.0467597 | 0.2012213 |
| FQU82_RS03685 | FQU82_RS03685 | copper resistance protein B | 0.582956579 | 0.0468028 | 1 |
| FQU82_RS09520 | FQU82_RS09520 | 6-carboxytetrahydropterin synthase | 0.240763062 | 0.0469194 | 0.2012213 |
| FQU82_RS16545 | tgt | tRNA guanosine(34) transglycosylase Tgt | -0.184136503 | 0.0469855 | 0.2012213 |
| FQU82_RS09065 | FQU82_RS09065 | amino acid ABC transporter ATP-binding protein | -0.333233816 | 0.0470399 | 0.2012213 |
| Novel00319 | - | PF00575:S1 RNA binding domain | -1.385052012 | 0.0470803 | 1 |
| FQU82_RS05230 | nfuA | Fe-S biogenesis protein NfuA | 0.162455692 | 0.047097 | 0.2012213 |
| FQU82_RS03935 | nuoE | NADH-quinone oxidoreductase subunit NuoE | -0.147796595 | 0.0473442 | 0.2017436 |
| FQU82_RS01670 | FQU82_RS01670 | sigma-54 dependent transcriptional regulator | 0.864729918 | 0.047447 | 1 |
| FQU82_RS07150 | FQU82_RS07150 | transposase | 0.691243704 | 0.0476515 | 1 |
| FQU82_RS14740 | lepA | translation elongation factor 4 | -0.138843733 | 0.0477506 | 0.2020969 |
| FQU82_RS12340 | miaA | tRNA (adenosine(37)-N6)-dimethylallyltransferase MiaA | 0.352676889 | 0.047778 | 0.2020969 |
| FQU82_RS06960 | FQU82_RS06960 | aspartate carbamoyltransferase catalytic subunit | -0.227055922 | 0.0478025 | 0.2020969 |
| FQU82_RS05890 | ssrS | 6S RNA | 1.244965929 | 0.0479293 | 1 |
| FQU82_RS01240 | FQU82_RS01240 | DUF3861 domain-containing protein | 1.066917645 | 0.0480869 | 1 |
| Novel00269 | - | PF00679:Elongation factor G C-terminus|PF00009:Elongation factor Tu GTP binding domain|PF03144:Elongation factor Tu domain 2|PF21018:TypA/BipA C-terminal domain | -0.560817979 | 0.0483762 | 0.2029994 |
| FQU82_RS14455 | FQU82_RS14455 | DUF3144 domain-containing protein | -0.340420549 | 0.0483867 | 0.2029994 |
| FQU82_RS11455 | cydB | cytochrome d ubiquinol oxidase subunit II | -0.139828133 | 0.0483931 | 0.2029994 |
| FQU82_RS09005 | ychF | redox-regulated ATPase Ych | -0.195455668 | 0.049014 | 0.2050715 |
| FQU82_RS17710 | aroB | 3-dehydroquinate synthase | -0.200125747 | 0.0494501 | 0.205526 |
| FQU82_RS12370 | FQU82_RS12370 | pseudouridine synthase | 0.433046928 | 0.0495027 | 1 |
| FQU82_RS09375 | sstT | serine/threonine transporter SstT | -0.152653642 | 0.0495041 | 0.205526 |
| FQU82_RS03345 | FQU82_RS03345 | HPr family phosphocarrier protein | 0.304921242 | 0.0495044 | 0.205526 |
| FQU82_RS05965 | FQU82_RS05965 | BON domain-containing protein | -0.202382984 | 0.0498758 | 0.2065368 |

**Table S4 The DEGs identified in *A. baumannii* after combination treatment**

| **Gene_id** | **Gene name** | **Gene description** | **log2FC (Combined/Control)** | **pvalue** | **padj** |
| --- | --- | --- | --- | --- | --- |
| FQU82_RS18060 | FQU82_RS18060 | NAD(P)H-dependent oxidoreductase | 6.961929832 | 0 | 0 |
| FQU82_RS02000 | FQU82_RS02000 | alpha/beta hydrolase | 7.545309777 | 0 | 0 |
| FQU82_RS02655 | FQU82_RS02655 | zinc-binding alcohol dehydrogenase family protein | 5.005670461 | 5.04E-296 | 5.86E-293 |
| FQU82_RS00340 | FQU82_RS00340 | MBL fold metallo-hydrolase | 6.165393651 | 2.74E-287 | 2.39E-284 |
| FQU82_RS13375 | adeG | multidrug efflux RND transporter permease subunit AdeG | 5.415521361 | 1.04E-249 | 7.22E-247 |
| FQU82_RS13380 | adeH | multidrug efflux RND transporter outer membrane subunit AdeH | 5.430825556 | 1.39E-228 | 8.10E-226 |
| FQU82_RS10500 | FQU82_RS10500 | fimbrial protein | 3.884650725 | 6.97E-204 | 3.47E-201 |
| FQU82_RS18365 | FQU82_RS18365 | alkene reductase | 3.873869414 | 4.50E-198 | 1.96E-195 |
| FQU82_RS00445 | ssuD | FMNH2-dependent alkanesulfonate monooxygenase | 4.695966965 | 2.97E-185 | 1.15E-182 |
| FQU82_RS13370 | adeF | multidrug efflux RND transporter periplasmic adaptor subunit AdeF | 5.295466107 | 5.93E-164 | 2.07E-161 |
| FQU82_RS02905 | FQU82_RS02905 | TonB-dependent siderophore receptor | -3.606285334 | 1.52E-157 | 4.83E-155 |
| FQU82_RS14475 | FQU82_RS14475 | LysR family transcriptional regulator | 3.268785852 | 3.72E-145 | 1.08E-142 |
| FQU82_RS10160 | FQU82_RS10160 | NAD(P)H-dependent oxidoreductase | 3.692042813 | 4.01E-138 | 1.08E-135 |
| FQU82_RS18805 | hutU | urocanate hydratase | 3.11305921 | 8.39E-138 | 2.09E-135 |
| FQU82_RS17175 | rpsJ | 30S ribosomal protein S10 | -2.985651262 | 7.06E-131 | 1.64E-128 |
| FQU82_RS09405 | FQU82_RS09405 | phosphoglycerate kinase | -3.429027209 | 1.14E-129 | 2.48E-127 |
| FQU82_RS04310 | FQU82_RS04310 | ribose-phosphate pyrophosphokinase | -3.263480443 | 4.63E-129 | 9.49E-127 |
| FQU82_RS01365 | FQU82_RS01365 | MFS transporter | 3.885672056 | 2.53E-127 | 4.89E-125 |
| FQU82_RS10775 | FQU82_RS10775 | cupin domain-containing protein | 3.337597769 | 5.00E-123 | 9.17E-121 |
| FQU82_RS12555 | FQU82_RS12555 | LLM class flavin-dependent oxidoreductase | 3.438403133 | 1.18E-122 | 2.06E-120 |
| FQU82_RS18325 | sfnG | dimethyl sulfone monooxygenase SfnG | 4.1137856 | 1.14E-118 | 1.89E-116 |
| FQU82_RS01335 | atpG | F0F1 ATP synthase subunit gamma | -2.955677714 | 1.39E-117 | 2.20E-115 |
| FQU82_RS10765 | FQU82_RS10765 | NAD(P)-dependent alcohol dehydrogenase | 3.131243243 | 1.99E-117 | 3.02E-115 |
| FQU82_RS03015 | ndk | nucleoside-diphosphate kinase | -3.206541983 | 7.42E-117 | 1.08E-114 |
| FQU82_RS00450 | FQU82_RS00450 | sulfonate ABC transporter substrate-binding protein | 4.315906811 | 9.21E-117 | 1.28E-114 |
| FQU82_RS01320 | FQU82_RS01320 | F0F1 ATP synthase subunit B | -3.157081912 | 3.15E-116 | 4.23E-114 |
| FQU82_RS09850 | FQU82_RS09850 | TonB-dependent receptor | -3.337156516 | 1.82E-115 | 2.35E-113 |
| FQU82_RS10770 | FQU82_RS10770 | carboxymuconolactone decarboxylase family protein | 3.252941234 | 1.22E-113 | 1.52E-111 |
| FQU82_RS18320 | msuE | FMN reductase | 3.573977402 | 3.91E-112 | 4.70E-110 |
| FQU82_RS18840 | fahA | fumarylacetoacetase | 3.231726221 | 5.32E-111 | 6.18E-109 |
| FQU82_RS16155 | typA | translational GTPase TypA | -3.388625203 | 9.55E-108 | 1.07E-105 |
| FQU82_RS07990 | FQU82_RS07990 | dihydrodipicolinate synthase family protein | 4.111924878 | 1.18E-100 | 1.28E-98 |
| FQU82_RS01405 | FQU82_RS01405 | TonB-dependent copper receptor | -3.088410418 | 1.25E-100 | 1.32E-98 |
| FQU82_RS01315 | atpE | F0F1 ATP synthase subunit C | -3.090402751 | 2.73E-99 | 2.79E-97 |
| FQU82_RS12700 | purH | bifunctional phosphoribosylaminoimidazolecarboxamide formyltransferase/IMP cyclohydrolase | -3.047812977 | 8.90E-97 | 8.87E-95 |
| FQU82_RS00435 | FQU82_RS00435 | ATP-binding cassette domain-containing protein | 5.44531756 | 4.11E-94 | 3.98E-92 |
| FQU82_RS17050 | rpsD | 30S ribosomal protein S4 | -2.630857858 | 1.03E-91 | 9.70E-90 |
| FQU82_RS01960 | FQU82_RS01960 | OmpW family outer membrane protein | -5.773342831 | 1.52E-90 | 1.39E-88 |
| FQU82_RS09375 | sstT | serine/threonine transporter SstT | -2.931451593 | 5.44E-90 | 4.87E-88 |
| FQU82_RS17055 | rpsK | 30S ribosomal protein S11 | -2.685824552 | 8.45E-88 | 7.37E-86 |
| Novel00354 | - | PF01979:Amidohydrolase family|PF00491:Arginase family|PF00324:Amino acid permease|PF01175:Urocanase Rossmann-like domain|PF17392:Urocanase C-terminal domain|PF17391:Urocanase N-terminal domain|PF00221:Aromatic amino acid lyase | 3.191232492 | 1.50E-87 | 1.27E-85 |
| FQU82_RS09005 | ychF | redox-regulated ATPase YchF | -3.318364008 | 2.90E-87 | 2.41E-85 |
| FQU82_RS08415 | FQU82_RS08415 | indolepyruvate ferredoxin oxidoreductase family protein | 3.66160992 | 3.47E-87 | 2.81E-85 |
| FQU82_RS11535 | brnQ | branched-chain amino acid transport system II carrier protein | -3.092602299 | 5.86E-86 | 4.64E-84 |
| FQU82_RS17580 | rpsP | 30S ribosomal protein S16 | -2.950468558 | 1.33E-85 | 1.03E-83 |
| FQU82_RS10780 | FQU82_RS10780 | AraC family transcriptional regulator | 3.367574873 | 2.06E-84 | 1.56E-82 |
| FQU82_RS17115 | rplX | 50S ribosomal protein L24 | -2.634042488 | 4.24E-84 | 3.14E-82 |
| FQU82_RS17100 | rpsH | 30S ribosomal protein S8 | -2.71676723 | 2.57E-83 | 1.87E-81 |
| FQU82_RS18850 | FQU82_RS18850 | VOC family protein | 2.489724974 | 9.56E-81 | 6.80E-79 |
| FQU82_RS02430 | FQU82_RS02430 | acyl-CoA dehydrogenase family protein | 3.105400819 | 2.42E-80 | 1.69E-78 |
| FQU82_RS10965 | FQU82_RS10965 | MBL fold metallo-hydrolase | 2.894641336 | 2.72E-80 | 1.86E-78 |
| FQU82_RS17155 | rplB | 50S ribosomal protein L2 | -2.831352528 | 3.02E-80 | 2.03E-78 |
| FQU82_RS14800 | FQU82_RS14800 | sulfate ABC transporter ATP-binding protein | 2.871240813 | 1.36E-79 | 8.97E-78 |
| FQU82_RS11455 | cydB | cytochrome d ubiquinol oxidase subunit II | -2.425155494 | 2.78E-78 | 1.80E-76 |
| FQU82_RS05490 | lon | endopeptidase La | 3.418388094 | 4.29E-78 | 2.72E-76 |
| FQU82_RS14805 | FQU82_RS14805 | CysB family HTH-type transcriptional regulator | 2.462242522 | 2.19E-76 | 1.36E-74 |
| FQU82_RS19020 | FQU82_RS19020 | efflux RND transporter permease subunit | 2.296582788 | 2.24E-75 | 1.37E-73 |
| FQU82_RS17040 | rplQ | 50S ribosomal protein L17 | -2.333296355 | 6.74E-75 | 4.05E-73 |
| FQU82_RS18370 | FQU82_RS18370 | helix-turn-helix transcriptional regulator | 3.731055492 | 7.37E-75 | 4.35E-73 |
| FQU82_RS02425 | FQU82_RS02425 | SfnB family sulfur acquisition oxidoreductase | 2.812832415 | 2.77E-72 | 1.61E-70 |
| FQU82_RS15300 | FQU82_RS15300 | phosphoenolpyruvate carboxykinase (GTP) | -2.742194479 | 9.96E-72 | 5.69E-70 |
| FQU82_RS01325 | FQU82_RS01325 | F0F1 ATP synthase subunit delta | -3.188662725 | 1.54E-71 | 8.67E-70 |
| FQU82_RS01905 | rplK | 50S ribosomal protein L11 | -2.908033251 | 4.66E-71 | 2.58E-69 |
| FQU82_RS17815 | FQU82_RS17815 | amino acid permease | -2.606793258 | 5.54E-71 | 3.02E-69 |
| FQU82_RS06935 | clpB | ATP-dependent chaperone ClpB | 3.286955868 | 1.26E-70 | 6.78E-69 |
| FQU82_RS00440 | ssuC | aliphatic sulfonate ABC transporter permease SsuC | 4.645957779 | 3.14E-70 | 1.66E-68 |
| FQU82_RS18095 | FQU82_RS18095 | aldo/keto reductase | 3.036778491 | 4.93E-70 | 2.56E-68 |
| FQU82_RS19040 | FQU82_RS19040 | solute carrier family 23 protein | -2.693951269 | 6.63E-70 | 3.40E-68 |
| FQU82_RS11450 | FQU82_RS11450 | cytochrome ubiquinol oxidase subunit I | -2.506996512 | 9.43E-70 | 4.77E-68 |
| FQU82_RS00455 | FQU82_RS00455 | sulfonate ABC transporter substrate-binding protein | 2.971460165 | 1.34E-69 | 6.68E-68 |
| Novel00335 | - | PF00724:NADH:flavin oxidoreductase / NADH oxidase family | 4.625046513 | 2.41E-69 | 1.18E-67 |
| FQU82_RS18860 | hppD | 4-hydroxyphenylpyruvate dioxygenase | 2.644214642 | 5.16E-69 | 2.50E-67 |
| FQU82_RS14635 | uvrB | excinuclease ABC subunit UvrBn | 2.535167914 | 1.44E-68 | 6.90E-67 |
| FQU82_RS12630 | rpsR | 30S ribosomal protein S18 | -2.15732655 | 4.18E-68 | 1.97E-66 |
| Novel00129 | - | PF02190:ATP-dependent protease La (LON) substrate-binding domain|PF05362:Lon protease (S16) C-terminal proteolytic domain|PF00004:ATPase family associated with various cellular activities (AAA) | 4.014556747 | 9.15E-68 | 4.25E-66 |
| FQU82_RS09415 | fba | fructose-bisphosphate aldolase class II | -2.021191973 | 1.85E-67 | 8.50E-66 |
| Novel00328 | - | PF08240:Alcohol dehydrogenase GroES-like domain|PF00107:Zinc-binding dehydrogenase|PF00440:Bacterial regulatory proteins, tetR family|PF16925:Tetracyclin repressor-like, C-terminal domain|PF06993:Protein of unknown function (DUF1304) | 2.520536831 | 2.07E-67 | 9.38E-66 |
| FQU82_RS17080 | rpmD | 50S ribosomal protein L30 | -2.379355013 | 2.74E-67 | 1.23E-65 |
| FQU82_RS18835 | FQU82_RS18835 | amino acid permease | 2.382351835 | 1.01E-65 | 4.45E-64 |
| FQU82_RS01955 | FQU82_RS01955 | Na+/H+ antiporter NhaC family protein | -3.153294534 | 1.20E-65 | 5.23E-64 |
| Novel00169 | - | PF16970:Type-1 fimbrial protein, A | 4.763046513 | 1.61E-64 | 6.93E-63 |
| FQU82_RS15395 | carA | glutamine-hydrolyzing carbamoyl-phosphate synthase small subunit | -2.43718673 | 1.89E-64 | 8.02E-63 |
| FQU82_RS14815 | dapD | 2,3,4,5-tetrahydropyridine-2,6-dicarboxylate N-succinyltransferase | -2.037277999 | 9.50E-64 | 3.99E-62 |
| FQU82_RS08785 | FQU82_RS08785 | monooxygenase | 2.565175276 | 1.79E-63 | 7.42E-62 |
| FQU82_RS16170 | FQU82_RS16170 | amino acid permease | -2.457450743 | 3.10E-63 | 1.27E-61 |
| FQU82_RS13890 | ahcY | adenosylhomocysteinase | -2.44075787 | 4.27E-63 | 1.73E-61 |
| FQU82_RS18030 | FQU82_RS18030 | hypothetical protein | -2.643771565 | 2.26E-62 | 8.97E-61 |
| FQU82_RS18240 | FQU82_RS18240 | DUF475 domain-containing protein | -2.788105478 | 2.26E-62 | 8.97E-61 |
| FQU82_RS16740 | rplM | 50S ribosomal protein L13 | -2.01670096 | 8.36E-62 | 3.27E-60 |
| FQU82_RS17105 | rpsN | 30S ribosomal protein S14 | -2.520803181 | 4.35E-61 | 1.69E-59 |
| FQU82_RS17365 | glyQ | glycine--tRNA ligase subunit alpha | -2.20055442 | 1.54E-60 | 5.90E-59 |
| FQU82_RS02745 | FQU82_RS02745 | MotA/TolQ/ExbB proton channel family protein | -2.192529544 | 5.28E-60 | 2.00E-58 |
| FQU82_RS08040 | FQU82_RS08040 | APC family permease | 4.734252045 | 6.90E-60 | 2.59E-58 |
| FQU82_RS17090 | rplR | 50S ribosomal protein L18 | -2.487039687 | 1.12E-59 | 4.15E-58 |
| FQU82_RS14930 | FQU82_RS14930 | class 1 fructose-bisphosphatase | -2.810561894 | 1.26E-59 | 4.62E-58 |
| FQU82_RS11740 | pyrH | UMP kinase | -2.51988722 | 3.96E-59 | 1.44E-57 |
| FQU82_RS05495 | ata | trimeric autotransporter adhesin Ata | -3.478126001 | 4.55E-59 | 1.64E-57 |
| FQU82_RS01915 | rplJ | 50S ribosomal protein L10 | -2.855119253 | 7.26E-59 | 2.58E-57 |
| FQU82_RS06555 | FQU82_RS06555 | NCS2 family permease | -2.271851174 | 7.95E-58 | 2.80E-56 |
| FQU82_RS14785 | FQU82_RS14785 | alpha/beta hydrolase | 2.464931729 | 1.41E-57 | 4.93E-56 |
| FQU82_RS16745 | rpsI | 30S ribosomal protein S9 | -1.874023933 | 8.03E-57 | 2.77E-55 |
| FQU82_RS13365 | adeL | multidrug efflux transcriptional repressor AdeL | 2.524489186 | 2.55E-56 | 8.71E-55 |
| FQU82_RS17880 | ettA | energy-dependent translational throttle protein EttA | -2.242712549 | 3.09E-56 | 1.05E-54 |
| FQU82_RS14790 | cysT | sulfate ABC transporter permease subunit CysT | 2.282198857 | 6.02E-56 | 2.02E-54 |
| FQU82_RS12600 | cyoA | ubiquinol oxidase subunit II | -2.027014879 | 1.21E-55 | 4.01E-54 |
| FQU82_RS08035 | FQU82_RS08035 | aldehyde dehydrogenase (NADP(+)) | 4.583337622 | 6.17E-55 | 2.03E-53 |
| FQU82_RS01330 | atpA | F0F1 ATP synthase subunit alpha | -2.871084288 | 8.56E-55 | 2.79E-53 |
| FQU82_RS14175 | FQU82_RS14175 | pirin-like bicupin family protein | 2.760307449 | 1.06E-54 | 3.41E-53 |
| FQU82_RS01345 | FQU82_RS01345 | F0F1 ATP synthase subunit epsilon | -2.174515681 | 1.68E-54 | 5.38E-53 |
| FQU82_RS14620 | FQU82_RS14620 | glyceraldehyde-3-phosphate dehydrogenase | -2.436016048 | 7.01E-54 | 2.22E-52 |
| FQU82_RS18570 | FQU82_RS18570 | outer membrane protein OmpK | -3.709502657 | 8.66E-54 | 2.72E-52 |
| FQU82_RS04610 | FQU82_RS04610 | bacteriohemerythrin | -4.44369598 | 1.42E-53 | 4.41E-52 |
| FQU82_RS09040 | FQU82_RS09040 | SfnB family sulfur acquisition oxidoreductase | 2.532517067 | 1.69E-53 | 5.23E-52 |
| FQU82_RS14285 | dacC | D-alanyl-D-alanine carboxypeptidase PBP5/6 | -2.504631947 | 1.83E-53 | 5.61E-52 |
| FQU82_RS12625 | rpsF | 30S ribosomal protein S6 | -1.786601994 | 2.77E-53 | 8.39E-52 |
| FQU82_RS16160 | FQU82_RS16160 | uracil-xanthine permease family protein | -2.881214877 | 3.10E-53 | 9.31E-52 |
| FQU82_RS17120 | rplN | 50S ribosomal protein L14 | -2.400133317 | 3.35E-53 | 9.97E-52 |
| FQU82_RS02280 | pnp | polyribonucleotide nucleotidyltransferase | -2.197743903 | 6.80E-53 | 2.01E-51 |
| FQU82_RS06045 | argG | argininosuccinate synthase | -2.184197641 | 1.06E-52 | 3.10E-51 |
| FQU82_RS08820 | tauD | taurine dioxygenase | 2.065580749 | 1.51E-52 | 4.38E-51 |
| FQU82_RS14345 | FQU82_RS14345 | substrate-binding domain-containing protein | -3.052302458 | 1.52E-52 | 4.38E-51 |
| FQU82_RS01310 | atpB | F0F1 ATP synthase subunit A | -2.328859448 | 3.39E-52 | 9.68E-51 |
| FQU82_RS12705 | purD | phosphoribosylamine glycine ligase | -2.067855903 | 4.19E-52 | 1.18E-50 |
| FQU82_RS17060 | rpsM | 30S ribosomal protein S13 | -2.463083223 | 4.19E-52 | 1.18E-50 |
| FQU82_RS17150 | rpsS | 30S ribosomal protein S19 | -2.69818223 | 4.35E-52 | 1.21E-50 |
| FQU82_RS17085 | rpsE | 30S ribosomal protein S5 | -2.35051991 | 6.89E-52 | 1.91E-50 |
| FQU82_RS14310 | purB | adenylosuccinate lyase | -2.138670015 | 1.11E-51 | 3.04E-50 |
| FQU82_RS10520 | adeA | multidrug efflux RND transporter periplasmic adaptor subunit AdeA | -2.235451374 | 1.44E-51 | 3.92E-50 |
| FQU82_RS09280 | metK | methionine adenosyltransferase | -2.113817001 | 1.50E-51 | 4.05E-50 |
| FQU82_RS01225 | guaA | glutamine-hydrolyzing GMP synthase | -1.973583684 | 2.71E-51 | 7.27E-50 |
| FQU82_RS04305 | rplY | 50S ribosomal protein L25 | -2.081436728 | 3.85E-51 | 1.02E-49 |
| FQU82_RS17065 | rpmJ | 50S ribosomal protein L36 | -2.092034274 | 4.84E-51 | 1.28E-49 |
| FQU82_RS02625 | gltP | glutamate/aspartate:proton symporter GltP | -2.200835547 | 6.39E-51 | 1.67E-49 |
| FQU82_RS08790 | FQU82_RS08790 | LLM class flavin-dependent oxidoreductase | 3.438315044 | 1.73E-50 | 4.51E-49 |
| FQU82_RS18800 | hutH | histidine ammonia-lyase | 2.261929591 | 2.40E-50 | 6.19E-49 |
| FQU82_RS17130 | rpmC | 50S ribosomal protein L29 | -2.418568349 | 6.47E-50 | 1.66E-48 |
| FQU82_RS11425 | FQU82_RS11425 | TonB-dependent siderophore receptor | -3.050253935 | 9.10E-50 | 2.32E-48 |
| FQU82_RS17160 | rplW | 50S ribosomal protein L23 | -2.906039924 | 5.22E-49 | 1.32E-47 |
| FQU82_RS09035 | FQU82_RS09035 | SfnB family sulfur acquisition oxidoreductase | 2.952445874 | 9.10E-49 | 2.28E-47 |
| FQU82_RS17165 | rplD | 50S ribosomal protein L4 | -2.872145761 | 1.01E-48 | 2.51E-47 |
| FQU82_RS15615 | rplU | 50S ribosomal protein L21 | -1.94819999 | 1.24E-48 | 3.05E-47 |
| FQU82_RS00210 | yidD | membrane protein insertion efficiency factor YidD | -2.527883619 | 1.29E-48 | 3.17E-47 |
| FQU82_RS05475 | FQU82_RS05475 | META and DUF4377 domain-containing protein | -2.234990904 | 1.56E-48 | 3.81E-47 |
| FQU82_RS01370 | FQU82_RS01370 | TetR/AcrR family transcriptional regulator | 2.558362829 | 4.02E-48 | 9.68E-47 |
| FQU82_RS11300 | eno | phosphopyruvate hydratase | -2.242329505 | 4.03E-48 | 9.68E-47 |
| FQU82_RS18760 | glmU | bifunctional UDP-N-acetylglucosamine diphosphorylase/glucosamine-1-phosphate N-acetyltransferase GlmU | -2.040698726 | 4.39E-48 | 1.05E-46 |
| Novel00122 | - | PF13356:Arm DNA-binding domain | 2.003076018 | 7.35E-48 | 1.74E-46 |
| FQU82_RS09030 | FQU82_RS09030 | LLM class flavin-dependent oxidoreductase | 3.817049435 | 8.28E-48 | 1.95E-46 |
| FQU82_RS11305 | kdsA | 3-deoxy-8-phosphooctulonate synthase | -2.000398108 | 1.53E-47 | 3.58E-46 |
| FQU82_RS17145 | rplV | 50S ribosomal protein L22 | -2.653145222 | 1.68E-47 | 3.91E-46 |
| FQU82_RS09755 | FQU82_RS09755 | Rrf2 family transcriptional regulator | 1.967072174 | 3.95E-47 | 9.12E-46 |
| FQU82_RS17170 | rplC | 50S ribosomal protein L3 | -2.811412022 | 5.05E-47 | 1.16E-45 |
| FQU82_RS10355 | antA | anthranilate 1,2-dioxygenase large subunit | 8.190586639 | 5.33E-47 | 1.21E-45 |
| FQU82_RS10055 | prmB | 50S ribosomal protein L3 N(5)-glutamine methyltransferase | -2.66221207 | 1.14E-46 | 2.59E-45 |
| FQU82_RS04510 | FQU82_RS04510 | metal-dependent hydrolase | 2.446409612 | 1.24E-46 | 2.79E-45 |
| FQU82_RS18260 | FQU82_RS18260 | zinc-binding dehydrogenase | 1.853593262 | 1.45E-46 | 3.25E-45 |
| FQU82_RS06570 | FQU82_RS06570 | hypothetical protein | -2.529252252 | 2.88E-46 | 6.40E-45 |
| FQU82_RS13835 | tsf | translation elongation factor Ts | -2.623074355 | 4.88E-46 | 1.08E-44 |
| FQU82_RS18765 | glmS | glutamine--fructose-6-phosphate transaminase (isomerizing) | -1.914576583 | 6.25E-46 | 1.37E-44 |
| FQU82_RS14795 | cysW | sulfate ABC transporter permease subunit CysW | 2.525997498 | 1.48E-45 | 3.23E-44 |
| FQU82_RS17140 | rpsC | 30S ribosomal protein S3 | -2.672011611 | 2.50E-45 | 5.42E-44 |
| FQU82_RS09935 | FQU82_RS09935 | TonB-dependent siderophore receptor | -2.874763132 | 6.08E-45 | 1.31E-43 |
| FQU82_RS08815 | tauC | taurine ABC transporter permease TauC | 1.648447482 | 1.06E-44 | 2.26E-43 |
| FQU82_RS13330 | yegQ | tRNA 5-hydroxyuridine modification protein YegQ | -2.973792208 | 1.07E-44 | 2.28E-43 |
| FQU82_RS18265 | FQU82_RS18265 | TetR/AcrR family transcriptional regulator | 1.655108325 | 6.86E-44 | 1.45E-42 |
| FQU82_RS09745 | iscU | Fe-S cluster assembly scaffold IscU | 1.930116328 | 7.07E-44 | 1.48E-42 |
| FQU82_RS17110 | rplE | 50S ribosomal protein L5 | -2.510426465 | 7.16E-44 | 1.49E-42 |
| Novel00170 | - | PF07883:Cupin domain|PF00107:Zinc-binding dehydrogenase|PF08240:Alcohol dehydrogenase GroES-like domain|PF02627:Carboxymuconolactone decarboxylase family | 3.583772165 | 1.42E-43 | 2.95E-42 |
| FQU82_RS16915 | FQU82_RS16915 | xanthine phosphoribosyltransferase | -2.124358479 | 2.65E-43 | 5.46E-42 |
| FQU82_RS17890 | FQU82_RS17890 | SDR family NAD(P)-dependent oxidoreductase | -2.758618108 | 3.54E-43 | 7.26E-42 |
| FQU82_RS15575 | FQU82_RS15575 | flavodoxin family protein | 2.993544804 | 5.20E-43 | 1.06E-41 |
| FQU82_RS15805 | FQU82_RS15805 | undecaprenyl-diphosphate phosphatase | -2.480771981 | 5.55E-43 | 1.12E-41 |
| FQU82_RS15885 | gatA | Asp-tRNA(Asn)/Glu-tRNA(Gln) amidotransferase subunit GatA | -2.073558627 | 1.04E-42 | 2.10E-41 |
| FQU82_RS18845 | maiA | maleylacetoacetate isomerase | 2.850517868 | 1.61E-42 | 3.22E-41 |
| FQU82_RS07585 | FQU82_RS07585 | type I secretion system permease/ATPase | -2.148570142 | 3.18E-42 | 6.34E-41 |
| FQU82_RS14825 | queC | 7-cyano-7-deazaguanine synthase QueC | -2.053651948 | 3.33E-42 | 6.59E-41 |
| FQU82_RS16550 | yajC | preprotein translocase subunit YajC | -2.14245529 | 8.76E-42 | 1.72E-40 |
| Novel00148 | - | PF20169:Family of unknown function (DUF6537)|PF01558:Pyruvate ferredoxin/flavodoxin oxidoreductase | 3.572243493 | 1.49E-41 | 2.91E-40 |
| FQU82_RS00895 | FQU82_RS00895 | phosphomannomutase/phosphoglucomutase | -2.054998303 | 4.44E-41 | 8.66E-40 |
| FQU82_RS14820 | queE | 7-carboxy-7-deazaguanine synthase QueE | -2.193763955 | 4.77E-41 | 9.24E-40 |
| FQU82_RS01910 | rplA | 50S ribosomal protein L1 | -2.719970042 | 6.65E-41 | 1.28E-39 |
| FQU82_RS13895 | metF | methylenetetrahydrofolate reductase [NAD(P)H] | -2.193548143 | 1.34E-40 | 2.56E-39 |
| FQU82_RS03425 | FQU82_RS03425 | hypothetical protein | -1.985192188 | 1.38E-40 | 2.63E-39 |
| Novel00014 | - | PF00296:Luciferase-like monooxygenase|PF00005:ABC transporter|PF00528:Binding-protein-dependent transport system inner membrane component|PF13379:NMT1-like family | 3.556829125 | 2.65E-40 | 5.03E-39 |
| Novel00269 | - | PF00679:Elongation factor G C-terminus|PF00009:Elongation factor Tu GTP binding domain|PF03144:Elongation factor Tu domain 2|PF21018:TypA/BipA C-terminal domain | -2.533709872 | 7.69E-40 | 1.45E-38 |
| FQU82_RS12235 | pepN | aminopeptidase N | 1.760772578 | 8.35E-40 | 1.57E-38 |
| FQU82_RS18795 | FQU82_RS18795 | amino acid permease | 1.991326746 | 1.11E-39 | 2.06E-38 |
| FQU82_RS02910 | tig | trigger factor | -1.930288884 | 1.18E-39 | 2.20E-38 |
| FQU82_RS10895 | FQU82_RS10895 | DUF637 domain-containing protein | -2.953046298 | 1.55E-39 | 2.86E-38 |
| FQU82_RS10345 | antC | anthranilate 1,2-dioxygenase electron transfer component AntC | 5.186484697 | 2.18E-39 | 4.00E-38 |
| FQU82_RS16215 | FQU82_RS16215 | nitrite/sulfite reductase | -1.761862349 | 3.20E-39 | 5.84E-38 |
| FQU82_RS15330 | folD | bifunctional methylenetetrahydrofolate dehydrogenase/methenyltetrahydrofolate cyclohydrolase FolD | -2.057723645 | 3.89E-39 | 7.05E-38 |
| FQU82_RS00205 | rnpA | ribonuclease P protein component | -2.115959966 | 3.91E-39 | 7.05E-38 |
| FQU82_RS16545 | tgt | tRNA guanosine(34) transglycosylase Tgt | -1.947140138 | 3.92E-39 | 7.05E-38 |
| FQU82_RS17440 | FQU82_RS17440 | Glu/Leu/Phe/Val dehydrogenase | -2.030908356 | 5.29E-39 | 9.45E-38 |
| FQU82_RS17125 | rpsQ | 30S ribosomal protein S17 | -2.248280352 | 5.34E-39 | 9.49E-38 |
| FQU82_RS09335 | putP | sodium/proline symporter PutP | -1.923057656 | 5.51E-39 | 9.76E-38 |
| FQU82_RS12280 | FQU82_RS12280 | aldehyde dehydrogenase family protein | -2.241014457 | 9.89E-39 | 1.74E-37 |
| FQU82_RS13840 | rpsB | 30S ribosomal protein S2 | -2.125699995 | 1.21E-38 | 2.13E-37 |
| FQU82_RS14780 | FQU82_RS14780 | sulfate ABC transporter substrate-binding protein | 1.644860863 | 1.54E-38 | 2.69E-37 |
| FQU82_RS14335 | pstA | phosphate ABC transporter permease PstA | -1.934770529 | 2.64E-38 | 4.58E-37 |
| FQU82_RS17095 | rplF | 50S ribosomal protein L6 | -2.42025659 | 2.87E-38 | 4.95E-37 |
| FQU82_RS00215 | yidC | membrane protein insertase YidC | -1.864941871 | 6.10E-38 | 1.05E-36 |
| FQU82_RS01340 | atpD | F0F1 ATP synthase subunit beta | -2.419669818 | 8.66E-38 | 1.48E-36 |
| FQU82_RS02515 | katG | catalase/peroxidase HPI | 1.698943591 | 2.25E-37 | 3.82E-36 |
| FQU82_RS07590 | FQU82_RS07590 | HlyD family type I secretion periplasmic adaptor subunit | -2.104930121 | 2.60E-37 | 4.40E-36 |
| Novel00061 | - | PF13602:Zinc-binding dehydrogenase | 4.284452642 | 3.37E-37 | 5.68E-36 |
| FQU82_RS17070 | secY | preprotein translocase subunit SecY | -2.08733076 | 5.72E-37 | 9.59E-36 |
| FQU82_RS07000 | trmB | tRNA (guanosine(46)-N7)-methyltransferase TrmB | -2.795121734 | 9.86E-37 | 1.64E-35 |
| FQU82_RS18790 | hutI | imidazolonepropionase | 1.655509717 | 9.98E-37 | 1.66E-35 |
| FQU82_RS03560 | raiA | ribosome-associated translation inhibitor RaiA | 2.076135204 | 1.33E-36 | 2.19E-35 |
| FQU82_RS19025 | FQU82_RS19025 | efflux RND transporter periplasmic adaptor subunit | 1.966671858 | 1.40E-36 | 2.30E-35 |
| FQU82_RS02895 | leuA | 2-isopropylmalate synthase | 1.709609806 | 1.51E-36 | 2.47E-35 |
| FQU82_RS17430 | astA | arginine N-succinyltransferase | -2.261973692 | 1.64E-36 | 2.67E-35 |
| FQU82_RS09285 | tkt | transketolase | -1.788622028 | 2.01E-36 | 3.25E-35 |
| FQU82_RS00875 | galU | UTP--glucose-1-phosphate uridylyltransferase GalU | -1.665846169 | 1.33E-35 | 2.14E-34 |
| FQU82_RS16450 | aspS | aspartate--tRNA ligase | -1.90546086 | 1.65E-35 | 2.65E-34 |
| FQU82_RS17425 | astD | succinylglutamate-semialdehyde dehydrogenase | -2.034464673 | 2.37E-35 | 3.78E-34 |
| Novel00291 | - | PF03947:Ribosomal Proteins L2, C-terminal domain|PF00181:Ribosomal Proteins L2, RNA binding domain|PF07650:KH domain|PF00189:Ribosomal protein S3, C-terminal domain|PF00297:Ribosomal protein L3|PF03118:Bacterial RNA polymerase, alpha chain C terminal domain|PF01193:RNA polymerase Rpb3/Rpb11 dimerisation domain|PF01000:RNA polymerase Rpb3/RpoA insert domain|PF00573:Ribosomal protein L4/L1 family|PF00344:SecY|PF00281:Ribosomal protein L5|PF00673:ribosomal L5P family C-terminus|PF00163:Ribosomal protein S4/S9 N-terminal domain|PF01479:S4 domain | -2.019961599 | 3.58E-35 | 5.70E-34 |
| FQU82_RS01075 | FQU82_RS01075 | amino acid permease | -2.207049186 | 4.08E-35 | 6.47E-34 |
| FQU82_RS01690 | thrC | threonine synthase | -1.681186593 | 5.69E-35 | 8.97E-34 |
| FQU82_RS14340 | pstC | phosphate ABC transporter permease subunit PstC | -2.336797073 | 8.41E-35 | 1.32E-33 |
| FQU82_RS16290 | FQU82_RS16290 | peroxiredoxin | 1.503844282 | 6.54E-34 | 1.02E-32 |
| FQU82_RS13800 | mltB | lytic murein transglycosylase B | -2.360284746 | 8.54E-34 | 1.33E-32 |
| FQU82_RS15400 | carB | carbamoyl-phosphate synthase large subunit | -2.074902974 | 1.05E-33 | 1.62E-32 |
| FQU82_RS16025 | dapE | succinyl-diaminopimelate desuccinylase | -2.084022968 | 1.54E-33 | 2.37E-32 |
| FQU82_RS18495 | FQU82_RS18495 | TonB-dependent siderophore receptor | -2.905579679 | 2.22E-33 | 3.41E-32 |
| FQU82_RS14870 | purL | phosphoribosylformylglycinamidine synthase | -1.673566263 | 2.34E-33 | 3.58E-32 |
| FQU82_RS06880 | alaS | alanine--tRNA ligase | -1.447165487 | 2.38E-33 | 3.63E-32 |
| FQU82_RS17565 | rplS | 50S ribosomal protein L19 | -1.465285382 | 2.73E-33 | 4.14E-32 |
| Novel00193 | - | PF17432:Domain of unknown function (DUF3458_C) ARM repeats|PF11940:Domain of unknown function (DUF3458) Ig-like fold|PF01433:Peptidase family M1 domain|PF17900:Peptidase M1 N-terminal domain | 2.223468259 | 3.03E-33 | 4.58E-32 |
| FQU82_RS14940 | FQU82_RS14940 | RNA polymerase sigma factor | 1.598588024 | 4.16E-33 | 6.25E-32 |
| FQU82_RS04495 | fusA | elongation factor G | -2.047155502 | 8.84E-33 | 1.32E-31 |
| FQU82_RS04580 | rep | DNA helicase Rep | -1.896010766 | 1.01E-32 | 1.51E-31 |
| FQU82_RS01060 | alr | alanine racemase | 1.960653041 | 1.03E-32 | 1.52E-31 |
| FQU82_RS15870 | mreC | rod shape-determining protein MreC | -1.711134073 | 1.19E-32 | 1.75E-31 |
| FQU82_RS06560 | FQU82_RS06560 | adenosine deaminase | -1.82451218 | 1.53E-32 | 2.25E-31 |
| FQU82_RS02935 | FQU82_RS02935 | fumarate hydratase | -1.594161852 | 1.84E-32 | 2.69E-31 |
| Novel00164 | - | PF01592:NifU-like N terminal domain|PF02082:Iron-dependent Transcriptional regulator|PF00266:Aminotransferase class-V|PF01521:Iron-sulphur cluster biosynthesis | 2.459427813 | 2.47E-32 | 3.60E-31 |
| FQU82_RS15160 | FQU82_RS15160 | FdhF/YdeP family oxidoreductase | 1.636702029 | 3.13E-32 | 4.54E-31 |
| FQU82_RS00900 | lldP | L-lactate permease | 2.153278185 | 7.34E-32 | 1.06E-30 |
| FQU82_RS02135 | tpiA | triose-phosphate isomerase | -1.97670271 | 9.09E-32 | 1.31E-30 |
| FQU82_RS18145 | FQU82_RS18145 | pirin family protein | 2.142969806 | 1.32E-31 | 1.89E-30 |
| FQU82_RS16870 | FQU82_RS16870 | malate dehydrogenase | -1.759593744 | 1.53E-31 | 2.18E-30 |
| FQU82_RS02720 | rpmB | 50S ribosomal protein L28 | -1.697500602 | 1.91E-31 | 2.71E-30 |
| FQU82_RS11215 | FQU82_RS11215 | DUF6438 domain-containing protein | -2.13577351 | 2.08E-31 | 2.95E-30 |
| FQU82_RS11710 | bamA | outer membrane protein assembly factor BamA | -1.533399316 | 2.24E-31 | 3.16E-30 |
| FQU82_RS05935 | FQU82_RS05935 | cation diffusion facilitator family transporter | -1.585951337 | 3.17E-31 | 4.46E-30 |
| FQU82_RS12675 | mnmG | tRNA uridine-5-carboxymethylaminomethyl(34) synthesis enzyme MnmG | -1.599445862 | 4.35E-31 | 6.09E-30 |
| FQU82_RS14740 | lepA | translation elongation factor 4 | -1.766425399 | 5.82E-31 | 8.12E-30 |
| FQU82_RS01895 | secE | preprotein translocase subunit SecE | -1.598989708 | 9.19E-31 | 1.28E-29 |
| FQU82_RS09390 | FQU82_RS09390 | cupin domain-containing protein | -2.186237182 | 9.25E-31 | 1.28E-29 |
| FQU82_RS17875 | FQU82_RS17875 | SEL1-like repeat protein | 1.49430673 | 1.68E-30 | 2.32E-29 |
| FQU82_RS01560 | ppa | inorganic diphosphatase | -1.646604927 | 2.65E-30 | 3.64E-29 |
| FQU82_RS09750 | FQU82_RS09750 | IscS subfamily cysteine desulfurase | 1.570209712 | 3.38E-30 | 4.62E-29 |
| FQU82_RS03435 | rho | transcription termination factor Rho | -1.656139685 | 3.80E-30 | 5.18E-29 |
| FQU82_RS00125 | tyrS | tyrosine--tRNA ligase | -1.596222019 | 4.39E-30 | 5.95E-29 |
| FQU82_RS11465 | FQU82_RS11465 | cyd operon YbgE family protein | -1.883376795 | 4.56E-30 | 6.16E-29 |
| FQU82_RS15995 | FQU82_RS15995 | tRNA-Asp | -2.440992625 | 4.71E-30 | 6.34E-29 |
| FQU82_RS02960 | FQU82_RS02960 | gluconate:H+ symporter | -1.642804047 | 4.84E-30 | 6.49E-29 |
| FQU82_RS13845 | map | type I methionyl aminopeptidase | -1.670078942 | 5.09E-30 | 6.80E-29 |
| FQU82_RS11730 | uppS | polyprenyl diphosphate synthase | -1.962809586 | 5.60E-30 | 7.45E-29 |
| FQU82_RS14980 | purN | phosphoribosylglycinamide formyltransferase | -1.802977655 | 7.52E-30 | 9.96E-29 |
| FQU82_RS17965 | hisA | 1-(5-phosphoribosyl)-5-[(5-phosphoribosylamino)methylideneamino]imidazole-4-carboxamide isomerase | -1.571142374 | 9.29E-30 | 1.23E-28 |
| FQU82_RS17900 | FQU82_RS17900 | Tex family protein | -1.821454356 | 1.04E-29 | 1.37E-28 |
| FQU82_RS17525 | serA | phosphoglycerate dehydrogenase | -1.435514941 | 1.13E-29 | 1.48E-28 |
| FQU82_RS11790 | FQU82_RS11790 | D-amino acid dehydrogenase | 1.6713946 | 1.59E-29 | 2.08E-28 |
| Novel00010 | - | - | 4.373881642 | 1.65E-29 | 2.15E-28 |
| FQU82_RS06530 | FQU82_RS06530 | pirin family protein | 3.089098027 | 1.85E-29 | 2.40E-28 |
| FQU82_RS02305 | FQU82_RS02305 | Hsp33 family molecular chaperone HslO | 1.438317514 | 3.03E-29 | 3.91E-28 |
| FQU82_RS14060 | hemE | uroporphyrinogen decarboxylase | -2.291591364 | 4.17E-29 | 5.36E-28 |
| FQU82_RS16165 | FQU82_RS16165 | class I SAM-dependent methyltransferase | -2.525996947 | 4.23E-29 | 5.43E-28 |
| FQU82_RS01920 | rplL | 50S ribosomal protein L7/L12 | -2.490310099 | 4.85E-29 | 6.19E-28 |
| FQU82_RS15640 | adeI | multidrug efflux RND transporter periplasmic adaptor subunit AdeI | -1.63617458 | 7.74E-29 | 9.85E-28 |
| FQU82_RS18620 | FQU82_RS18620 | type VI secretion system Vgr family protein | -3.127777528 | 9.11E-29 | 1.16E-27 |
| FQU82_RS11725 | FQU82_RS11725 | phosphatidate cytidylyltransferase | -1.910239356 | 9.90E-29 | 1.25E-27 |
| FQU82_RS14215 | efp | elongation factor P | -1.501321948 | 1.63E-28 | 2.05E-27 |
| FQU82_RS11545 | FQU82_RS11545 | hypothetical protein | 2.539409531 | 1.81E-28 | 2.26E-27 |
| FQU82_RS05325 | lysS | lysine--tRNA ligase | -1.88884074 | 2.35E-28 | 2.94E-27 |
| FQU82_RS10175 | FQU82_RS10175 | fumarate reductase/succinate dehydrogenase flavoprotein subunit | 2.882485461 | 3.01E-28 | 3.75E-27 |
| FQU82_RS02785 | lgt | prolipoprotein diacylglyceryl transferase | -1.721309588 | 4.78E-28 | 5.93E-27 |
| FQU82_RS07865 | FQU82_RS07865 | type VI secretion system tube protein Hcp | 1.39213668 | 5.06E-28 | 6.26E-27 |
| FQU82_RS17045 | rpoA | DNA-directed RNA polymerase subunit alpha | -2.326130539 | 5.14E-28 | 6.33E-27 |
| FQU82_RS02165 | infB | translation initiation factor IF-2 | -1.682569931 | 5.50E-28 | 6.76E-27 |
| FQU82_RS00330 | grpE | nucleotide exchange factor GrpE | 1.564368829 | 5.73E-28 | 7.01E-27 |
| FQU82_RS15875 | FQU82_RS15875 | rod shape-determining protein | -1.829202298 | 5.78E-28 | 7.04E-27 |
| FQU82_RS08635 | FQU82_RS08635 | serine acetyltransferase | 3.142043573 | 9.53E-28 | 1.16E-26 |
| FQU82_RS03260 | rsfS | ribosome silencing factor | 1.389684032 | 9.92E-28 | 1.20E-26 |
| Novel00009 | - | PF00012:Hsp70 protein|PF01025:GrpE | 2.554679365 | 1.04E-27 | 1.26E-26 |
| FQU82_RS18055 | FQU82_RS18055 | hypothetical protein | 4.244790715 | 1.13E-27 | 1.36E-26 |
| FQU82_RS17570 | trmD | tRNA (guanosine(37)-N1)-methyltransferase TrmD | -1.529533072 | 1.23E-27 | 1.47E-26 |
| FQU82_RS15965 | FQU82_RS15965 | PepSY domain-containing protein | -1.711257649 | 1.65E-27 | 1.97E-26 |
| FQU82_RS11735 | frr | ribosome recycling factor | -1.728986759 | 3.48E-27 | 4.14E-26 |
| FQU82_RS03440 | trxA | thioredoxin | 1.631391782 | 4.60E-27 | 5.45E-26 |
| FQU82_RS09015 | FQU82_RS09015 | ATP-binding cassette domain-containing protein | 3.104023496 | 5.52E-27 | 6.52E-26 |
| FQU82_RS04075 | FQU82_RS04075 | 5'-methylthioadenosine/S-adenosylhomocysteine nucleosidase | -3.105022603 | 8.44E-27 | 9.94E-26 |
| FQU82_RS10515 | adeB | multidrug efflux RND transporter permease subunit AdeB | -1.668711522 | 8.80E-27 | 1.03E-25 |
| FQU82_RS18280 | omp33-36 | porin Omp33-36 | -1.527900773 | 8.80E-27 | 1.03E-25 |
| FQU82_RS11745 | rimO | 30S ribosomal protein S12 methylthiotransferase RimO | -1.858237413 | 1.15E-26 | 1.34E-25 |
| FQU82_RS14630 | FQU82_RS14630 | lipocalin family protein | 1.763363409 | 1.29E-26 | 1.50E-25 |
| FQU82_RS17435 | FQU82_RS17435 | aspartate aminotransferase family protein | -2.157579528 | 1.65E-26 | 1.91E-25 |
| FQU82_RS14975 | purM | phosphoribosylformylglycinamidine cyclo-ligase | -1.775927362 | 1.66E-26 | 1.92E-25 |
| FQU82_RS03820 | FQU82_RS03820 | methionine synthase | -1.693523882 | 2.04E-26 | 2.35E-25 |
| FQU82_RS01055 | FQU82_RS01055 | D-amino acid dehydrogenase | 1.659601827 | 2.26E-26 | 2.60E-25 |
| FQU82_RS16515 | FQU82_RS16515 | HAMP domain-containing sensor histidine kinase | 1.518294993 | 2.35E-26 | 2.68E-25 |
| FQU82_RS15010 | FQU82_RS15010 | lipoprotein-releasing ABC transporter permease subunit | -1.530987987 | 2.60E-26 | 2.96E-25 |
| FQU82_RS15550 | FQU82_RS15550 | DUF493 domain-containing protein | 2.023490464 | 2.75E-26 | 3.12E-25 |
| FQU82_RS02735 | purU | formyltetrahydrofolate deformylase | -1.662389465 | 3.29E-26 | 3.72E-25 |
| FQU82_RS05500 | FQU82_RS05500 | OmpA family protein | -2.970117745 | 4.01E-26 | 4.52E-25 |
| FQU82_RS04585 | dut | dUTP diphosphatase | -3.090402435 | 5.38E-26 | 6.05E-25 |
| FQU82_RS02160 | nusA | transcription termination factor NusA | -1.724167208 | 6.31E-26 | 7.07E-25 |
| Novel00230 | - | PF03466:LysR substrate binding domain|PF00126:Bacterial regulatory helix-turn-helix protein, lysR family|PF00375:Sodium:dicarboxylate symporter family | 2.40400156 | 6.61E-26 | 7.39E-25 |
| FQU82_RS12505 | FQU82_RS12505 | AMP-binding protein | 2.739559515 | 1.29E-25 | 1.44E-24 |
| FQU82_RS00325 | dnaK | molecular chaperone DnaK | 1.804701548 | 1.40E-25 | 1.55E-24 |
| FQU82_RS04255 | fabG | 3-oxoacyl-ACP reductase FabG | -1.394946463 | 1.86E-25 | 2.06E-24 |
| FQU82_RS18310 | FQU82_RS18310 | hypothetical protein | 2.201123049 | 1.90E-25 | 2.09E-24 |
| FQU82_RS01985 | FQU82_RS01985 | DsbA family oxidoreductase | 1.699578725 | 1.99E-25 | 2.19E-24 |
| FQU82_RS11665 | FQU82_RS11665 | S4 domain-containing protein | 1.709303117 | 2.66E-25 | 2.91E-24 |
| FQU82_RS02950 | edd | phosphogluconate dehydratase | -1.637143911 | 2.83E-25 | 3.09E-24 |
| FQU82_RS12685 | prmA | 50S ribosomal protein L11 methyltransferase | -1.60756805 | 3.01E-25 | 3.28E-24 |
| FQU82_RS13245 | FQU82_RS13245 | dicarboxylate/amino acid:cation symporter | -1.485259735 | 3.42E-25 | 3.71E-24 |
| FQU82_RS05230 | nfuA | Fe-S biogenesis protein NfuA | 1.477720866 | 3.89E-25 | 4.21E-24 |
| FQU82_RS17370 | glyS | glycine--tRNA ligase subunit beta | -1.743178789 | 5.85E-25 | 6.31E-24 |
| FQU82_RS18740 | ribE | 6,7-dimethyl-8-ribityllumazine synthase | -1.318989178 | 7.35E-25 | 7.91E-24 |
| FQU82_RS03495 | FQU82_RS03495 | organic hydroperoxide resistance protein | 1.727393441 | 7.49E-25 | 8.03E-24 |
| FQU82_RS02290 | FQU82_RS02290 | chaperone modulator CbpM | 1.454803166 | 1.16E-24 | 1.24E-23 |
| FQU82_RS10060 | aroC | chorismate synthase | -1.697372128 | 1.20E-24 | 1.28E-23 |
| FQU82_RS08000 | FQU82_RS08000 | GntR family transcriptional regulator | 2.091818232 | 1.24E-24 | 1.32E-23 |
| FQU82_RS06965 | FQU82_RS06965 | dihydroorotase | -1.460991309 | 1.62E-24 | 1.72E-23 |
| FQU82_RS18515 | FQU82_RS18515 | DUF2147 domain-containing protein | 1.222783492 | 1.84E-24 | 1.94E-23 |
| FQU82_RS08810 | FQU82_RS08810 | ATP-binding cassette domain-containing protein | 1.490338404 | 2.05E-24 | 2.16E-23 |
| FQU82_RS06960 | FQU82_RS06960 | aspartate carbamoyltransferase catalytic subunit | -1.883943136 | 3.14E-24 | 3.30E-23 |
| FQU82_RS14040 | FQU82_RS14040 | extended-spectrum class C beta-lactamase ADC-158 | -1.40903976 | 3.32E-24 | 3.48E-23 |
| FQU82_RS02755 | msrA | peptide-methionine (S)-S-oxide reductase MsrA | -1.407604638 | 4.12E-24 | 4.30E-23 |
| FQU82_RS09875 | FQU82_RS09875 | HAD family hydrolase | 1.603147324 | 4.47E-24 | 4.65E-23 |
| FQU82_RS03385 | rpmI | 50S ribosomal protein L35 | -1.38216286 | 5.87E-24 | 6.09E-23 |
| FQU82_RS15930 | FQU82_RS15930 | alanine/glycine:cation symporter family protein | -2.57808006 | 6.41E-24 | 6.63E-23 |
| FQU82_RS15235 | ttcA | tRNA 2-thiocytidine(32) synthetase TtcA | -1.879027099 | 7.17E-24 | 7.39E-23 |
| FQU82_RS02020 | lpxO | lipid A hydroxylase LpxO | -1.531119737 | 8.41E-24 | 8.65E-23 |
| FQU82_RS12580 | pgaA | poly-beta-1,6 N-acetyl-D-glucosamine exporter porin PgaA | 3.442502643 | 1.45E-23 | 1.49E-22 |
| FQU82_RS14470 | FQU82_RS14470 | L-cystine transporter | 1.687927535 | 1.87E-23 | 1.91E-22 |
| FQU82_RS08900 | FQU82_RS08900 | hypothetical protein | -1.434881745 | 2.16E-23 | 2.21E-22 |
| FQU82_RS05195 | FQU82_RS05195 | inorganic phosphate transporter | -1.29026258 | 2.28E-23 | 2.32E-22 |
| FQU82_RS02470 | FQU82_RS02470 | long-chain-acyl-CoA synthetase | -1.276068967 | 2.35E-23 | 2.38E-22 |
| FQU82_RS08365 | FQU82_RS08365 | enoyl-CoA hydratase | -2.04794863 | 2.82E-23 | 2.85E-22 |
| FQU82_RS11490 | FQU82_RS11490 | hypothetical protein | 1.370418216 | 2.85E-23 | 2.87E-22 |
| FQU82_RS18500 | pyrE | orotate phosphoribosyltransferase | -1.818724676 | 3.11E-23 | 3.12E-22 |
| FQU82_RS12720 | FQU82_RS12720 | methionine ABC transporter permease | -1.502982487 | 3.63E-23 | 3.63E-22 |
| Novel00235 | - | PF12344:Ultra-violet resistance protein B|PF02151:UvrB/uvrC motif|PF04851:Type III restriction enzyme, res subunit|PF17757:UvrB interaction domain|PF00271:Helicase conserved C-terminal domain|PF08212:Lipocalin-like domain | 2.700838716 | 4.95E-23 | 4.94E-22 |
| FQU82_RS12715 | FQU82_RS12715 | methionine ABC transporter ATP-binding protein | -1.642636245 | 5.08E-23 | 5.06E-22 |
| FQU82_RS09220 | FQU82_RS09220 | hypothetical protein | 1.449570039 | 5.41E-23 | 5.37E-22 |
| FQU82_RS14265 | FQU82_RS14265 | peptidoglycan DD-metalloendopeptidase family protein | -1.636078834 | 5.85E-23 | 5.79E-22 |
| FQU82_RS00345 | FQU82_RS00345 | hypothetical protein | -2.151842112 | 7.91E-23 | 7.81E-22 |
| FQU82_RS03125 | leuS | leucine--tRNA ligase | -1.322673308 | 9.41E-23 | 9.27E-22 |
| FQU82_RS14945 | FQU82_RS14945 | hypothetical protein | 1.565342526 | 9.62E-23 | 9.45E-22 |
| FQU82_RS16525 | queA | tRNA preQ1(34) S-adenosylmethionine ribosyltransferase-isomerase QueA | -2.106208518 | 1.05E-22 | 1.03E-21 |
| FQU82_RS11460 | cydX | cytochrome bd-I oxidase subunit CydX | -2.412307151 | 1.21E-22 | 1.18E-21 |
| FQU82_RS14460 | FQU82_RS14460 | pitrilysin family protein | -1.451995793 | 1.22E-22 | 1.19E-21 |
| FQU82_RS15610 | rpmA | 50S ribosomal protein L27 | -1.361530433 | 1.28E-22 | 1.25E-21 |
| FQU82_RS04485 | rpsL | 30S ribosomal protein S12 | -1.340195607 | 1.40E-22 | 1.36E-21 |
| Novel00356 | - | PF00903:Glyoxalase/Bleomycin resistance protein/Dioxygenase superfamily|PF14696:Hydroxyphenylpyruvate dioxygenase, HPPD, N-terminal | 3.663579314 | 1.53E-22 | 1.48E-21 |
| FQU82_RS11475 | FQU82_RS11475 | nitroreductase family protein | 1.431581506 | 2.12E-22 | 2.04E-21 |
| FQU82_RS11970 | FQU82_RS11970 | FMN-binding glutamate synthase family protein | -1.865775389 | 2.18E-22 | 2.10E-21 |
| FQU82_RS15680 | FQU82_RS15680 | valine--tRNA ligase | -1.476334557 | 3.41E-22 | 3.26E-21 |
| FQU82_RS15570 | FQU82_RS15570 | APC family permease | -1.522892539 | 3.67E-22 | 3.51E-21 |
| FQU82_RS09175 | FQU82_RS09175 | cytosine permease | 1.867972761 | 3.98E-22 | 3.79E-21 |
| FQU82_RS10730 | FQU82_RS10730 | phosphoribosyltransferase family protein | -2.054961916 | 4.08E-22 | 3.88E-21 |
| FQU82_RS02955 | eda | bifunctional 4-hydroxy-2-oxoglutarate aldolase/2-dehydro-3-deoxy-phosphogluconate aldolase | -1.615997126 | 5.26E-22 | 4.99E-21 |
| Novel00331 | - | PF00296:Luciferase-like monooxygenase | 3.204217253 | 5.79E-22 | 5.47E-21 |
| FQU82_RS16860 | FQU82_RS16860 | hypothetical protein | 1.363577968 | 5.90E-22 | 5.56E-21 |
| FQU82_RS17495 | FQU82_RS17495 | MFS transporter | -1.456412851 | 6.50E-22 | 6.11E-21 |
| FQU82_RS04335 | ispE | 4-(cytidine 5'-diphospho)-2-C-methyl-D-erythritol kinase | -1.471354317 | 7.09E-22 | 6.65E-21 |
| FQU82_RS06950 | rlmKL | bifunctional 23S rRNA (guanine(2069)-N(7))-methyltransferase RlmK/23S rRNA (guanine(2445)-N(2))-methyltransferase RlmL | -1.340187107 | 8.48E-22 | 7.92E-21 |
| FQU82_RS17075 | rplO | 50S ribosomal protein L15 | -1.894135623 | 9.57E-22 | 8.92E-21 |
| FQU82_RS00890 | galE | UDP-glucose 4-epimerase GalE | -1.367956688 | 1.29E-21 | 1.20E-20 |
| FQU82_RS18090 | FQU82_RS18090 | helix-turn-helix transcriptional regulator | 2.115754993 | 2.24E-21 | 2.08E-20 |
| FQU82_RS12635 | rplI | 50S ribosomal protein L9 | -1.766940134 | 2.32E-21 | 2.15E-20 |
| FQU82_RS03555 | FQU82_RS03555 | RNA polymerase factor sigma-54 | 1.411434859 | 2.45E-21 | 2.26E-20 |
| FQU82_RS12530 | prfA | peptide chain release factor 1 | -1.387981497 | 3.64E-21 | 3.35E-20 |
| FQU82_RS10255 | FQU82_RS10255 | 3-oxoacid CoA-transferase subunit B | 1.579079435 | 4.46E-21 | 4.09E-20 |
| FQU82_RS11940 | accC | acetyl-CoA carboxylase biotin carboxylase subunit | -1.672129074 | 5.24E-21 | 4.79E-20 |
| Novel00068 | - | PF00682:HMGL-like|PF08502:LeuA allosteric (dimerisation) domain | 2.343038389 | 5.64E-21 | 5.14E-20 |
| FQU82_RS12620 | cyoE | heme o synthase | -1.857439312 | 6.02E-21 | 5.48E-20 |
| FQU82_RS17420 | astB | N-succinylarginine dihydrolase | -1.639430461 | 7.58E-21 | 6.89E-20 |
| FQU82_RS07580 | FQU82_RS07580 | TolC family protein | -2.260428034 | 9.18E-21 | 8.31E-20 |
| FQU82_RS04490 | rpsG | 30S ribosomal protein S7 | -1.428972515 | 1.02E-20 | 9.22E-20 |
| FQU82_RS16415 | FQU82_RS16415 | glutathione S-transferase family protein | 4.096357039 | 1.03E-20 | 9.23E-20 |
| FQU82_RS15890 | gatB | Asp-tRNA(Asn)/Glu-tRNA(Gln) amidotransferase subunit GatB | -1.574039113 | 1.12E-20 | 1.01E-19 |
| FQU82_RS08370 | FQU82_RS08370 | acyl-CoA dehydrogenase family protein | -1.692975197 | 1.29E-20 | 1.15E-19 |
| FQU82_RS10350 | antB | anthranilate 1,2-dioxygenase small subunit | 6.327413446 | 1.35E-20 | 1.21E-19 |
| FQU82_RS17400 | FQU82_RS17400 | S8 family serine peptidase | 2.149919187 | 1.36E-20 | 1.21E-19 |
| FQU82_RS12175 | FQU82_RS12175 | TonB-dependent receptor | -3.210733047 | 1.64E-20 | 1.46E-19 |
| FQU82_RS09565 | pyrF | orotidine-5'-phosphate decarboxylase | -1.883551062 | 1.91E-20 | 1.70E-19 |
| FQU82_RS01070 | FQU82_RS01070 | amino acid permease | 1.300351612 | 2.21E-20 | 1.96E-19 |
| FQU82_RS09325 | putA | trifunctional transcriptional regulator/proline dehydrogenase/L-glutamate gamma-semialdehyde dehydrogenase | -1.223170333 | 2.23E-20 | 1.97E-19 |
| FQU82_RS02705 | FQU82_RS02705 | hypothetical protein | 2.047032975 | 2.60E-20 | 2.29E-19 |
| FQU82_RS00285 | mpl | UDP-N-acetylmuramate:L-alanyl-gamma-D-glutamyl-meso-diaminopimelate ligase | -1.344939467 | 2.80E-20 | 2.45E-19 |
| FQU82_RS12310 | FQU82_RS12310 | peptidylprolyl isomerase | -1.302482837 | 3.18E-20 | 2.79E-19 |
| Novel00342 | - | PF09917:Uncharacterized protein conserved in bacteria (DUF2147) | 1.907977883 | 3.19E-20 | 2.79E-19 |
| FQU82_RS02770 | thyA | thymidylate synthase | -1.221592113 | 4.37E-20 | 3.81E-19 |
| FQU82_RS17300 | FQU82_RS17300 | inositol monophosphatase family protein | -1.287449412 | 4.46E-20 | 3.87E-19 |
| FQU82_RS14735 | lepB | signal peptidase I | -1.494223509 | 4.99E-20 | 4.32E-19 |
| FQU82_RS09740 | iscA | iron-sulfur cluster assembly protein IscA | 2.136381045 | 5.92E-20 | 5.12E-19 |
| FQU82_RS02725 | FQU82_RS02725 | coniferyl aldehyde dehydrogenase | 1.062525773 | 6.67E-20 | 5.75E-19 |
| FQU82_RS16990 | FQU82_RS16990 | M3 family metallopeptidase | 1.156153523 | 7.86E-20 | 6.77E-19 |
| FQU82_RS13385 | FQU82_RS13385 | serine hydroxymethyltransferase | -1.391723738 | 8.63E-20 | 7.41E-19 |
| FQU82_RS13080 | tsaD | tRNA (adenosine(37)-N6)-threonylcarbamoyltransferase complex transferase subunit TsaD | -1.659774789 | 9.13E-20 | 7.82E-19 |
| FQU82_RS15470 | sucC | ADP-forming succinate--CoA ligase subunit beta | -1.634444681 | 1.01E-19 | 8.65E-19 |
| FQU82_RS00140 | erpA | iron-sulfur cluster insertion protein ErpA | 1.649952595 | 1.06E-19 | 9.06E-19 |
| FQU82_RS03070 | grxC | glutaredoxin 3 | 1.203321372 | 1.07E-19 | 9.12E-19 |
| FQU82_RS17575 | rimM | ribosome maturation factor RimM | -1.604737294 | 1.24E-19 | 1.05E-18 |
| FQU82_RS18785 | hutG | formimidoylglutamase | 1.338186432 | 1.54E-19 | 1.31E-18 |
| FQU82_RS01695 | FQU82_RS01695 | homoserine dehydrogenase | -1.162339249 | 1.63E-19 | 1.37E-18 |
| FQU82_RS18475 | murC | UDP-N-acetylmuramate--L-alanine ligase | -1.314112999 | 1.66E-19 | 1.40E-18 |
| FQU82_RS10295 | FQU82_RS10295 | GntP family permease | 1.558649894 | 1.86E-19 | 1.56E-18 |
| FQU82_RS16555 | secD | protein translocase subunit SecD | -1.651325133 | 2.54E-19 | 2.13E-18 |
| FQU82_RS18745 | nusB | transcription antitermination factor NusB | -1.154253188 | 2.72E-19 | 2.28E-18 |
| FQU82_RS02940 | pta | phosphate acetyltransferase | -1.100919917 | 2.90E-19 | 2.42E-18 |
| FQU82_RS09775 | FQU82_RS09775 | SurA N-terminal domain-containing protein | -1.302606607 | 3.21E-19 | 2.67E-18 |
| Novel00179 | - | PF10431:C-terminal, D2-small domain, of ClpB protein|PF02861:Clp amino terminal domain, pathogenicity island component|PF07724:AAA domain (Cdc48 subfamily)|PF17871:AAA lid domain|PF00004:ATPase family associated with various cellular activities (AAA) | 2.023014861 | 3.72E-19 | 3.09E-18 |
| FQU82_RS17920 | FQU82_RS17920 | acetyl-CoA hydrolase/transferase family protein | -1.426868226 | 4.46E-19 | 3.69E-18 |
| Novel00024 | - | PF01042:Endoribonuclease L-PSP|PF00842:Alanine racemase, C-terminal domain|PF01168:Alanine racemase, N-terminal domain|PF01266:FAD dependent oxidoreductase|PF00324:Amino acid permease | 2.117884679 | 5.24E-19 | 4.33E-18 |
| FQU82_RS15460 | trpS | tryptophan--tRNA ligase | -1.522873015 | 5.76E-19 | 4.75E-18 |
| FQU82_RS15280 | FQU82_RS15280 | co-chaperone GroES | -1.362740315 | 5.82E-19 | 4.79E-18 |
| FQU82_RS01725 | murD | UDP-N-acetylmuramoyl-L-alanine--D-glutamate ligase | -1.487454266 | 6.86E-19 | 5.62E-18 |
| FQU82_RS05025 | pgaB | poly-beta-1,6-N-acetyl-D-glucosamine N-deacetylase PgaB | -1.251791344 | 7.76E-19 | 6.35E-18 |
| FQU82_RS02980 | FQU82_RS02980 | alpha/beta hydrolase | 1.510078262 | 9.10E-19 | 7.43E-18 |
| FQU82_RS18415 | FQU82_RS18415 | TonB-dependent siderophore receptor | -1.579648794 | 9.64E-19 | 7.85E-18 |
| FQU82_RS04250 | fabD | ACP S-malonyltransferase | -1.153372232 | 9.98E-19 | 8.11E-18 |
| FQU82_RS11935 | accB | acetyl-CoA carboxylase biotin carboxyl carrier protein | -1.648705169 | 1.30E-18 | 1.05E-17 |
| FQU82_RS15645 | adeJ | multidrug efflux RND transporter permease subunit AdeJ | -1.401920111 | 1.35E-18 | 1.09E-17 |
| FQU82_RS04560 | minC | septum site-determining protein MinC | -1.630137168 | 1.46E-18 | 1.18E-17 |
| FQU82_RS12730 | FQU82_RS12730 | hypothetical protein | 1.494086422 | 1.53E-18 | 1.23E-17 |
| FQU82_RS13795 | rodA | rod shape-determining protein RodA | -2.633971096 | 1.55E-18 | 1.25E-17 |
| FQU82_RS09990 | FQU82_RS09990 | HIT family protein | 1.164940732 | 2.14E-18 | 1.71E-17 |
| FQU82_RS02035 | uvrC | excinuclease ABC subunit UvrC | 1.410855078 | 2.42E-18 | 1.93E-17 |
| FQU82_RS03170 | FQU82_RS03170 | peptide chain release factor 3 | -1.691334579 | 2.60E-18 | 2.08E-17 |
| FQU82_RS15305 | glpD | glycerol-3-phosphate dehydrogenase | 1.679555648 | 3.14E-18 | 2.50E-17 |
| FQU82_RS08930 | FQU82_RS08930 | pyridoxal phosphate-dependent aminotransferase | 1.128831486 | 3.28E-18 | 2.60E-17 |
| FQU82_RS12400 | FQU82_RS12400 | MFS transporter | -2.167510892 | 3.36E-18 | 2.66E-17 |
| FQU82_RS04170 | bfr | bacterioferritin | 1.0172174 | 4.84E-18 | 3.83E-17 |
| FQU82_RS04300 | pth | aminoacyl-tRNA hydrolase | -1.36346901 | 4.97E-18 | 3.92E-17 |
| FQU82_RS09765 | FQU82_RS09765 | phasin family protein | 2.007670163 | 5.09E-18 | 4.01E-17 |
| FQU82_RS12605 | cyoB | cytochrome o ubiquinol oxidase subunit I | -1.475435696 | 5.29E-18 | 4.15E-17 |
| FQU82_RS12575 | pgaB | poly-beta-1,6-N-acetyl-D-glucosamine N-deacetylase PgaB | 3.070143897 | 5.33E-18 | 4.17E-17 |
| FQU82_RS12595 | FQU82_RS12595 | RDD family protein | -1.454428519 | 5.37E-18 | 4.20E-17 |
| FQU82_RS14430 | hemL | glutamate-1-semialdehyde 2,1-aminomutase | -1.14325752 | 5.47E-18 | 4.27E-17 |
| FQU82_RS17765 | murF | UDP-N-acetylmuramoyl-tripeptide--D-alanyl-D-alanine ligase | -1.320872586 | 5.58E-18 | 4.34E-17 |
| FQU82_RS13910 | FQU82_RS13910 | NADP-dependent malic enzyme | -1.183902271 | 9.67E-18 | 7.51E-17 |
| FQU82_RS16425 | FQU82_RS16425 | RcnB family protein | 2.767484263 | 1.10E-17 | 8.50E-17 |
| FQU82_RS03055 | der | ribosome biogenesis GTPase Der | -1.142758816 | 1.10E-17 | 8.54E-17 |
| FQU82_RS11000 | FQU82_RS11000 | NADH:flavin oxidoreductase/NADH oxidase family protein | 2.353546797 | 1.16E-17 | 8.91E-17 |
| FQU82_RS11795 | FQU82_RS11795 | hypothetical protein | -2.69729327 | 1.18E-17 | 9.11E-17 |
| FQU82_RS11880 | FQU82_RS11880 | molybdopterin-dependent oxidoreductase | 2.160659094 | 1.21E-17 | 9.32E-17 |
| FQU82_RS06585 | FQU82_RS06585 | aspartate kinase | -1.208467626 | 1.60E-17 | 1.22E-16 |
| Novel00355 | - | PF00324:Amino acid permease | 2.618608172 | 1.66E-17 | 1.27E-16 |
| FQU82_RS06020 | FQU82_RS06020 | TonB-dependent receptor | -1.829495885 | 2.13E-17 | 1.63E-16 |
| FQU82_RS15850 | FQU82_RS15850 | hypothetical protein | 1.74776471 | 2.40E-17 | 1.82E-16 |
| FQU82_RS09425 | FQU82_RS09425 | LPS-assembly protein LptD | -1.205903021 | 2.52E-17 | 1.91E-16 |
| FQU82_RS14365 | FQU82_RS14365 | aldehyde dehydrogenase family protein | 1.247151405 | 2.70E-17 | 2.04E-16 |
| FQU82_RS09820 | FQU82_RS09820 | SidA/IucD/PvdA family monooxygenase | -3.061993411 | 2.96E-17 | 2.24E-16 |
| FQU82_RS09945 | FQU82_RS09945 | CerR family C-terminal domain-containing protein | 2.366698129 | 3.01E-17 | 2.27E-16 |
| FQU82_RS11310 | FQU82_RS11310 | CTP synthase | -1.256230269 | 3.34E-17 | 2.51E-16 |
| FQU82_RS01765 | FQU82_RS01765 | TetR/AcrR family transcriptional regulator | 3.689556156 | 3.64E-17 | 2.74E-16 |
| FQU82_RS18400 | guaB | IMP dehydrogenase | -1.215259901 | 4.09E-17 | 3.07E-16 |
| FQU82_RS14005 | trpD | anthranilate phosphoribosyltransferase | -1.255473874 | 4.42E-17 | 3.31E-16 |
| FQU82_RS10245 | FQU82_RS10245 | thiolase family protein | 1.286469046 | 5.61E-17 | 4.19E-16 |
| FQU82_RS11570 | FQU82_RS11570 | fructose-specific PTS transporter subunit EIIC | -1.351740065 | 6.65E-17 | 4.96E-16 |
| FQU82_RS09370 | FQU82_RS09370 | YoaK family protein | -1.918431212 | 7.84E-17 | 5.83E-16 |
| FQU82_RS12115 | FQU82_RS12115 | universal stress protein | 1.049928452 | 8.50E-17 | 6.31E-16 |
| FQU82_RS15790 | hchA | protein deglycase HchA | -1.284503278 | 8.62E-17 | 6.37E-16 |
| FQU82_RS03190 | acnA | aconitate hydratase AcnA | 1.218785073 | 8.63E-17 | 6.37E-16 |
| FQU82_RS14205 | FQU82_RS14205 | YbdD/YjiX family protein | 1.544352644 | 8.69E-17 | 6.41E-16 |
| FQU82_RS02765 | FQU82_RS02765 | dihydrofolate reductase | -1.299322887 | 9.69E-17 | 7.12E-16 |
| FQU82_RS15625 | FQU82_RS15625 | polyprenyl synthetase family protein | -1.51392856 | 1.02E-16 | 7.50E-16 |
| FQU82_RS13860 | FQU82_RS13860 | MFS transporter | 1.490163638 | 1.08E-16 | 7.90E-16 |
| FQU82_RS05680 | FQU82_RS05680 | hypothetical protein | 1.179461111 | 1.12E-16 | 8.16E-16 |
| FQU82_RS02155 | rimP | ribosome maturation factor RimP | -1.255950128 | 1.14E-16 | 8.31E-16 |
| FQU82_RS04500 | tuf | elongation factor Tu | -2.062200106 | 1.16E-16 | 8.44E-16 |
| FQU82_RS15005 | lolD | lipoprotein-releasing ABC transporter ATP-binding protein LolD | -1.482044438 | 1.17E-16 | 8.50E-16 |
| Novel00214 | - | PF00318:Ribosomal protein S2|PF00889:Elongation factor TS | -1.553753681 | 1.21E-16 | 8.76E-16 |
| FQU82_RS15405 | greA | transcription elongation factor GreA | -1.311947996 | 1.48E-16 | 1.07E-15 |
| FQU82_RS08540 | FQU82_RS08540 | hypothetical protein | 1.41789527 | 1.95E-16 | 1.41E-15 |
| FQU82_RS12170 | FQU82_RS12170 | mechanosensitive ion channel | -1.374101035 | 1.96E-16 | 1.41E-15 |
| FQU82_RS03135 | FQU82_RS03135 | acetolactate synthase 3 large subunit | -1.016614871 | 2.01E-16 | 1.45E-15 |
| FQU82_RS14300 | mnmA | tRNA 2-thiouridine(34) synthase MnmA | -1.188259256 | 2.02E-16 | 1.45E-15 |
| FQU82_RS14520 | FQU82_RS14520 | D-alanyl-D-alanine carboxypeptidase PBP6B | -1.353601477 | 2.28E-16 | 1.63E-15 |
| FQU82_RS12550 | FQU82_RS12550 | hypothetical protein | 1.996970832 | 2.82E-16 | 2.01E-15 |
| FQU82_RS03095 | FQU82_RS03095 | enoyl-ACP reductase | -1.314690001 | 3.89E-16 | 2.77E-15 |
| FQU82_RS15650 | adeK | multidrug efflux RND transporter outer membrane channel subunit AdeK | -1.228502524 | 4.09E-16 | 2.91E-15 |
| FQU82_RS07775 | FQU82_RS07775 | SRPBCC family protein | 1.578412907 | 4.27E-16 | 3.03E-15 |
| FQU82_RS11850 | FQU82_RS11850 | winged helix-turn-helix transcriptional regulator | 1.277311659 | 4.31E-16 | 3.05E-15 |
| FQU82_RS04995 | FQU82_RS04995 | acyl-CoA thioesterase | 1.266189629 | 4.50E-16 | 3.18E-15 |
| FQU82_RS04570 | FQU82_RS04570 | acyltransferase | -1.553790546 | 4.61E-16 | 3.25E-15 |
| Novel00058 | - | PF00141:Peroxidase | 1.991380272 | 4.68E-16 | 3.29E-15 |
| FQU82_RS12275 | FQU82_RS12275 | helix-turn-helix domain-containing protein | 1.51481501 | 4.88E-16 | 3.43E-15 |
| FQU82_RS15990 | FQU82_RS15990 | tRNA-Asp | -3.168279406 | 7.45E-16 | 5.23E-15 |
| FQU82_RS16995 | FQU82_RS16995 | YheV family putative zinc ribbon protein | 1.933195139 | 9.46E-16 | 6.62E-15 |
| FQU82_RS14855 | FQU82_RS14855 | enoyl-CoA hydratase-related protein | 0.93726053 | 9.50E-16 | 6.64E-15 |
| FQU82_RS14640 | FQU82_RS14640 | hypothetical protein | 1.290467776 | 1.12E-15 | 7.79E-15 |
| FQU82_RS01660 | gpmI | 2,3-bisphosphoglycerate-independent phosphoglycerate mutase | -1.219896258 | 1.18E-15 | 8.21E-15 |
| FQU82_RS12140 | FQU82_RS12140 | TonB-dependent siderophore receptor | -2.979828498 | 1.22E-15 | 8.48E-15 |
| FQU82_RS11595 | FQU82_RS11595 | universal stress protein | 1.483700952 | 1.35E-15 | 9.39E-15 |
| FQU82_RS04060 | metG | methionine--tRNA ligase | -1.064556069 | 1.39E-15 | 9.63E-15 |
| FQU82_RS07720 | FQU82_RS07720 | divalent metal cation transporter | -1.429495558 | 1.39E-15 | 9.63E-15 |
| FQU82_RS11365 | clpA | ATP-dependent Clp protease ATP-binding subunit ClpA | 1.387469475 | 1.48E-15 | 1.02E-14 |
| Novel00134 | - | PF20979:Arginosuccinate synthase C-terminal domain|PF00764:Arginosuccinate synthase N-terminal HUP domain | -1.959176141 | 1.58E-15 | 1.09E-14 |
| FQU82_RS07040 | ahpC | alkyl hydroperoxide reductase subunit C | 1.001425661 | 1.64E-15 | 1.13E-14 |
| FQU82_RS01065 | FQU82_RS01065 | RidA family protein | 1.365530527 | 1.68E-15 | 1.15E-14 |
| FQU82_RS00880 | FQU82_RS00880 | nucleotide sugar dehydrogenase | -1.231115333 | 1.73E-15 | 1.19E-14 |
| FQU82_RS08960 | glnD | [protein-PII] uridylyltransferase | 1.087949611 | 1.97E-15 | 1.34E-14 |
| FQU82_RS15750 | FQU82_RS15750 | patatin-like phospholipase family protein | -1.683510954 | 2.96E-15 | 2.02E-14 |
| FQU82_RS17760 | mraY | phospho-N-acetylmuramoyl-pentapeptide-transferase | -1.485247578 | 3.02E-15 | 2.05E-14 |
| FQU82_RS05210 | FQU82_RS05210 | hypothetical protein | 2.200465932 | 3.04E-15 | 2.06E-14 |
| FQU82_RS05380 | FQU82_RS05380 | copper resistance protein NlpE | -1.244036385 | 3.24E-15 | 2.20E-14 |
| FQU82_RS03410 | pheS | phenylalanine--tRNA ligase subunit alpha | -1.068112518 | 3.32E-15 | 2.24E-14 |
| FQU82_RS07605 | FQU82_RS07605 | universal stress protein | 0.945402622 | 3.34E-15 | 2.25E-14 |
| FQU82_RS09430 | FQU82_RS09430 | phosphotransferase | -1.384703754 | 3.51E-15 | 2.36E-14 |
| Novel00324 | - | PF02525:Flavodoxin-like fold | 9.386287216 | 3.66E-15 | 2.46E-14 |
| FQU82_RS03900 | bfmR | response regulator transcription factor BfmR | -1.221882614 | 4.11E-15 | 2.76E-14 |
| FQU82_RS17625 | gmk | guanylate kinase | -1.170645468 | 4.27E-15 | 2.85E-14 |
| FQU82_RS05990 | FQU82_RS05990 | LysM peptidoglycan-binding domain-containing protein | -0.920917113 | 4.67E-15 | 3.12E-14 |
| Novel00114 | - | PF00164:Ribosomal protein S12/S23|PF10118:Predicted metal-dependent hydrolase|PF00177:Ribosomal protein S7p/S5e|PF03143:Elongation factor Tu C-terminal domain|PF03144:Elongation factor Tu domain 2|PF00009:Elongation factor Tu GTP binding domain|PF03764:Elongation factor G, domain IV|PF00679:Elongation factor G C-terminus|PF14492:Elongation Factor G, domain III | -1.287885324 | 4.70E-15 | 3.14E-14 |
| FQU82_RS02965 | FQU82_RS02965 | gluconokinase | -1.323283324 | 5.00E-15 | 3.33E-14 |
| Novel00055 | - | PF08028:Acyl-CoA dehydrogenase, C-terminal domain | 2.590882485 | 5.10E-15 | 3.39E-14 |
| FQU82_RS02645 | FQU82_RS02645 | ATP-binding cassette domain-containing protein | -1.200983604 | 5.14E-15 | 3.40E-14 |
| FQU82_RS08630 | FQU82_RS08630 | family 2A encapsulin nanocompartment shell protein | 2.193032496 | 5.79E-15 | 3.83E-14 |
| FQU82_RS04455 | FQU82_RS04455 | NAD+ synthase | -1.04950796 | 7.11E-15 | 4.69E-14 |
| FQU82_RS09950 | FQU82_RS09950 | HlyD family efflux transporter periplasmic adaptor subunit | 2.445489962 | 7.17E-15 | 4.72E-14 |
| Novel00072 | - | PF05681:Fumarate hydratase (Fumerase)|PF05683:Fumarase C-terminus | -1.623344009 | 7.46E-15 | 4.91E-14 |
| FQU82_RS15285 | blp1 | biofilm-associated Ig-like repeat protein Blp1 | -1.103136601 | 8.17E-15 | 5.36E-14 |
| FQU82_RS09010 | FQU82_RS09010 | methionine ABC transporter permease | 2.598104739 | 8.55E-15 | 5.60E-14 |
| FQU82_RS02590 | FQU82_RS02590 | AraC family transcriptional regulator | 1.062796545 | 9.42E-15 | 6.16E-14 |
| FQU82_RS09955 | FQU82_RS09955 | ATP-binding cassette domain-containing protein | 2.213250899 | 1.07E-14 | 6.97E-14 |
| FQU82_RS18270 | uvrA | excinuclease ABC subunit UvrA | 1.03074034 | 1.09E-14 | 7.12E-14 |
| sRNA00046 | - | - | 3.21365113 | 1.12E-14 | 7.28E-14 |
| FQU82_RS05520 | FQU82_RS05520 | hypothetical protein | 3.402346503 | 1.28E-14 | 8.30E-14 |
| FQU82_RS01700 | FQU82_RS01700 | DsbC family protein | -1.269502216 | 1.48E-14 | 9.58E-14 |
| FQU82_RS12265 | FQU82_RS12265 | iron-containing alcohol dehydrogenase | -2.206419481 | 1.53E-14 | 9.92E-14 |
| FQU82_RS16560 | secF | protein translocase subunit SecF | -1.401475672 | 1.61E-14 | 1.04E-13 |
| FQU82_RS02190 | FQU82_RS02190 | monovalent cation/H+ antiporter subunit D | -1.132103466 | 1.72E-14 | 1.11E-13 |
| FQU82_RS09085 | FQU82_RS09085 | 1-acyl-sn-glycerol-3-phosphate acyltransferase | -3.078086453 | 1.87E-14 | 1.20E-13 |
| FQU82_RS04120 | FQU82_RS04120 | tRNA-dihydrouridine synthase | -2.29466302 | 1.93E-14 | 1.24E-13 |
| FQU82_RS01490 | FQU82_RS01490 | DUF2726 domain-containing protein | 1.517976323 | 1.97E-14 | 1.26E-13 |
| FQU82_RS15940 | FQU82_RS15940 | hypothetical protein | -2.154358175 | 1.99E-14 | 1.27E-13 |
| FQU82_RS01375 | FQU82_RS01375 | DMT family transporter | 1.400221921 | 2.37E-14 | 1.51E-13 |
| FQU82_RS11045 | FQU82_RS11045 | LysR family transcriptional regulator | 1.612498143 | 2.43E-14 | 1.55E-13 |
| FQU82_RS09660 | FQU82_RS09660 | SOS response-associated peptidase family protein | 1.945691386 | 2.65E-14 | 1.68E-13 |
| FQU82_RS14900 | tolQ | protein TolQ | -1.649277063 | 2.65E-14 | 1.69E-13 |
| FQU82_RS08360 | FQU82_RS08360 | SDR family oxidoreductase | -2.135682246 | 2.80E-14 | 1.78E-13 |
| FQU82_RS08710 | mdcA | malonate decarboxylase subunit alpha | 4.08050851 | 2.98E-14 | 1.89E-13 |
| Novel00143 | - | PF00578:AhpC/TSA family | 1.881810601 | 3.10E-14 | 1.96E-13 |
| FQU82_RS01265 | argS | arginine--tRNA ligase | -1.001841346 | 3.28E-14 | 2.07E-13 |
| FQU82_RS14915 | tolB | Tol-Pal system beta propeller repeat protein TolB | -1.185101815 | 3.66E-14 | 2.30E-13 |
| FQU82_RS17415 | astE | succinylglutamate desuccinylase | -1.170271251 | 3.79E-14 | 2.38E-13 |
| FQU82_RS15635 | FQU82_RS15635 | phosphatase PAP2 family protein | -0.950965121 | 4.79E-14 | 3.00E-13 |
| FQU82_RS03040 | hisS | histidine--tRNA ligase | -1.079429464 | 4.92E-14 | 3.08E-13 |
| FQU82_RS03265 | FQU82_RS03265 | TIM barrel protein | 1.161142805 | 5.40E-14 | 3.37E-13 |
| FQU82_RS07985 | FQU82_RS07985 | I78 family peptidase inhibitor | -1.25773615 | 5.60E-14 | 3.49E-13 |
| FQU82_RS10305 | FQU82_RS10305 | AraC family transcriptional regulator | 1.843002363 | 5.63E-14 | 3.50E-13 |
| FQU82_RS14950 | FQU82_RS14950 | DUF3106 domain-containing protein | 1.815216603 | 5.76E-14 | 3.58E-13 |
| FQU82_RS15855 | rng | ribonuclease G | -1.051652011 | 6.04E-14 | 3.75E-13 |
| FQU82_RS11565 | pfkB | 1-phosphofructokinase | -1.317851667 | 8.17E-14 | 5.06E-13 |
| FQU82_RS12525 | prmC | peptide chain release factor N(5)-glutamine methyltransferase | -2.089998654 | 8.18E-14 | 5.06E-13 |
| FQU82_RS01925 | rpoB | DNA-directed RNA polymerase subunit beta | -1.279785613 | 8.50E-14 | 5.24E-13 |
| FQU82_RS15075 | gyrA | DNA gyrase subunit A | -1.11333453 | 1.01E-13 | 6.25E-13 |
| FQU82_RS14445 | FQU82_RS14445 | CYTH and CHAD domain-containing protein | 1.207782619 | 1.04E-13 | 6.41E-13 |
| FQU82_RS09225 | FQU82_RS09225 | hypothetical protein | 1.817371774 | 1.39E-13 | 8.55E-13 |
| FQU82_RS11560 | ptsP | phosphoenolpyruvate--protein phosphotransferase | -1.036820387 | 1.58E-13 | 9.65E-13 |
| FQU82_RS04700 | FQU82_RS04700 | DHA2 family efflux MFS transporter permease subunit | 1.193748537 | 1.86E-13 | 1.14E-12 |
| FQU82_RS09365 | FQU82_RS09365 | DUF2058 domain-containing protein | -2.09390463 | 1.90E-13 | 1.16E-12 |
| FQU82_RS03220 | dusA | tRNA dihydrouridine(20/20a) synthase DusA | -1.527838976 | 1.98E-13 | 1.21E-12 |
| FQU82_RS09540 | FQU82_RS09540 | SRPBCC family protein | -2.230849195 | 2.29E-13 | 1.39E-12 |
| FQU82_RS02850 | FQU82_RS02850 | DUF839 domain-containing protein | -2.494856332 | 2.37E-13 | 1.44E-12 |
| FQU82_RS00795 | FQU82_RS00795 | FKBP-type peptidyl-prolyl cis-trans isomerase | -1.082395392 | 2.59E-13 | 1.57E-12 |
| FQU82_RS00270 | FQU82_RS00270 | TerC family protein | 1.822166235 | 2.60E-13 | 1.57E-12 |
| FQU82_RS00870 | FQU82_RS00870 | sugar transferase | -1.333797387 | 2.95E-13 | 1.78E-12 |
| Novel00274 | - | PF10417:C-terminal domain of 1-Cys peroxiredoxin|PF00753:Metallo-beta-lactamase superfamily | 2.114471717 | 3.58E-13 | 2.16E-12 |
| FQU82_RS01400 | FQU82_RS01400 | DUF2946 family protein | -1.128188486 | 3.92E-13 | 2.36E-12 |
| FQU82_RS08355 | FQU82_RS08355 | 3-hydroxyacyl-CoA dehydrogenase | -1.775770075 | 5.06E-13 | 3.04E-12 |
| FQU82_RS01870 | FQU82_RS01870 | tRNA-Tyr | -1.291157311 | 5.10E-13 | 3.06E-12 |
| FQU82_RS03690 | FQU82_RS03690 | cation transporter | 2.110078954 | 5.32E-13 | 3.19E-12 |
| FQU82_RS14045 | FQU82_RS14045 | DUF4105 domain-containing protein | -1.658369434 | 5.35E-13 | 3.20E-12 |
| FQU82_RS04215 | rluB | 23S rRNA pseudouridine(2605) synthase RluB | -0.947316485 | 5.39E-13 | 3.22E-12 |
| Novel00047 | - | - | 5.292529519 | 5.57E-13 | 3.32E-12 |
| FQU82_RS14705 | pdxJ | pyridoxine 5'-phosphate synthase | -1.289898947 | 6.17E-13 | 3.67E-12 |
| FQU82_RS06575 | rnhB | ribonuclease HII | -1.586884176 | 7.30E-13 | 4.34E-12 |
| FQU82_RS00515 | rph | ribonuclease PH | -1.123533793 | 7.44E-13 | 4.41E-12 |
| FQU82_RS01885 | tuf | elongation factor Tu | -3.444624414 | 7.46E-13 | 4.42E-12 |
| FQU82_RS03750 | FQU82_RS03750 | acyl-CoA dehydrogenase | 1.310950184 | 7.81E-13 | 4.61E-12 |
| FQU82_RS09815 | FQU82_RS09815 | IucA/IucC family protein | -2.147365072 | 8.29E-13 | 4.89E-12 |
| Novel00362 | - | PF00873:AcrB/AcrD/AcrF family|PF16576:Barrel-sandwich domain of CusB or HlyD membrane-fusion | 2.338821269 | 8.43E-13 | 4.96E-12 |
| FQU82_RS02660 | FQU82_RS02660 | winged helix-turn-helix transcriptional regulator | 1.732105079 | 1.00E-12 | 5.90E-12 |
| FQU82_RS09080 | FQU82_RS09080 | YebC/PmpR family DNA-binding transcriptional regulator | -1.349282156 | 1.01E-12 | 5.90E-12 |
| FQU82_RS15795 | FQU82_RS15795 | NAD(P)H-quinone oxidoreductase | -1.10768191 | 1.16E-12 | 6.79E-12 |
| FQU82_RS09315 | trmA | tRNA (uridine(54)-C5)-methyltransferase TrmA | -2.023441888 | 1.32E-12 | 7.70E-12 |
| FQU82_RS11685 | FQU82_RS11685 | YbgF trimerization domain-containing protein | -1.70055393 | 1.39E-12 | 8.10E-12 |
| FQU82_RS17355 | FQU82_RS17355 | putative DNA modification/repair radical SAM protein | 1.764906523 | 1.61E-12 | 9.37E-12 |
| FQU82_RS09180 | FQU82_RS09180 | hypothetical protein | 1.834365134 | 1.70E-12 | 9.92E-12 |
| FQU82_RS15375 | rlmE | 23S rRNA (uridine(2552)-2'-O)-methyltransferase RlmE | 0.862052058 | 1.81E-12 | 1.05E-11 |
| FQU82_RS07870 | tssE | type VI secretion system baseplate subunit TssE | 2.467429054 | 2.10E-12 | 1.22E-11 |
| FQU82_RS08350 | FQU82_RS08350 | 3-oxoadipyl-CoA thiolase | -1.885507647 | 2.29E-12 | 1.33E-11 |
| FQU82_RS15085 | FQU82_RS15085 | electron transfer flavoprotein subunit beta/FixA family protein | -1.088254936 | 2.35E-12 | 1.36E-11 |
| Novel00344 | - | - | 1.343781398 | 2.48E-12 | 1.43E-11 |
| FQU82_RS16105 | FQU82_RS16105 | DsbC family protein | -1.164662376 | 2.63E-12 | 1.52E-11 |
| FQU82_RS03570 | murA | UDP-N-acetylglucosamine 1-carboxyvinyltransferase | -1.216131653 | 2.69E-12 | 1.55E-11 |
| FQU82_RS13315 | FQU82_RS13315 | ferredoxin--NADP reductase | -1.079225595 | 2.75E-12 | 1.58E-11 |
| FQU82_RS04505 | FQU82_RS04505 | metal-dependent hydrolase | 1.896965256 | 2.93E-12 | 1.68E-11 |
| FQU82_RS18735 | ribBA | bifunctional 3,4-dihydroxy-2-butanone-4-phosphate synthase/GTP cyclohydrolase II | -0.912946534 | 3.37E-12 | 1.93E-11 |
| FQU82_RS15520 | gltA | citrate synthase | -0.974269728 | 3.52E-12 | 2.01E-11 |
| FQU82_RS17445 | FQU82_RS17445 | amino acid permease | -1.001199181 | 3.70E-12 | 2.11E-11 |
| FQU82_RS08640 | FQU82_RS08640 | rhodanese-like domain-containing protein | 2.265918316 | 4.15E-12 | 2.37E-11 |
| FQU82_RS12570 | pgaC | poly-beta-1,6-N-acetyl-D-glucosamine synthase | 2.378967032 | 4.76E-12 | 2.71E-11 |
| FQU82_RS13110 | FQU82_RS13110 | M48 family metalloprotease | 1.388172886 | 4.78E-12 | 2.71E-11 |
| FQU82_RS01970 | htpG | molecular chaperone HtpG | 1.124344851 | 4.78E-12 | 2.71E-11 |
| FQU82_RS02970 | FQU82_RS02970 | NADP-dependent glyceraldehyde-3-phosphate dehydrogenase | -0.964763961 | 4.81E-12 | 2.72E-11 |
| FQU82_RS17835 | FQU82_RS17835 | hypothetical protein | 1.334585305 | 4.89E-12 | 2.76E-11 |
| FQU82_RS15465 | sucD | succinate--CoA ligase subunit alpha | -1.300974128 | 5.02E-12 | 2.83E-11 |
| FQU82_RS17340 | FQU82_RS17340 | PspC domain-containing protein | 1.685352256 | 5.93E-12 | 3.34E-11 |
| FQU82_RS05965 | FQU82_RS05965 | BON domain-containing protein | -1.09719404 | 6.01E-12 | 3.38E-11 |
| FQU82_RS00465 | FQU82_RS00465 | RcnB family protein | 2.835349013 | 6.68E-12 | 3.75E-11 |
| FQU82_RS18720 | FQU82_RS18720 | hypothetical protein | 1.009023641 | 6.76E-12 | 3.79E-11 |
| FQU82_RS11695 | fabZ | 3-hydroxyacyl-ACP dehydratase FabZ | -1.128974973 | 6.97E-12 | 3.90E-11 |
| FQU82_RS04960 | mqo | malate dehydrogenase (quinone) | -1.019341871 | 8.18E-12 | 4.57E-11 |
| FQU82_RS12300 | FQU82_RS12300 | glutamine amidotransferase | -2.104183478 | 8.37E-12 | 4.67E-11 |
| FQU82_RS17295 | FQU82_RS17295 | DEAD/DEAH box helicase | -1.193098665 | 8.44E-12 | 4.70E-11 |
| FQU82_RS11720 | ispC | 1-deoxy-D-xylulose-5-phosphate reductoisomerase | -1.052193849 | 8.53E-12 | 4.74E-11 |
| FQU82_RS11355 | FQU82_RS11355 | 3-deoxy-7-phosphoheptulonate synthase | 0.866258064 | 9.22E-12 | 5.12E-11 |
| FQU82_RS02680 | FQU82_RS02680 | HdeD family acid-resistance protein | 1.287204145 | 9.71E-12 | 5.38E-11 |
| FQU82_RS12040 | FQU82_RS12040 | MaoC/PaaZ C-terminal domain-containing protein | -1.205049192 | 9.76E-12 | 5.40E-11 |
| Novel00289 | - | - | 2.381469275 | 9.81E-12 | 5.42E-11 |
| FQU82_RS07090 | FQU82_RS07090 | LysR family transcriptional regulator | 1.489983396 | 1.01E-11 | 5.56E-11 |
| FQU82_RS01900 | nusG | transcription termination/antitermination protein NusG | -1.255013276 | 1.09E-11 | 6.01E-11 |
| FQU82_RS15530 | FQU82_RS15530 | rhodanese-related sulfurtransferase | -1.324009412 | 1.16E-11 | 6.37E-11 |
| FQU82_RS01530 | FQU82_RS01530 | trimeric intracellular cation channel family protein | -2.033514269 | 1.18E-11 | 6.48E-11 |
| FQU82_RS10260 | FQU82_RS10260 | CoA transferase subunit A | 1.414869625 | 1.24E-11 | 6.81E-11 |
| FQU82_RS08920 | FQU82_RS08920 | type II asparaginase | -1.06489946 | 1.30E-11 | 7.10E-11 |
| FQU82_RS16395 | FQU82_RS16395 | hypothetical protein | 2.042248945 | 1.36E-11 | 7.43E-11 |
| FQU82_RS05880 | FQU82_RS05880 | hypothetical protein | 1.559734848 | 1.41E-11 | 7.71E-11 |
| FQU82_RS16175 | FQU82_RS16175 | RtcB family protein | -1.91174012 | 1.48E-11 | 8.07E-11 |
| FQU82_RS16565 | coaBC | bifunctional phosphopantothenoylcysteine decarboxylase/phosphopantothenate--cysteine ligase CoaBC | -1.024790604 | 1.52E-11 | 8.24E-11 |
| FQU82_RS03390 | rplT | 50S ribosomal protein L20 | -1.032877763 | 1.54E-11 | 8.39E-11 |
| FQU82_RS04110 | FQU82_RS04110 | hypothetical protein | 1.266513905 | 1.61E-11 | 8.73E-11 |
| FQU82_RS02205 | FQU82_RS02205 | AAA family ATPase | 0.946410171 | 1.63E-11 | 8.82E-11 |
| FQU82_RS13900 | FQU82_RS13900 | 16S rRNA (uracil(1498)-N(3))-methyltransferase | -2.023186642 | 1.80E-11 | 9.75E-11 |
| FQU82_RS05595 | FQU82_RS05595 | LexA family transcriptional regulator | 1.118102874 | 1.97E-11 | 1.06E-10 |
| FQU82_RS13090 | FQU82_RS13090 | GatB/YqeY domain-containing protein | -0.818747312 | 2.04E-11 | 1.10E-10 |
| FQU82_RS13225 | FQU82_RS13225 | bifunctional prephenate dehydrogenase/3-phosphoshikimate 1-carboxyvinyltransferase | -0.846640443 | 2.08E-11 | 1.12E-10 |
| Novel00198 | - | PF00296:Luciferase-like monooxygenase | 1.749712704 | 2.19E-11 | 1.18E-10 |
| FQU82_RS09020 | FQU82_RS09020 | MetQ/NlpA family ABC transporter substrate-binding protein | 3.139228571 | 2.30E-11 | 1.23E-10 |
| FQU82_RS10635 | FQU82_RS10635 | hypothetical protein | -2.656776623 | 2.44E-11 | 1.30E-10 |
| FQU82_RS01790 | argH | argininosuccinate lyase | -1.048027595 | 2.44E-11 | 1.30E-10 |
| FQU82_RS12980 | FQU82_RS12980 | hypothetical protein | -2.269116565 | 2.79E-11 | 1.49E-10 |
| FQU82_RS17660 | FQU82_RS17660 | O-antigen ligase family protein | -1.555502282 | 2.80E-11 | 1.49E-10 |
| FQU82_RS04985 | FQU82_RS04985 | choline transporter | -1.323059824 | 2.89E-11 | 1.54E-10 |
| FQU82_RS13145 | FQU82_RS13145 | NAD(P)H-dependent glycerol-3-phosphate dehydrogenase | -1.066997655 | 3.01E-11 | 1.60E-10 |
| FQU82_RS08925 | FQU82_RS08925 | dicarboxylate/amino acid:cation symporter | -1.558472877 | 3.02E-11 | 1.60E-10 |
| FQU82_RS17990 | FQU82_RS17990 | homoserine kinase | -1.381682699 | 3.27E-11 | 1.73E-10 |
| FQU82_RS15880 | gatC | Asp-tRNA(Asn)/Glu-tRNA(Gln) amidotransferase subunit GatC | -1.426282035 | 3.29E-11 | 1.74E-10 |
| FQU82_RS07510 | cysS | cysteine--tRNA ligase | -0.989306735 | 4.00E-11 | 2.11E-10 |
| FQU82_RS13065 | FQU82_RS13065 | DUF3336 domain-containing protein | 1.129171595 | 4.11E-11 | 2.17E-10 |
| FQU82_RS01840 | FQU82_RS01840 | type II secretion system protein N | -1.228980288 | 4.30E-11 | 2.26E-10 |
| FQU82_RS00785 | murJ | murein biosynthesis integral membrane protein MurJ | -1.073028101 | 4.35E-11 | 2.28E-10 |
| FQU82_RS11865 | FQU82_RS11865 | molybdenum cofactor biosynthesis protein MoaE | 1.808020377 | 4.35E-11 | 2.28E-10 |
| FQU82_RS03815 | FQU82_RS03815 | DUF1852 domain-containing protein | -1.160588871 | 5.30E-11 | 2.78E-10 |
| FQU82_RS03150 | FQU82_RS03150 | GGDEF domain-containing phosphodiesterase | 1.217969138 | 5.73E-11 | 3.00E-10 |
| FQU82_RS15170 | FQU82_RS15170 | TetR/AcrR family transcriptional regulator | 1.569059314 | 5.82E-11 | 3.04E-10 |
| FQU82_RS17035 | FQU82_RS17035 | NAD(P)/FAD-dependent oxidoreductase | 0.924010654 | 5.89E-11 | 3.07E-10 |
| FQU82_RS05450 | adk | adenylate kinase | -0.911463258 | 6.39E-11 | 3.33E-10 |
| FQU82_RS18375 | prfB | peptide chain release factor 2 | -1.012067017 | 6.78E-11 | 3.53E-10 |
| FQU82_RS03920 | ndhC | NADH-quinone oxidoreductase subunit A | -0.834473009 | 8.59E-11 | 4.46E-10 |
| FQU82_RS15865 | mreD | rod shape-determining protein MreD | -1.420107363 | 8.82E-11 | 4.58E-10 |
| FQU82_RS05970 | FQU82_RS05970 | alpha/beta hydrolase | -1.485472409 | 9.23E-11 | 4.78E-10 |
| FQU82_RS04685 | hemB | porphobilinogen synthase | 0.892616717 | 9.61E-11 | 4.97E-10 |
| FQU82_RS05005 | FQU82_RS05005 | GTP-binding protein | 1.621186248 | 1.00E-10 | 5.18E-10 |
| FQU82_RS18505 | FQU82_RS18505 | exodeoxyribonuclease III | -1.075690473 | 1.06E-10 | 5.48E-10 |
| FQU82_RS10415 | pqqC | pyrroloquinoline-quinone synthase PqqC | -1.175247752 | 1.20E-10 | 6.20E-10 |
| FQU82_RS13120 | FQU82_RS13120 | CvpA family protein | 1.675332827 | 1.25E-10 | 6.42E-10 |
| FQU82_RS12610 | cyoC | cytochrome o ubiquinol oxidase subunit III | -1.16208676 | 1.33E-10 | 6.84E-10 |
| FQU82_RS05580 | FQU82_RS05580 | hypothetical protein | 1.164380842 | 1.56E-10 | 8.01E-10 |
| FQU82_RS12735 | FQU82_RS12735 | dicarboxylate/amino acid:cation symporter | -1.139329157 | 1.74E-10 | 8.90E-10 |
| Novel00212 | - | - | 3.079682229 | 1.76E-10 | 8.98E-10 |
| FQU82_RS15515 | sdhC | succinate dehydrogenase, cytochrome b556 subunit | -0.815464356 | 1.85E-10 | 9.42E-10 |
| FQU82_RS02185 | FQU82_RS02185 | Na+/H+ antiporter subunit E | -1.443540087 | 2.08E-10 | 1.06E-09 |
| FQU82_RS08375 | FQU82_RS08375 | MFS transporter | -1.89435453 | 2.11E-10 | 1.08E-09 |
| FQU82_RS08310 | FQU82_RS08310 | MFS transporter | -2.203481187 | 2.25E-10 | 1.14E-09 |
| FQU82_RS18360 | FQU82_RS18360 | 3-deoxy-D-manno-octulosonic acid transferase | -1.230583742 | 2.44E-10 | 1.24E-09 |
| FQU82_RS14730 | FQU82_RS14730 | DUF4845 domain-containing protein | -1.650818131 | 2.50E-10 | 1.26E-09 |
| FQU82_RS18035 | FQU82_RS18035 | potassium transporter Kup | -1.057939944 | 2.80E-10 | 1.42E-09 |
| FQU82_RS09910 | FQU82_RS09910 | 3-deoxy-7-phosphoheptulonate synthase | -0.912723034 | 2.82E-10 | 1.42E-09 |
| Novel00200 | - | PF00510:Cytochrome c oxidase subunit III|PF01281:Ribosomal protein L9, N-terminal domain|PF03948:Ribosomal protein L9, C-terminal domain|PF00772:DnaB-like helicase N terminal domain|PF01040:UbiA prenyltransferase family|PF00115:Cytochrome C and Quinol oxidase polypeptide I|PF03626:Prokaryotic Cytochrome C oxidase subunit IV|PF01250:Ribosomal protein S6 | -1.138833421 | 2.85E-10 | 1.44E-09 |
| FQU82_RS13305 | FQU82_RS13305 | DUF4010 domain-containing protein | 1.569555348 | 3.01E-10 | 1.52E-09 |
| FQU82_RS03420 | FQU82_RS03420 | integration host factor subunit alpha | 0.977999898 | 3.09E-10 | 1.56E-09 |
| FQU82_RS10185 | FQU82_RS10185 | ABC transporter substrate-binding protein | 2.135993063 | 3.11E-10 | 1.56E-09 |
| FQU82_RS02740 | FQU82_RS02740 | energy transducer TonB | -1.107365584 | 3.13E-10 | 1.57E-09 |
| FQU82_RS17940 | FQU82_RS17940 | GNAT family N-acetyltransferase | 0.951461088 | 3.31E-10 | 1.66E-09 |
| FQU82_RS17550 | truB | tRNA pseudouridine(55) synthase TruB | -1.761980102 | 3.38E-10 | 1.69E-09 |
| FQU82_RS15830 | FQU82_RS15830 | outer membrane protein transport protein | 0.842945739 | 3.43E-10 | 1.71E-09 |
| FQU82_RS01845 | gspD | type II secretion system secretin GspD | -1.032240473 | 3.66E-10 | 1.83E-09 |
| FQU82_RS09985 | FQU82_RS09985 | porin | -2.481835029 | 3.67E-10 | 1.83E-09 |
| FQU82_RS07630 | mutS | DNA mismatch repair protein MutS | -0.9809497 | 3.68E-10 | 1.83E-09 |
| FQU82_RS02140 | secG | preprotein translocase subunit SecG | -1.055109669 | 3.97E-10 | 1.97E-09 |
| FQU82_RS13240 | FQU82_RS13240 | acyl-CoA dehydrogenase family protein | -1.179533605 | 4.04E-10 | 2.00E-09 |
| Novel00307 | - | PF01245:Ribosomal protein L19|PF01746:tRNA (Guanine-1)-methyltransferase|PF01782:RimM N-terminal domain|PF00886:Ribosomal protein S16 | -1.195551196 | 4.13E-10 | 2.05E-09 |
| Novel00172 | - | PF02082:Iron-dependent Transcriptional regulator|PF12706:Beta-lactamase superfamily domain | 3.355848828 | 4.39E-10 | 2.17E-09 |
| FQU82_RS15230 | FQU82_RS15230 | SCP2 sterol-binding domain-containing protein | -1.002628244 | 4.63E-10 | 2.29E-09 |
| FQU82_RS08645 | FQU82_RS08645 | SDR family oxidoreductase | 2.590520353 | 4.68E-10 | 2.31E-09 |
| FQU82_RS03005 | FQU82_RS03005 | phosphatase PAP2 family protein | 1.217224393 | 4.81E-10 | 2.37E-09 |
| FQU82_RS10960 | FQU82_RS10960 | TetR/AcrR family transcriptional regulator | 2.120406406 | 4.96E-10 | 2.44E-09 |
| Novel00188 | - | PF01266:FAD dependent oxidoreductase | 2.48805097 | 5.13E-10 | 2.52E-09 |
| FQU82_RS07970 | FQU82_RS07970 | epoxyqueuosine reductase QueH | -1.176280987 | 5.24E-10 | 2.57E-09 |
| FQU82_RS02170 | FQU82_RS02170 | ribosome-binding factor A | -1.257145016 | 5.24E-10 | 2.57E-09 |
| FQU82_RS05070 | FQU82_RS05070 | aromatic ring-hydroxylating dioxygenase subunit alpha | 1.817404944 | 5.83E-10 | 2.85E-09 |
| FQU82_RS05235 | FQU82_RS05235 | TonB-dependent siderophore receptor | -1.442643086 | 6.39E-10 | 3.12E-09 |
| FQU82_RS13135 | gspL | type II secretion system protein GspL | -1.126830805 | 6.43E-10 | 3.13E-09 |
| FQU82_RS09550 | rpsA | 30S ribosomal protein S1 | -1.327005647 | 6.48E-10 | 3.16E-09 |
| FQU82_RS02750 | FQU82_RS02750 | biopolymer transporter ExbD | -0.938572399 | 6.62E-10 | 3.22E-09 |
| FQU82_RS11510 | FQU82_RS11510 | aspartate aminotransferase family protein | -1.07701468 | 7.17E-10 | 3.48E-09 |
| FQU82_RS13810 | FQU82_RS13810 | DUF962 domain-containing protein | 1.808733403 | 7.33E-10 | 3.55E-09 |
| FQU82_RS09420 | FQU82_RS09420 | peptidylprolyl isomerase | -0.868286394 | 7.44E-10 | 3.60E-09 |
| FQU82_RS16840 | FQU82_RS16840 | hypothetical protein | 1.825929301 | 7.46E-10 | 3.61E-09 |
| FQU82_RS09265 | FQU82_RS09265 | FxsA family protein | 0.881815885 | 7.75E-10 | 3.74E-09 |
| FQU82_RS18435 | aceE | pyruvate dehydrogenase (acetyl-transferring), homodimeric type | -0.902830158 | 8.23E-10 | 3.97E-09 |
| Novel00301 | - | PF02812:Glu/Leu/Phe/Val dehydrogenase, dimerisation domain|PF00208:Glutamate/Leucine/Phenylalanine/Valine dehydrogenase | -1.428116933 | 8.30E-10 | 3.99E-09 |
| FQU82_RS11430 | FQU82_RS11430 | adenosine kinase | -0.889459043 | 8.72E-10 | 4.20E-09 |
| FQU82_RS09995 | FQU82_RS09995 | YARHG domain-containing protein | 0.912409907 | 9.54E-10 | 4.58E-09 |
| FQU82_RS08730 | mdcE | biotin-independent malonate decarboxylase subunit gamma | 2.538746977 | 9.96E-10 | 4.78E-09 |
| Novel00287 | - | PF09526:Probable metal-binding protein (DUF2387)|PF01432:Peptidase family M3 | 1.726031419 | 9.98E-10 | 4.78E-09 |
| FQU82_RS08100 | paaF | phenylacetate--CoA ligase | 1.451511598 | 1.05E-09 | 5.03E-09 |
| FQU82_RS01245 | FQU82_RS01245 | pirin family protein | 0.914900426 | 1.06E-09 | 5.06E-09 |
| FQU82_RS08940 | FQU82_RS08940 | glutathione peroxidase | 1.039008913 | 1.13E-09 | 5.40E-09 |
| FQU82_RS10410 | pqqB | pyrroloquinoline quinone biosynthesis protein PqqB | -1.315361694 | 1.33E-09 | 6.32E-09 |
| Novel00262 | - | PF01016:Ribosomal L27 protein|PF00829:Ribosomal prokaryotic L21 protein | -1.033713772 | 1.43E-09 | 6.80E-09 |
| FQU82_RS03720 | FQU82_RS03720 | tRNA-Arg | -1.935014885 | 1.43E-09 | 6.80E-09 |
| FQU82_RS03465 | FQU82_RS03465 | YggT family protein | -1.026420205 | 1.56E-09 | 7.39E-09 |
| Novel00277 | - | - | 2.331314194 | 1.73E-09 | 8.19E-09 |
| FQU82_RS12535 | FQU82_RS12535 | type 1 glutamine amidotransferase domain-containing protein | 2.497931531 | 1.83E-09 | 8.63E-09 |
| FQU82_RS14065 | FQU82_RS14065 | L,D-transpeptidase | -0.773919249 | 1.85E-09 | 8.74E-09 |
| FQU82_RS00200 | rpmH | 50S ribosomal protein L34 | -1.747572333 | 1.89E-09 | 8.90E-09 |
| FQU82_RS13790 | FQU82_RS13790 | hypothetical protein | -0.994459403 | 2.01E-09 | 9.48E-09 |
| Novel00311 | - | PF01493:GXGXG motif|PF01645:Conserved region in glutamate synthase|PF00310:Glutamine amidotransferases class-II|PF04898:Glutamate synthase central domain|PF07992:Pyridine nucleotide-disulphide oxidoreductase|PF14691:Dihydroprymidine dehydrogenase domain II, 4Fe-4S cluster | 0.916851373 | 2.02E-09 | 9.50E-09 |
| FQU82_RS03595 | FQU82_RS03595 | hypothetical protein | 2.315531791 | 2.04E-09 | 9.57E-09 |
| FQU82_RS05940 | FQU82_RS05940 | LysE/ArgO family amino acid transporter | 1.577579914 | 2.22E-09 | 1.04E-08 |
| FQU82_RS10215 | FQU82_RS10215 | TonB-dependent siderophore receptor | -1.875365565 | 2.22E-09 | 1.04E-08 |
| FQU82_RS14700 | FQU82_RS14700 | tRNA-(ms[2]io[6]A)-hydroxylase | -1.105978326 | 2.55E-09 | 1.19E-08 |
| FQU82_RS09530 | FQU82_RS09530 | enoyl-CoA hydratase/isomerase family protein | -0.853393955 | 2.55E-09 | 1.19E-08 |
| FQU82_RS10970 | FQU82_RS10970 | Rrf2 family transcriptional regulato | 1.208773172 | 2.60E-09 | 1.21E-08 |
| FQU82_RS14270 | surE | 5'/3'-nucleotidase SurE | -1.437667622 | 2.75E-09 | 1.28E-08 |
| FQU82_RS12895 | proP | glycine betaine/L-proline transporter ProP | -1.649133597 | 2.77E-09 | 1.29E-08 |
| FQU82_RS11875 | moaA | GTP 3',8-cyclase MoaA | 1.243787603 | 2.90E-09 | 1.35E-08 |
| FQU82_RS13255 | FQU82_RS13255 | YeaC family protein | 1.379816382 | 3.29E-09 | 1.53E-08 |
| FQU82_RS03075 | FQU82_RS03075 | rhodanese-like domain-containing protein | 0.814588312 | 3.45E-09 | 1.60E-08 |
| FQU82_RS18350 | FQU82_RS18350 | mechanosensitive ion channel family protein | -1.004670847 | 3.45E-09 | 1.60E-08 |
| FQU82_RS12615 | FQU82_RS12615 | cytochrome o ubiquinol oxidase subunit IV | -0.912404444 | 3.59E-09 | 1.66E-08 |
| FQU82_RS16685 | FQU82_RS16685 | OmpA family protein | -1.122825072 | 3.59E-09 | 1.66E-08 |
| FQU82_RS14505 | FQU82_RS14505 | rRNA large subunit pseudouridine synthase E | -0.929435033 | 3.61E-09 | 1.66E-08 |
| FQU82_RS01730 | ftsW | putative lipid II flippase FtsW | -0.937556851 | 3.81E-09 | 1.75E-08 |
| FQU82_RS10475 | FQU82_RS10475 | S-(hydroxymethyl)glutathione dehydrogenase/class III alcohol dehydrogenase | -0.827906019 | 4.08E-09 | 1.88E-08 |
| FQU82_RS15030 | serC | 3-phosphoserine/phosphohydroxythreonine transaminase | -0.913972087 | 4.10E-09 | 1.88E-08 |
| FQU82_RS15505 | sdhA | succinate dehydrogenase flavoprotein subunit | -0.881439647 | 4.30E-09 | 1.97E-08 |
| FQU82_RS17545 | FQU82_RS17545 | TSUP family transporter | -1.437761571 | 4.34E-09 | 1.99E-08 |
| FQU82_RS03575 | hisG | ATP phosphoribosyltransferase | -0.990039267 | 4.43E-09 | 2.03E-08 |
| FQU82_RS02260 | FQU82_RS02260 | UvrD-helicase domain-containing protein | 0.929874397 | 4.64E-09 | 2.12E-08 |
| FQU82_RS04970 | betB | betaine-aldehyde dehydrogenase | -0.888074512 | 4.66E-09 | 2.12E-08 |
| FQU82_RS15985 | FQU82_RS15985 | tRNA-Val | -2.333800384 | 4.66E-09 | 2.12E-08 |
| Novel00360 | - | PF00873:AcrB/AcrD/AcrF family | 2.157432096 | 4.80E-09 | 2.19E-08 |
| FQU82_RS10190 | FQU82_RS10190 | ABC transporter permease | 2.997438139 | 4.92E-09 | 2.24E-08 |
| FQU82_RS15080 | FQU82_RS15080 | FAD-binding protein | -0.898332177 | 5.14E-09 | 2.33E-08 |
| FQU82_RS02600 | FQU82_RS02600 | asparaginase | -1.038633914 | 5.44E-09 | 2.46E-08 |
| FQU82_RS02005 | FQU82_RS02005 | hypothetical protein | 1.431759813 | 5.52E-09 | 2.50E-08 |
| FQU82_RS06580 | csrA | carbon storage regulator CsrA | 1.147535883 | 5.68E-09 | 2.57E-08 |
| FQU82_RS02080 | ruvX | Holliday junction resolvase RuvX | 0.885001779 | 6.01E-09 | 2.71E-08 |
| FQU82_RS03940 | nuoF | NADH-quinone oxidoreductase subunit NuoF | -1.010128848 | 6.22E-09 | 2.81E-08 |
| FQU82_RS05015 | FQU82_RS05015 | YaeQ family protein | -2.542788723 | 6.25E-09 | 2.82E-08 |
| FQU82_RS14480 | FQU82_RS14480 | YbfB/YjiJ family MFS transporter | 3.313338874 | 7.45E-09 | 3.35E-08 |
| FQU82_RS01880 | FQU82_RS01880 | tRNA-Thr | -3.472325576 | 7.85E-09 | 3.52E-08 |
| FQU82_RS17675 | FQU82_RS17675 | TPM domain-containing protein | 1.394222411 | 7.87E-09 | 3.53E-08 |
| FQU82_RS03970 | nuoL | NADH-quinone oxidoreductase subunit L | -0.888653925 | 8.22E-09 | 3.68E-08 |
| FQU82_RS02520 | ptsP | phosphoenolpyruvate--protein phosphotransferase | 0.721025596 | 8.64E-09 | 3.87E-08 |
| FQU82_RS17210 | FQU82_RS17210 | metal-dependent hydrolase | 1.054715378 | 9.47E-09 | 4.23E-08 |
| FQU82_RS04965 | betA | choline dehydrogenase | -0.797774121 | 9.51E-09 | 4.24E-08 |
| FQU82_RS18645 | FQU82_RS18645 | DUF445 domain-containing protein | 0.92739927 | 9.55E-09 | 4.26E-08 |
| Novel00085 | - | PF02410:Ribosomal silencing factor during starvation | 2.674120232 | 1.03E-08 | 4.61E-08 |
| FQU82_RS03935 | nuoE | NADH-quinone oxidoreductase subunit NuoE | -0.909843299 | 1.06E-08 | 4.70E-08 |
| FQU82_RS04710 | ssrA | transfer-messenger RNA | 1.094354322 | 1.10E-08 | 4.90E-08 |
| FQU82_RS00245 | hpt | hypoxanthine phosphoribosyltransferase | -1.100131454 | 1.12E-08 | 4.96E-08 |
| Novel00256 | - | PF01434:Peptidase family M41|PF06480:FtsH Extracellular|PF00004:ATPase family associated with various cellular activities (AAA)|PF17862:AAA+ lid domain|PF01728:FtsJ-like methyltransferase | 1.124009752 | 1.12E-08 | 4.96E-08 |
| FQU82_RS05315 | FQU82_RS05315 | SRPBCC domain-containing protein | 1.920835677 | 1.13E-08 | 4.99E-08 |
| FQU82_RS00310 | FQU82_RS00310 | hypothetical protein | 0.842920548 | 1.14E-08 | 5.03E-08 |
| FQU82_RS04065 | FQU82_RS04065 | hypothetical protein | 0.769482848 | 1.22E-08 | 5.36E-08 |
| FQU82_RS07540 | FQU82_RS07540 | TolC family protein | -1.548828881 | 1.27E-08 | 5.60E-08 |
| FQU82_RS14990 | sppA | signal peptide peptidase SppA | -0.822093918 | 1.31E-08 | 5.76E-08 |
| FQU82_RS13975 | glnA | type I glutamate--ammonia ligase | -0.878594357 | 1.33E-08 | 5.83E-08 |
| FQU82_RS05435 | mrdA | penicillin-binding protein 2 | -1.058322504 | 1.33E-08 | 5.86E-08 |
| FQU82_RS07740 | FQU82_RS07740 | biotin carboxylase N-terminal domain-containing protein | -0.849195995 | 1.35E-08 | 5.91E-08 |
| FQU82_RS00930 | prpB | methylisocitrate lyase | -0.932297984 | 1.35E-08 | 5.91E-08 |
| FQU82_RS09645 | FQU82_RS09645 | ABC transporter permease | -1.411574812 | 1.35E-08 | 5.91E-08 |
| FQU82_RS00295 | purE | 5-(carboxyamino)imidazole ribonucleotide mutase | -1.018365797 | 1.39E-08 | 6.05E-08 |
| FQU82_RS14675 | FQU82_RS14675 | PaaI family thioesterase | 1.502873132 | 1.48E-08 | 6.46E-08 |
| FQU82_RS16135 | mutM | bifunctional DNA-formamidopyrimidine glycosylase/DNA-(apurinic or apyrimidinic site) lyase | 0.961951506 | 1.60E-08 | 6.98E-08 |
| FQU82_RS15735 | FQU82_RS15735 | DcaP family trimeric outer membrane transporter | -0.903652605 | 1.60E-08 | 6.98E-08 |
| FQU82_RS03145 | ilvC | ketol-acid reductoisomerase | -1.002698848 | 1.71E-08 | 7.43E-08 |
| FQU82_RS15155 | fdhD | formate dehydrogenase accessory sulfurtransferase FdhD | 1.600315196 | 1.87E-08 | 8.13E-08 |
| FQU82_RS05585 | FQU82_RS05585 | hypothetical protein | 0.704945124 | 1.97E-08 | 8.55E-08 |
| Novel00084 | - | PF00330:Aconitase family (aconitate hydratase)|PF00694:Aconitase C-terminal domain | 1.558370093 | 1.99E-08 | 8.61E-08 |
| FQU82_RS01675 | FQU82_RS01675 | PAS domain-containing sensor histidine kinase | 1.651003674 | 2.10E-08 | 9.09E-08 |
| FQU82_RS11690 | lpxA | acyl-ACP--UDP-N-acetylglucosamine O-acyltransferase | -0.882542476 | 2.12E-08 | 9.17E-08 |
| Novel00120 | - | PF00490:Delta-aminolevulinic acid dehydratase | 1.493075483 | 2.16E-08 | 9.31E-08 |
| FQU82_RS14530 | FQU82_RS14530 | peptidylprolyl isomerase | -0.787072706 | 2.16E-08 | 9.31E-08 |
| FQU82_RS11470 | FQU82_RS11470 | DUF2057 domain-containing protein | -2.224432056 | 2.28E-08 | 9.82E-08 |
| FQU82_RS09025 | FQU82_RS09025 | MetQ/NlpA family ABC transporter substrate-binding protein | 3.255001561 | 2.37E-08 | 1.02E-07 |
| FQU82_RS17795 | FQU82_RS17795 | hypothetical protein | 0.956958189 | 2.44E-08 | 1.05E-07 |
| FQU82_RS12470 | kdpB | potassium-transporting ATPase subunit KdpB | 1.628791216 | 2.69E-08 | 1.15E-07 |
| FQU82_RS12560 | FQU82_RS12560 | hypothetical protein | 2.291371715 | 2.87E-08 | 1.23E-07 |
| FQU82_RS17465 | FQU82_RS17465 | hypothetical protein | 0.901762034 | 3.01E-08 | 1.29E-07 |
| FQU82_RS14770 | mltG | endolytic transglycosylase MltG | -1.093056398 | 3.28E-08 | 1.40E-07 |
| FQU82_RS12340 | miaA | tRNA (adenosine(37)-N6)-dimethylallyltransferase MiaA | 1.015127616 | 3.55E-08 | 1.51E-07 |
| FQU82_RS03965 | nuoK | NADH-quinone oxidoreductase subunit NuoK | -0.792928688 | 3.56E-08 | 1.52E-07 |
| FQU82_RS12650 | FQU82_RS12650 | proteasome-type protease | 0.942490419 | 3.64E-08 | 1.55E-07 |
| FQU82_RS04695 | FQU82_RS04695 | EmrA/EmrK family multidrug efflux transporter periplasmic adaptor subunit | 0.766658908 | 3.64E-08 | 1.55E-07 |
| FQU82_RS17385 | FQU82_RS17385 | LysR family transcriptional regulator | 1.816389086 | 3.73E-08 | 1.59E-07 |
| FQU82_RS08735 | FQU82_RS08735 | malonate decarboxylase holo-ACP synthase | 3.467150869 | 3.85E-08 | 1.63E-07 |
| FQU82_RS13915 | FQU82_RS13915 | multifunctional CCA addition/repair protein | 0.776273266 | 3.90E-08 | 1.65E-07 |
| FQU82_RS09465 | msbA | lipid A export permease/ATP-binding protein MsbA | -0.758381205 | 4.05E-08 | 1.71E-07 |
| FQU82_RS18065 | FQU82_RS18065 | helix-turn-helix domain-containing protein | 2.192451177 | 4.05E-08 | 1.71E-07 |
| FQU82_RS09925 | FQU82_RS09925 | PepSY-associated TM helix domain-containing protein | -1.956206255 | 4.12E-08 | 1.74E-07 |
| FQU82_RS08650 | FQU82_RS08650 | LysR family transcriptional regulator | 1.601587155 | 4.22E-08 | 1.78E-07 |
| FQU82_RS18960 | FQU82_RS18960 | NAD(P)-dependent alcohol dehydrogenase | 0.8346078 | 4.24E-08 | 1.79E-07 |
| FQU82_RS08805 | tauA | taurine ABC transporter substrate-binding protein | 0.700618633 | 4.30E-08 | 1.81E-07 |
| FQU82_RS04715 | FQU82_RS04715 | tyrosine-type recombinase/integrase | 1.037568308 | 4.32E-08 | 1.81E-07 |
| FQU82_RS13190 | FQU82_RS13190 | thiazole synthase | -0.894529739 | 4.43E-08 | 1.86E-07 |
| FQU82_RS17800 | gltX | glutamate--tRNA ligase | -0.750153402 | 4.46E-08 | 1.87E-07 |
| FQU82_RS03280 | FQU82_RS03280 | ATP-binding protein | 0.832066688 | 4.56E-08 | 1.91E-07 |
| FQU82_RS00915 | dld | D-lactate dehydrogenase | 1.585302163 | 4.76E-08 | 1.99E-07 |
| FQU82_RS10075 | FQU82_RS10075 | helix-turn-helix domain-containing protein | 0.995793182 | 4.80E-08 | 2.00E-07 |
| FQU82_RS19035 | ppc | phosphoenolpyruvate carboxylase | 0.718395798 | 5.13E-08 | 2.14E-07 |
| FQU82_RS12640 | dnaB | replicative DNA helicase | -0.968612301 | 5.88E-08 | 2.45E-07 |
| FQU82_RS12510 | FQU82_RS12510 | acyl-CoA dehydrogenase family protein | 2.09797878 | 6.15E-08 | 2.56E-07 |
| FQU82_RS14925 | FQU82_RS14925 | NF038105 family protein | 1.949629293 | 6.22E-08 | 2.58E-07 |
| FQU82_RS12695 | fis | DNA-binding transcriptional regulator Fis | -1.135440259 | 6.25E-08 | 2.59E-07 |
| FQU82_RS00160 | FQU82_RS00160 | ATP-binding cassette domain-containing protein | -0.853024276 | 6.25E-08 | 2.59E-07 |
| FQU82_RS18380 | FQU82_RS18380 | putative porin | 0.67477348 | 6.32E-08 | 2.62E-07 |
| FQU82_RS10005 | FQU82_RS10005 | hypothetical protein | 1.404954919 | 6.46E-08 | 2.67E-07 |
| FQU82_RS14395 | FQU82_RS14395 | acyl-CoA desaturase | -1.091280258 | 6.67E-08 | 2.76E-07 |
| FQU82_RS02255 | FQU82_RS02255 | exodeoxyribonuclease V subunit gamma | 0.828138898 | 6.90E-08 | 2.85E-07 |
| FQU82_RS01250 | FQU82_RS01250 | OsmC family protein | 1.00586478 | 6.91E-08 | 2.85E-07 |
| Novel00101 | - | PF00317:Ribonucleotide reductase, all-alpha domain|PF02867:Ribonucleotide reductase, barrel domain|PF03477:ATP cone domain | 1.109270959 | 7.39E-08 | 3.04E-07 |
| FQU82_RS18430 | FQU82_RS18430 | 2-oxo acid dehydrogenase subunit E2 | -0.786495541 | 7.41E-08 | 3.05E-07 |
| FQU82_RS02920 | clpX | ATP-dependent Clp protease ATP-binding subunit ClpX | 0.788226134 | 8.00E-08 | 3.28E-07 |
| FQU82_RS08725 | FQU82_RS08725 | biotin-independent malonate decarboxylase subunit beta | 2.945375967 | 8.02E-08 | 3.29E-07 |
| FQU82_RS04355 | FQU82_RS04355 | DNA primase | 0.860023459 | 8.06E-08 | 3.30E-07 |
| FQU82_RS08225 | FQU82_RS08225 | aldehyde dehydrogenase | 1.343242584 | 8.18E-08 | 3.35E-07 |
| FQU82_RS09700 | FQU82_RS09700 | Dyp-type peroxidase | -0.861271633 | 8.30E-08 | 3.39E-07 |
| FQU82_RS10250 | FQU82_RS10250 | TIGR00366 family protein | 1.238280661 | 8.43E-08 | 3.44E-07 |
| FQU82_RS03980 | nuoN | NADH-quinone oxidoreductase subunit NuoN | -0.843696427 | 8.50E-08 | 3.47E-07 |
| FQU82_RS02580 | FQU82_RS02580 | lysozyme inhibitor LprI family protein | 1.154356864 | 8.58E-08 | 3.49E-07 |
| FQU82_RS09300 | FQU82_RS09300 | lecithin retinol acyltransferase family protein | 1.374235816 | 8.96E-08 | 3.64E-07 |
| FQU82_RS18165 | gabT | 4-aminobutyrate--2-oxoglutarate transaminase | 1.309616217 | 9.00E-08 | 3.66E-07 |
| Novel00121 | - | PF13437:HlyD family secretion protein|PF07690:Major Facilitator Superfamily | 1.499699182 | 9.67E-08 | 3.92E-07 |
| FQU82_RS03020 | rlmN | 23S rRNA (adenine(2503)-C(2))-methyltransferase RlmN | 0.670929935 | 9.75E-08 | 3.95E-07 |
| Novel00174 | - | PF03952:Enolase, N-terminal domain|PF00113:Enolase, C-terminal TIM barrel domain|PF00793:DAHP synthetase I family | -1.525972433 | 9.86E-08 | 3.99E-07 |
| FQU82_RS03065 | secB | protein-export chaperone SecB | 0.687350574 | 1.01E-07 | 4.07E-07 |
| FQU82_RS00225 | FQU82_RS00225 | metal/formaldehyde-sensitive transcriptional repressor | 1.955649063 | 1.01E-07 | 4.07E-07 |
| Novel00069 | - | PF00593:TonB dependent receptor | -1.770411094 | 1.04E-07 | 4.22E-07 |
| FQU82_RS11765 | adeN | multidrug efflux transcriptional repressor AdeN | 1.205461817 | 1.09E-07 | 4.37E-07 |
| FQU82_RS09860 | FQU82_RS09860 | PepSY-associated TM helix domain-containing protein | -1.628402389 | 1.09E-07 | 4.38E-07 |
| FQU82_RS09585 | zapE | cell division protein ZapE | -0.852660332 | 1.10E-07 | 4.42E-07 |
| FQU82_RS08220 | FQU82_RS08220 | LLM class flavin-dependent oxidoreductase | 1.614755494 | 1.11E-07 | 4.46E-07 |
| FQU82_RS17405 | FQU82_RS17405 | hypothetical protein | 2.375522409 | 1.14E-07 | 4.59E-07 |
| FQU82_RS01770 | FQU82_RS01770 | EamA family transporter | 2.702734474 | 1.15E-07 | 4.60E-07 |
| FQU82_RS11860 | moaCB | bifunctional molybdenum cofactor biosynthesis protein MoaC/MoaB | 1.049987016 | 1.16E-07 | 4.64E-07 |
| FQU82_RS04015 | FQU82_RS04015 | mechanosensitive ion channel | 1.254916941 | 1.20E-07 | 4.80E-07 |
| FQU82_RS02450 | FQU82_RS02450 | SDR family oxidoreductase | 1.865382169 | 1.25E-07 | 5.01E-07 |
| FQU82_RS03140 | ilvN | acetolactate synthase small subunit | -0.721840798 | 1.27E-07 | 5.08E-07 |
| FQU82_RS02200 | FQU82_RS02200 | monovalent cation/H+ antiporter subunit A | -0.657991856 | 1.30E-07 | 5.19E-07 |
| FQU82_RS01210 | FQU82_RS01210 | DedA family protein | -1.334479791 | 1.32E-07 | 5.25E-07 |
| FQU82_RS00220 | mnmE | tRNA uridine-5-carboxymethylaminomethyl(34) synthesis GTPase MnmE | -0.922459173 | 1.35E-07 | 5.38E-07 |
| FQU82_RS00185 | dnaN | DNA polymerase III subunit beta | -0.673977149 | 1.40E-07 | 5.56E-07 |
| Novel00007 | - | PF14849:YidC periplasmic domain|PF02096:60Kd inner membrane protein|PF10396:GTP-binding protein TrmE N-terminus|PF01809:Putative membrane protein insertion efficiency factor | -1.073077158 | 1.40E-07 | 5.57E-07 |
| FQU82_RS05305 | rubB | rubredoxin reductase RubB | -0.852554977 | 1.44E-07 | 5.70E-07 |
| FQU82_RS09385 | FQU82_RS09385 | TetR/AcrR family transcriptional regulator | -1.635313368 | 1.46E-07 | 5.76E-07 |
| FQU82_RS13875 | FQU82_RS13875 | acyl-CoA dehydrogenase | 0.663216161 | 1.47E-07 | 5.81E-07 |
| FQU82_RS18635 | FQU82_RS18635 | DUF924 family protein | 1.000007499 | 1.47E-07 | 5.81E-07 |
| FQU82_RS02105 | rlmB | 23S rRNA (guanosine(2251)-2'-O)-methyltransferase RlmB | -0.877661012 | 1.49E-07 | 5.89E-07 |
| FQU82_RS08450 | FQU82_RS08450 | TetR/AcrR family transcriptional regulator | 0.873535366 | 1.57E-07 | 6.19E-07 |
| FQU82_RS16895 | ybeY | rRNA maturation RNase YbeY | 0.964678186 | 1.63E-07 | 6.41E-07 |
| FQU82_RS19080 | FQU82_RS19080 | sodium-dependent transporter | 0.852424464 | 1.63E-07 | 6.41E-07 |
| FQU82_RS02275 | rpsO | 30S ribosomal protein S15 | -0.933559956 | 1.64E-07 | 6.44E-07 |
| FQU82_RS14775 | pabC | aminodeoxychorismate lyase | 1.195688576 | 1.65E-07 | 6.46E-07 |
| FQU82_RS04070 | apbC | iron-sulfur cluster carrier protein ApbC | 0.77841204 | 1.65E-07 | 6.47E-07 |
| Novel00155 | - | - | 3.515745309 | 1.74E-07 | 6.81E-07 |
| FQU82_RS18300 | FQU82_RS18300 | DUF485 domain-containing protein | -0.814311875 | 1.79E-07 | 6.98E-07 |
| Novel00002 | - | PF01521:Iron-sulphur cluster biosynthesis | 3.383740474 | 1.81E-07 | 7.07E-07 |
| FQU82_RS10630 | FQU82_RS10630 | GH3 auxin-responsive promoter family protein | -2.292948731 | 1.85E-07 | 7.23E-07 |
| FQU82_RS08245 | FQU82_RS08245 | NAD-dependent succinate-semialdehyde dehydrogenase | 1.74452106 | 1.93E-07 | 7.51E-07 |
| FQU82_RS02410 | FQU82_RS02410 | HopJ type III effector protein | -1.229748869 | 2.12E-07 | 8.25E-07 |
| FQU82_RS09555 | FQU82_RS09555 | integration host factor subunit beta | -0.634904802 | 2.29E-07 | 8.90E-07 |
| FQU82_RS18470 | FQU82_RS18470 | D-alanine--D-alanine ligase | -0.784362128 | 2.31E-07 | 8.99E-07 |
| FQU82_RS03330 | panB | 3-methyl-2-oxobutanoate hydroxymethyltransferase | -0.726288825 | 2.35E-07 | 9.10E-07 |
| FQU82_RS18450 | lpxC | UDP-3-O-acyl-N-acetylglucosamine deacetylase | -0.993690319 | 2.38E-07 | 9.22E-07 |
| FQU82_RS15980 | FQU82_RS15980 | tRNA-Asp | -2.580055776 | 2.55E-07 | 9.87E-07 |
| FQU82_RS12385 | FQU82_RS12385 | LysR family transcriptional regulator | 0.971872877 | 2.68E-07 | 1.03E-06 |
| FQU82_RS00300 | FQU82_RS00300 | 5-(carboxyamino)imidazole ribonucleotide synthase | -0.863582419 | 2.69E-07 | 1.04E-06 |
| FQU82_RS01760 | FQU82_RS01760 | hypothetical protein | 1.518207553 | 2.70E-07 | 1.04E-06 |
| FQU82_RS10400 | ppk1 | polyphosphate kinase 1 | 0.9527367 | 2.70E-07 | 1.04E-06 |
| FQU82_RS12665 | FQU82_RS12665 | circularly permuted type 2 ATP-grasp protein | 0.959313089 | 2.79E-07 | 1.07E-06 |
| FQU82_RS05190 | metH | methionine synthase | -0.83217681 | 2.83E-07 | 1.09E-06 |
| FQU82_RS04385 | coaD | pantetheine-phosphate adenylyltransferase | 0.782599732 | 3.16E-07 | 1.21E-06 |
| FQU82_RS18295 | FQU82_RS18295 | cation acetate symporter | -0.986032916 | 3.22E-07 | 1.24E-06 |
| Novel00257 | - | PF00117:Glutamine amidotransferase class-I|PF02786:Carbamoyl-phosphate synthase L chain, ATP binding domain|PF02787:Carbamoyl-phosphate synthetase large chain, oligomerisation domain|PF02142:MGS-like domain|PF01272:Transcription elongation factor, GreA/GreB, C-term|PF03449:Transcription elongation factor, N-terminal | -0.981681728 | 3.27E-07 | 1.25E-06 |
| FQU82_RS14905 | tolR | protein TolR | -1.847149631 | 3.38E-07 | 1.29E-06 |
| FQU82_RS03930 | nuoC | NADH-quinone oxidoreductase subunit C/D | -0.842256571 | 3.47E-07 | 1.33E-06 |
| FQU82_RS02295 | FQU82_RS02295 | DnaJ C-terminal domain-containing protein | 1.068264909 | 3.66E-07 | 1.40E-06 |
| FQU82_RS18660 | FQU82_RS18660 | SDR family NAD(P)-dependent oxidoreductase | 0.749147363 | 3.69E-07 | 1.41E-06 |
| FQU82_RS10385 | FQU82_RS10385 | isochorismatase family protein | -2.148177944 | 3.70E-07 | 1.41E-06 |
| FQU82_RS00910 | lldD | FMN-dependent L-lactate dehydrogenase LldD | 1.106830811 | 3.78E-07 | 1.44E-06 |
| FQU82_RS18160 | FQU82_RS18160 | NAD-dependent succinate-semialdehyde dehydrogenase | 0.770648012 | 3.91E-07 | 1.49E-06 |
| FQU82_RS13995 | FQU82_RS13995 | M1 family metallopeptidase | -0.963496126 | 4.01E-07 | 1.52E-06 |
| FQU82_RS16245 | FQU82_RS16245 | 3-hydroxyacyl-CoA dehydrogenase NAD-binding domain-containing protein | 0.826962077 | 4.07E-07 | 1.54E-06 |
| FQU82_RS05375 | FQU82_RS05375 | isocitrate lyase | 0.833714977 | 4.08E-07 | 1.54E-06 |
| FQU82_RS18600 | FQU82_RS18600 | YqiA/YcfP family alpha/beta fold hydrolase | 0.912811901 | 4.09E-07 | 1.55E-06 |
| FQU82_RS08935 | msrB | peptide-methionine (R)-S-oxide reductase MsrB | 0.736409292 | 4.39E-07 | 1.66E-06 |
| FQU82_RS17005 | FQU82_RS17005 | TRAP transporter large permease subunit | 0.822293421 | 4.43E-07 | 1.67E-06 |
| Novel00094 | - | PF02566:OsmC-like protein | 2.190561295 | 4.43E-07 | 1.67E-06 |
| FQU82_RS14845 | FQU82_RS14845 | CAP domain-containing protein | 1.965182133 | 4.46E-07 | 1.68E-06 |
| FQU82_RS00275 | FQU82_RS00275 | phosphatase PAP2 family protein | -2.325610448 | 4.51E-07 | 1.70E-06 |
| FQU82_RS00965 | FQU82_RS00965 | DUF4126 domain-containing protein | 0.938831219 | 4.56E-07 | 1.71E-06 |
| FQU82_RS16310 | folC | bifunctional tetrahydrofolate synthase/dihydrofolate synthase | -0.79566819 | 4.73E-07 | 1.78E-06 |
| FQU82_RS03335 | panC | pantoate--beta-alanine ligase | -0.775302802 | 4.89E-07 | 1.83E-06 |
| Novel00203 | - | PF01808:AICARFT/IMPCHase bienzyme|PF02843:Phosphoribosylglycinamide synthetase, C domain|PF01071:Phosphoribosylglycinamide synthetase, ATP-grasp (A) domain|PF02844:Phosphoribosylglycinamide synthetase, N domain | -1.240435143 | 5.08E-07 | 1.91E-06 |
| FQU82_RS16235 | FQU82_RS16235 | AraC family transcriptional regulator | 0.664170874 | 5.18E-07 | 1.94E-06 |
| FQU82_RS02310 | FQU82_RS02310 | monovalent cation:proton antiporter-2 (CPA2) family protein | 0.775927328 | 5.45E-07 | 2.04E-06 |
| FQU82_RS15925 | FQU82_RS15925 | patatin-like phospholipase family protein | 1.131424692 | 5.50E-07 | 2.06E-06 |
| Novel00124 | - | PF01106:NifU-like domain|PF07715:TonB-dependent Receptor Plug Domain | 2.181182935 | 5.52E-07 | 2.06E-06 |
| FQU82_RS01395 | def | peptide deformylase | 0.626166353 | 5.63E-07 | 2.10E-06 |
| FQU82_RS08200 | FQU82_RS08200 | GntR family transcriptional regulator | 1.148952822 | 5.72E-07 | 2.13E-06 |
| Novel00071 | - | PF11008:Protein of unknown function (DUF2846)|PF00574:Clp protease|PF07724:AAA domain (Cdc48 subfamily)|PF10431:C-terminal, D2-small domain, of ClpB protein|PF06689:ClpX C4-type zinc finger | 1.03452498 | 5.75E-07 | 2.14E-06 |
| FQU82_RS04140 | FQU82_RS04140 | biotin--[acetyl-CoA-carboxylase] ligase | -0.949868341 | 5.93E-07 | 2.21E-06 |
| FQU82_RS14035 | folE | GTP cyclohydrolase I FolE | -1.11766728 | 6.00E-07 | 2.23E-06 |
| FQU82_RS14150 | basB | acinetobactin non-ribosomal peptide synthetase subunit BasB | -1.900343309 | 6.04E-07 | 2.24E-06 |
| FQU82_RS08055 | paaA | 1,2-phenylacetyl-CoA epoxidase subunit A | 1.261506237 | 6.04E-07 | 2.24E-06 |
| Novel00217 | - | PF02777:Iron/manganese superoxide dismutases, C-terminal domain | 1.400549222 | 6.08E-07 | 2.25E-06 |
| FQU82_RS10300 | FQU82_RS10300 | 3-hydroxybutyrate dehydrogenase | 1.269201413 | 6.20E-07 | 2.29E-06 |
| FQU82_RS14210 | FQU82_RS14210 | carbon starvation CstA family protein | 0.709926951 | 6.28E-07 | 2.32E-06 |
| FQU82_RS05020 | FQU82_RS05020 | FKBP-type peptidyl-prolyl cis-trans isomerase | -1.349787404 | 6.40E-07 | 2.36E-06 |
| Novel00053 | - | PF13591:MerR HTH family regulatory protein | 2.573724605 | 6.47E-07 | 2.38E-06 |
| FQU82_RS16625 | trxC | thioredoxin TrxC | 0.772161088 | 6.78E-07 | 2.49E-06 |
| FQU82_RS11975 | FQU82_RS11975 | alpha/beta hydrolase | 0.886117029 | 6.94E-07 | 2.55E-06 |
| FQU82_RS12315 | FQU82_RS12315 | UDP-2,3-diacylglucosamine diphosphatase | -1.963827738 | 7.02E-07 | 2.58E-06 |
| FQU82_RS05360 | FQU82_RS05360 | CitMHS family transporter | -0.686459071 | 7.31E-07 | 2.68E-06 |
| FQU82_RS04900 | FQU82_RS04900 | LysR family transcriptional regulator | 1.916643885 | 7.74E-07 | 2.84E-06 |
| FQU82_RS02945 | FQU82_RS02945 | acetate kinase | -0.679124609 | 7.84E-07 | 2.87E-06 |
| FQU82_RS00010 | FQU82_RS00010 | plasmid replication DNA-binding protein | 1.070839574 | 8.08E-07 | 2.96E-06 |
| FQU82_RS05220 | FQU82_RS05220 | arylsulfatase | 1.414818207 | 8.21E-07 | 3.00E-06 |
| FQU82_RS04055 | FQU82_RS04055 | NF038215 family lipoprotein | 3.338545047 | 8.24E-07 | 3.01E-06 |
| FQU82_RS02285 | FQU82_RS02285 | magnesium transporter CorA family protein | 0.94887653 | 8.82E-07 | 3.22E-06 |
| FQU82_RS03250 | FQU82_RS03250 | NAD(P)(+) transhydrogenase (Re/Si-specific) subunit beta | -0.761440992 | 8.87E-07 | 3.23E-06 |
| FQU82_RS10220 | FQU82_RS10220 | aspartate ammonia-lyase | -1.074237749 | 9.00E-07 | 3.27E-06 |
| FQU82_RS02085 | FQU82_RS02085 | YqgE/AlgH family protein | 0.595365315 | 9.02E-07 | 3.28E-06 |
| FQU82_RS09715 | FQU82_RS09715 | HIT domain-containing protein | -0.641766996 | 9.43E-07 | 3.43E-06 |
| FQU82_RS15415 | FQU82_RS15415 | universal stress protein | 0.899858379 | 9.53E-07 | 3.46E-06 |
| FQU82_RS14415 | rapA | RNA polymerase-associated protein RapA | -0.674547803 | 9.59E-07 | 3.47E-06 |
| FQU82_RS12260 | FQU82_RS12260 | AAA family ATPase | -0.966459865 | 9.60E-07 | 3.47E-06 |
| FQU82_RS02525 | FQU82_RS02525 | RNA pyrophosphohydrolase | 0.755878641 | 9.96E-07 | 3.60E-06 |
| FQU82_RS07030 | FQU82_RS07030 | O-methyltransferase | 0.993779106 | 1.01E-06 | 3.64E-06 |
| FQU82_RS09790 | FQU82_RS09790 | acyl-CoA dehydrogenase family protein | 1.163174335 | 1.02E-06 | 3.67E-06 |
| FQU82_RS07660 | FQU82_RS07660 | MFS transporter | 1.976561467 | 1.05E-06 | 3.78E-06 |
| Novel00050 | - | PF02622:Uncharacterized ACR, COG1678|PF03652:Holliday junction resolvase | 1.245881496 | 1.12E-06 | 4.02E-06 |
| FQU82_RS05030 | pgaC | poly-beta-1,6-N-acetyl-D-glucosamine synthase | -0.789892214 | 1.21E-06 | 4.36E-06 |
| FQU82_RS02060 | FQU82_RS02060 | IclR family transcriptional regulator C-terminal domain-containing protein | 0.868825279 | 1.22E-06 | 4.37E-06 |
| FQU82_RS14655 | FQU82_RS14655 | pyridoxal phosphate-dependent aminotransferase | -0.694887303 | 1.26E-06 | 4.51E-06 |
| FQU82_RS15510 | sdhD | succinate dehydrogenase, hydrophobic membrane anchor protein | -0.811795437 | 1.26E-06 | 4.53E-06 |
| FQU82_RS18185 | FQU82_RS18185 | AzlC family ABC transporter permease | -0.985813023 | 1.28E-06 | 4.57E-06 |
| FQU82_RS03665 | rpe | ribulose-phosphate 3-epimerase | -0.674211602 | 1.31E-06 | 4.68E-06 |
| FQU82_RS15970 | FQU82_RS15970 | YdcF family protein | -3.042206897 | 1.33E-06 | 4.76E-06 |
| FQU82_RS18440 | FQU82_RS18440 | M23 family metallopeptidase | -1.052637602 | 1.35E-06 | 4.83E-06 |
| FQU82_RS16210 | FQU82_RS16210 | DUF934 domain-containing protei | -0.762619793 | 1.36E-06 | 4.84E-06 |
| FQU82_RS04390 | FQU82_RS04390 | YfhL family 4Fe-4S dicluster ferredoxin | 0.766006539 | 1.38E-06 | 4.92E-06 |
| FQU82_RS17220 | FQU82_RS17220 | acyl-CoA thioesterase II | 0.64791591 | 1.43E-06 | 5.11E-06 |
| FQU82_RS10625 | FQU82_RS10625 | TolC family protein | -2.428557513 | 1.44E-06 | 5.11E-06 |
| FQU82_RS18820 | FQU82_RS18820 | acyltransferase | -2.536382424 | 1.44E-06 | 5.11E-06 |
| FQU82_RS18480 | murG | undecaprenyldiphospho-muramoylpentapeptide beta-N-acetylglucosaminyltransferase | -0.832579037 | 1.46E-06 | 5.20E-06 |
| FQU82_RS14130 | bauB | siderophore-binding periplasmic lipoprotein BauB | -2.866126481 | 1.49E-06 | 5.28E-06 |
| FQU82_RS10975 | FQU82_RS10975 | FAD-dependent oxidoreductase | 1.317197329 | 1.49E-06 | 5.28E-06 |
| FQU82_RS15860 | FQU82_RS15860 | Maf-like protein | -1.154125242 | 1.52E-06 | 5.40E-06 |
| FQU82_RS07125 | FQU82_RS07125 | LysE family transporter | -1.624334826 | 1.58E-06 | 5.57E-06 |
| FQU82_RS08770 | cydB | cytochrome d ubiquinol oxidase subunit II | 1.161799488 | 1.60E-06 | 5.66E-06 |
| FQU82_RS04595 | argB | acetylglutamate kinase | -1.008640894 | 1.65E-06 | 5.82E-06 |
| FQU82_RS14435 | thiE | thiamine phosphate synthase | -1.30737016 | 1.71E-06 | 6.04E-06 |
| Novel00229 | - | PF00202:Aminotransferase class-III|PF00282:Pyridoxal-dependent decarboxylase conserved domain | 0.980841756 | 1.76E-06 | 6.19E-06 |
| FQU82_RS01515 | FQU82_RS01515 | TIGR00730 family Rossman fold protein | 0.79632965 | 1.81E-06 | 6.36E-06 |
| FQU82_RS19140 | FQU82_RS19140 | hypothetical protein | 0.991879938 | 1.83E-06 | 6.44E-06 |
| FQU82_RS17995 | hisF | imidazole glycerol phosphate synthase subunit HisF | -0.680411257 | 1.85E-06 | 6.50E-06 |
| FQU82_RS03345 | FQU82_RS03345 | HPr family phosphocarrier protein | 0.829072255 | 1.85E-06 | 6.51E-06 |
| FQU82_RS11040 | FQU82_RS11040 | TorF family putative porin | 0.638375666 | 1.88E-06 | 6.58E-06 |
| FQU82_RS18255 | FQU82_RS18255 | DUF1304 domain-containing protein | 0.773479832 | 1.90E-06 | 6.64E-06 |
| FQU82_RS00885 | pgi | glucose-6-phosphate isomerase | -0.880901927 | 1.90E-06 | 6.66E-06 |
| FQU82_RS14325 | sohB | protease SohB | 0.621291193 | 1.91E-06 | 6.68E-06 |
| FQU82_RS03945 | nuoG | NADH-quinone oxidoreductase subunit NuoG | -0.776074724 | 2.03E-06 | 7.09E-06 |
| FQU82_RS15595 | serS | serine--tRNA ligase | -0.765337453 | 2.04E-06 | 7.12E-06 |
| FQU82_RS16435 | FQU82_RS16435 | phospholipase D family protein | 0.66214699 | 2.06E-06 | 7.17E-06 |
| FQU82_RS03785 | FQU82_RS03785 | LysR substrate-binding domain-containing protein | 1.365984813 | 2.29E-06 | 7.97E-06 |
| FQU82_RS04535 | FQU82_RS04535 | DNA translocase FtsK | 0.59557787 | 2.33E-06 | 8.10E-06 |
| FQU82_RS11810 | fumC | class II fumarate hydratase | 0.608651697 | 2.39E-06 | 8.31E-06 |
| FQU82_RS03580 | hisD | histidinol dehydrogenase | -0.631562827 | 2.44E-06 | 8.46E-06 |
| FQU82_RS11955 | FQU82_RS11955 | iron-containing redox enzyme family protein | 0.829655935 | 2.46E-06 | 8.51E-06 |
| FQU82_RS04940 | FQU82_RS04940 | alpha/beta fold hydrolase | 1.173646127 | 2.49E-06 | 8.63E-06 |
| FQU82_RS03240 | FQU82_RS03240 | Re/Si-specific NAD(P)(+) transhydrogenase subunit alpha | -0.778590133 | 2.52E-06 | 8.73E-06 |
| Novel00048 | - | PF00378:Enoyl-CoA hydratase/isomerase|PF02737:3-hydroxyacyl-CoA dehydrogenase, NAD binding domain|PF00725:3-hydroxyacyl-CoA dehydrogenase, C-terminal domain|PF00108:Thiolase, N-terminal domain|PF02803:Thiolase, C-terminal domain | 0.897657473 | 2.55E-06 | 8.82E-06 |
| Novel00092 | - | PF00085:Thioredoxin | 1.524616295 | 2.57E-06 | 8.88E-06 |
| FQU82_RS04540 | rhtC | threonine export protein RhtC | 0.704580886 | 2.57E-06 | 8.88E-06 |
| FQU82_RS02435 | abeM | multidrug efflux MATE transporter AbeM | -0.922740205 | 2.61E-06 | 9.01E-06 |
| FQU82_RS09965 | FQU82_RS09965 | ABC transporter permease | 1.862842865 | 2.68E-06 | 9.21E-06 |
| FQU82_RS14420 | FQU82_RS14420 | RluA family pseudouridine synthase | -1.121339862 | 2.77E-06 | 9.52E-06 |
| FQU82_RS18640 | FQU82_RS18640 | hypothetical protein | 0.801988842 | 2.79E-06 | 9.60E-06 |
| FQU82_RS10640 | FQU82_RS10640 | DHA2 family efflux MFS transporter permease subunit | -2.91829332 | 2.84E-06 | 9.74E-06 |
| FQU82_RS09435 | FQU82_RS09435 | nucleotidyltransferase family protein | -0.830180764 | 2.87E-06 | 9.82E-06 |
| FQU82_RS14935 | FQU82_RS14935 | RNA methyltransferase | -1.390088127 | 3.08E-06 | 1.05E-05 |
| FQU82_RS17620 | ispH | 4-hydroxy-3-methylbut-2-enyl diphosphate reductase | 0.662238133 | 3.22E-06 | 1.10E-05 |
| FQU82_RS02650 | FQU82_RS02650 | SlyX family protein | 1.049270482 | 3.26E-06 | 1.11E-05 |
| FQU82_RS03975 | nuoM | NADH-quinone oxidoreductase subunit M | -0.830038703 | 3.36E-06 | 1.15E-05 |
| Novel00112 | - | - | 0.800654309 | 3.43E-06 | 1.17E-05 |
| FQU82_RS11950 | FQU82_RS11950 | MFS transporter | -2.344113756 | 3.50E-06 | 1.19E-05 |
| FQU82_RS16505 | FQU82_RS16505 | branched-chain amino acid transaminase | -0.692161459 | 3.55E-06 | 1.21E-05 |
| FQU82_RS12565 | pgaD | poly-beta-1,6-N-acetyl-D-glucosamine biosynthesis protein PgaD | 2.578086905 | 3.61E-06 | 1.23E-05 |
| FQU82_RS07010 | FQU82_RS07010 | glutathione binding-like protein | -0.770029022 | 3.64E-06 | 1.24E-05 |
| FQU82_RS00905 | lldR | transcriptional regulator LldR | 1.159361778 | 3.64E-06 | 1.24E-05 |
| FQU82_RS14085 | barB | acinetobactin export ABC transporter permease/ATP-binding subunit BarB | -1.991784588 | 3.66E-06 | 1.24E-05 |
| FQU82_RS13780 | FQU82_RS13780 | ABC transporter permease | -0.969808168 | 3.74E-06 | 1.27E-05 |
| FQU82_RS14920 | pal | peptidoglycan-associated lipoprotein Pal | -0.729314612 | 3.88E-06 | 1.31E-05 |
| FQU82_RS18595 | parE | DNA topoisomerase IV subunit B | 0.695204736 | 4.01E-06 | 1.36E-05 |
| FQU82_RS19055 | FQU82_RS19055 | hypothetical protein | 0.767570171 | 4.01E-06 | 1.36E-05 |
| FQU82_RS17770 | FQU82_RS17770 | UDP-N-acetylmuramoyl-L-alanyl-D-glutamate--2,6-diaminopimelate ligase | -0.706051696 | 4.15E-06 | 1.40E-05 |
| Novel00026 | - | PF00958:GMP synthase C terminal domain | -1.155883761 | 4.31E-06 | 1.45E-05 |
| FQU82_RS15475 | lpdA | dihydrolipoyl dehydrogenase | -0.919911496 | 4.32E-06 | 1.45E-05 |
| FQU82_RS11315 | FQU82_RS11315 | hypothetical protein | -0.749406201 | 4.43E-06 | 1.49E-05 |
| FQU82_RS04155 | smc | chromosome segregation protein SMC | -0.86243682 | 4.45E-06 | 1.49E-05 |
| FQU82_RS02395 | FQU82_RS02395 | TIGR03862 family flavoprotein | -1.898390746 | 4.69E-06 | 1.58E-05 |
| FQU82_RS16890 | FQU82_RS16890 | PhoH family protein | 0.664875946 | 4.72E-06 | 1.58E-05 |
| FQU82_RS19085 | FQU82_RS19085 | TIGR04219 family outer membrane beta-barrel protein | -1.198774976 | 4.79E-06 | 1.61E-05 |
| FQU82_RS16940 | FQU82_RS16940 | fumarylacetoacetate hydrolase family protein | -0.745843897 | 4.90E-06 | 1.64E-05 |
| FQU82_RS05265 | FQU82_RS05265 | FtsX-like permease family protein | 0.869064657 | 4.91E-06 | 1.64E-05 |
| FQU82_RS14995 | FQU82_RS14995 | lysophospholipid acyltransferase family protein | -0.948654029 | 4.92E-06 | 1.64E-05 |
| FQU82_RS18895 | FQU82_RS18895 | phosphoribosylaminoimidazolesuccinocarboxamide synthase | -0.834749865 | 5.15E-06 | 1.72E-05 |
| FQU82_RS01680 | FQU82_RS01680 | response regulator | 0.769245123 | 5.18E-06 | 1.73E-05 |
| FQU82_RS13455 | FQU82_RS13455 | tape measure protein | 1.028632854 | 5.39E-06 | 1.80E-05 |
| FQU82_RS13085 | rpsU | 30S ribosomal protein S21 | -0.74356566 | 5.45E-06 | 1.82E-05 |
| FQU82_RS09685 | FQU82_RS09685 | nucleoside-diphosphate sugar epimerase | 1.35221595 | 5.50E-06 | 1.83E-05 |
| FQU82_RS17745 | ponA | penicillin-binding protein PBP1a | -0.609386943 | 5.78E-06 | 1.92E-05 |
| FQU82_RS10150 | FQU82_RS10150 | AraC family transcriptional regulator | 1.461154707 | 5.79E-06 | 1.92E-05 |
| FQU82_RS18965 | FQU82_RS18965 | PLP-dependent aminotransferase family protein | 0.862850601 | 6.11E-06 | 2.03E-05 |
| Novel00079 | - | PF02556:Preprotein translocase subunit SecB|PF00581:Rhodanese-like domain|PF00462:Glutaredoxin | 1.647754063 | 6.12E-06 | 2.03E-05 |
| FQU82_RS01415 | FQU82_RS01415 | oxygenase MpaB family protein | -1.658526537 | 6.12E-06 | 2.03E-05 |
| FQU82_RS07515 | FQU82_RS07515 | KpsF/GutQ family sugar-phosphate isomerase | -0.664218236 | 6.26E-06 | 2.07E-05 |
| FQU82_RS07520 | FQU82_RS07520 | HAD-IIIA family hydrolase | -0.926065074 | 6.41E-06 | 2.12E-05 |
| FQU82_RS04115 | FQU82_RS04115 | hypothetical protein | 1.449369634 | 6.41E-06 | 2.12E-05 |
| FQU82_RS17135 | rplP | 50S ribosomal protein L16 | -2.154104805 | 6.73E-06 | 2.22E-05 |
| FQU82_RS16065 | FQU82_RS16065 | efflux RND transporter periplasmic adaptor subunit | -1.216326424 | 6.76E-06 | 2.23E-05 |
[truncated: 114,620 more chars]
